# “Vermellogens” and the Development of CB[8]-Based Supramolecular Switches Using pH-Responsive and Non-Toxic Viologen Analogues

**DOI:** 10.1021/jacs.2c08575

**Published:** 2022-10-07

**Authors:** Liliana Barravecchia, Arturo Blanco-Gómez, Iago Neira, Raminta Skackauskaite, Alejandro Vila, Ana Rey-Rico, Carlos Peinador, Marcos D. García

**Affiliations:** † Departamento de Química and Centro de Investigaciones Científicas Avanzadas (CICA), Facultad de Ciencias, Universidade da Coruña, 15071A Coruña, Spain; ‡ Gene & Cell Therapy Research Group (G-CEL), Centro de Investigaciones Científicas Avanzadas (CICA), Universidade da Coruña, 15071A Coruña, Spain

## Abstract

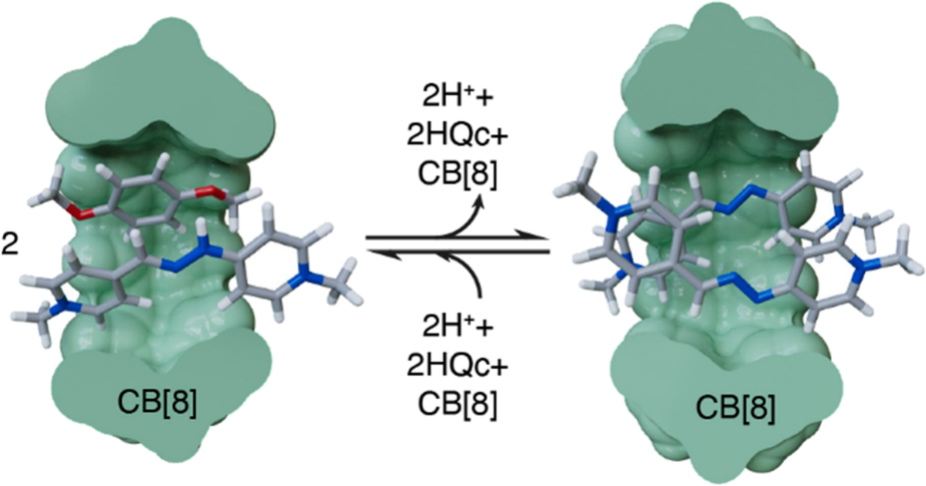

We present herein the “vermellogens”, a new class of pH-responsive viologen analogues, which replace the direct linking between *para*-substituted pyridinium moieties within those by a hydrazone functional group. A series of such compounds have been efficiently synthesized in aqueous media by hydrazone exchange reactions, displaying a marked pH-responsivity. Furthermore, the parent *N*,*N*′-dimethylated “vermellogen”: the “red thread”, an analogue of the herbicide paraquat and used herein as a representative model of the series, showed anion-recognition abilities, non-reversible electrochemical behavior, and non-toxicity of the modified bis-pyridinium core. The host–guest chemistry for the “red thread” with the CB[7,8] macrocyclic receptors has been extensively studied experimentally and by dispersion corrected density functional theory methods, showing a parallel behavior to that previously described for the herbicide but, crucially, swapping the well-known redox reactive capabilities of the viologen-based inclusion complexes by acid–base supramolecular responsiveness.

## Introduction

Supramolecular switches are non-covalently bonded complexes, able to translate the structural swapping abilities of the components from the molecular to the supramolecular level. Therefore, these species can be thought as composed of two or more self-assembled units, whose association can be transiently controlled by external stimuli, such as light, electrical potential, or chemical effectors.^1−5^ By doing so, these compounds interconvert between structurally different equilibrium states, sequentially using divergent energy inputs without the production of net mechanical work.^6^ In this context, supramolecular switches have arisen as controlling units in a myriad of currently relevant practical applications, such as, among others, the development of artificial molecular machines,^6,7^ supramolecular drug delivery systems,^8^ or controllable catalysis.^9^

**Scheme 1:**
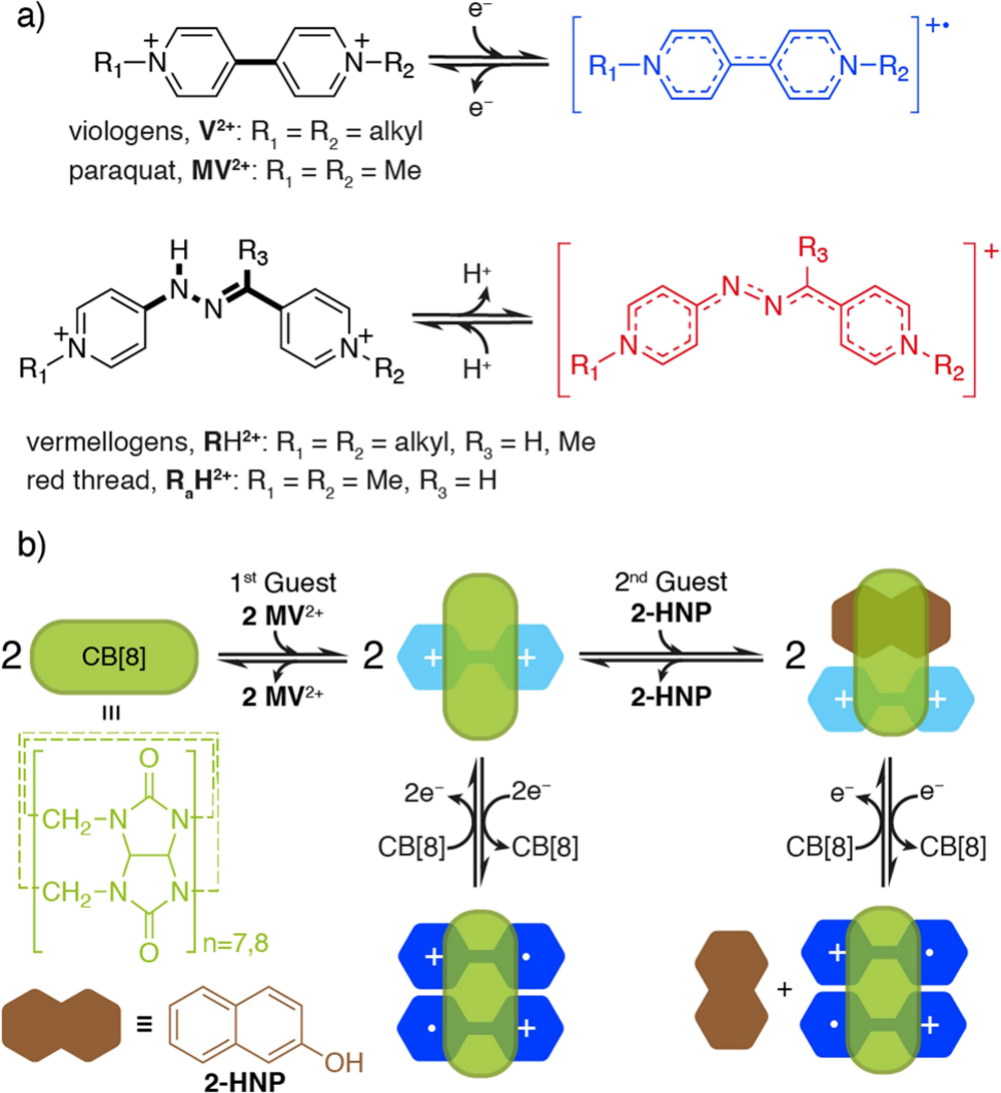
(a) Redox-Responsive Viologens **V**^2+^ and Its Parent Compound Paraquat **MV**^2+^; pH-Responsive “Vermellogens” **R**H^2+^ and Its Parent Derivative “Red Thread” **R_a_**H^2+^; (b) Redox-Responsive CB[7,8]-**V**^2+^ Host–Guest Chemistry

Within the context of macrocyclic host–guest chemistry, the conjunction of the curcurbit[*n*]uril family of hosts (CB[*n*]s,^10^ i.p. CB[7,8]),^11^ and viologens as guests (**V**^2+^, salts derived from the dialkylation of 4,4-bipyridine),^12^ is a paradigmatic example of supramolecular switches (Scheme 1a).^13,14^ Although both CB[7,8] form 1:1 binary complexes with **V**^2+^, optimizing cation–dipole interactions with the two carbonyl-based portals of the hosts, CB[8] is one of the few receptors that can form 1:2 heteroternary complexes with a suitable electron donor as the second guest (e.g., dihydroxynaphthalenes). In this case, the otherwise non-complexed second guest is able to enter the cavity of the host, establishing enhanced donor–acceptor interactions with the bis-pyridinium first guest.^15^ Furthermore, the well-known behavior of viologens as redox switches^16,17^ can be translated from the molecular to the supramolecular level, as electrochemical stimulation can reversibly push the host–guest complex from its original 1:1 arrangement with the dicationic form of the guest **V**^2+^ to an homoternary configuration provoked by a strong radical pairing of two **V**^+·^moieties (Scheme 1b).^13,14,18^ The highly convenient qualities of CB[*n*]s as hosts (commercial availability, low reactivity and toxicity, and adequate solubility in aqueous media),^10^ and those of viologens (synthetic accessibility, tunability, etc.), have spurred the development of a myriad of supramolecular switches based on CB[7,8]:**V**^2+^ systems.^13,14^ Nevertheless, a key factor considerably limits the applicability of these systems in the context of biologically relevant milieus:^19^ the high cytotoxicity of viologens as producers of oxygenated reactive species.^20^

Following our continuous interest in the supramolecular chemistry of pyridinium salts,^21^ we have recently reported a series of hydrazone-based analogues of the **V**^2+^-containing host “blue box”,^22−24^ developed by Stoddart and co-workers,^25^ in which the two viologen moieties within the model macrocycle are replaced by hydrazones linking the pyridinium rings. In these reports, we have not only found the pseudoviologen moieties acting as archetypic electron acceptors but, additionally, those behaving as acid–base responsive motives, because of an anomalous p*K*_a_ for the imine protons. Moved by these findings, as well as our interest in the host–guest chemistry of cucurbit[*n*]urils,^13,14^ we present herein an in-depth study of the cation (*E*)-1-methyl-4-((2-(1-methylpyridin-1-ium-4-yl)hydrazineylidene)methyl)pyridin-1-ium (the “vermellogen” “red thread”, **R_a_**H^2+^, Scheme 1b),^26^ as a model of pH-responsive non-toxic viologen-like guest for the development of CB[7,8]-based supramolecular switches.

## Results and Discussion

### Synthesis, Characterization, and Stimuli-Responsive Properties of the “Vermellogens”

As for viologens,^12,16,17^ the most straightforward methodology for the synthesis of the “vermellogens”^26^ would consist of the per- or sequential alkylation of an appropriate bis-pyridine precursor.^27,28^ Nevertheless, this approach was found not very satisfactory, especially when applied to asymmetrically substituted analogues (**R_c,d_**H·2X, Table 1), as the attempted monoalkylation of the precursor yielded complex mixtures of products. Alternatively, considering the high hydrolytic stability of these hydrazone-linked bis-pyridinium salts,^22−24^ we tackled their synthesis by hydrazone exchange in acidic water of the already alkylated intermediates **3_a–b_**·I and **4_a–c_**·I. Typically, a mixture of the matching aldehyde/ketone and hydrazone was reacted for 24 h at 60 °C, and the corresponding mixture was cooled down and saturated with solid KPF_6_, producing the precipitation of **R_a–e_**H·2PF_6_. Water-soluble chloride salts were obtained in good overall yields by ion metathesis with TBACl, with no significant impurities being observed for the crude reaction products by HPLC-UV-MS.^28^

**Table 1:**
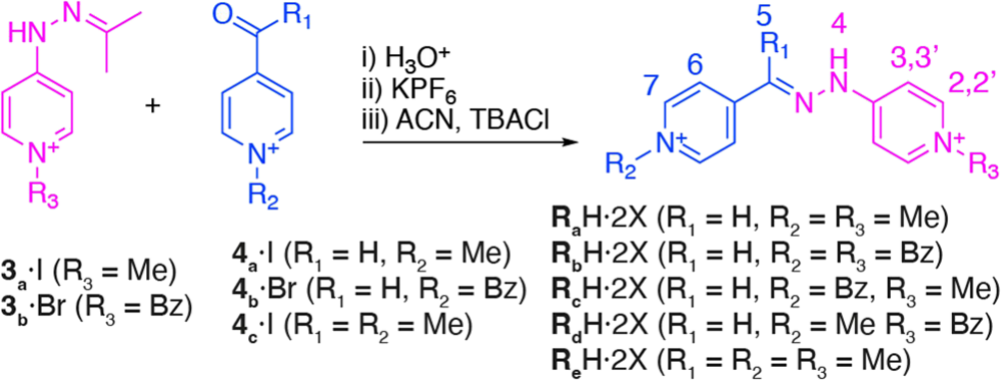
Synthesis of “Vermellogens” **R_a–e_**H·2X and **M_a,b_**H·X, Including Selected Physicochemical Properties

comp.	yield (%)^a^	Δ*G*^#^_rot_^b^	λ^1^_max_	λ^2^_max_	p*K*_a_^c^
**R_a_**H·2X	90/93	14.7	369	465	9.0
**R_b_**H·2X	72/51	14.6	377	474	8.6
**R_c_**H·2X	79/91	14.5	375	475	8.7
**R_d_**H·2X	98/85	14.7	372	466	8.6
**R_e_**H·2X	74/96	14.4	366	460	9.6
**M_a_**H·X	86/72	15.9	336	376	10.7
**M_b_**H·X	92/87		420	506	>13

a X = PF_6_^–^/Cl^–^.

b CD_3_CN.

c **R**H^2+^/**R**^+^ or **M**H^+^/**M**.

**Figure 1:**
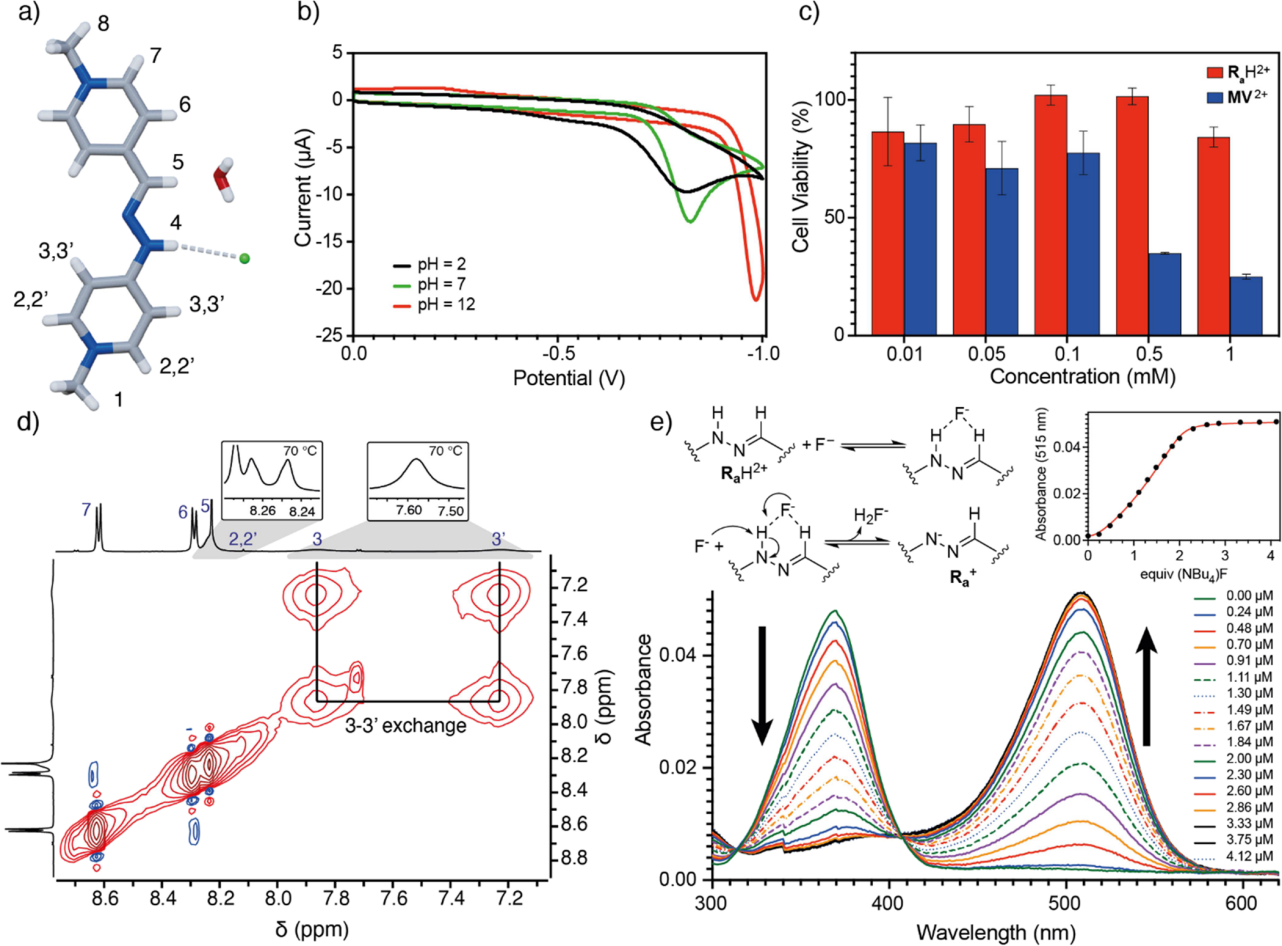
(a) Stick representation of **R_a_**H·2Cl obtained from single-crystal X-ray diffraction analysis. Color code: Carbon, gray; nitrogen, blue; oxygen, red; chloride, green; hydrogen, white. N-H···Cl hydrogen bonding is represented as white dotted lines; (b) Cyclic voltammogram for **R_a_**H·2Cl at 2 mM in aqueous solution at pH 2 (black), pH 7 (green), and pH 12 (red); (c) Viability of HFF-1 cells upon contact with different concentrations (0.01, 0.05, 0.1, and 0.5 mM) of **R_a_**H·2Cl and paraquat **MV**·2I; (d) Partial EXSY/NOESY NMR spectra (CD_3_CN, 500 MHz) for **R_a_**H·2PF_6_, showing key exchange peaks between H_3_ and H_3′_. Inset: partial ^1^H-NMR spectra at 70 °C showing the collapse of H_2_/H_2′_ and H_3_/H_3′_. (e) UV–vis spectra for the titration of 1 μM **R_a_**H·2PF_6_ solution with TBAF in acetonitrile. Insets: a proposed mechanism for the fluoride-assisted deprotonation and fitting of the UV–vis titration data.

The obtained compounds were fully characterized by 1D/2D NMR techniques, both in CD_3_CN (**R_a–e_**H·2PF_6_) and D_2_O (**R_a–e_**H·2Cl), showing data in good agreement with that expected.^28^ A quite unusual, but well-known,^22−24,29−32^ common feature was observed in all cases, with the restricted rotation around the NH-C*sp*^2^(py) bond within the hydrazinylpyridinium moiety resulting in the non-equivalence of the protons on the upper and lower sides of the heterocycle, which appear in a near-coalescence situation, exchanging moderately slow on the NMR-timescale (e.g., **R_a_**H·2PF_6_, Figure 1d). This end was demonstrated both by the exchange peaks found on the corresponding NOESY/EXSY experiments and by VT ^1^H-NMR (inset Figure 1d). Typically, the later technique showed the swapping to a situation of quick exchange upon heating for signals H_2/2′_ and H_3/3′_, which in turn allowed for the calculation of Δ*G*^#^_rot_ ∼ 15 kcal/mol for the impeded rotations (Table 1). Furthermore, ^1^H-NMR experiments recorded in buffered solutions at pD = 12 showed two interesting features: the shielding of the signals of the compound, as it would be expected for the deprotonation of the NH moiety and the consequent loss of one positive charge, and the quick deuteration of the H_7_ hydrogens of the pyridinium ring closer to iminic bond (Figure S6).^33^ Regarding the ESI-MS data for the compounds, intense peaks were observed corresponding to the loss of H^+^PF_6_^–^, in good agreement with the expected unusual acidity of the amine protons in these compounds.^28^

Finally, diffraction-grade single crystals for **R_a_**H·2Cl could be obtained, with the solid-state structure showing hydrogen bonding between the hydrazone moiety with a chloride counterion and a crystallization water (Figure 1a).^34^ Moved by this observation, and other reported evidence regarding the anion binding abilities of this type of hydrazone moieties,^30,35^ we decided to study the ability of the “red thread” **R_a_**H^2+^ to recognize halide anions in acetonitrile by UV–vis titrations and ^1^H-NMR. For I^–^, no interaction with **R_a_**H·2PF_6_ was observed. Conversely, the addition of increasing amounts of TBACl/Br to 33–20 μM solutions of **R_a_**H·2PF_6_, led to the absorption bands associated with the free form of **R_a_**H^2+^ at λ_max_ = 370 nm, decreasing in favor of new bands with a slight shift to λ_max_ = 378 and 370 nm, tentatively assigned to **R_a_**H^2+^···Cl/Br complexes and with the data fitting appropriately to 1:1 association processes (*K*_a_ = (7.03 ± 0.14)·10^4^ M^–1^ for Cl^–^, (1.41 ± 0.07)·10^4^ M^–1^ for Br^–^). In both cases, the results agreed with those expected for the recognition through hydrogen bonding between the hydrazone group and the anions, resulting in a bathochromic shift of the absorption band.^30,35^ Further evidence of the interaction was observed on the ^1^H-NMR for **R_a_**H·2PF_6_ in CD_3_CN (Figures S112 and S114), showing the characteristic deshielding of the iminic signal upon addition of increasing amounts of the corresponding halide. In the case of the UV–vis titration of **R_a_**H·2PF_6_ (1 μM) with TBAF, the results obtained were quite different, with the band at 370 nm decreasing with the concomitant development of a new one at λ_max_ = 515 nm, associated with the deprotonated **R_a_**^+^ form of the cation (vide infra). The obtained data fitted in this case to a 2:1 process (Figure 1e), with the first F^–^ equivalent establishing a strong interaction with the hydrazone group (*K*_a1_ = (4.49 ± 0.77)·10^7^ M^–1^), followed by deprotonation of the NH assisted by a second fluoride (*K*_a2_ = (5.43 ± 0.59)·10^7^ M^–1^).^34−37^

Next, in our study of the stimuli-responsiveness of the “vermellogens”, we proceeded to verify the new analogues as pH-based molecular switches, by conducting UV–vis acid–base titrations in water for the compounds **R_a–e_**H·2Cl. All the new “vermellogens” show similar π–π* main absorption bands centered at λ^1^_max_ = 366–377 nm at neutral or slightly acidic pH. An increase in the basicity of the solution produces the decrease of the aforementioned bands and the concomitant rise of new absorptions associated with the deprotonated compounds and centered at λ^2^_max_ = 460–475 nm. The p*K*_a_ values obtained by the aforementioned UV–vis titrations for **R_a–e_**H·2Cl are quite similar, showing no dependence on the substituent on the pyridinium N^+^ atoms within their structures (Table 1 and Figures S115–S125). Furthermore, to evaluate the effect of the two different pyridinium heterocycles on the observed anomalous p*K*_a_’s, two analogues were prepared (**M_a–b_**H·Cl), in which one of these moieties was substituted by a neutral phenyl ring. In this case, accounting for the potentially high degree of adjustability on the iminic acidity of these compounds, while the p*K*_a_ increases slightly for **M_a_**H·Cl compared with the other bis-pyridinium derivatives prepared, that estimated for **M_b_**H·Cl was larger than 13 p*K*_a_ units, establishing the hydrazynyl-pyridinium moiety as mainly responsible for the anomalously decreased p*K*_a_ of the “vermellogens”.^28^

To conclude with this part of our work on the molecular responsiveness of the “vermellogens,” we decided to substantiate whether the bis-pyridinium core in the compounds would have or not a viologen-like reversible redox behavior. Thus, cyclic voltamograms were recorded for **R_a_**H·2Cl (2 mM) in buffered aqueous solutions at pH = 2 (0.05 M H_3_PO_4_/NaH_2_PO_4_), 7 (0.05 M NaH_2_PO_4_/Na_2_HPO_4_), and 12 (0.05 M Na_2_HPO_4_/Na_3_PO_4_). Those shown both the acidic (**R_a_**H^2+^) and basic (**R_a_**^+^) forms of the “red thread” owning non-reversible redox peaks (Figure 1b). Motivated by this observation, contrary to that exhibited by viologens and responsible for their known toxicity as redox-cyclers, we proceeded to obtain cytotoxic profiles in a human fibroblastic cell line for **R_a_**H^2+^, comparing the results with those for paraquat (**MV**^2+^, Figure 1c).^28^ Percentages of cell survival in the presence of the two substances were estimated, with those incubated with **R_a_**H^2+^ always showing higher levels of viability, even with concentrations as large as 0.5 mM (∼90%; *p* ≥ 0.82), and only with a slight reduction on cell survival noted at the highest concentration tested (1 mM). In sharp contrast, incubation of cells with **MV**^2+^ led, as previously reported,^38^ to a severe decrease in cell survival from 0.5 mM concentration (*p* ≤ 0.001), leading to a ∼3-fold decrease of those percentages obtained with **R_a_**H^2+^ (*p* < 0.0002).

### Host–Guest Chemistry with CB[7,8]: From Molecular to Supramolecular Switches by pH-Stimulation

**Scheme 2:**
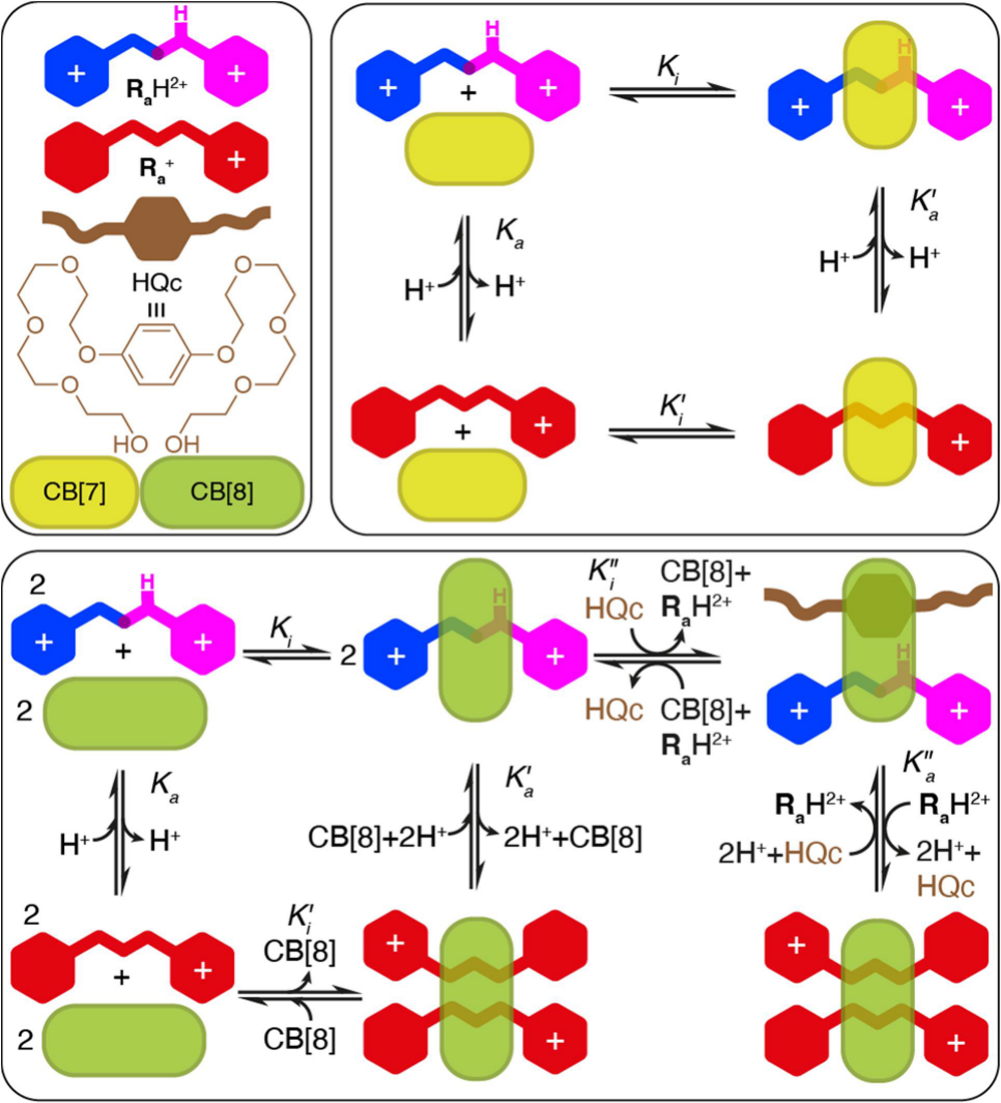
Schematic Representation of the Acid–Base and Complexation Processes Discussed in This Work Top: CB[7]; bottom: CB[8].

Once the stimuli-responsiveness of the “vermellogens” was explored, we moved our attention to the host–guest chemistry of the model compound “red thread” **R_a_**H^2+^ and the CB[7,8] macrocycles (Scheme 2). As shown, we envisioned not only to study the formation of binary complexes with the receptors but also the self-assembly of homo and heteroternary complexes. Hence, in the case of CB[8], we planned to use as a second guest the hydroquinone derivative HQc, a prototypical electron donor with an appropriate water solubility for the determination of the association constants (*K*_i_s), and a non-interfering nature of the −OH groups on the acid/base-modulated complexation processes. In this regard, Scheme 2 shows the expected pH-responsiveness of the inclusion complexes and the thermodynamic cycles correlating the acidity of the complexed and non-complexed “red thread” (*K*_a_s), with the association processes (*K*_i_s).

**Figure 2:**
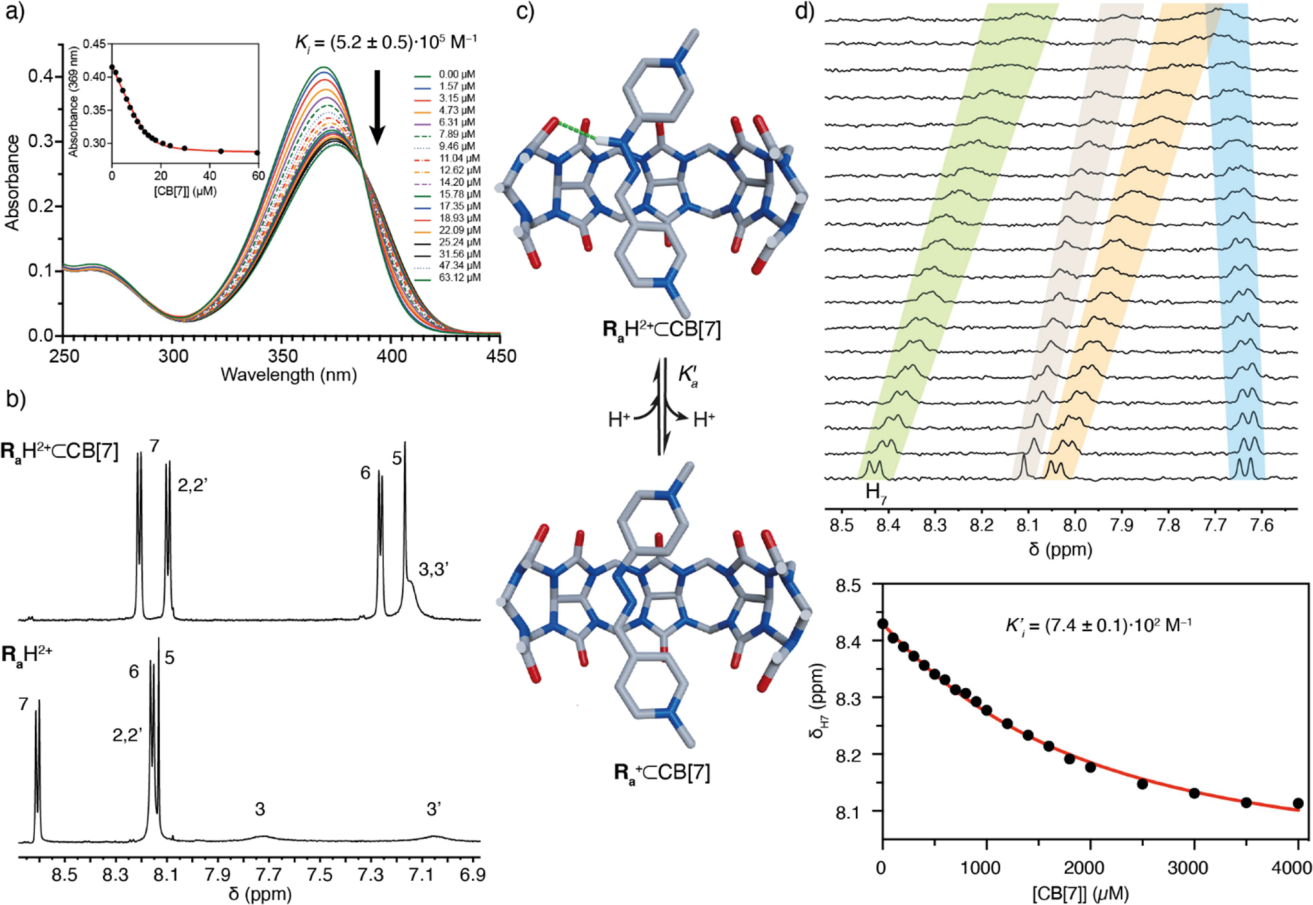
Relevant data for the formation of **R_a_**H^2+^/**R_a_**^+^ ⊂ CB[7]. (a) UV–vis spectra for the titration of 15.8 μM **R_a_**H·2Cl solution with CB[7] in buffered aqueous solution at pH = 7. Inset: Fitting of the UV–vis titration data; (b) Partial ^1^H-NMR spectra (500 MHz, D_2_O) for: top, equimolecular 2.5 mM mixture of **R_a_**H·2Cl and CB[7], bottom, **R_a_**H·2Cl. (c) Schematic representation of the **R_a_**H^2+^ ⊂ CB[7] ⇌ **R_a_**^+^ ⊂ CB[7] + H^+^ equilibrium, using stick representations for the structures of the local minima found on the potential energy surface for the inclusion complexes by DFT-D methods (color code as in Figure 1). (d) ^1^H-NMR (400 MHz, D_2_O) titration experiments for **R_a_**^+^ ⊂ CB[7] (top), and fitting of the NMR titration data to a 1:1 isotherm (bottom).

Consequently, we first evaluated by ^1^H-NMR the simplest of the cases: the complexation between **R_a_**H·2Cl and CB[7], using buffered aqueous media at pD = 7 to ensure the complete protonation of the substrate. The obtained results were in good agreement with the complexation taking place, with signals for the interacting species appearing in the spectra in a situation of rapid exchange in the NMR timescale, and diffusing as a whole in the subsequent DOSY experiment (Figure S141). In essence (Figure 2b), a substantial shielding of the guest signals is observed, attributable to the expected binding mode resulting from the **R_a_**H^2+^ core inserted within the cavity of the host. This end was validated by the complete assignment of the ^1^H signals of the species aided by 1D/2D NMR experiments, with complexation-induced shifts being less pronounced in the case of the pyridinium ring closer to the NH moiety.^39^ This fact suggests a binding mode with a significant displacement of the guest from the center of mass of the free host, which in turn could be explained by a potential hydrogen bonding between the acidic NH group of the guest and the carbonyl-laced portals of the macrocycle (vide infra). Additionally, HR ESI-MS experiments pointed out the formation of the expected binary complex **R_a_**H^2+^ ⊂ CB[7] (*m/z* = 695.2405 found for M^2+^, calculated: 695.2400). Finally, UV–vis titrations allowed for the assessment of the association constant for **R_a_**H^2+^ ⊂ CB[7] as *K*_i_ = (5.2 ± 0.5)·10^5^ M^–1^,^40,41^ due to the modification of the main absorption band of the guest upon complexation by the macrocycle (Figure 2a and Table 2). As would be expected, this value is in good agreement with that previously reported by Kaifer and Ong for the inclusion complex **MV**^2+^ ⊂ CB[7] at pH = 7.2.^42^

**Table 2:** Thermodynamic Data and Geometrical Parameters for Complexes

guest ⊂ host	Δ*G*_exp_^a^ (kcal/mol)	Δ*G*_DFT_^43^ (kcal/mol)	D^40^^e^	Pc^51^ (%)
**R_a_**H^2+^ ⊂ CB[7]	–7.8	–8.3	1.4	37
**R_a_**^+^ ⊂ CB[7]	–3.9	–2.5	1.0	36
**R_a_**H^2+^ ⊂ CB[8]	–7.3^b^^c^	–4.6	1.5	28
(**R_a_**^+^)_2_ ⊂ CB[8]	–10.5^b^^c^	–16.6	1.2, 1.2	51
**R_a_**^+^ ⊂ CB[8]	--	–2.2	1.0	27
**R_a_**H^2+^·HQ ⊂ CB[8]	–11.4	–13.3	1.4	51
**R_a_**^+^·HQ ⊂ CB[8]		–10.7	1.2	52
**MV**^2+^ ⊂ CB[7]	–7.3^d^	–8.7	1.0	44
**MV**^2+^ ⊂ CB[8]	–6.9^c^	–5.7	1.0	32

a Δ*G*_exp_ = – *RT*Ln*K*_i_ , calculated at *T* = 298.15 K using the association constants (*K*_i_s) discussed in the text.

b Estimated by NMR competition experiments with **MV**^2+ .^

c Considering *K*_i_ (**MV**^2+^⊂CB[8]) = 1.1 10^5^ M^–1^.^18^

d Considering *K*_i_ (**MV**^2+^⊂CB[7]) = 2.2 10^5^ M^–1^.^42^

e
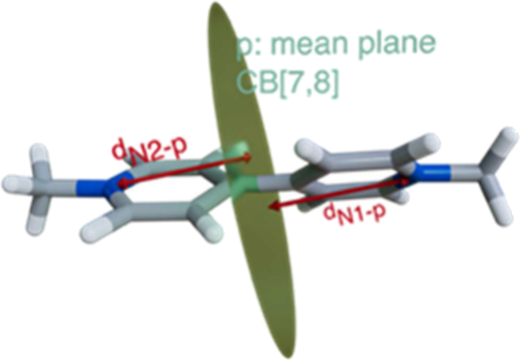

Next, to establish the responsiveness of the complex to a swap to more basic pH values, the effect of the complexation by CB[7] on the p*K*_a_′ of the guest was first evaluated (Figure 2c). Thus, an UV–vis experiments were carried out on a 20 μM solution of **R_a_**H^2+^ ⊂ CB[7], which was titrated with aliquots of appropriate solutions of NaH_2_PO_4_/Na_2_HPO_4_, KHCO_3_/K_2_CO_3_, and Na_2_HPO_4_/Na_3_PO_4_ buffers of increasing pH. As shown in Figure S150, the recorded spectra follow a similar qualitative trend to that described above for the guest itself, with the main original absorption for the acidic form of the compound (λ^1^_max_ = 378 nm), disappearing and being transformed into a new band at λ^2^_max_ = 467 nm, associated to the conjugated base. The obtained data indicated a slight decrease in the acidity of the NH group once complexed by CB[7] (Δp*K*_a_ = 0.7), pointing out the above-mentioned stabilizing hydrogen bonding between this moiety and the carbonyl in the presence of the host (vide infra). With this p*K*_a_ shift in mind, we proceeded to test the complexation of the conjugate base of the guest, **R_a_**^+^, by performing NMR experiments at pD = 12 to ensure the complete deprotonation of the compound. The recorded spectra showed that the host–guest system was also formed at this pD, with a rapid equilibrium situation being observed, and a subsequent DOSY experiment for the mixture showed signals of both host and guest diffusing as a whole (Figure S146). As previously discussed, despite some of the guest signals being quickly deuterated at the specified pD, we could conclude that the complexation-induced shifts observed for the complex agreed with an insertion mode for the guest more centered within the macrocycle (Figure S145), as it would be expected from the loss of the hydrogen bonding interaction. In this case, the lack of changes in the UV–vis spectra of the deprotonated thread upon complexation by CB[7], moves us to estimate the association constant through an NMR titration experiment, which rendered a value of *K*_i_′ = (7.4 ± 0.1)·10^2^ M^–1^ for **R_a_**^+^ ⊂ CB[7] (Figure 2d),^40,41^

In an effort to obtain further information on the potential structures and free energies of association for the binary complexes **R**_a_^+^/**R_a_**H^2+^ ⊂ CB[7], these were studied by means of dispersion-corrected density functional theory (DFT-D),^43−50^ and the results compared with those of the well-known complexes with paraquat, **MV**^2+^ ⊂ CB[7/8].^10,18,43^ Despite having similar computed and experimental free energies of association (Table 2), the minima found for **R_a_**H^2+^and **MV**^2+^ as guests showed a clear difference: while the paraquat complex exhibits a larger packing coefficient (Pc, Table 2),^51,52^ and better alignment of the nitrogen atoms on the guest with the carbonyl-laced portals (D, Table 2),^53^ the complex with **R_a_**H^2+^ shows on the optimized structure the proposed hydrogen bonding stabilizing interaction between the acidic NH group on the guest and one of the carbonyls on the host. As expected, deprotonation of the guest causes the disappearance of this interaction, which in conjunction with the loss of a positive charge, leads to a decrease in the computed free energy for **R**_a_^+^ ⊂ CB[7] despite a better host–guest alignment to establish cation–dipole interactions (Figure 2c).

In the case of the self-assembly of **R_a_**H·2Cl with CB[8] at pD = 7, similar features on the NMR experiments were observed than those discussed above for CB[7]. Again, the nuclei of the complexed guest were assigned with the aid of 1D/2D NMR, VT-NMR, and DOSY experiments, observing similar complexation-induced shifts for the species to those discussed for **R_a_**H^2+^ ⊂ CB[7], which imply a similar insertion mode with the possibility of a host–guest hydrogen bonding (Figures S152–156).^53^ In this case, for the complex formed between **R_a_**H^2+^ and CB[8], the equilibrium binding constant was estimated by using NMR competitive experiments, with paraquat **MV**^2+^ as a standard for the calculation.^10,18^ The host–guest interaction was observed to be stronger than that of the standard and the guest-exchange process was under no kinetic barriers. Consequently, the titration data allowed us to estimate *K*_i_ = (2.2 ± 0.2)·10^5^ M^–1^ for the binary complex **R_a_**H^2+^ ⊂ CB[8], with a good fitting of the data to a 1:1 model (Figures S158 and 159),^40,41^ and in good agreement with that reported by Kim and co-workers for **MV**^2+^ ⊂ CB[8].^18^ As for **R_a_**H^2+^, the p*K*_a_′ of the complexed guest was estimated by a UV–Vis titration (Figures S166 and S167), showing a moderate shift of +0.4 p*K*_a_ units caused by the CB[8] host, which again can be produced by an interaction of the NH moiety with the host. DFT-D results supported this fact, with similar results on the comparison of the minima obtained for the complex **R_a_**H^2+^ ⊂ CB[8] and its paraquat analogue, as those discussed for the CB[7] analogue (Table 2).

Moving to the study of the complexation of **R_a_**H·2Cl with CB[8] at basic conditions, we found how the ^1^H-NMR spectrum at pD = 12, for mixtures of host and guest at different stoichiometries, showed a quite complex situation. In all cases, a strong broadening is observed for almost all the signals corresponding to the deprotonated form of the guest (**R_a_**^+^). However, VT-NMR showed a shift to a fast exchange regime at 338.15 K (Figure S161), with the spectrum displaying the signals for the complexed “vermellogen” in good agreement with the formation of an inclusion complex with CB[8], which we hypothesized could be the homoternary 2:1 species (**R**_a_^+^)_2_ ⊂ CB[8].^54^ As for pD = 7, the association constant was determined by an NMR competitive experiment with **MV**^2+^ as the standard (Figures S164 and S165).^10,18^ The analysis of the data obtained for the titration fitted well on a 2:1 model, yielding a value of *K*_i_′ = (4.9 ± 0.3)·10^7^ M^–2^.^40,41^

**Figure 3:**
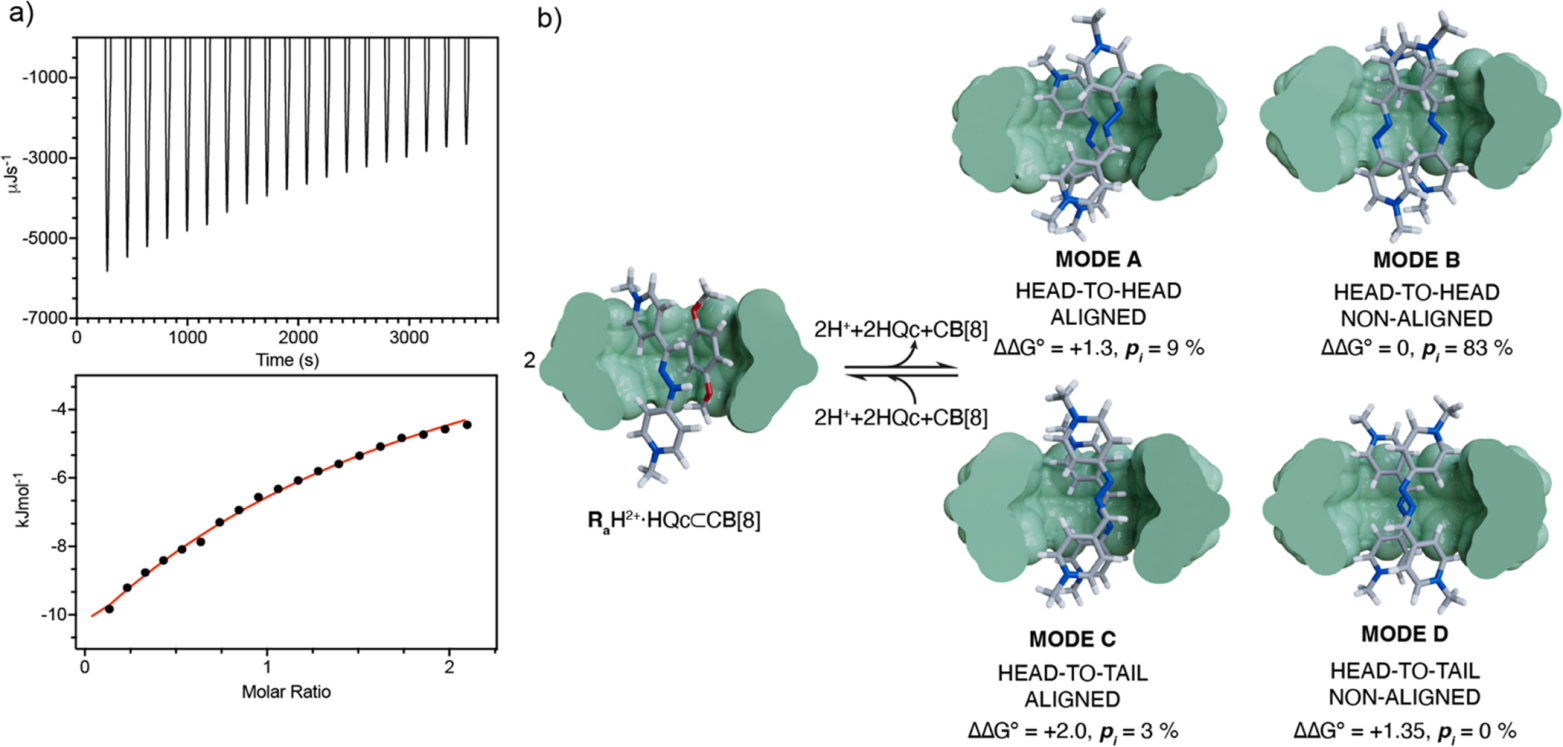
(a) ITC titration data and fitting for **R_a_**H^2+^ ⊂ CB[8] + HQc ⇌ **R_a_**H^2+^·HQc ⊂ CB[8]; (b) Schematic representation of the {2(**R_a_**H^2+^·HQc ⊂ CB[8])}⇄{(**R_a_**^+^)_2_ ⊂ CB[8] + CB[8] + 2HQc + 2H^+^} supramolecular switch, including molecular models of each of the different minima for the complexes involved. For clarity: one half of the CB[8] host depicted using a van der Waals representation in green, guest depicted using sticks; color code as in Figure 1.

Once more, the potential structures of the complexes formed and their stability were studied using DFT-D calculations, considering in this case the four potential relative poses of the two **R**_a_^+^ guests within the cavity of the receptor (modes **A**–**D**, Figure 3b). Four different minima were found matching each of those isomers on the potential energy surface of the inclusion complex, and their computed free energies were compared. Surprisingly, the head-to-head isomers **A** and **B** were found to be more stable at room temperature than the more intuitive head-to-tail counterparts, **C** and **D**. This would imply that, in order to establish two stabilizing cation–dipole interactions with CB[8], a non-symmetric distribution of the electron density on each of the complexed cations would be required, which is allowed by the highly delocalized nature of the π system in **R_a_**^+^. Furthermore, the computational results also support the formation of (**R_a_**^+^)_2_ ⊂ CB[8] over **R_a_**^+^ ⊂ CB[8], with the former being 14.4 kcal/mol more stable than the latter (Table 2).

To complete our study, we proceeded to evaluate the ability of **R_a_**H·2Cl to form heteroternary complexes with CB[8] and HQc as an appropriate second guest. First, we tested the complexation process by recording a ^1^H-NMR spectrum at pD = 7 of a solution of 1:1:1 (**R_a_**H·2Cl:HQc:CB[8]). Although some of the resonances for the two electronically complementary guests disappear because of a fast but near-coalescence exchange regime on the technique at r.t., no signals corresponding to the free but complexed HQc substrate were observed (Figure S168). To attain more information on the process, VT-NMR experiments were performed, showing two different phenomena on increasing the temperature: the sharpening of the signals of the two substrates moving out of the coalescence situation and the sequential decomplexation of the second guest (Figure S169). Nevertheless, the analysis of the evolution of the signals on the VT spectra on increasing the temperature, allowed us to qualitatively confirm the formation of the **R_a_**H^2+^·HQc ⊂ CB[8] and a tentative binding mode, in which the second guest situates itself within the cavity of the host, affecting more those resonances for the imine moiety of the first guest, which appear slightly shielded due to the guest–guest interaction.^54^ Again, this is in good agreement with the local minima for a simplified model of the structure of the complex found by DFT-D (Figure 3b), with this complex being 8.7 kcal/mol more stable in terms of free energy compared to **R_a_**H^2+^ ⊂ CB[8] (Table 2). Furthermore, the ability of HQc to act as a second guest was also corroborated by an ITC titration (Figure 3a), which allowed us to estimate a *K*_i_^″^ = (1.0 ± 0.2)·10^3^ M^–1^ and, hence, an overall association constant for the formation of **R_a_**H^2+^·HQc ⊂ CB[8] as *K* = *K*_i_ × *K*_i_^″^ = (2.2 ± 0.5)·10^8^ M^–2^, in good agreement with other thermodynamic values obtained for heteroternary complexes of CB[8] with viologens as the first guests.^10^

As it would be expected from the *K*_i_ values obtained for the different CB[8]-based complexes discussed herein, a swap to more basic conditions of the **R_a_**H^2+^·HQc ⊂ CB[8] complex, and the subsequent deprotonation of **R_a_**H^2+^, was expected to produce the pH-based supramolecular switch {2(**R_a_**H^2+^·HQc ⊂ CB[8])} ⇄ {(**R_a_**^+^)_2_ ⊂ CB[8] + CB[8] + 2HQc + 2H^+^}, similar to the classical Kim’s supramolecular switch produced upon reduction/oxidation of viologen·electron donor heteroternary complexes with CB[8].^18^ To corroborate this end, a 1:1:1 **R_a_**H·2Cl:HQc:CB[8] equimolar solution was prepared at pD = 12 and the corresponding ^1^H-NMR experiment was recorded, showing the expected formation of (**R_a_**^+^)_2_ ⊂ CB[8] and signals corresponding to the free HQc guest (Figure S172). DFT-D calculations also support this end, with an estimated value of ΔΔ*G*° = −5.9 kcal/mol in favor of the local minima found for the homoternary complex when compared to that of the potential heteroternary aggregate **R_a_**^+^·HQc ⊂ CB[8], which can be rationalized both on the basis of the lesser qualities of **R_a_**^+^ as an electron acceptor and/or the entropic penalties associated with the formation of the heteroternary complex.

To summarize, we have reported herein the development of a new class of organic salts with molecular switching capabilities, the “vermellogens”, which can be efficiently synthesized in acidic water by hydrazone exchange reactions and show a marked acid–base responsivity on biologically relevant pH-values. Although “vermellogens” can be considered as structural analogues of viologens, we anticipated that the introduction of the hydrazone moiety would disrupt the ability of the conjugated pyridinium rings to be reversibly reduced, which in turn would decrease the cytotoxicity of our compounds compared to viologens. This end was demonstrated herein by comparison of the viabilities of HFF-1 cells when exposed to the model compounds “red thread” and the well-known toxic herbicide paraquat, resulting in striking differences in the cell survival rates for the two salts. Furthermore, the study of the host–guest chemistry of the model “vermellogen”, with the popular CB[7/8] hosts, showed marked parallelism with that of paraquat, substituting the redox-responsiveness of the latter by an acid–base conditioned binding for the “red thread”-based complexes. In our opinion, the results reported herein open new avenues for the development of functional stimuli-responsive supramolecular systems, in particular those for which the well-known toxicity of viologens is a clear handicap.

## Supporting Information Available

Experimental details, synthetic procedures and characterization data for new compounds, titration data for the determination of p*K*_a_ values and supramolecular association constants (*K*_i_s), details on the cell-viability assays for **R_a_**H·2Cl and **MV**·2I, crystallographic data for **R_a_**H·2Cl (CCDC: 2196219), computational details and Cartesian coordinates for the different energy minima discussed in the manuscript and additional figures (PDF)
