# Supplementary material for: “Vermellogens” and the Development of CB[8]-Based Supramolecular Switches Using pH-Responsive and Non-Toxic Viologen Analogues
Source: J Am Chem Soc. 2022 Oct 7;144(41):19127–36. doi: 10.1021/jacs.2c08575 (PMC9682480; doi:10.1021/jacs.2c08575)
Supplement: Supplementary file 1 — ja2c08575_si_001.pdf [file ja2c08575_si_001.pdf]

Supporting information

"Vermellogens" and the development of CB[8]-based Supramolecular Switches Using pH-responsive and Non-Toxic Viologen Analogues.

Liliana Barravecchia,[a] Arturo Blanco-Gómez,[a] Iago Neira,[a] Raminta Skackauskaite,[a] Alejandro Vila,[a] Ana Rey-Rico,[b] Carlos Peinador,[a,*] and Marcos D. García[a,*]

[a]Departamento de Química and Centro de Investigaciones Científicas Avanzadas (CICA). Facultad de Ciencias, Universidade da Coruña, 15071, A Coruña, Spain.

[b]Gene & Cell therapy research group (G-CEL). Centro de Investigacións Científicas Avanzadas (CICA), Universidade da Coruña, 15071, A Coruña, Spain.

Table of contents

1. General procedures. 4
2. Synthetic procedures. 5
  - 2.1. Synthesis and characterization data of 4_b·Br 5
  - 2.2. Synthesis and characterization data of 3_b·Br 6
  - 2.3. Characterization of R_a·Cl 8
  - 2.4. Synthesis and characterization data of R_bH·2Cl 10
  - 2.5. Synthesis and characterization data of R_cH·2Cl 20
  - 2.6. Synthesis and characterization data of R_dH·2Cl 30
  - 2.7. Synthesis and characterization data of R_eH·2Cl 40
  - 2.8. Synthesis and characterization data of M_aH 50
  - 2.9. Synthesis and characterization data of M_bH·Cl 60
3. Anion interaction study with R_aH^{2+} 70
  - 3.1. 2F□R_aH^{2+} titration by UV-vis in ACN. 70
  - 3.2. 2F□R_aH^{2+} study by ^{1}H-NMR. 71
  - 3.3. Cl□R_aH^{2+} titration by UV-vis in ACN. 72
  - 3.4. Cl□R_aH^{2+} study by ^{1}H-NMR. 73
  - 3.5. Br□R_aH^{2+} titration by UV-vis in ACN. 74
  - 3.6. Br□R_aH^{2+} study by ^{1}H-NMR. 75
4. Acid/base spectroscopy study of synthesized compounds 76
  - 4.1. Spectroscopy study of R_b·2Cl in H_2O 76
  - 4.2. pK_a determination for R_b by UV-Vis 77
  - 4.3. Spectroscopy study of R_c in H_2O 78
  - 4.4. pK_a determination for R_c by UV-Vis 79
  - 4.5. Spectroscopy study of R_d in H_2O 80
  - 4.6. pK_a determination for R_d by UV-Vis 81
  - 4.7. Spectroscopy study of R_e in H_2O 82
  - 4.8. pK_a determination for R_e by UV-Vis 83
  - 4.9. Spectroscopy study of M_a in H_2O 84
  - 4.10. pK_a determination for M_a by UV-Vis 85
  - 4.11. pK_a determination for M_b by UV-Vis 86
5. Determination of rotational energy barrier ($\Delta G^{\neq}$) 87
6. Cyclic voltammetry studies of R_aH·2Cl in water at different pH. 93
7. Cell viability assays 94

8. Host-guest chemistry 95
8.1. Self-assembly of $R_aH \cdot 2Cl$ with cucurbit[7]uril: 95
8.2. pK_a determination for $R_a \subset CB[7]$ by UV-Vis 103
8.3. Self-assembly of $R_aH \cdot 2Cl$ with cucurbit[8]uril: 104
8.4. pK_a determination for $R_aH^{2+} \subset CB[8]$ by UV-Vis 114
8.5. Synthesis and characterization of the heteroternary complex:..115
9. X-ray crystallographic data. 119
10. Computational details: 122

1. General procedures.

Starting materials were purchased from commercial suppliers and used without further purification. Compounds 4-hydrazineyl-1-methylpyridinium iodide,[1] 4-formyl-1-methylpyridinium iodide[2] and **R**$_{\mathbf{a}}$H·2Cl[1] were prepared according to published procedures. Milli-Q water was purified with a Millipore Gradient A10 apparatus. Merck 60 F254 foils were used for thin layer chromatography, and Merck 60 (230-400 mesh) silica gel was used for flash chromatography. NMR spectra were recorded on a Bruker Advance 300, 400 or 500 MHz for ^{1}H, and 75, 101 or 126 MHz for ^{13}C, equipped with a dual cryoprobe. The solvents used for NMR experiments were D_2O or CD_3CN. Mass spectrometry experiments were carried out in a LCQ-q-TOF Applied Biosystems QSTAR Elite spectrometer for low and high resolution ESI. UV/Vis spectra were recorded on a Jasco V-650 spectrometer. Potentiometric measure was carried out with CRISON 5028 pH electrode for microsamples with Ag/AgCl reference element. Titration experiments were carried out in 10 mM sodium phosphate buffer at 25 °C on a Nano-ITC calorimeter from TA instruments. HPLC-MS analysis were performed using a Thermo Scientific UltiMate 3000 connected to a photo-diode array (PDA) detector and a single quadrupole mass spectrometer Thermo Scientific MSQ Plus or a UHPLC Thermo Scientific Accela connected to a PDA detector and a linear trap quadrupole mass spectrometer Thermo Scientific Orbitrap Discovery, using an Aeris analytical column from Phenomenex (peptide XB-C18 stationary phase, 3.6 μm, 100 Å pore size, 150 × 2.1 mm).

[1] Blanco-Gómez, A.; Neira, I.; Barriada J. L.; Melle-Franco, M.; Peinador, C.; García, M. D. Thinking outside the "Blue Box": from molecular to supramolecular pH-responsiveness. *Chem. Sci.* **2019**,*10*, 10680-10686.

[2] Blanco-Gómez A.; Fernández-Blanco, Á.; Blanco, V.; Rodríguez, J.; Peinador, C.; García, M. D. Thinking Outside the "Blue Box": Induced Fit within a Unique Self-Assembled Polycationic Cyclophane. *J. Am. Chem. Soc*. **2019**, *141*, 3959-3964.

2. Synthetic procedures.

2.1. Synthesis and characterization data of 4_b·Br

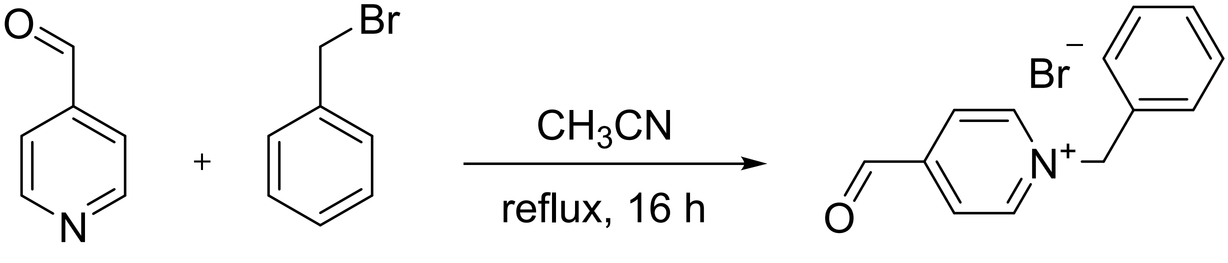

4_b·Br

Isonicotinaldehyde (0.22 g, 0.198 mL, 1.8 mmol, 1 eq.) and (bromomethyl)benzene (0.92 g, 0.65 mL, 5.4 mmol, 9 eq.) were dissolved in acetonitrile (20 mL) and heated at reflux for 16 hours. The crude solution was concentrated under vacuum and the resulting residue was redissolved in H_2O (20 mL). The aqueous phase was washed with hexane (10 mL x 3), concentrated and dried under vacuum to yield **4_b**·Br (0.425 g, 80%) as a reddish sticky oil**. ^{1}H NMR** (300 MHz, D_2O) δ 8.93 (d, 2H), 8.15 (d, 2H), 7.60 – 7.41 (m, 5H), 6.21 (s, 1H), 5.83 (s, 2H). **HRMS (ESI)** (*m/z*): calcd for $[M+OCH_4]^+$ 230.1176, found 230.117.

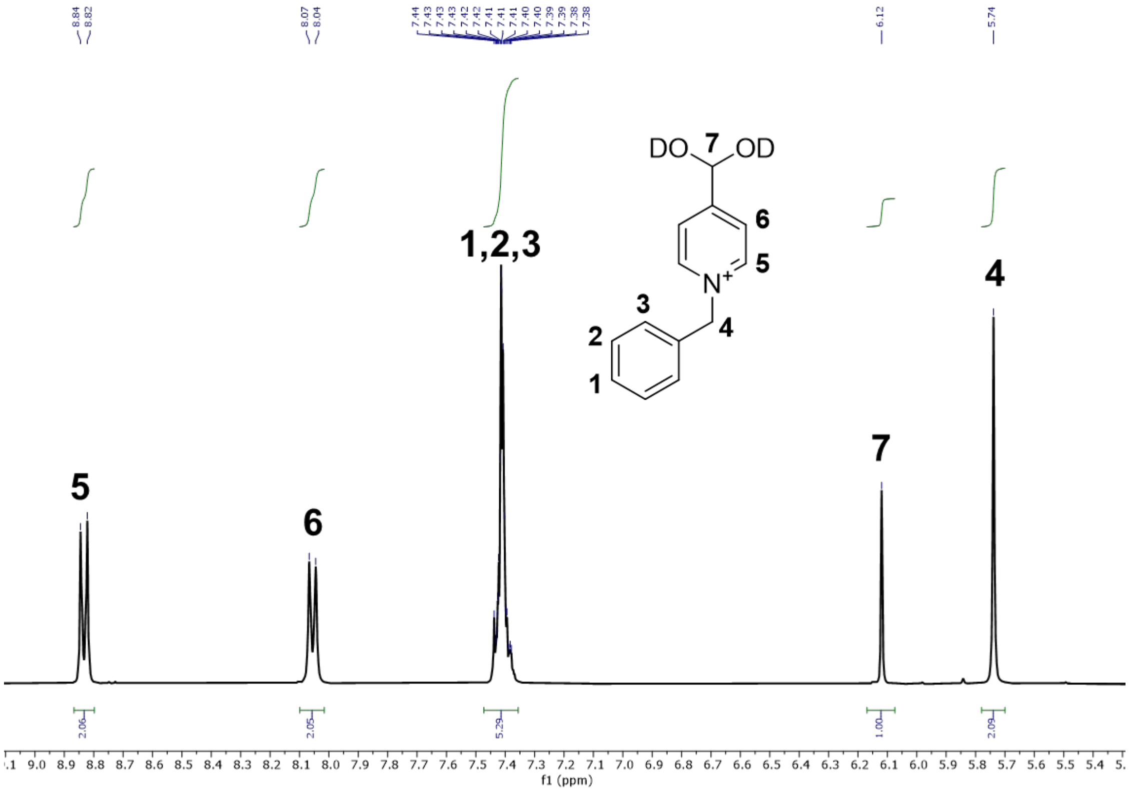

Figure S 1. ^{1}H NMR (300 MHz, D_2O) spectrum of **4_b**·Br.

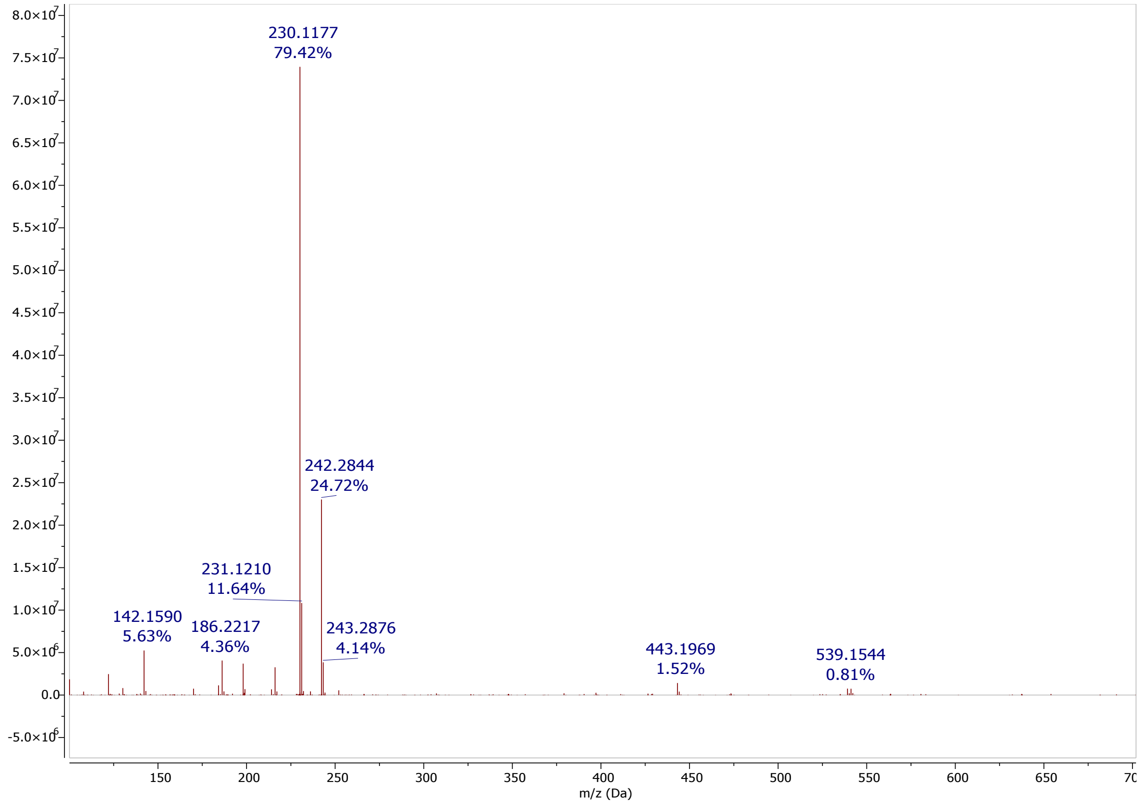

Figure S 2. HR ESI-MS spectrum of $\mathbf{4_b}$·Br.

2.2. Synthesis and characterization data of $\mathbf{3_b}$·Br

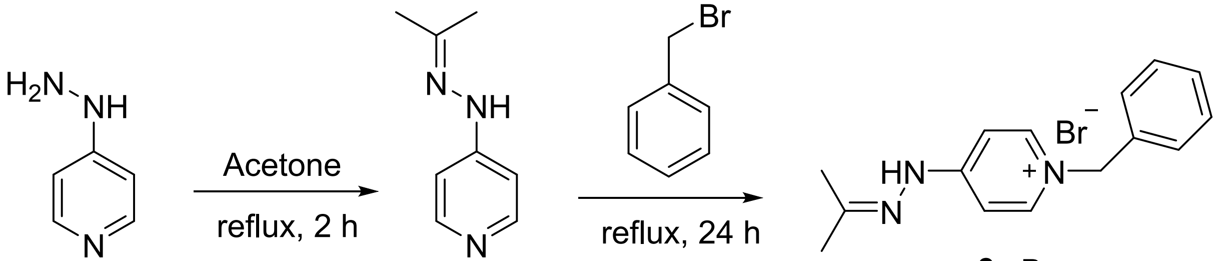

A solution of 4-hydrazineylpyridine (2.15 g., 19.7 mmol, 1 eq.) in 20 mL acetone was heated at reflux for 2 hours. (Bromomethyl)benzene (7 mL, 59.1 mmol, 3 eq.) was added to the resulting solution and heated at reflux for 24 hours. The crude solution was concentrated under vacuum and the resulting residue was redissolved in H_2O (30 mL). The aqueous phase was washed with hexane (10 mL x 3), concentrated and dried under vacuum to yield $\mathbf{3_b}$·Br (5.7 g, 90%) as an orange oil. **^{1}H NMR** (300 MHz, D_2O) δ 8.17 (m, 2H), 7.52 – 7.38 (m, 6H), 7.05 (d, 1H), 5.36 (s, 2H) **HRMS (ESI)** (*m/z*): calcd for $[M]^+$ 240.1496, found 240.1497.

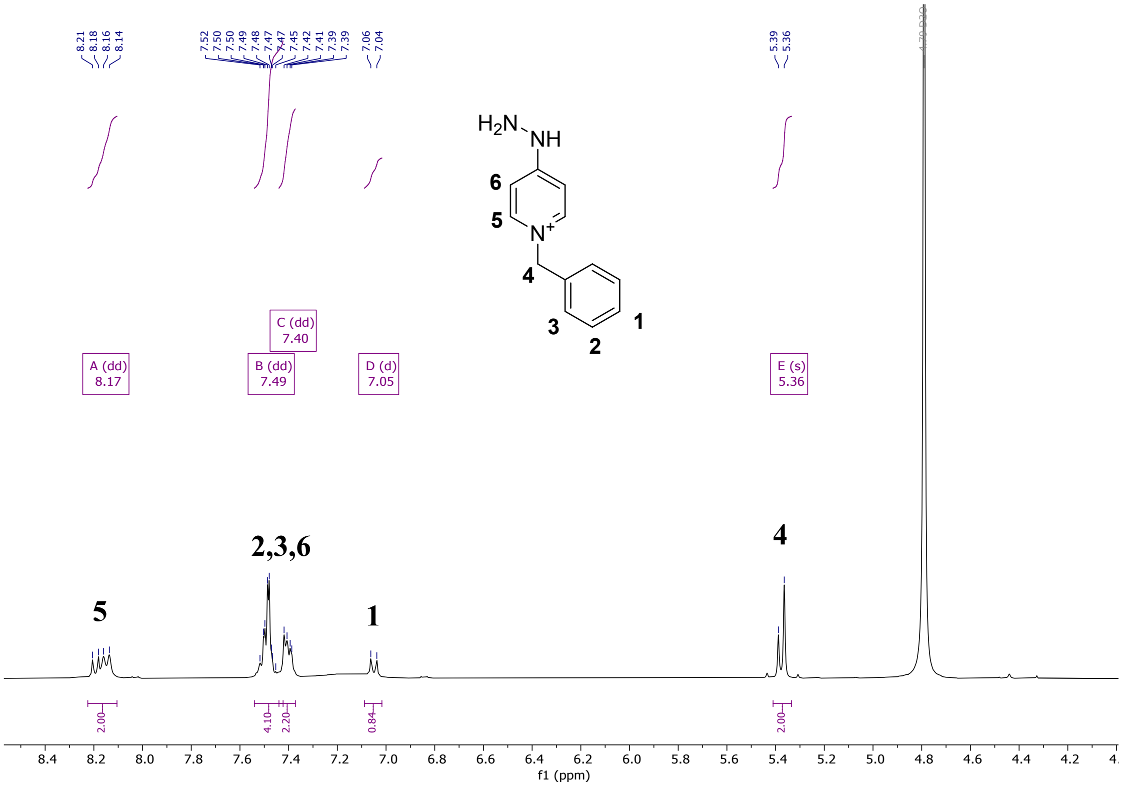

Figure S 3: ^{1}H NMR (300 MHz, D_2O) spectrum of $\mathbf{3_b}$·Br.

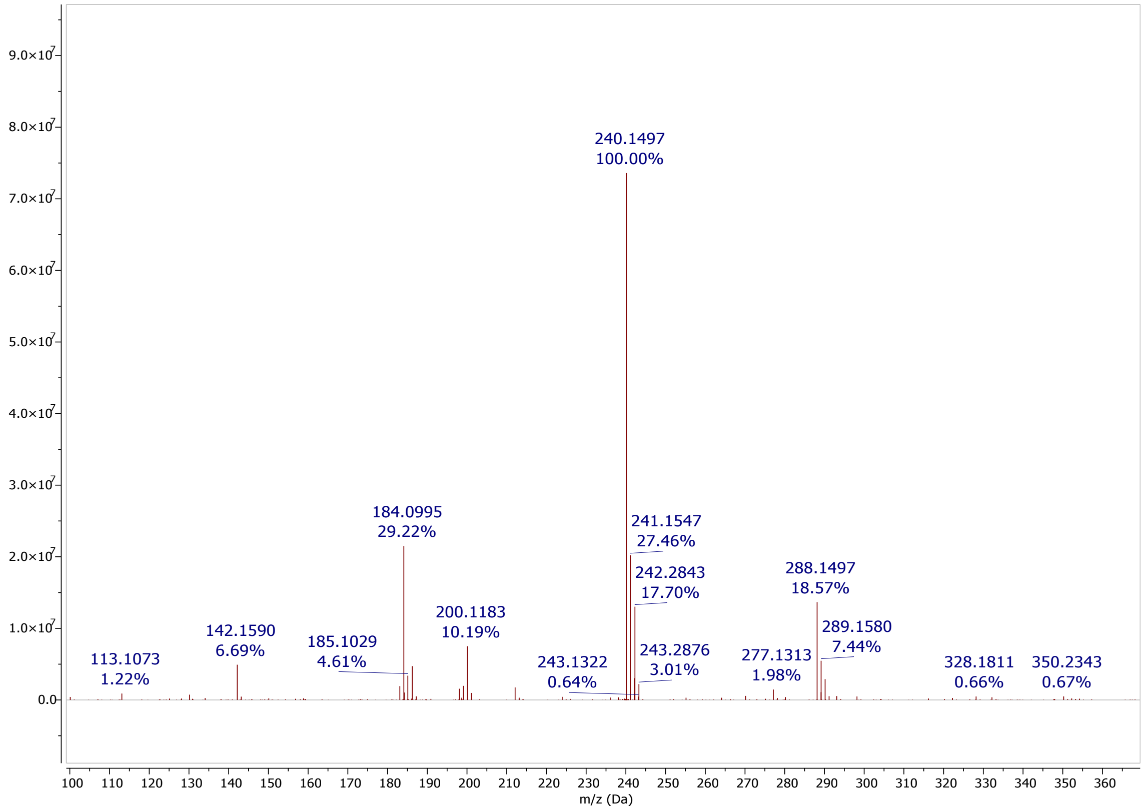

Figure S 4. HR ESI-MS spectrum of $\mathbf{3_b}$·Br.

2.3. Characterization of R_a·Cl

- **R_a·Cl at pD = 12.**

^{1}H NMR (300 MHz, D_2O) δ 8.42 (d, *J* = 6.8 Hz, 2H), 8.10 (s, 1H), 8.04 (d, *J* = 6.9 Hz, 2H), 7.63 (d, *J* = 7.7 Hz, 2H), 7.30 (s, 1H), 6.73 (s, 1H), 4.16 (s, 3H), 3.72 (s, 3H).

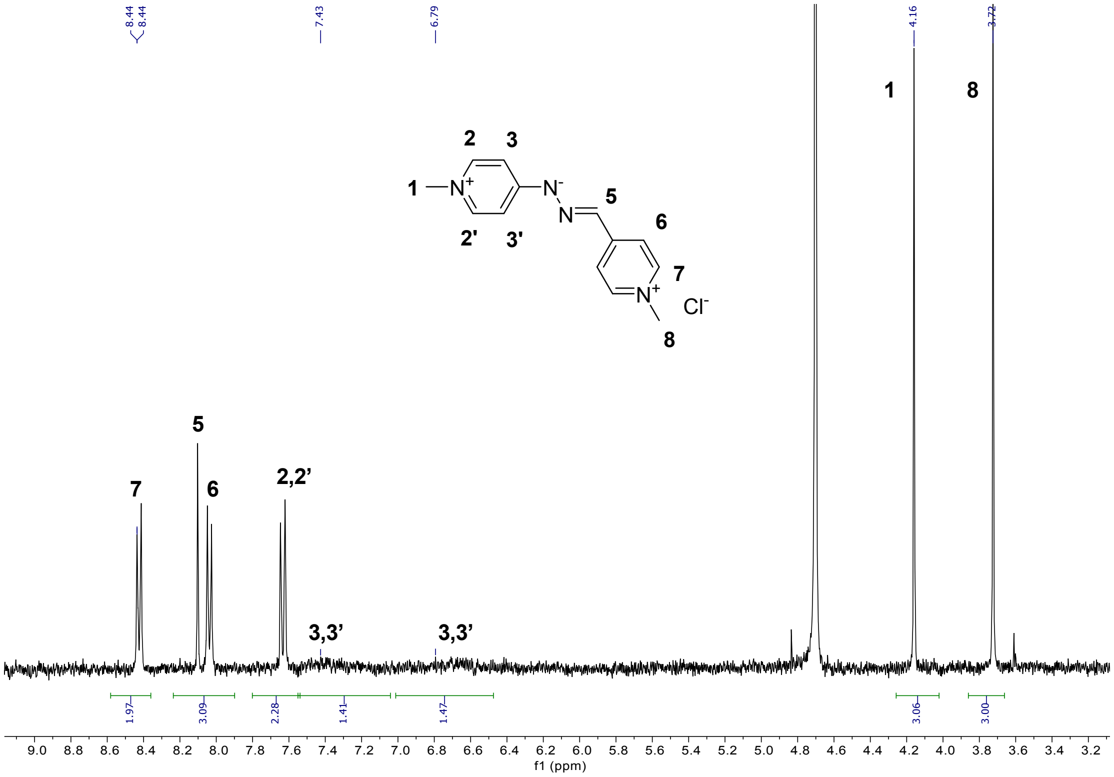

Figure S 5: ^{1}H NMR (500 MHz, D_2O) spectrum of **R_a**·Cl at pD 12.

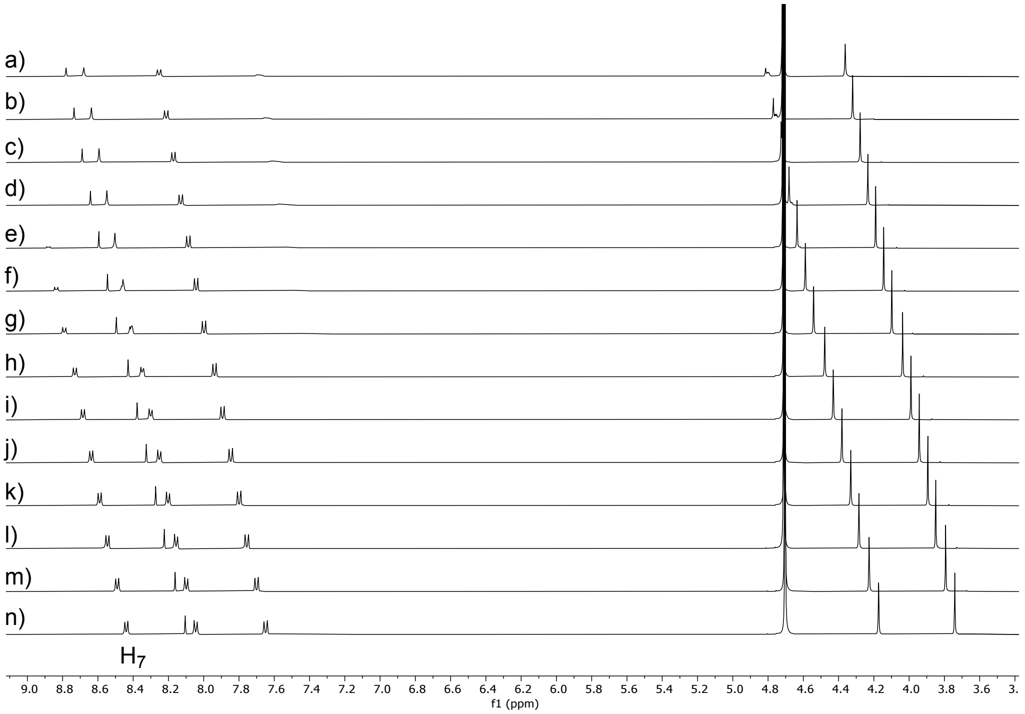

Figure S 6: VT ^{1}H NMR (400 MHz, D_2O) stacked spectra of $\mathbf{R_a}$·Cl (1 mM) at pD 12. Deuterated signal (H_7) marked in. a) 363.15 K b) 358.15 K c) 353.15 K d) 348.15 K e) 343.15 K f) 338.15 K g) 333.15 K h) 328.15 K i) 323.15 K j) 318.15 K k) 313.15 K l) 308.15 K m) 303.15 K n) 298.15 K.

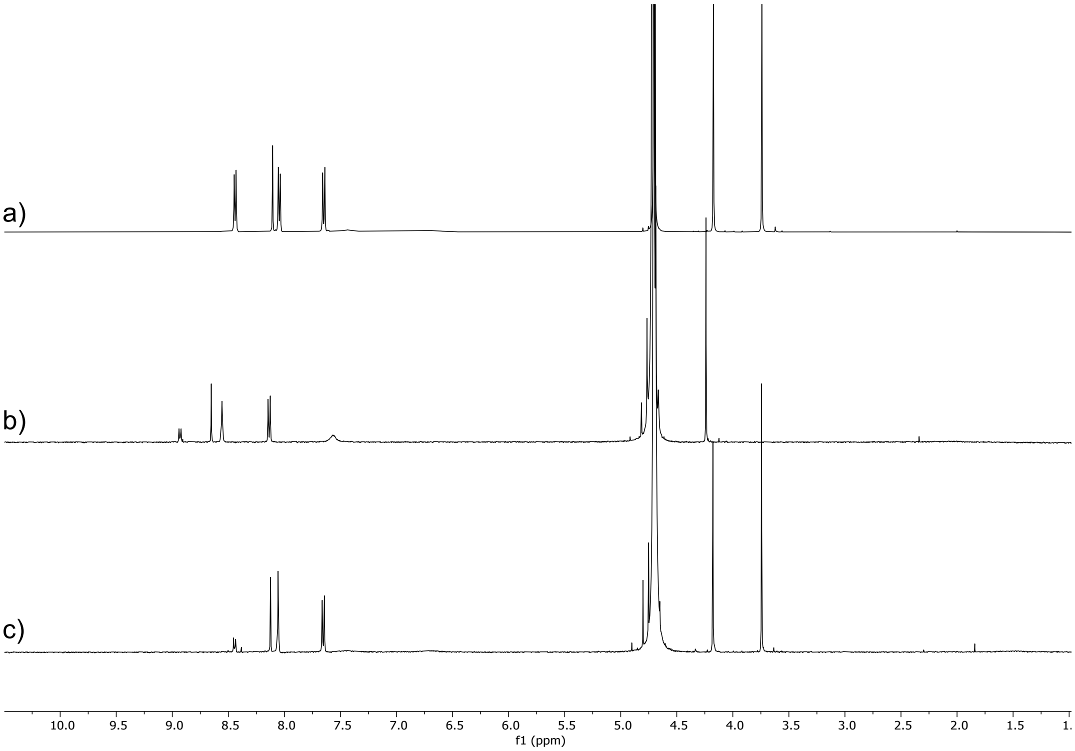

Figure S 7: ^{1}H NMR (400 MHz, D_2O) stacked spectra of a) $\mathbf{R_a}$·Cl (1 mM, pD 12, 298 K), b) $\mathbf{R_a}$·Cl (1 mM, pD 12, 348 K), c) $\mathbf{R_a}$·Cl (1 mM, pD 12, 298 K) after heating.

2.4. Synthesis and characterization data of $R_bH{\cdot}2Cl$

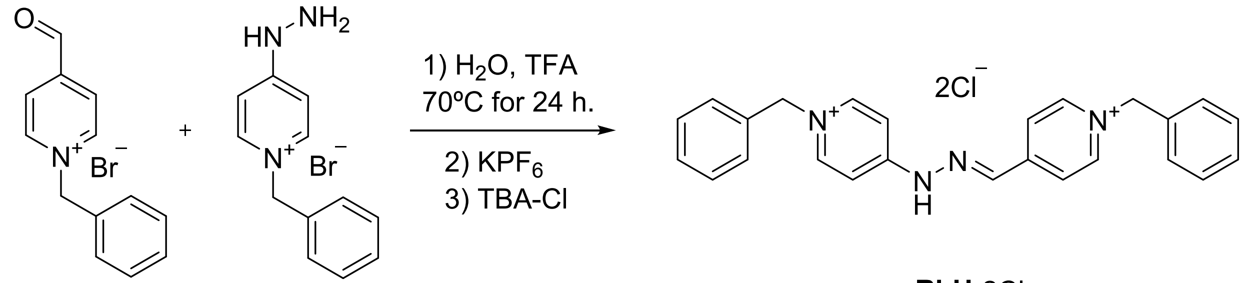

RbH·2Cl

4b·Br **3b**·Br

A solution of $\mathbf{4_bH}$·Br (0.826 g, 2.579 mmol, 1.3 eq.), $\mathbf{3_bH}$·Br (0.552 g, 1.985 mmol, 1 eq.) and TFA (10 % molar) in 100 mL of H_2O was heated at 60 ºC for 24 hours. After cooling, excess of KPF_6 was added until no further precipitation was observed. The obtained solid was filtrated, washed with water (3 x 10 mL) and Et_2O (3 x 10 mL) and dried under vacuum to yield $\mathbf{R_bH}{\cdot}2PF_6$ as a burnt orange powder (958 mg, 70 %). $\mathbf{R_bH}{\cdot}2PF_6$ was dissolved in a minimum quantity of CH_3CN (5 mL) and precipitated with TBACl. The resulting precipitated was washed with CH_3CN (3 x 10 mL), Et_2O (3 x 10 mL) and dried under vacuum to yield $\mathbf{R_bH}{\cdot}2Cl$ as an orange solid (446 mg, 51 %).

^{1}H NMR (500 MHz, D_2O) δ 8.85 (d, J = 6.8 Hz, 2H), 8.40 (s, 2H), 8.32 – 8.28 (m, 2H), 8.26 (s, 1H), 7.86 (s, 1H), 7.53 – 7.43 (m, 8H), 7.43 – 7.35 (m, 2H), 7.21 (s, 1H), 5.77 (s, 2H), 5.49 (s, 2H) ppm. **^{13}C NMR** (126 MHz, D_2O) δ 154.51 (C), 149.81 (C), 144.39 (CH), 143.81 (CH), 141.13 (C=N), 133.80 (C), 132.69 (C), 129.87 (CH), 129.52 (CH), 129.40 (CH), 129.37 (CH), 128.99 (CH), 128.45 (CH), 124.77 (CH), 110.53 (CH), 109.25 (CH), 64.08 (CH_2), 61.92 (CH_2) ppm. **HRMS (ESI)** (*m/z*): calcd for $[C_{25}H_{24}N_4\text{-}H]^+$ 379.1918, found 379.1917.

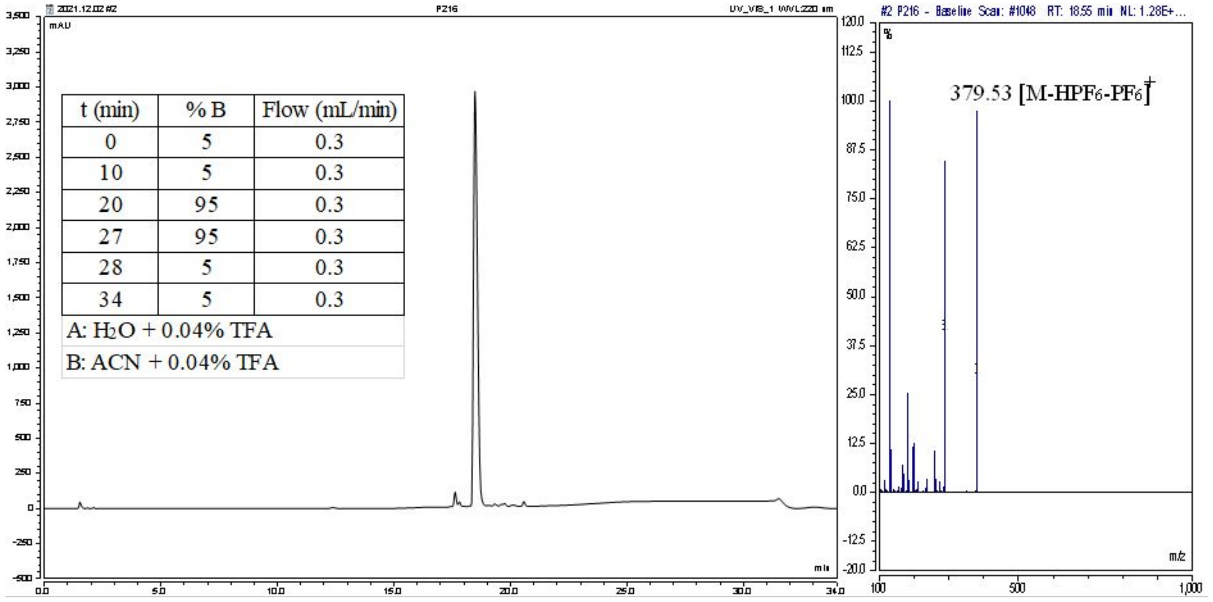

Figure S 8. HPLC-MS chromatogram of $\mathbf{R_bH}{\cdot}2PF_6$. *Inset.* Elution conditions.

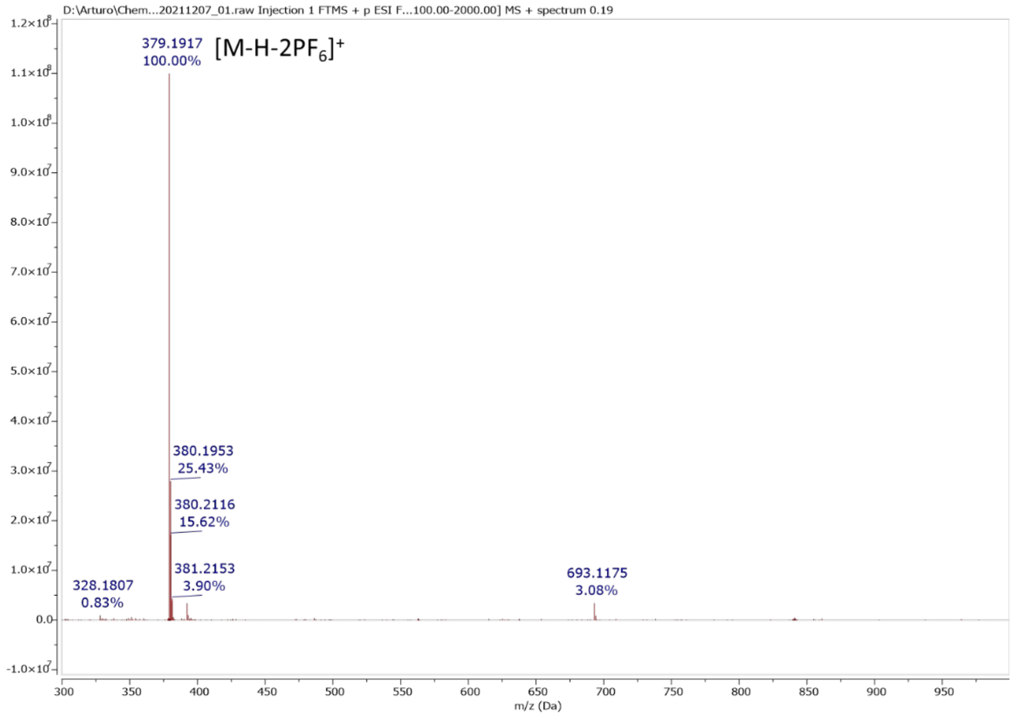

Figure S 9. HR ESI-MS spectrum of $\mathbf{R_b}$H·2PF_6.

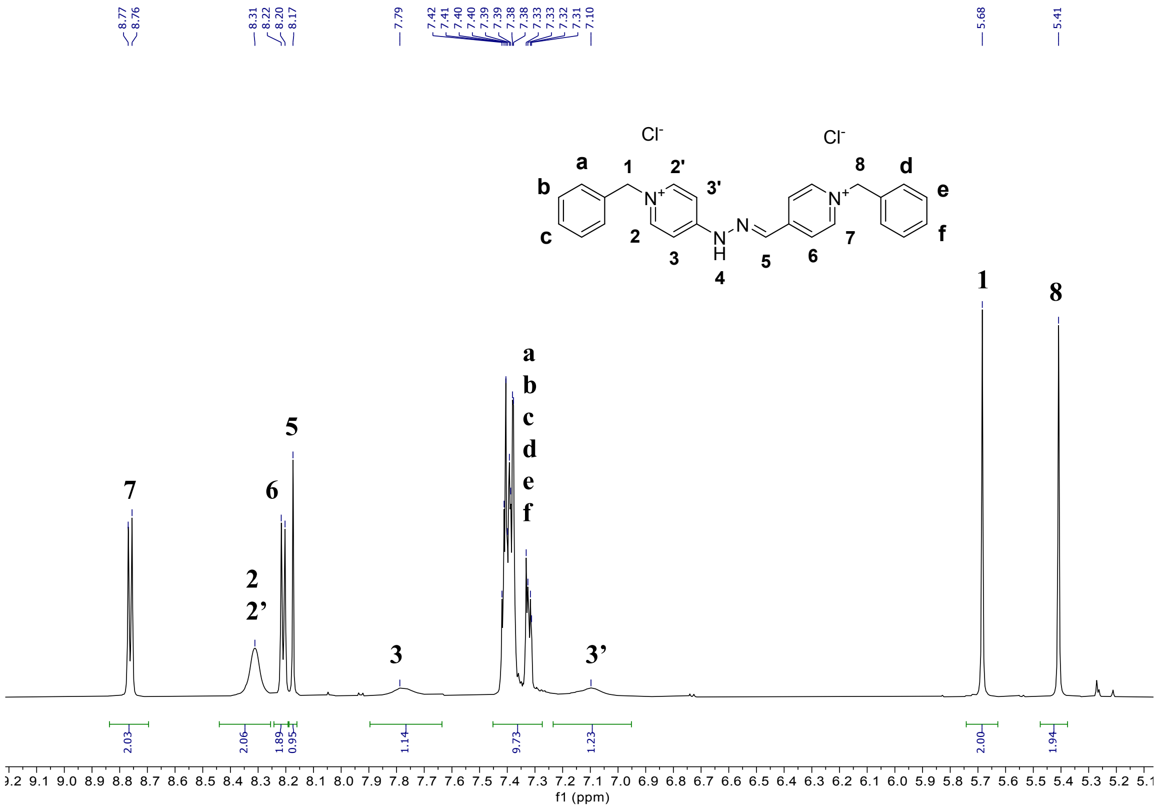

Figure S 10: ^{1}H NMR (500 MHz, D_2O) spectrum of $\mathbf{R_bH}$·2Cl.

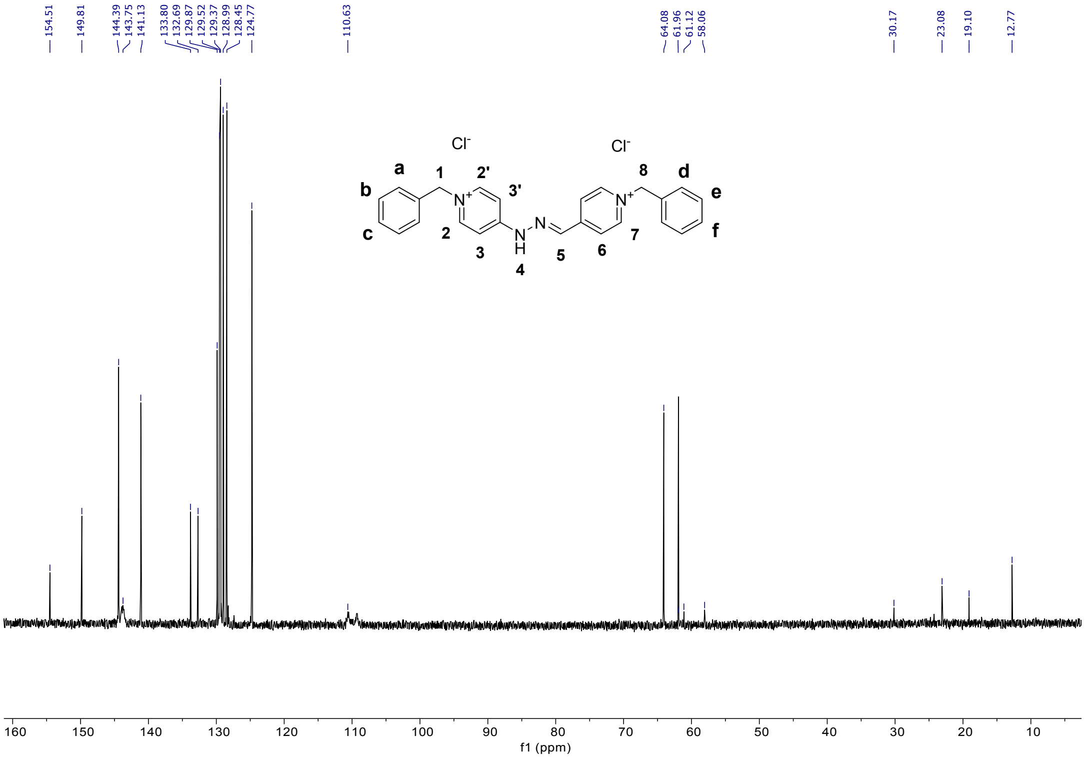

Figure S 11: ^{13}C NMR (126 MHz, D_2O) spectrum of $\mathbf{R_bH}$·2Cl.

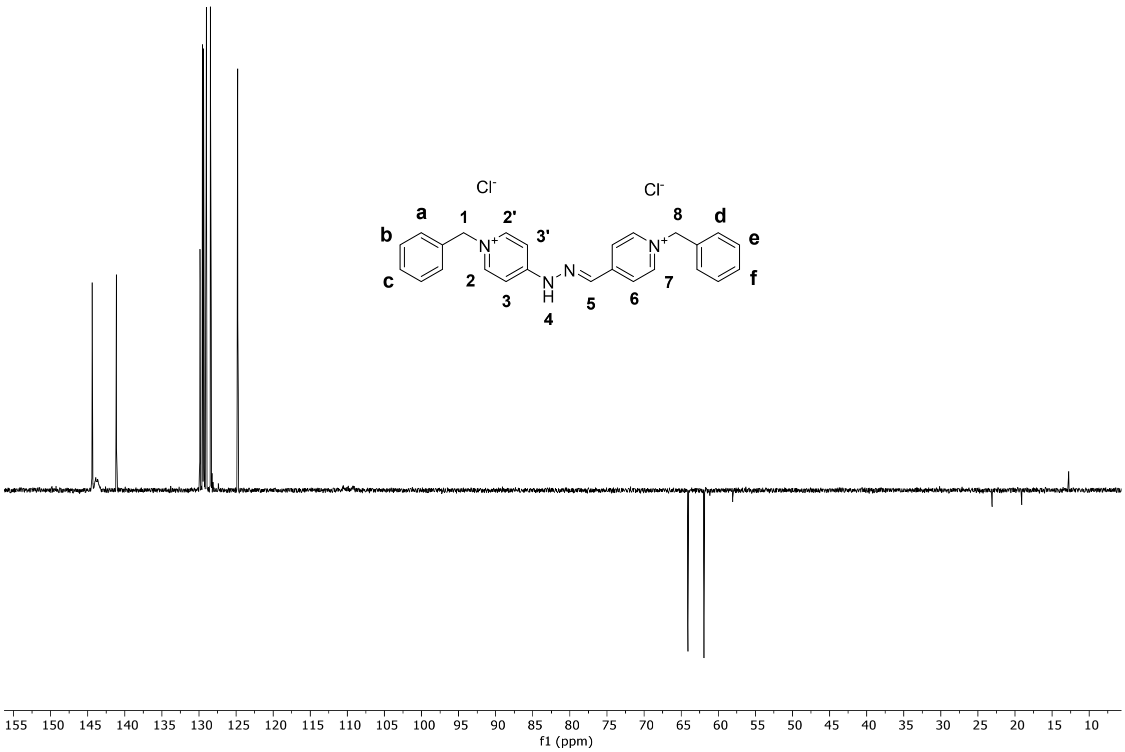

Figure S 12: DEPT-135 (126 MHz, D_2O) spectrum of $\mathbf{R_bH}$·2Cl.

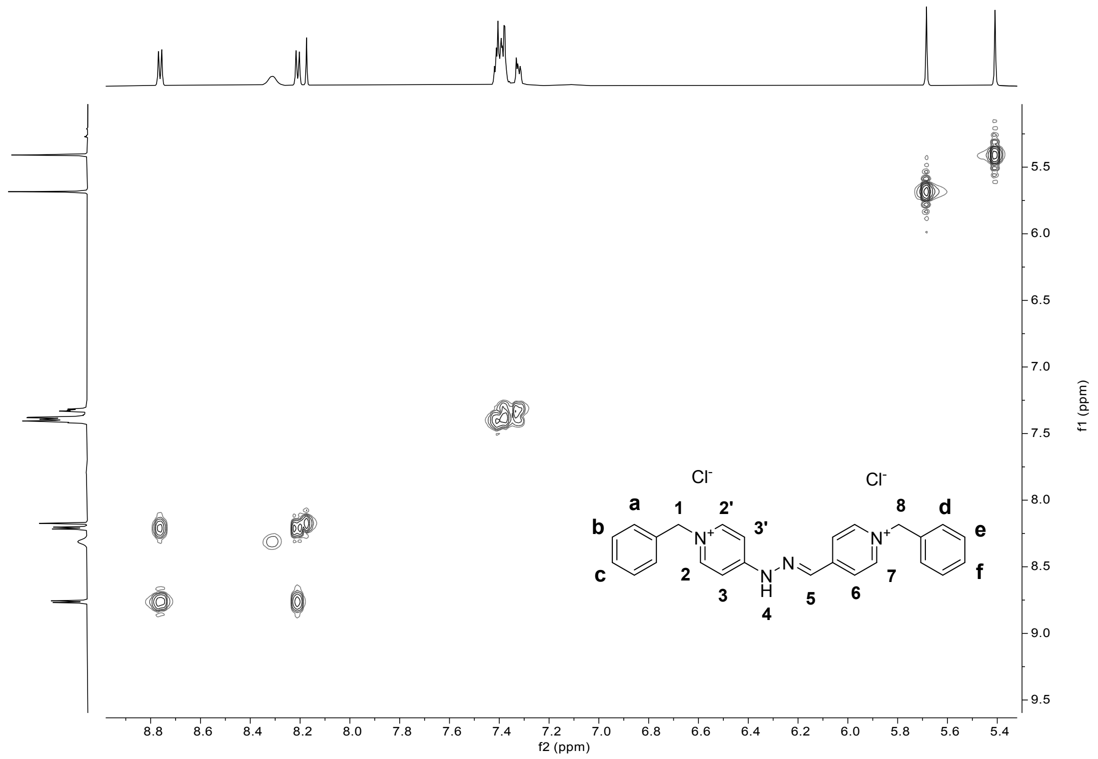

Figure S 13: COSY (500 MHz, D_2O) spectrum of $\mathbf{R_bH}$·2Cl.

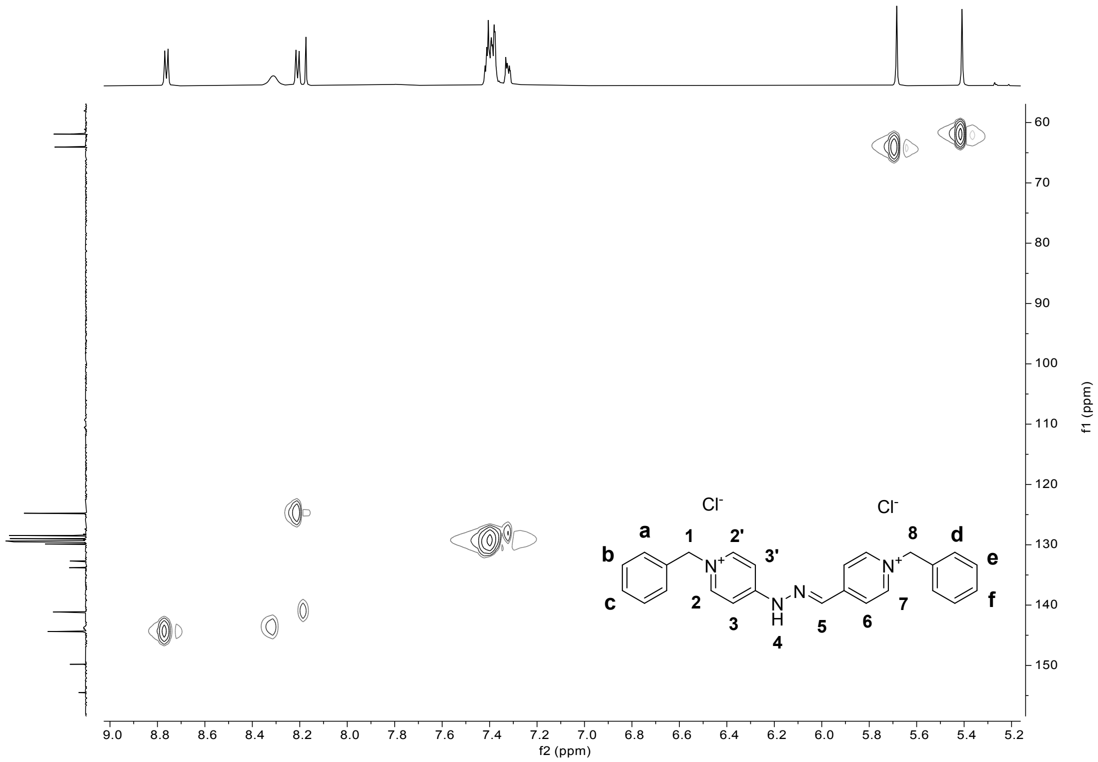

Figure S 14: HSQC (500 and 126 MHz, D_2O) spectrum of $\mathbf{R_bH}$·2Cl.

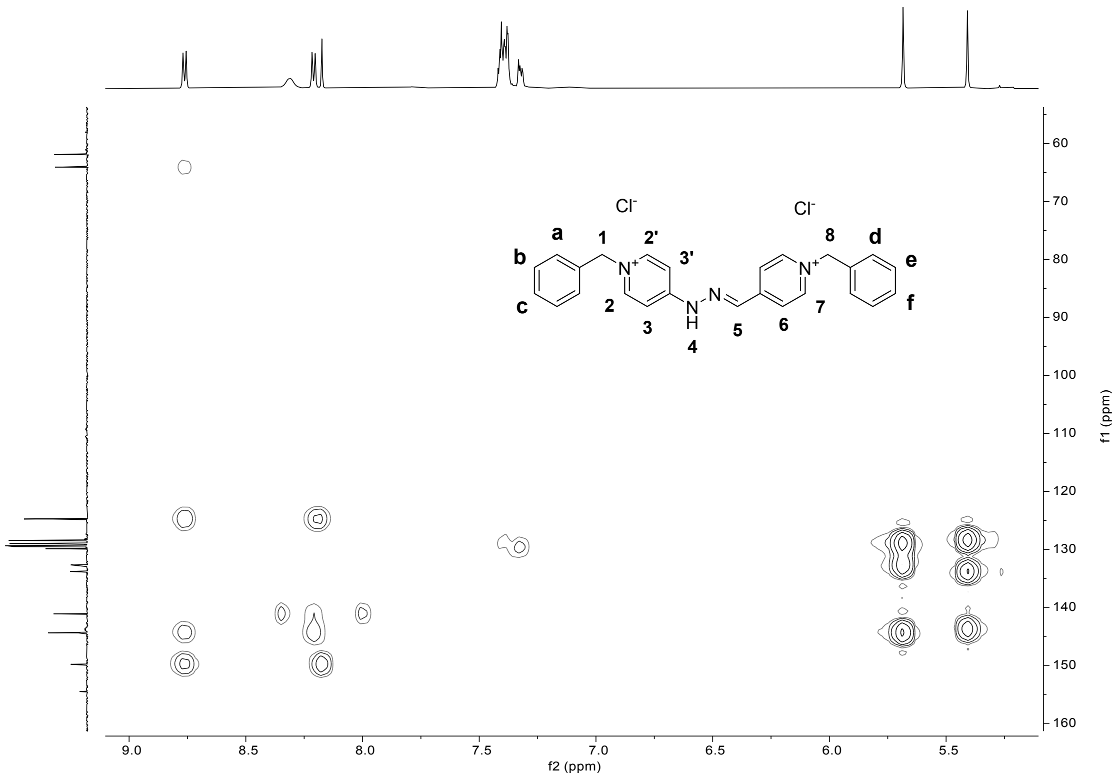

Figure S 15: HMBC (500 and 126 MHz, D_2O) spectrum of $\mathbf{R_bH}$·2Cl.

- **R_b·Cl at pD = 12.**

^{1}H NMR (300 MHz, D_2O) δ 8.52 (d, *J* = 6.7 Hz, 2H), 8.11 (s, 1H), 8.04 (d, *J* = 7.0 Hz, 2H), 7.72 (s, 2H), 7.55 – 7.32 (m, 11H), 6.61 (s, 1H), 5.59 (s, 2H), 5.15 (s, 2H) ppm.

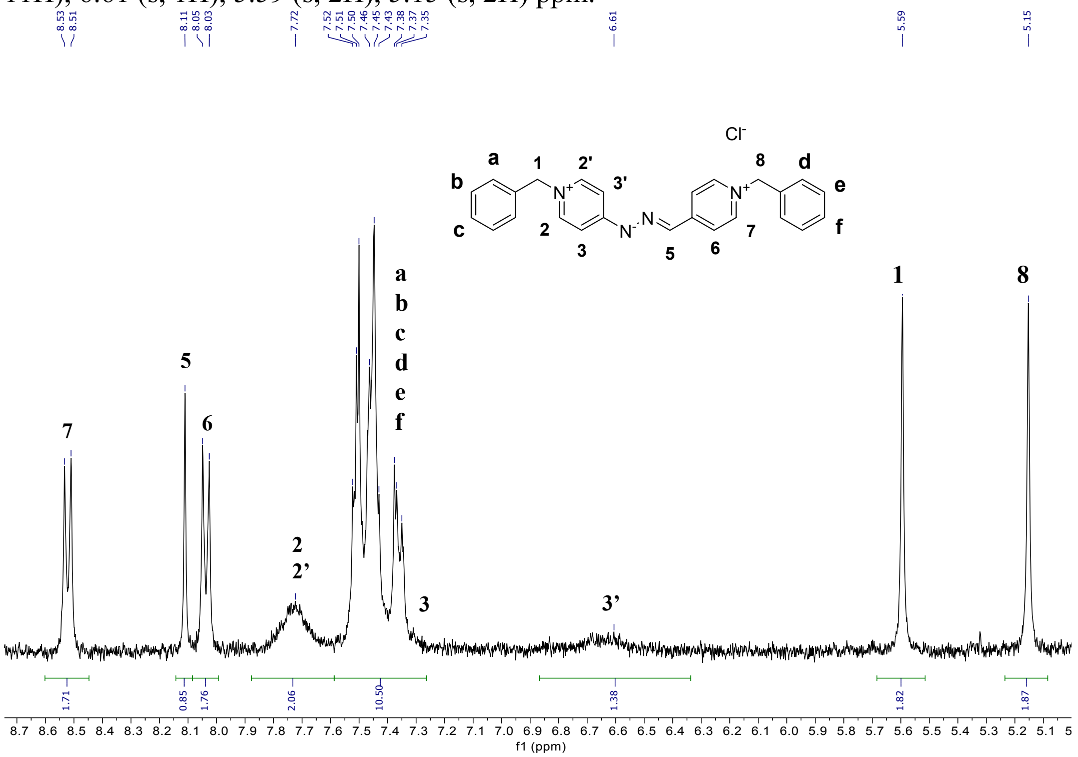

Figure S 16: ^{1}H NMR (300 MHz, D_2O) spectrum of $\mathbf{R_b}$·Cl.

- **$R_bH\cdot 2PF_6$:**

^{1}H NMR (500 MHz, CD_3CN) δ 12.53 (s, 1H), 8.70 (d, 2H), 8.49 (s, 1H), 8.39 – 8.20 (m, 4H), 7.80 (s, 1H), 7.59 – 7.20 (m, 11H), 5.68 (s, 2H), 5.43 (s, 2H) ppm. **^{13}C NMR** (126 MHz, CD_3CN) δ 154.41 (C), 149.63 (C), 144.31 (CH), 143.49 (CH), 140.83, 133.74 (C), 132.67 (C), 130.62 (CH), 129.57 (CH), 129.20 (CH), 129.09 (CH), 129.05 (CH), 128.79 (CH), 128.19 (CH), 124.74 (CH), 110.63 (CH), 109.03 (CH), 63.68 (CH_2), 61.57 (CH_3) ppm.

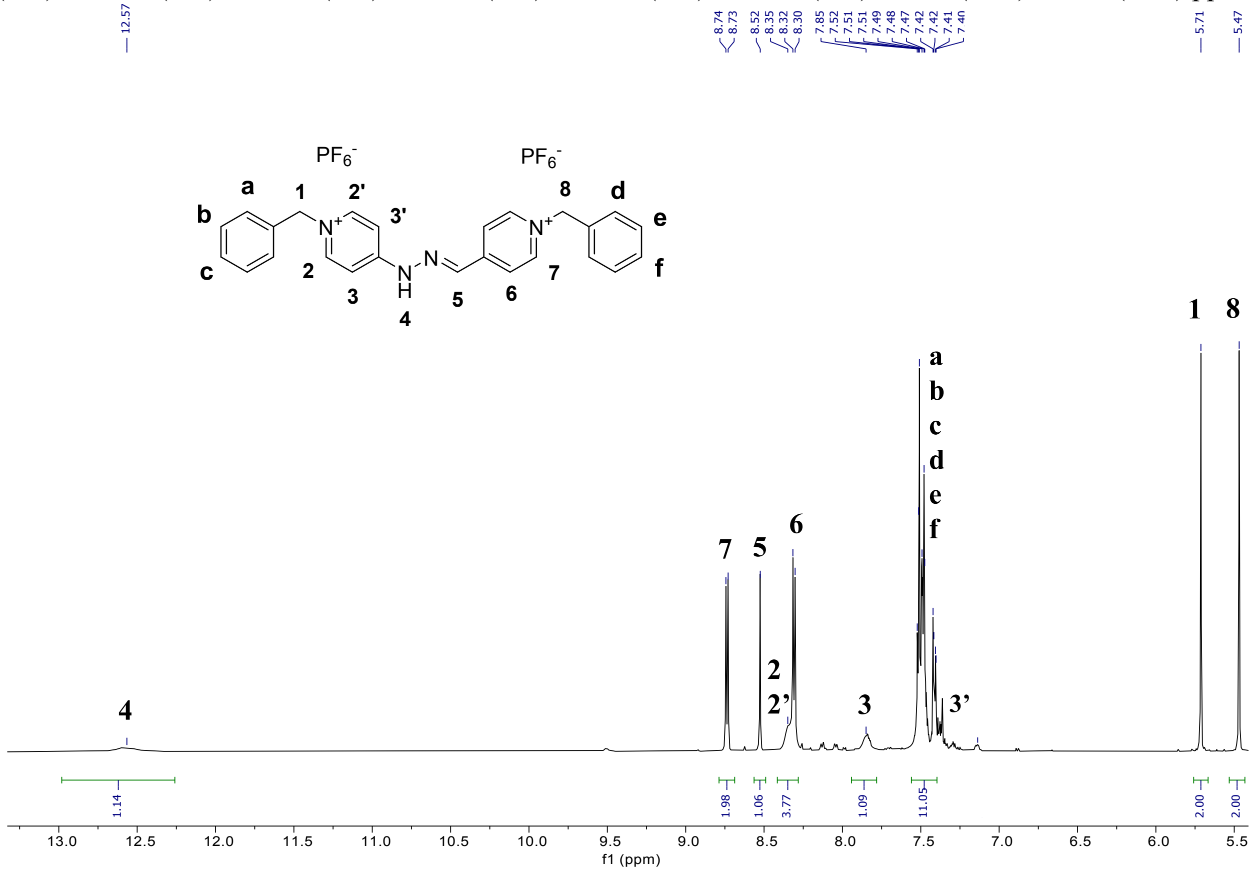

Figure S 17: ^{1}H NMR (500 MHz, CD_3CN) spectrum of **R_bH**$\cdot 2PF_6$.

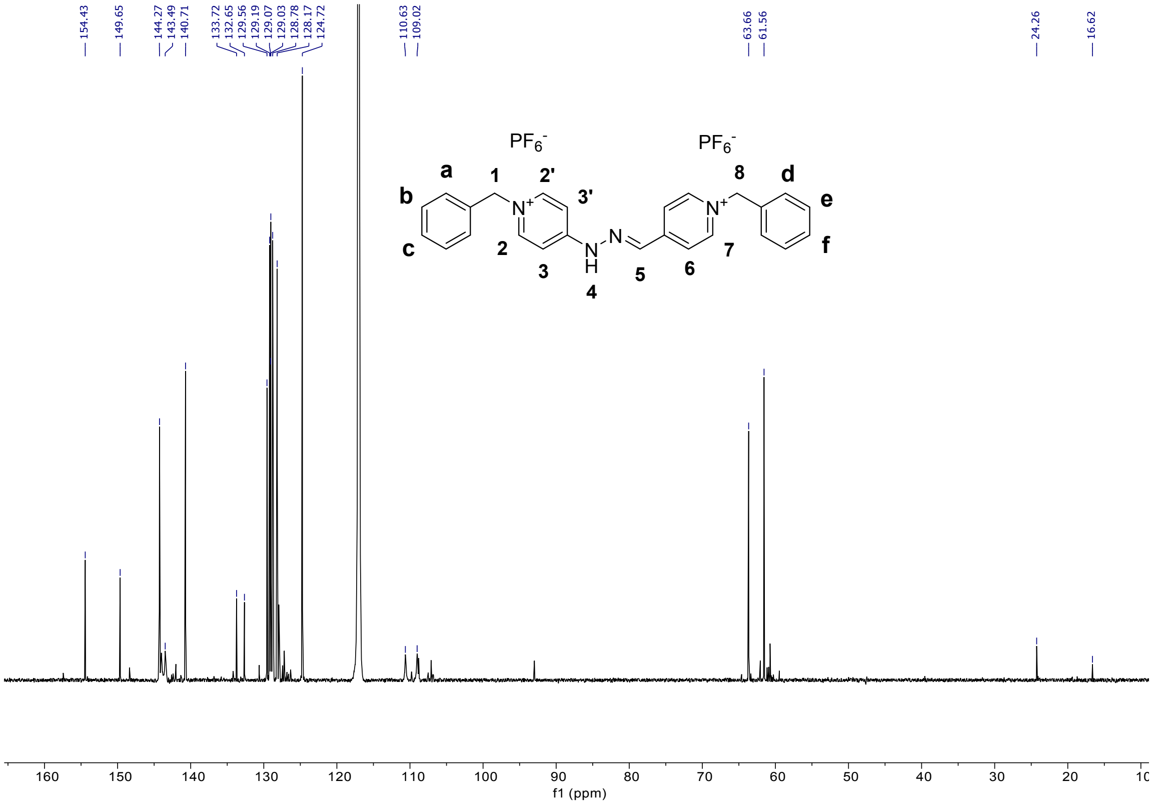

Figure S 18: ^{13}C NMR (126 MHz, CD_3CN) spectrum of $\mathbf{R_bH}\cdot 2PF_6$.

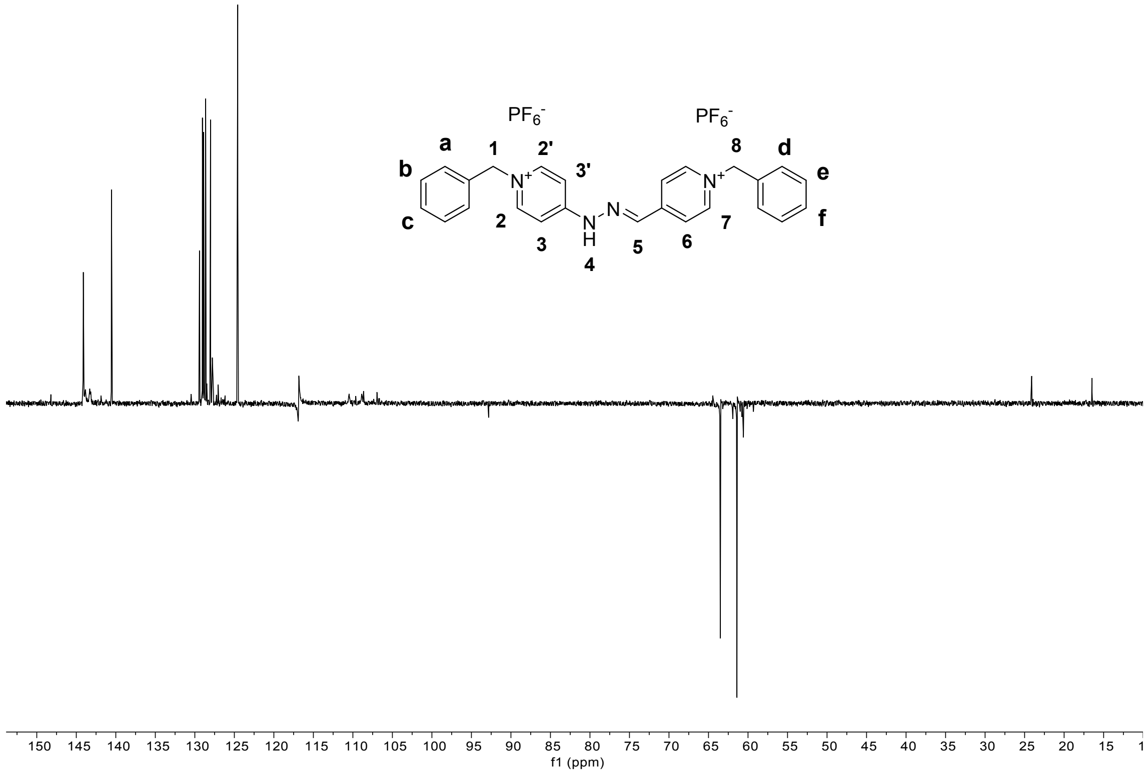

Figure S 19: DEPT-135 (126 MHz, CD_3CN) spectrum of $\mathbf{R_bH}\cdot 2PF_6$.

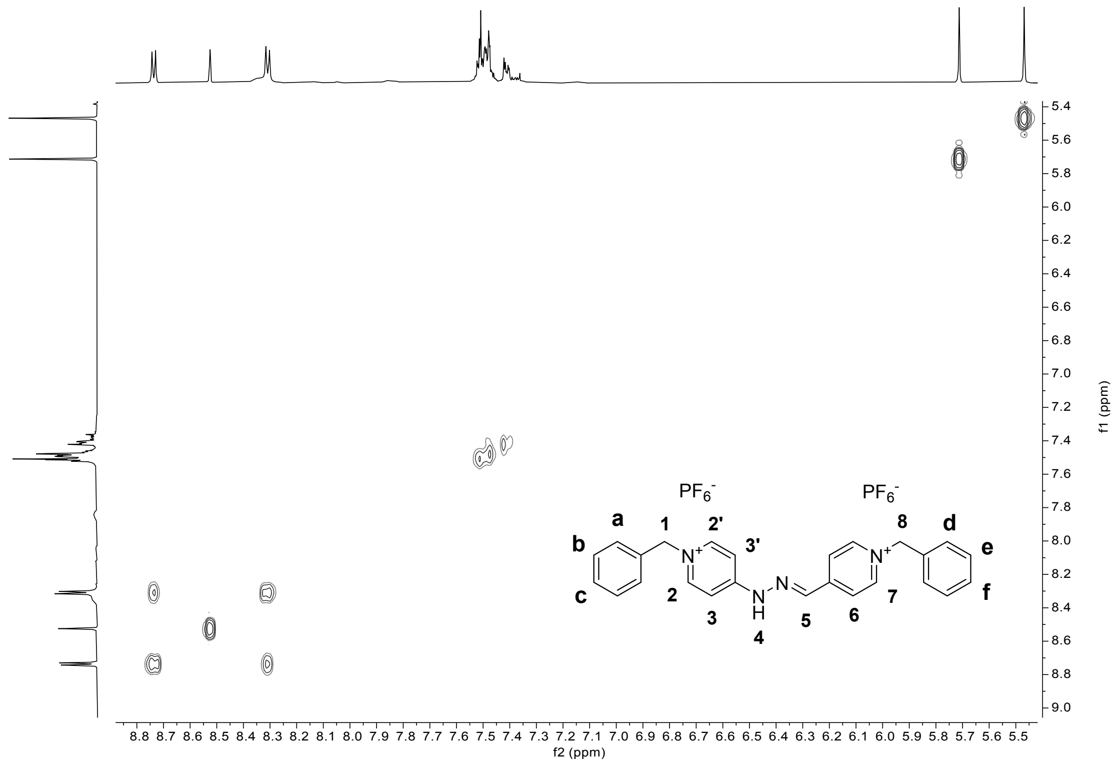

Figure S 20: COSY (500 MHz, CD_3CN) spectrum of $\mathbf{R_bH}\cdot 2PF_6$.

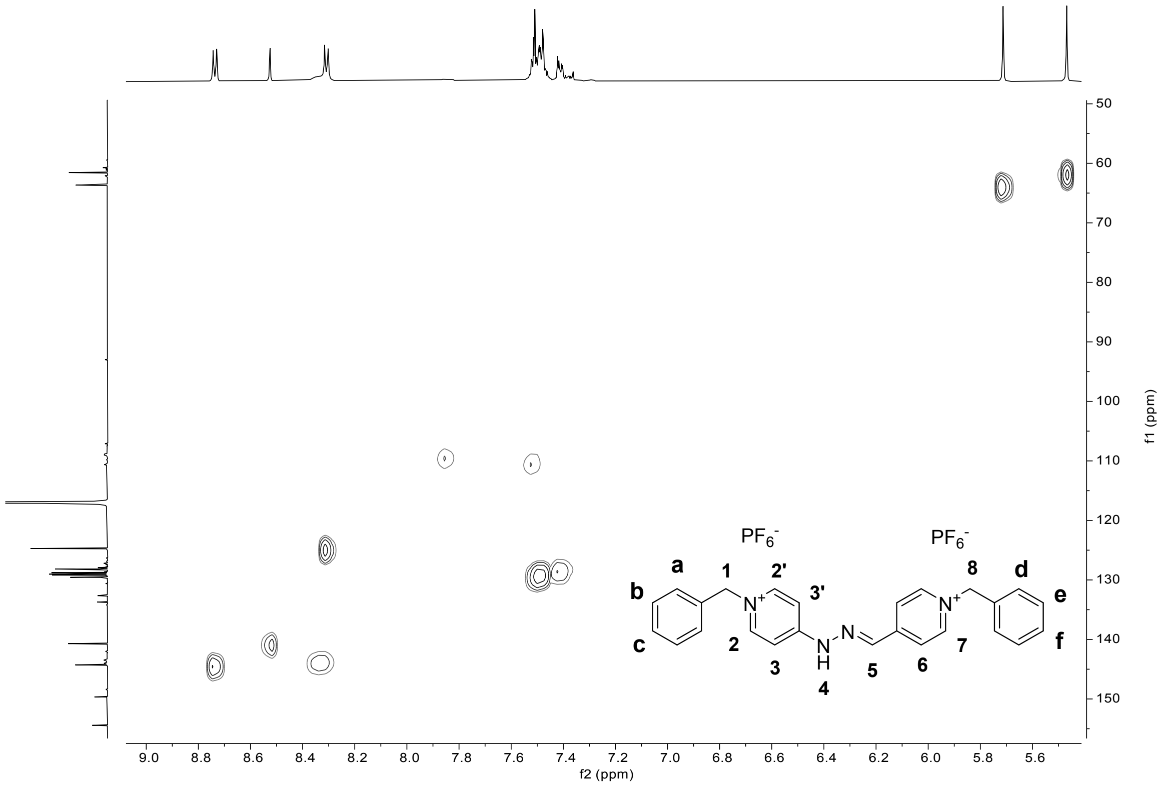

Figure S 21: HSQC (500 and 126 MHz, CD_3CN) spectrum of $\mathbf{R_bH}\cdot 2PF_6$.

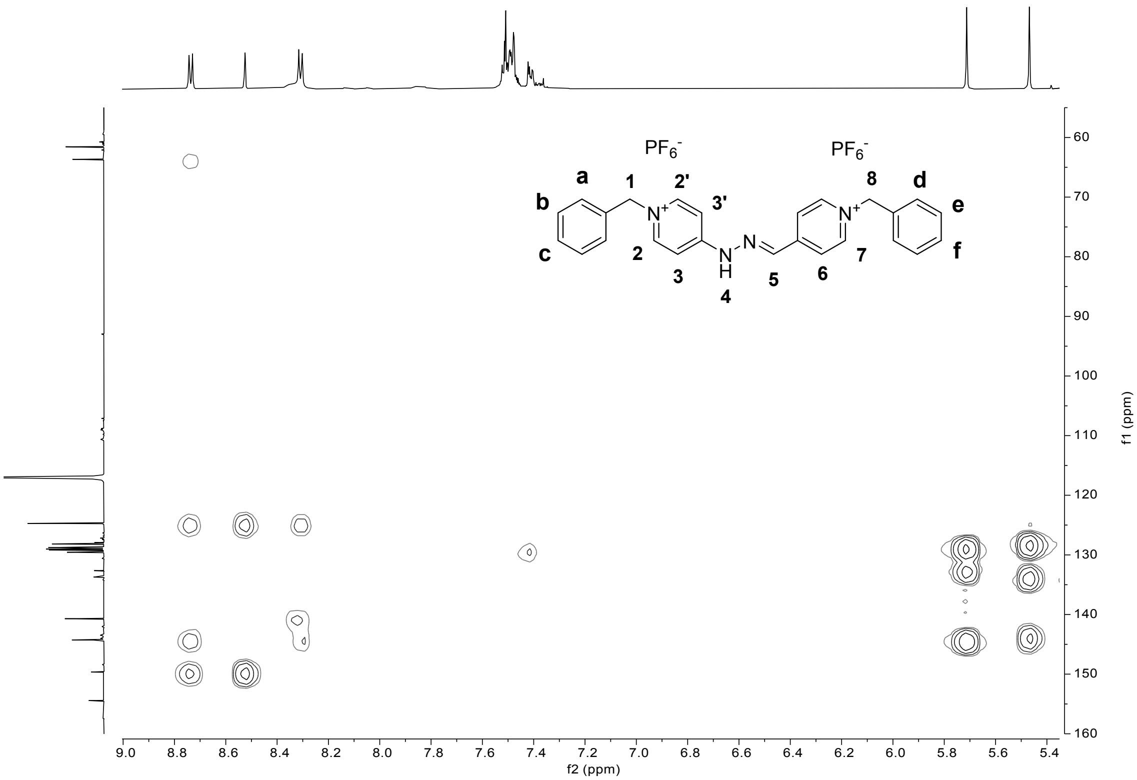

Figure S 22: HMBC (500 and 126 MHz, CD_3CN) spectrum of $\mathbf{R_bH}\cdot 2PF_6$.

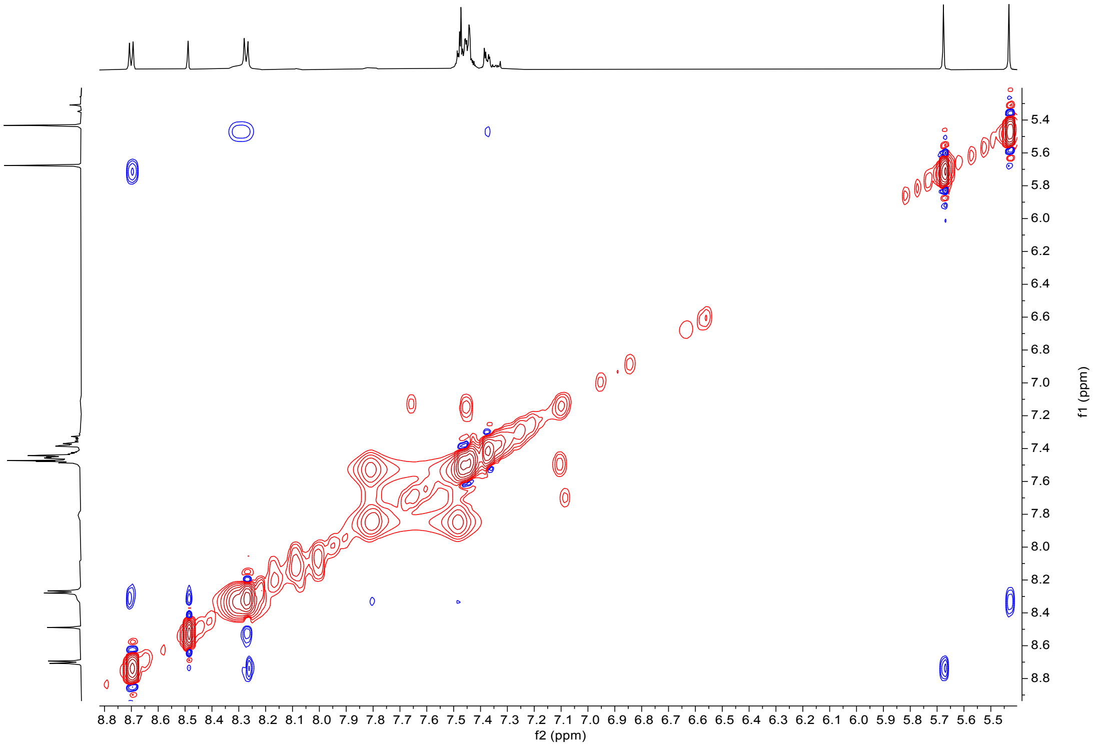

Figure S 23: NOESY (500 MHz, CD_3CN) spectrum of $\mathbf{R_bH}\cdot 2PF_6$.

2.5. Synthesis and characterization data of $\mathbf{R_cH}$·2Cl

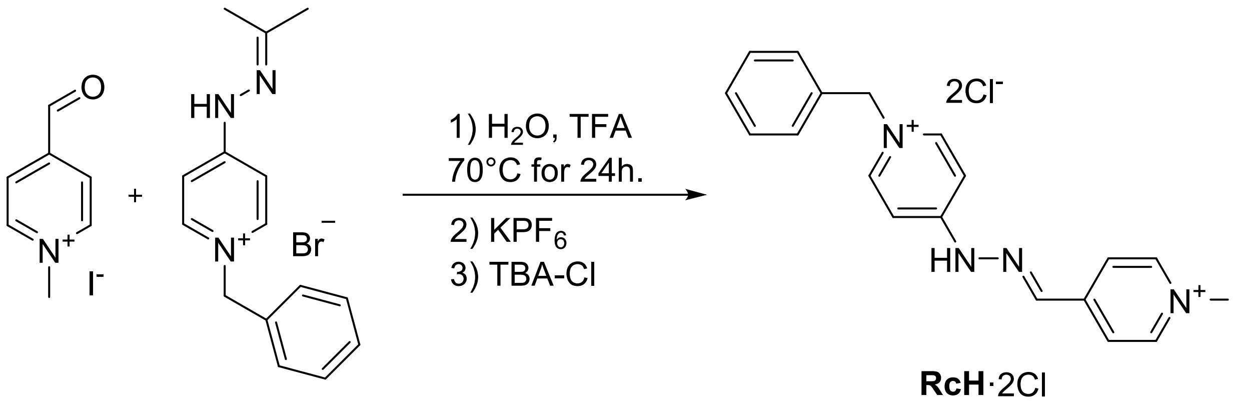

RcH·2Cl

Same synthetic procedure described for $\mathbf{R_bH^{2+}}$.

$\mathbf{R_cH}$·2PF_6: dark orange powder (850 mg, 98%). $\mathbf{R_cH}$·2Cl: yellowish sticky solid (455 mg, 83 %).

^{1}H NMR (500 MHz, D_2O) δ 8.65 (d, J = 6.7 Hz, 2H), 8.32 (s, 2H), 8.26 – 8.16 (m, 3H), 7.78 (s, 1H), 7.43 – 7.28 (m, 5H), 7.10 (s, 1H), 5.41 (s, 2H), 4.26 (s, 3H) ppm. **^{13}C NMR** (126 MHz, D_2O) δ 154.47 (C), 149.15 (C), 145.27 (CH), 143.87 (CH), 141.29 (CH), 133.82 (C=N), 129.56 (C), 129.42 (CH), 128.38 (CH), 124.46 (CH), 110.52 (CH), 109.17 (CH), 61.85 (CH_2), 47.64 (CH_3) ppm. HRMS (ESI) (*m/z*): calcd for $[C_{19}H_{20}N_4\text{-}H]^+$ 303.1605, found 303.1605.

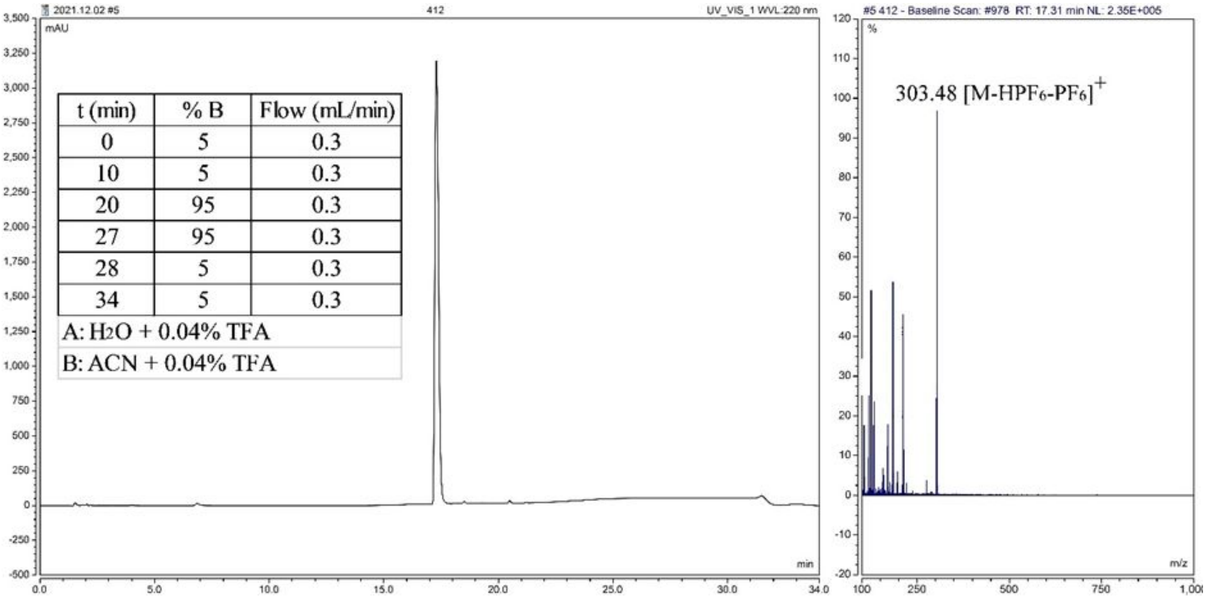

Figure S 24. HPLC-MS chormatogram of $\mathbf{R_cH}$·2PF_6. *Inset.* Elution conditions.

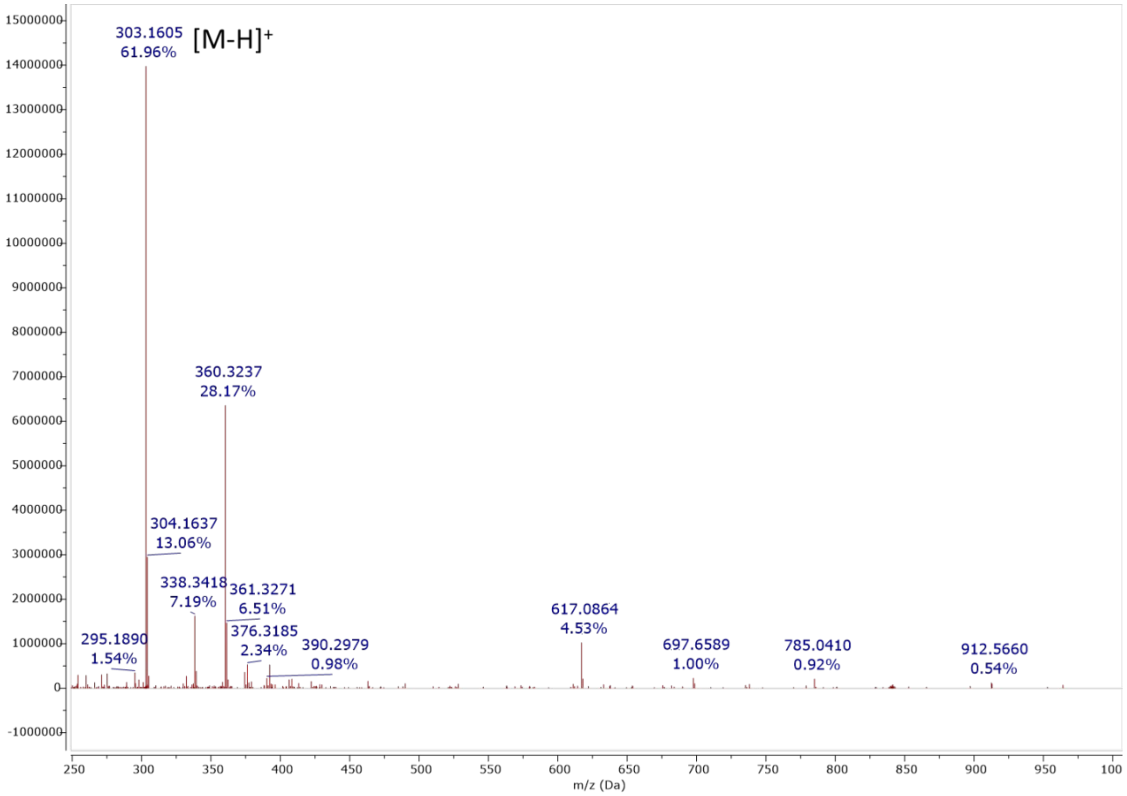

Figure S 25. HR ESI-MS spectrum of $\mathbf{R_c}$H·2PF$_6$.

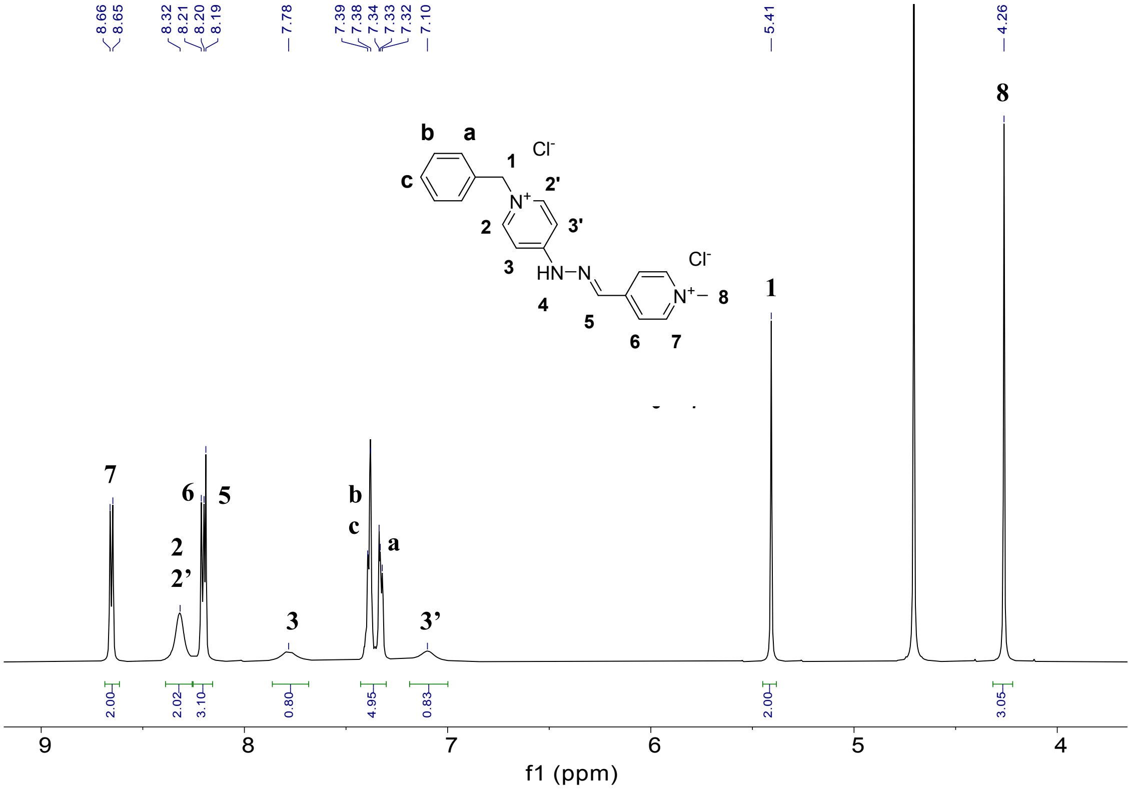

Figure S 26: ^{1}H NMR (500 MHz, D$_2$O) spectrum of $\mathbf{R_cH}$·2Cl.

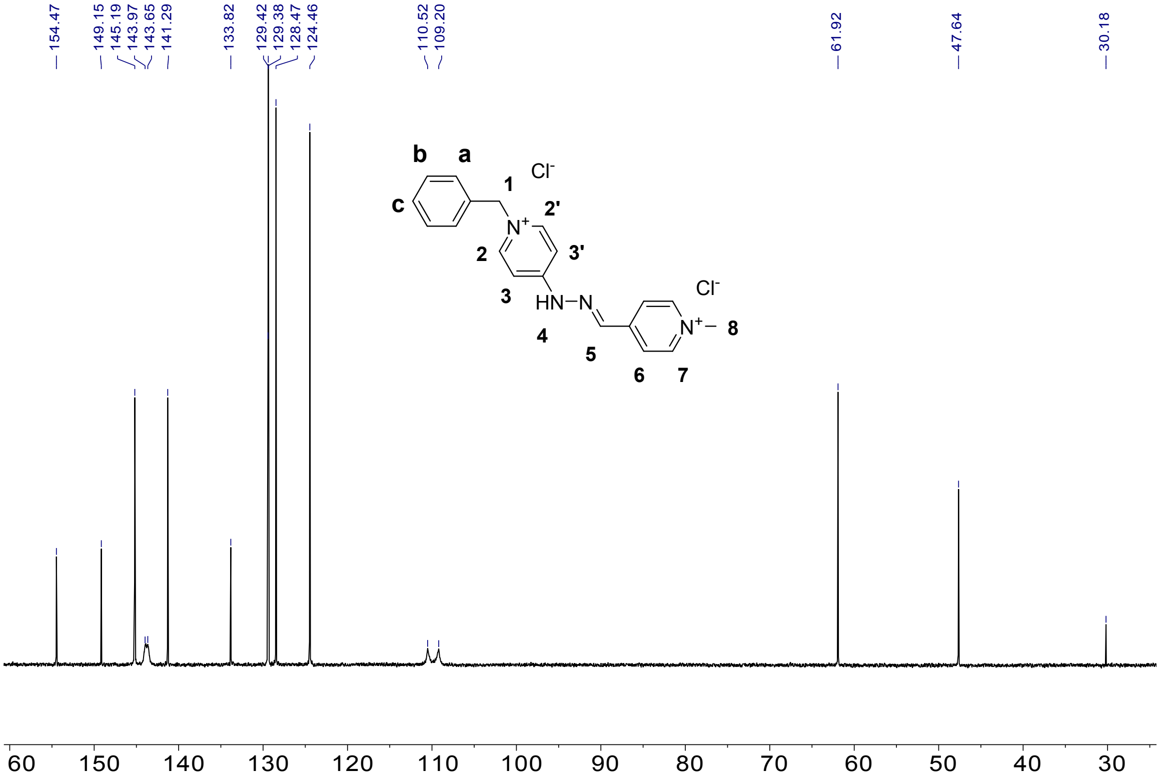

Figure S 27: ^{13}C NMR (126 MHz, D_2O) spectrum of $\mathbf{R_cH}$·2Cl.

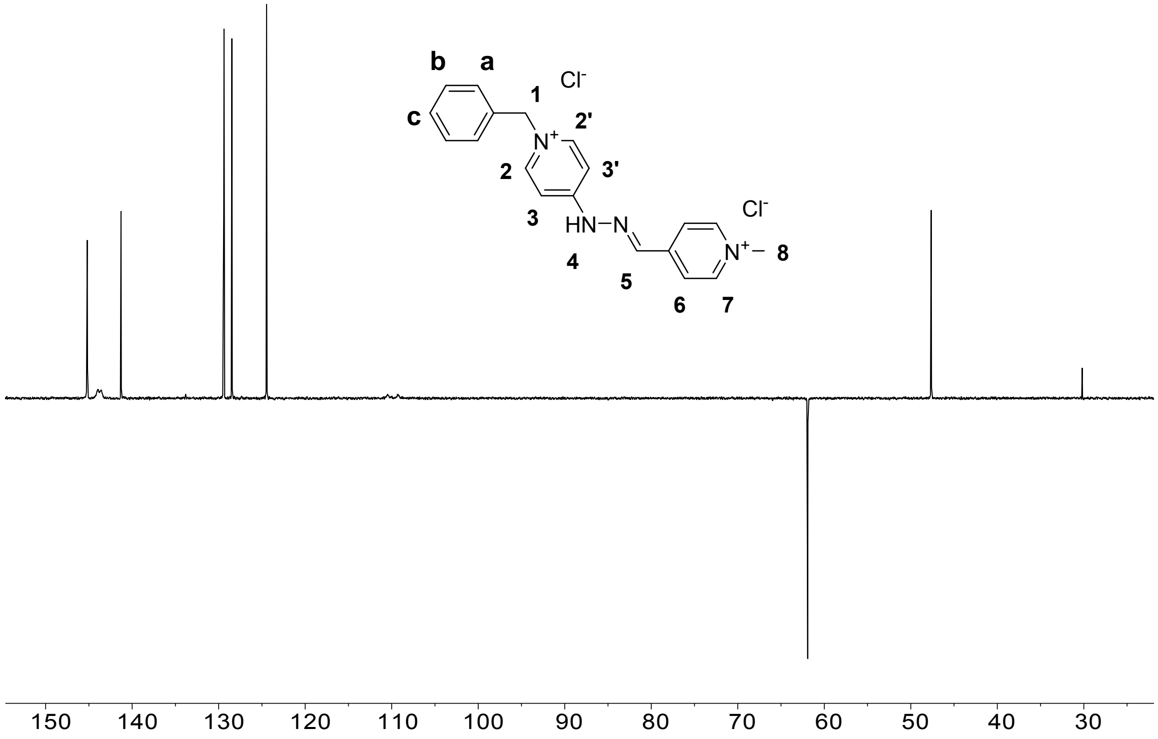

Figure S 28: ^{13}C NMR (126 MHz, D_2O) spectrum of $\mathbf{R_cH}$·2Cl.

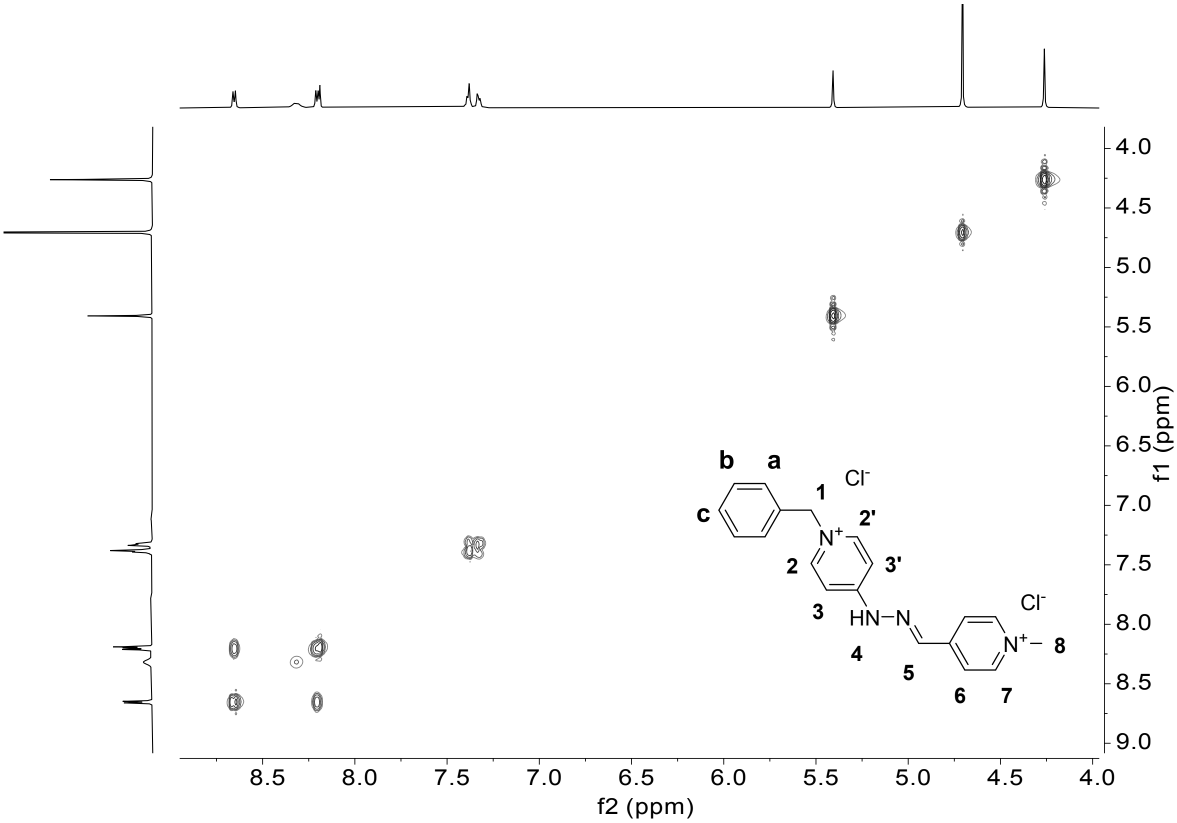

Figure S 29: COSY (500 MHz, D_2O) spectrum of $\mathbf{R_cH}$·2Cl.

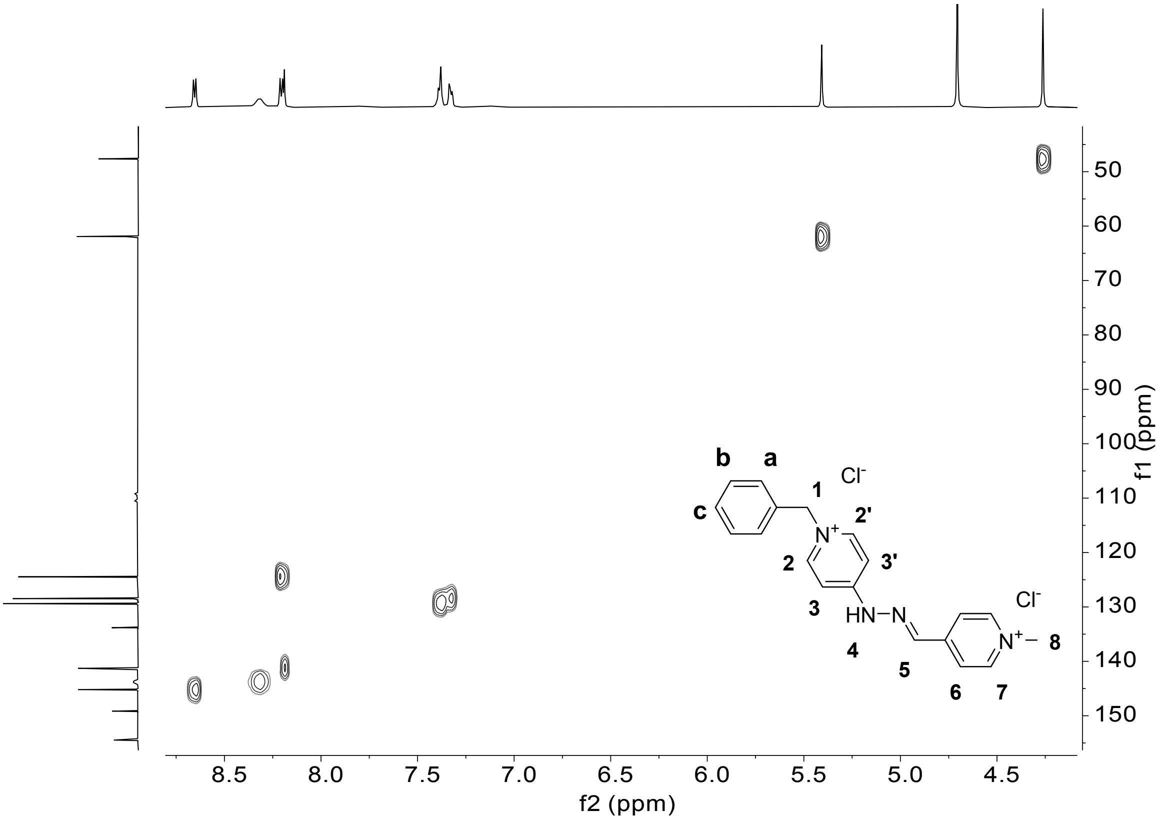

Figure S 30: HSQC (500 and 126 MHz, D_2O) spectrum of $\mathbf{R_cH}$·2Cl.

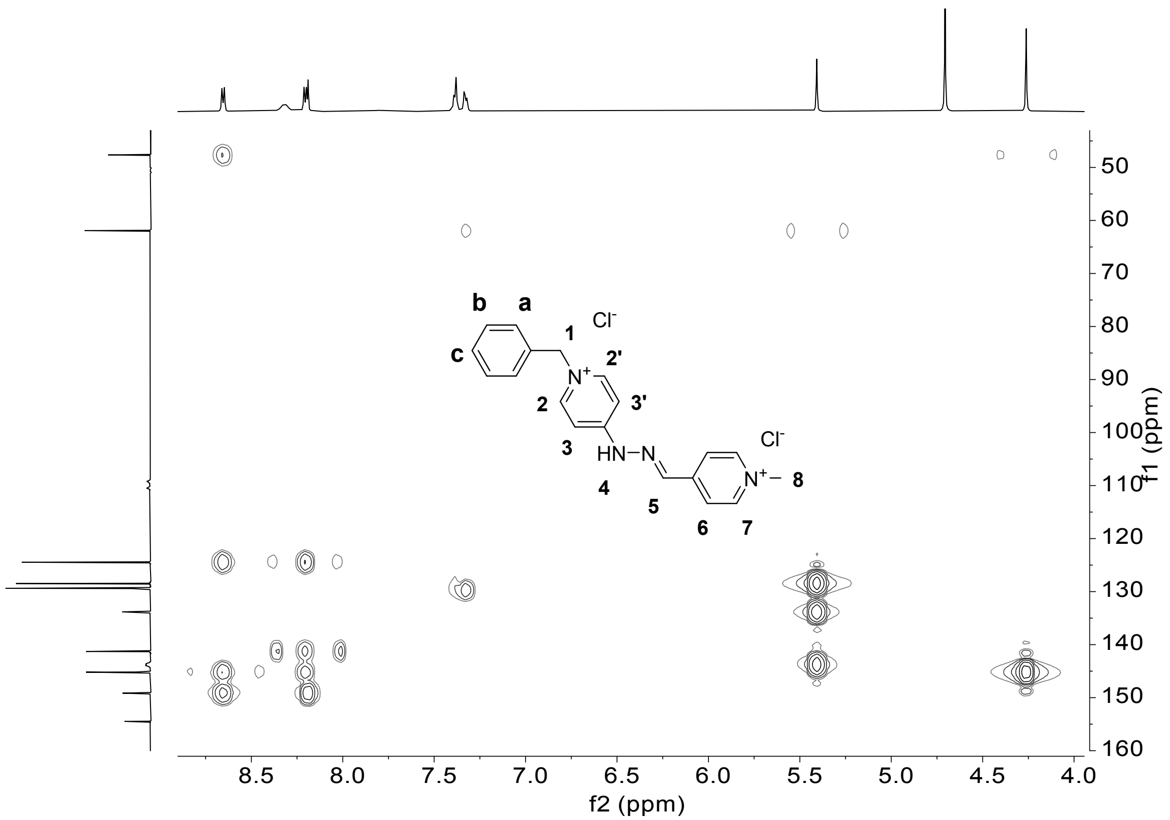

Figure S 31: HMBC (500 and 126 MHz, D_2O) spectrum of $\mathbf{R_cH}$·2Cl.

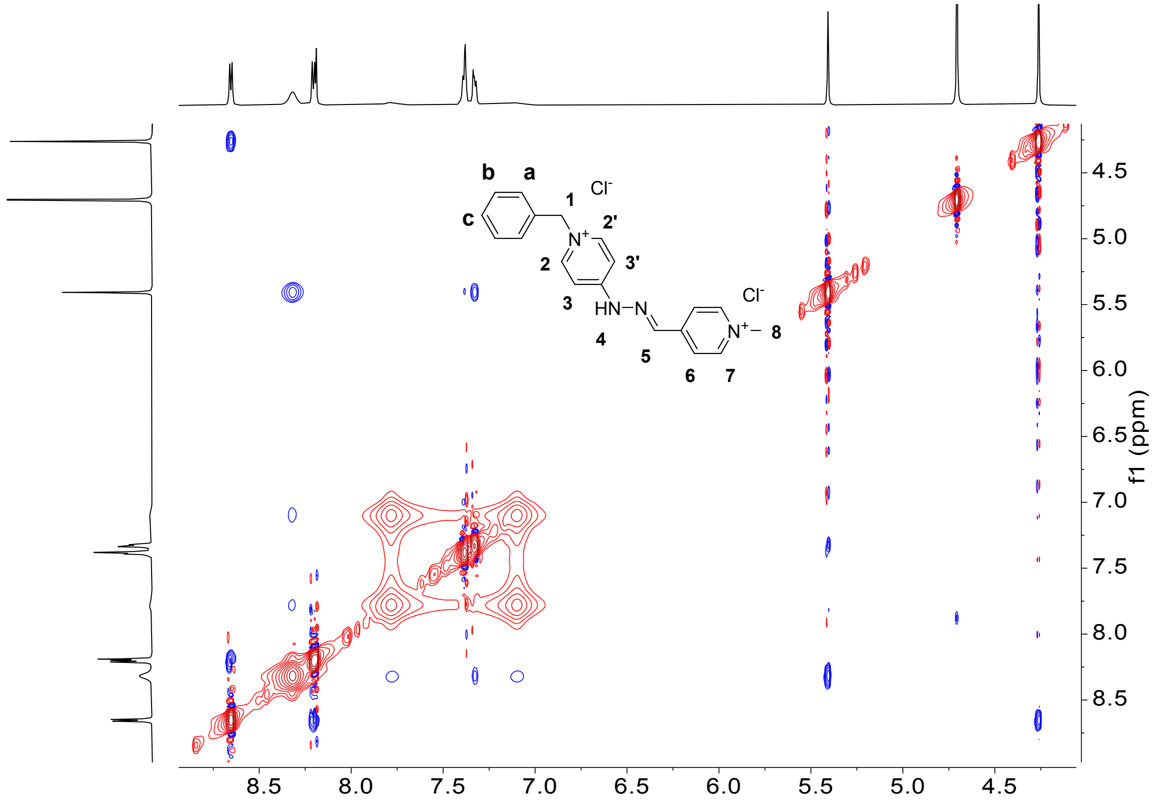

Figure S 32: NOESY (500 MHz, D_2O) spectrum of $\mathbf{R_cH}$·2Cl.

- **R_c·Cl at pD = 12.**

^{1}H NMR (300 MHz, D_2O) δ 8.36 (d, *J* = 5.9 Hz, 2H), 8.14 – 7.90 (m, 3H), 7.73 (d, *J* = 7.0 Hz, 2H), 7.54 – 7.21 (m, 6H), 6.60 (s, 1H), 5.11 (s, 2H), 4.11 (s, 3H) ppm.

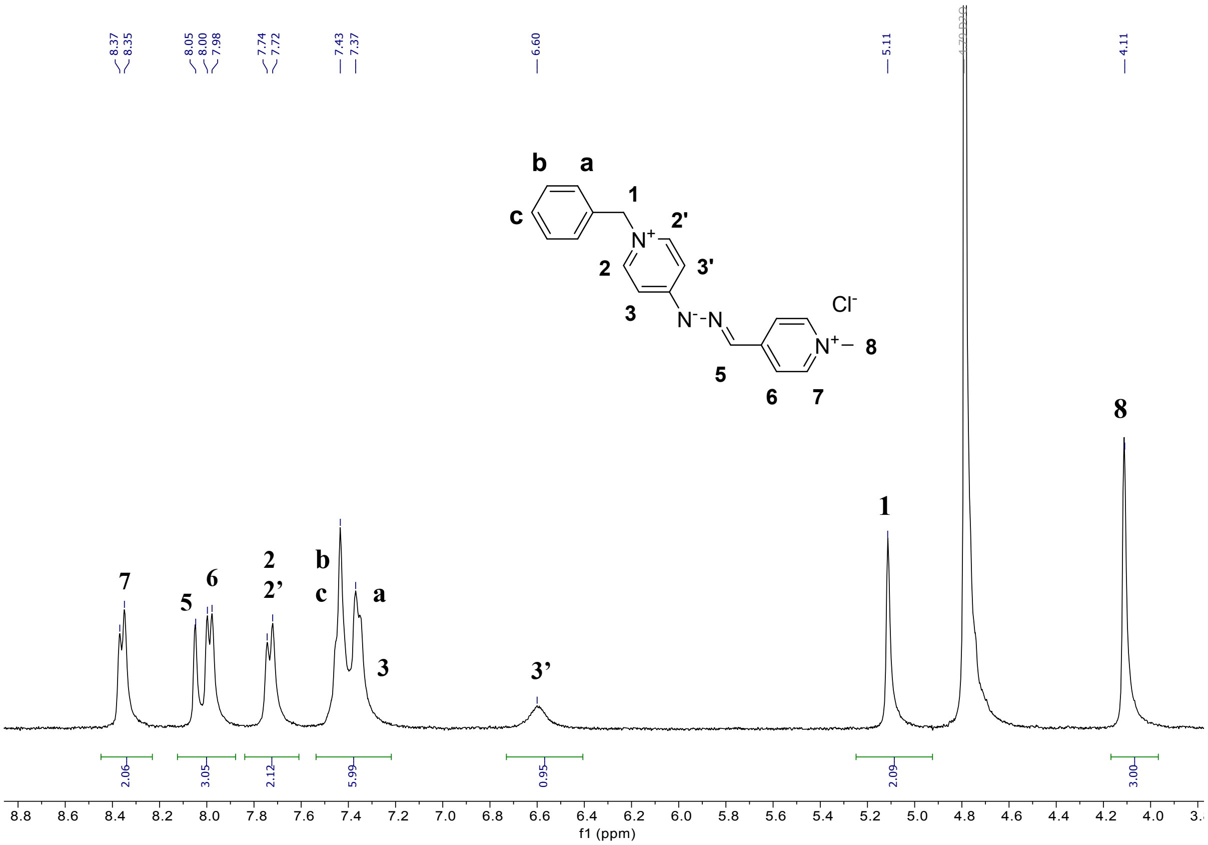

Figure S 33: ^{1}H NMR (300 MHz, D_2O) spectrum of **R_c**·Cl.

- **$R_cH{\cdot}2PF_6$:**

^{1}H NMR (500 MHz, CD_3CN) δ 10.84 (s, 1H), 8.74 – 8.55 (m, 2H), 8.38 (s, 1H), 8.31 – 8.27 (m, 2H), 8.24 (s, 1H), 7.86 (s, 1H), 7.54 – 7.37 (m, 5H), 7.27 (s, 1H), 5.49 (s, 2H), 4.30 (s, 3H) ppm. **^{13}C NMR** (126 MHz, CD_3CN) δ 154.20 (C), 148.66 (C), 145.24 (CH), 144.16 (CH), 143.70 (CH), 141.02 (C=N), 133.65 (C), 129.13 (CH), 129.07 (CH), 128.22 (CH), 124.30 (CH), 110.52 (CH), 109.17 (CH), 61.65 (CH_2), 47.74 (CH_3) ppm.

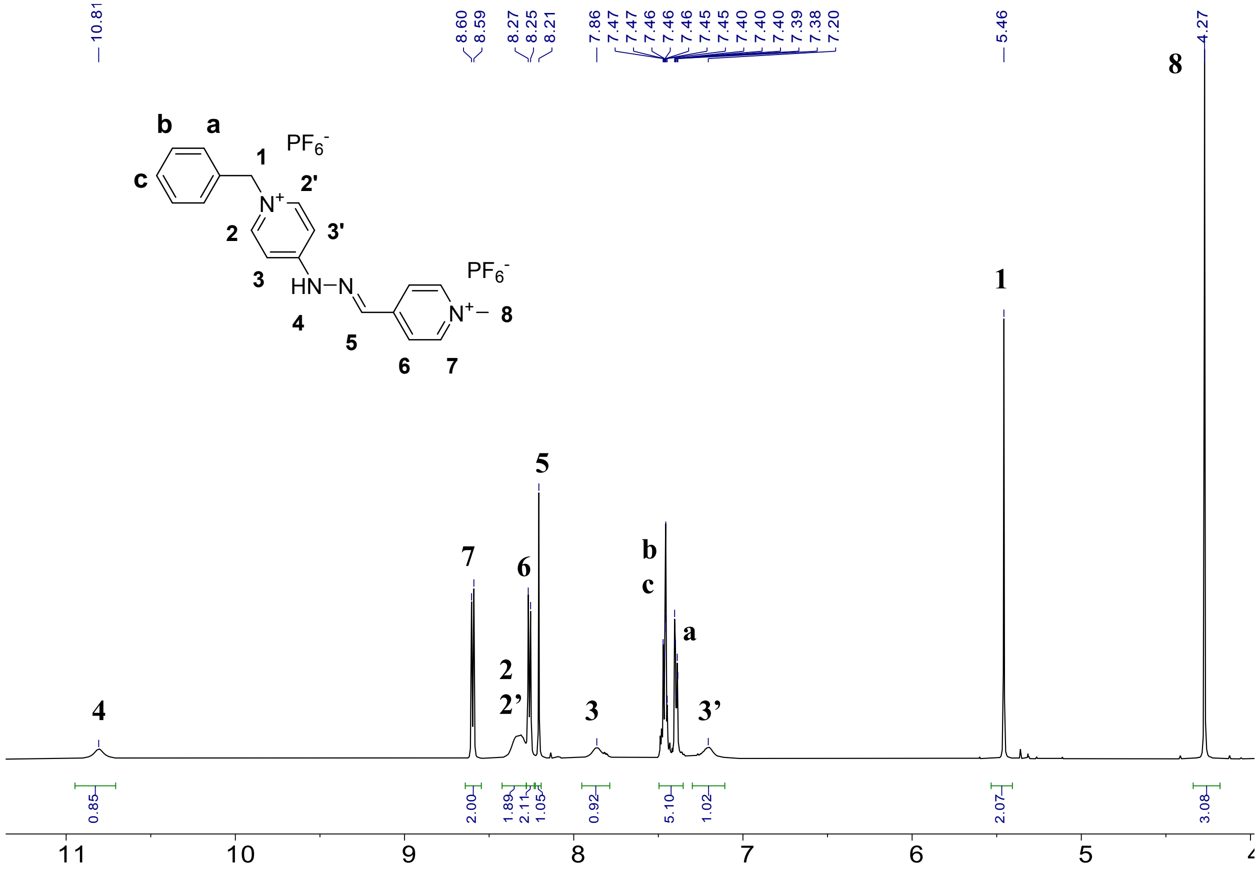

Figure S 34: ^{1}H NMR (500 MHz, CD_3CN) spectrum of $\mathbf{R_cH}{\cdot}2PF_6$.

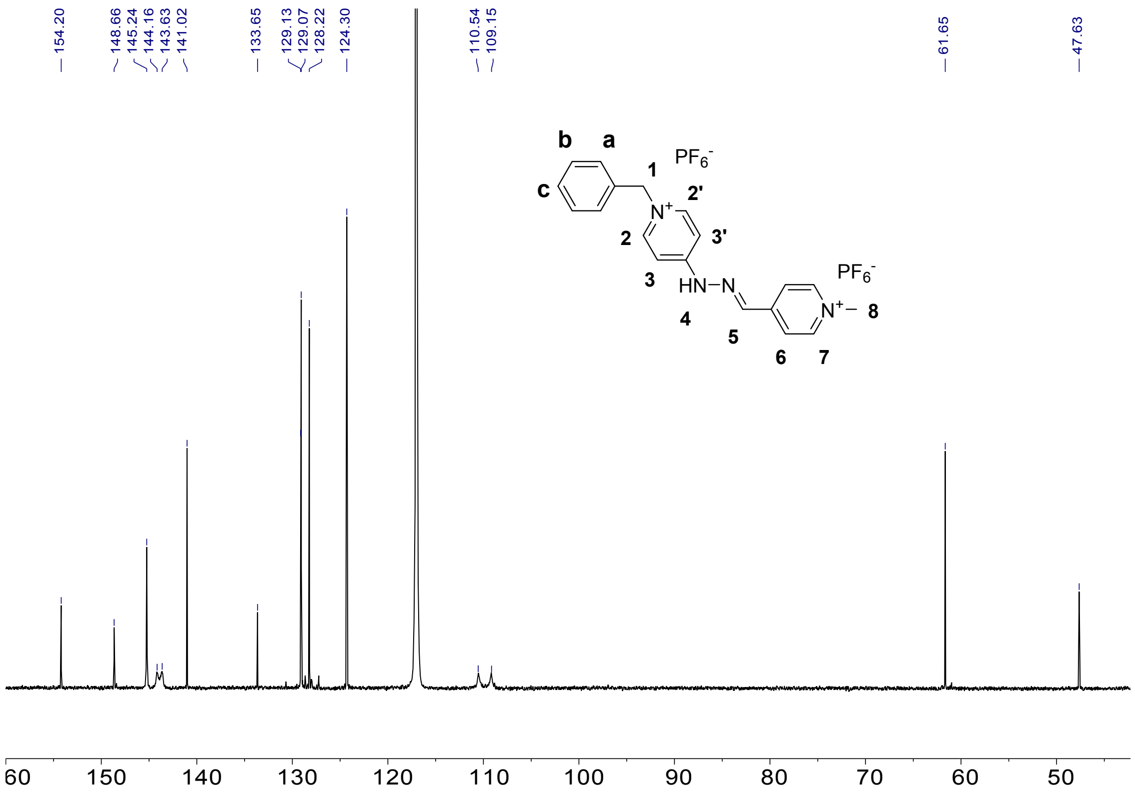

Figure S 35: ^{13}C NMR (126 MHz, CD_3CN) spectrum of **R$_c$H**·$2PF_6$.

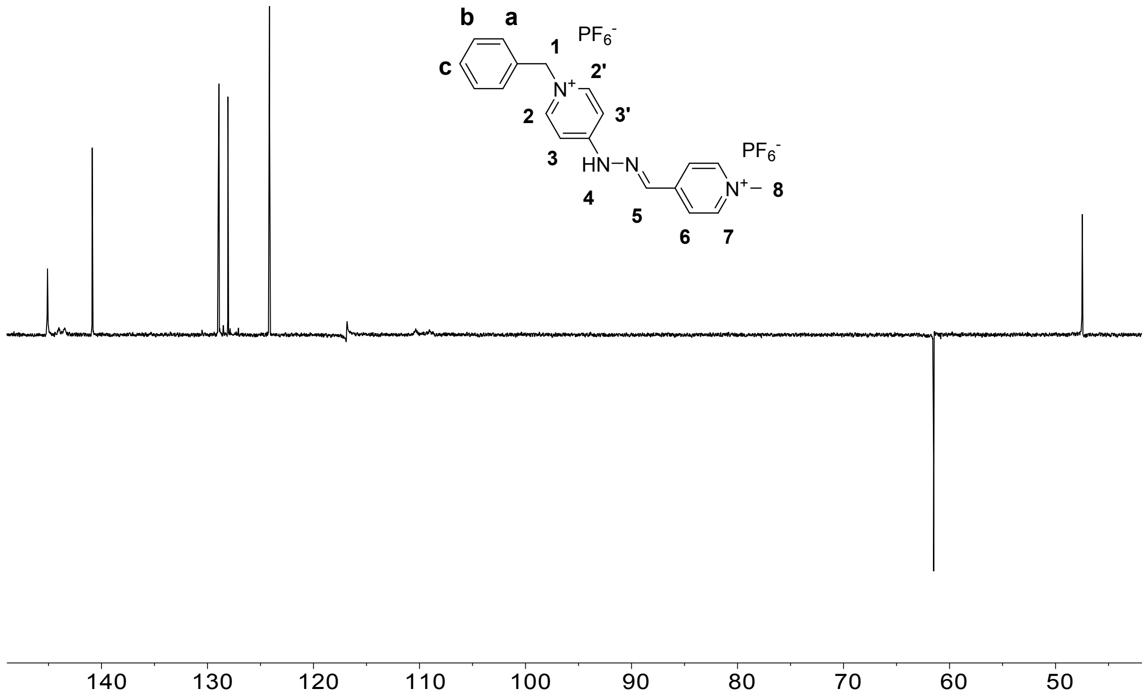

Figure S 36: DEPT-135 (126 MHz, CD_3CN) spectrum of **R$_c$H**·$2PF_6$.

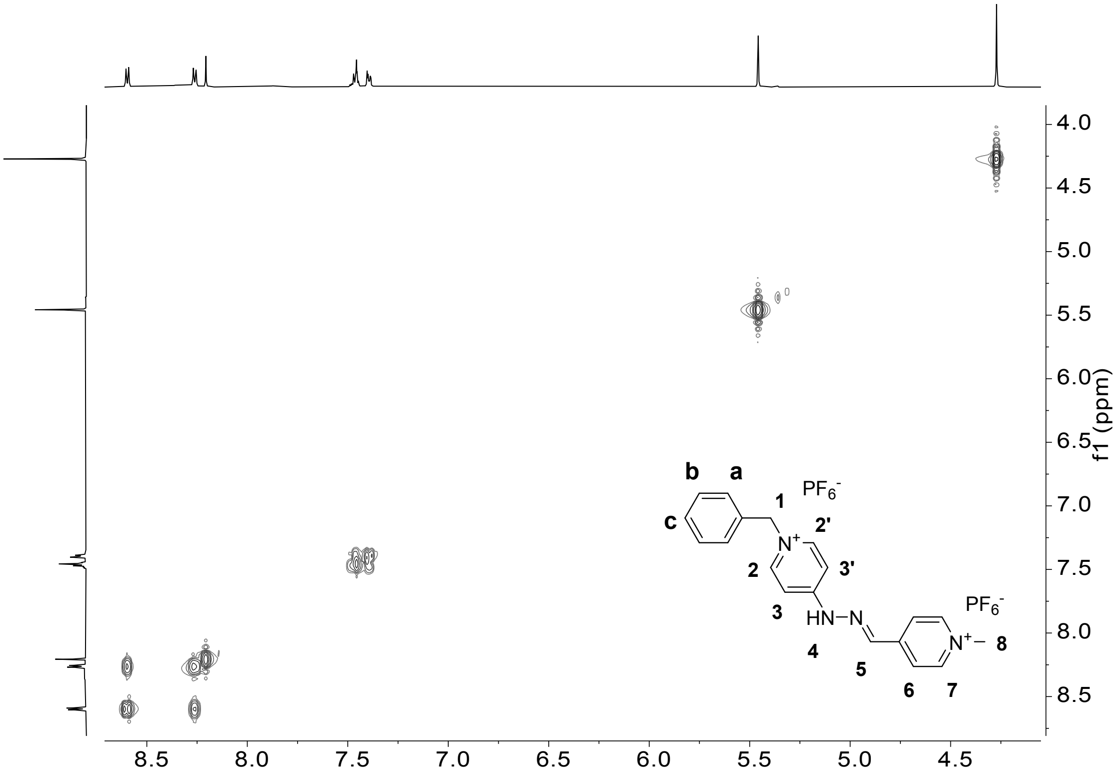

Figure S 37: COSY (500 MHz, CD_3CN) spectrum of $\mathbf{R_cH}\cdot 2PF_6$.

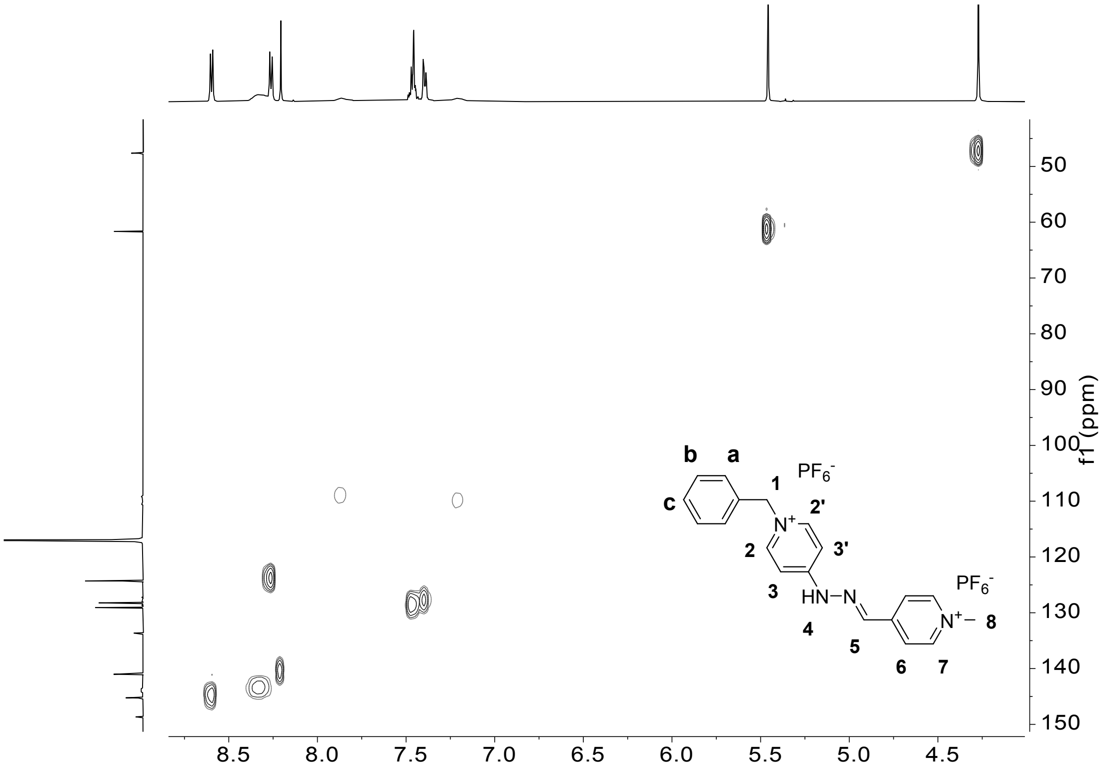

Figure S 38: HSQC (500 and 126 MHz, CD_3CN) spectrum of $\mathbf{R_cH}\cdot 2PF_6$.

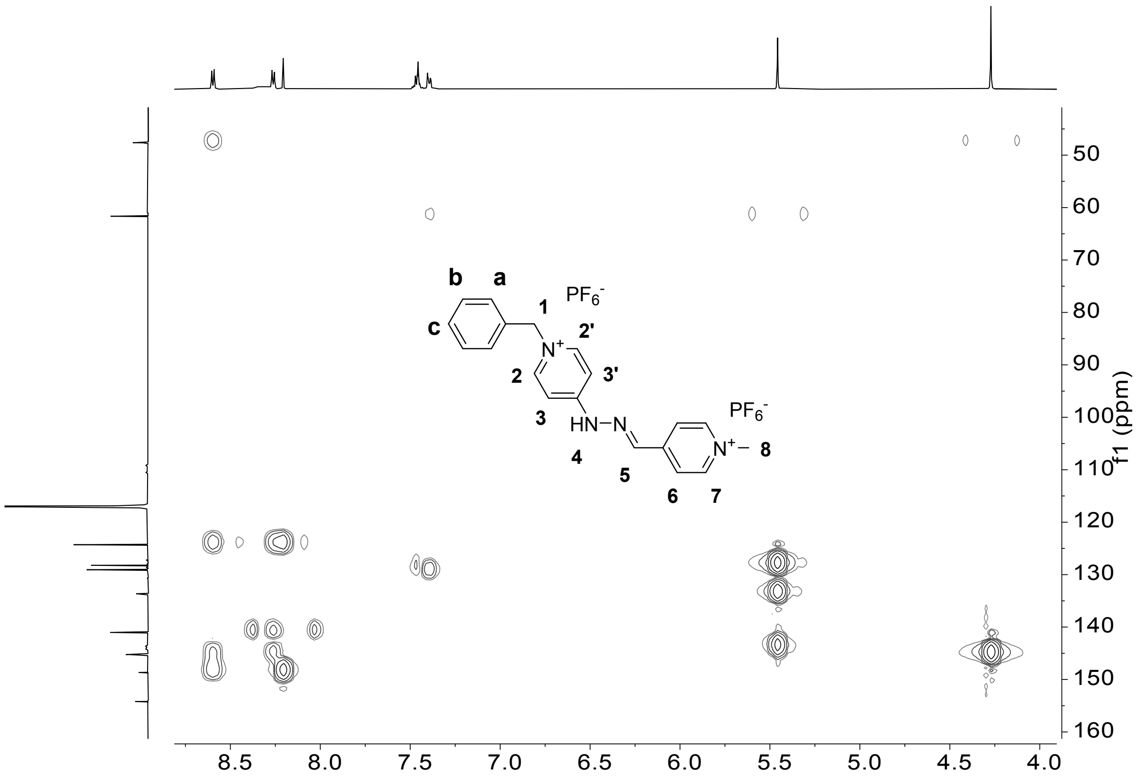

Figure S 39: HMBC (500 and 126 MHz, CD_3CN) spectrum of $\mathbf{R_cH}\cdot 2PF_6$.

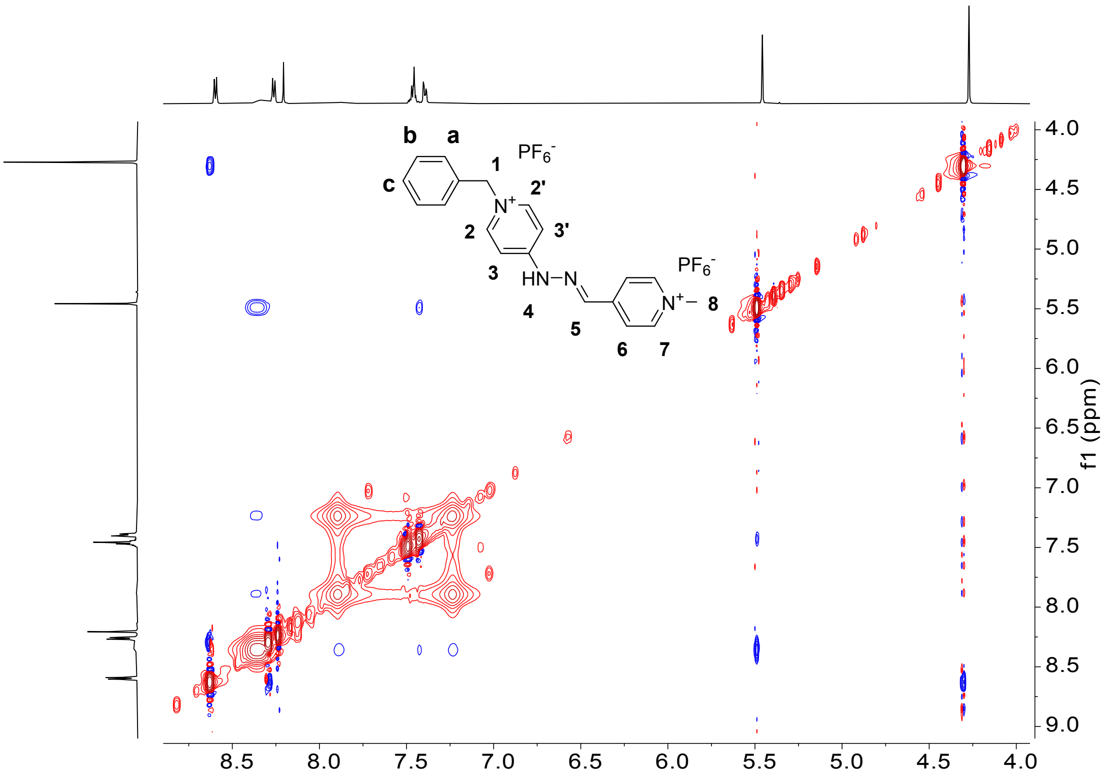

Figure S 40: NOESY (500 MHz, CD_3CN) spectrum of $\mathbf{R_cH}\cdot 2PF_6$.

2.6. Synthesis and characterization data of $R_dH \cdot 2Cl$

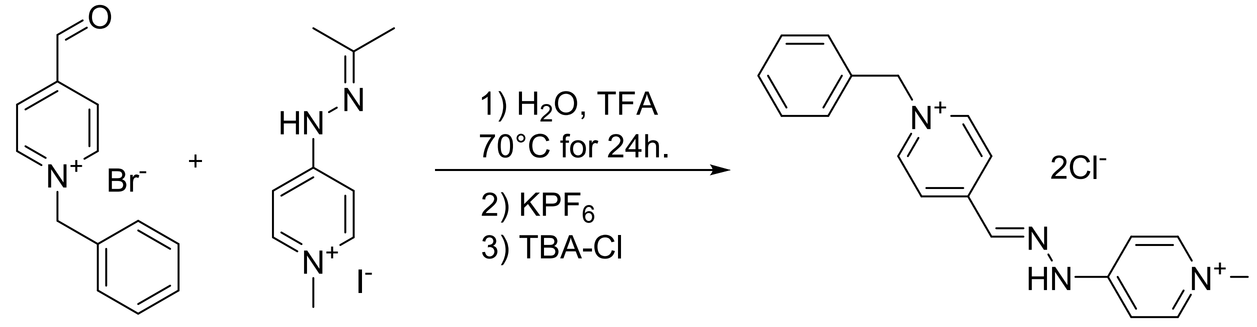

3bH·Br **RdH**·2Cl

Same synthetic procedure described for $\mathbf{R_bH^{2+}}$.

$\mathbf{R_dH}\cdot 2PF_6$: dark orange powder (850 mg, 79 %). $\mathbf{R_dH}\cdot 2Cl$: yellow solid (614 mg, 91 %).

^{1}H NMR (500 MHz, D_2O) δ 8.88 (d, J = 6.6 Hz, 2H), 8.39 – 8.26 (m, 5H), 7.69 – 7.42 (m, 5H), 5.80 (s, 2H), 4.10 (s, 3H) ppm. **^{13}C NMR** (126 MHz, D_2O) δ 149.91 (C), 147.21 (C), 144.54 (CH), 144.38 (CH), 140.69 (C=N), 132.70 (C), 129.87 (CH), 129.58 (CH), 128.99 (CH), 124.71 (CH), 110.14 (CH), 109.17 (CH), 64.12 (CH_2), 45.52 (CH_3) ppm. **HRMS (ESI)** (*m/z*): calcd for $[C_{19}H_{20}N_4\text{-}H]^+$ 303.1605, found 303.1605.

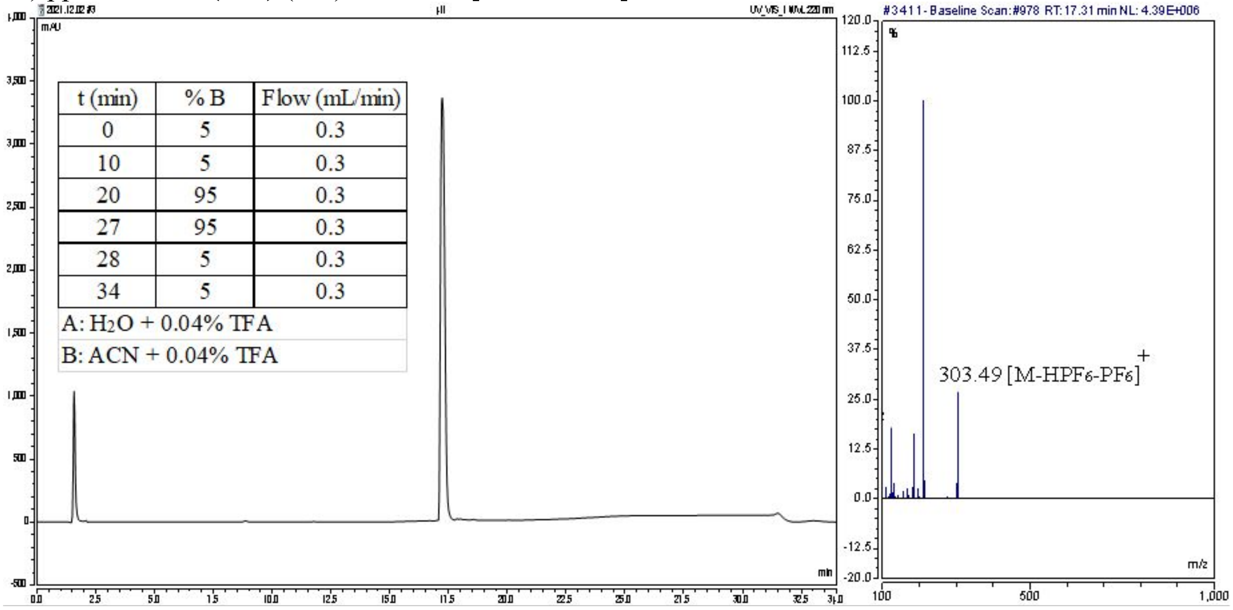

t (min)	% B	Flow (mL/min)
0	5	0.3
10	5	0.3
20	95	0.3
27	95	0.3
28	5	0.3
34	5	0.3

A: H_2O + 0.04% TFA
B: ACN + 0.04% TFA

Figure S 41. HPLC-MS chromatogram of $\mathbf{R_dH}\cdot 2PF_6$. *Inset.* Table of the elution conditions.

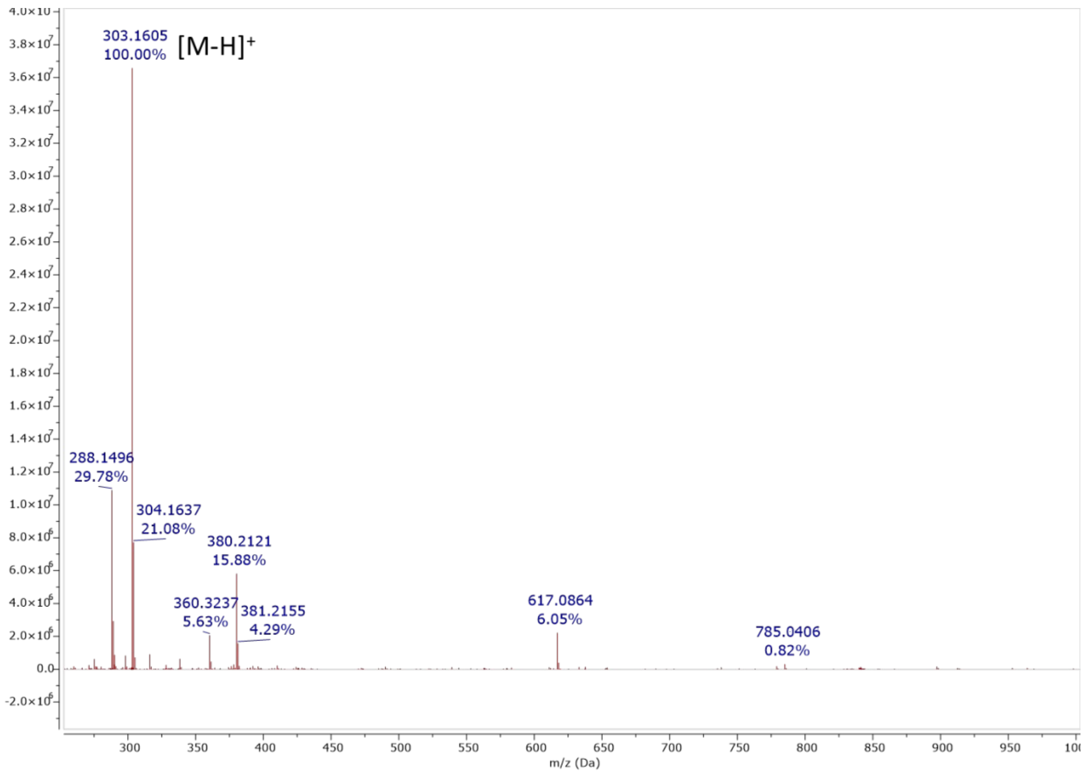

Figure S 42. HR ESI-MS spectrum of $\mathbf{R_d}$H·2PF_6.

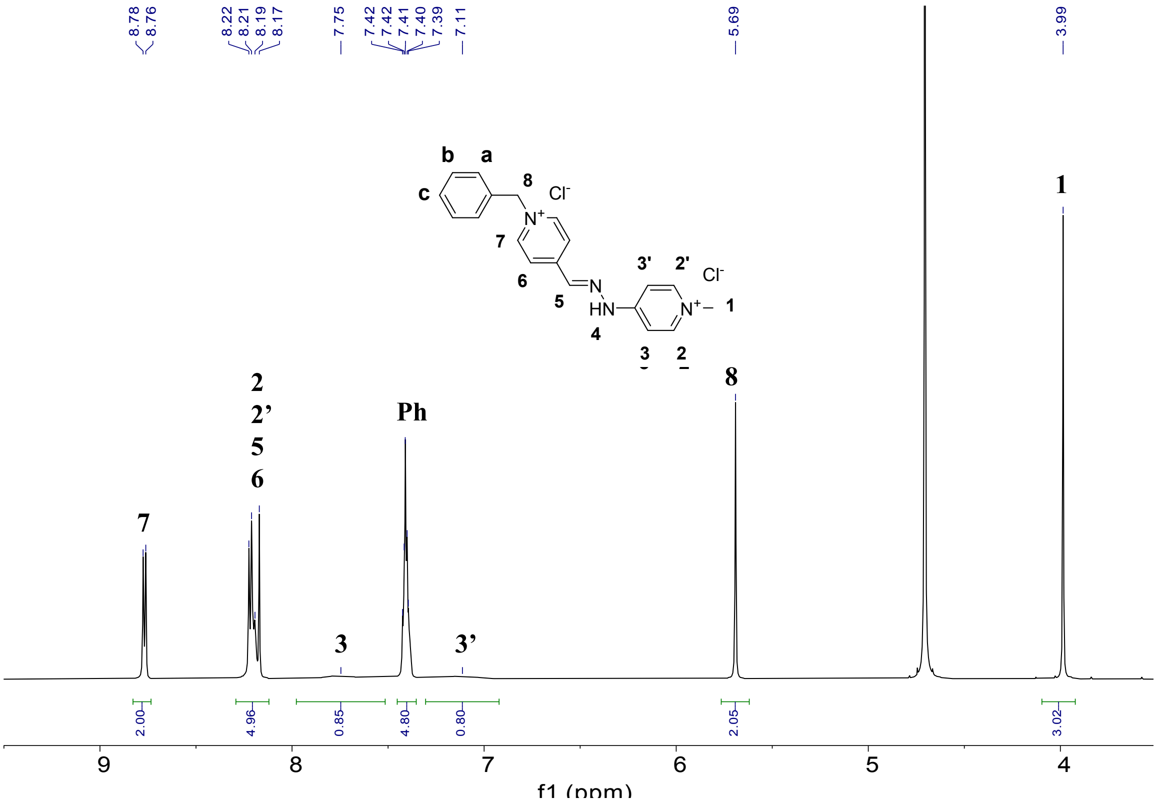

Figure S 43: ^{1}H NMR (500 MHz, D_2O) spectrum of $\mathbf{R_dH}$·2Cl.

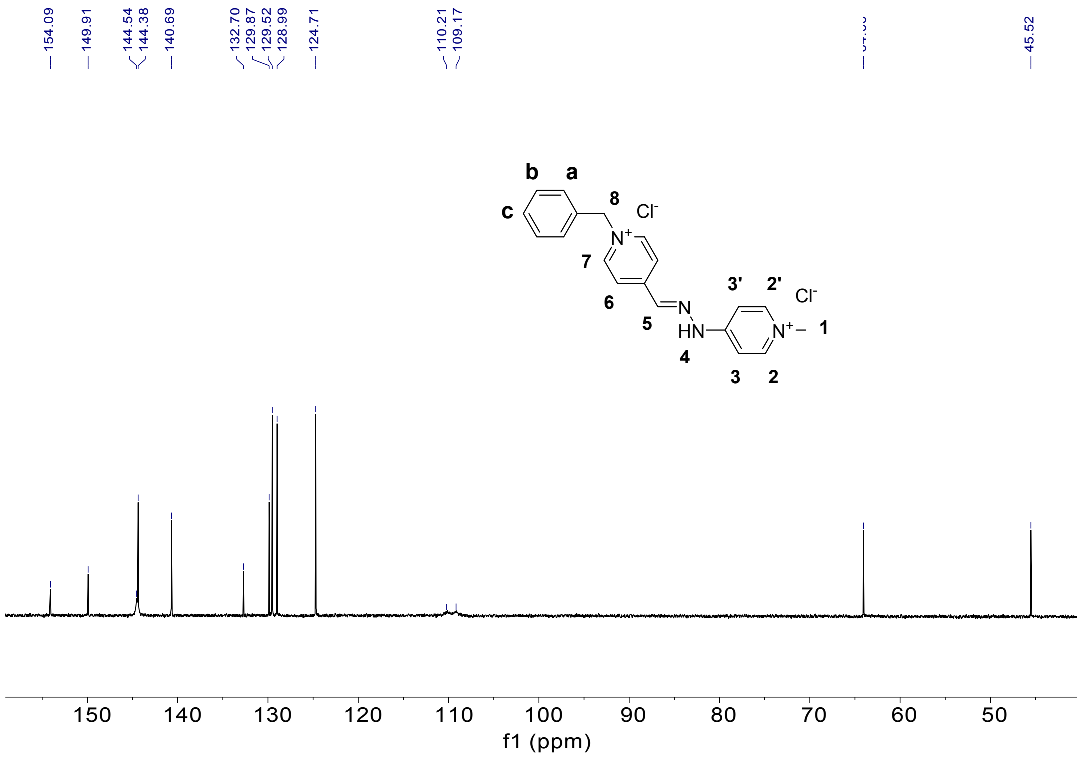

Figure S 44: ^{13}C NMR (126 MHz, D_2O) spectrum of $\mathbf{R_dH}\cdot 2Cl$.

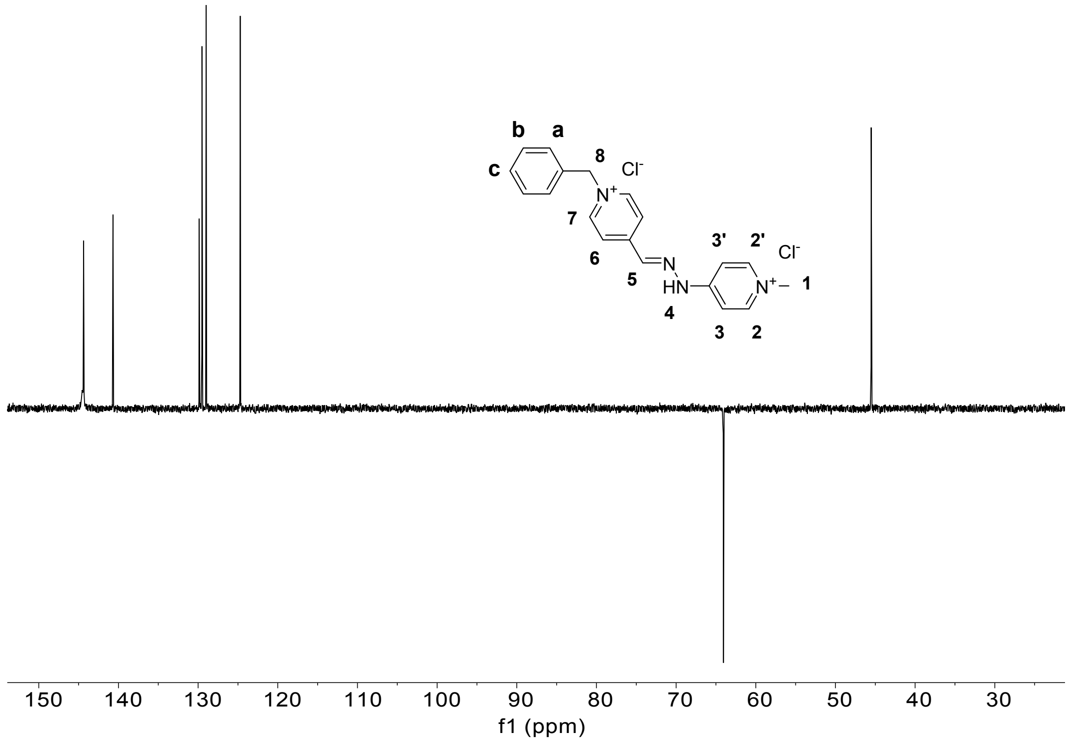

Figure S 45: DEPT-135 (126 MHz, D_2O) spectrum of $\mathbf{R_dH}\cdot 2Cl$.

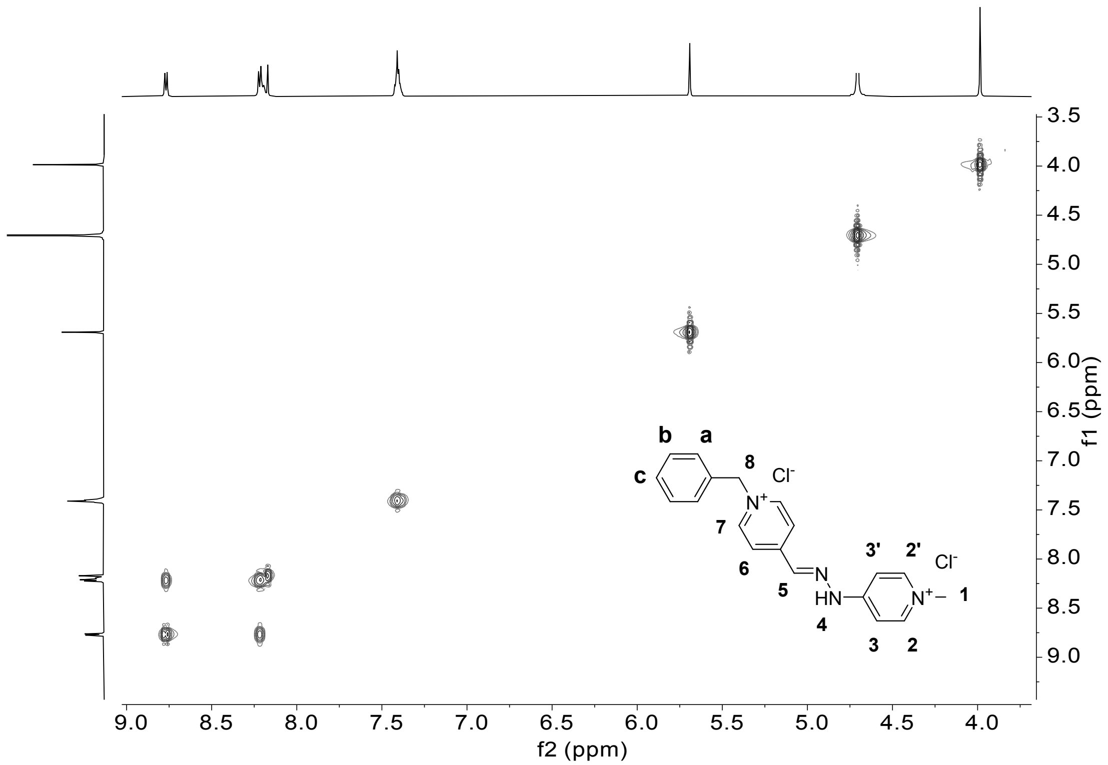

Figure S 46: COSY (500 MHz, D_2O) spectrum of $\mathbf{R_dH}\cdot 2Cl$.

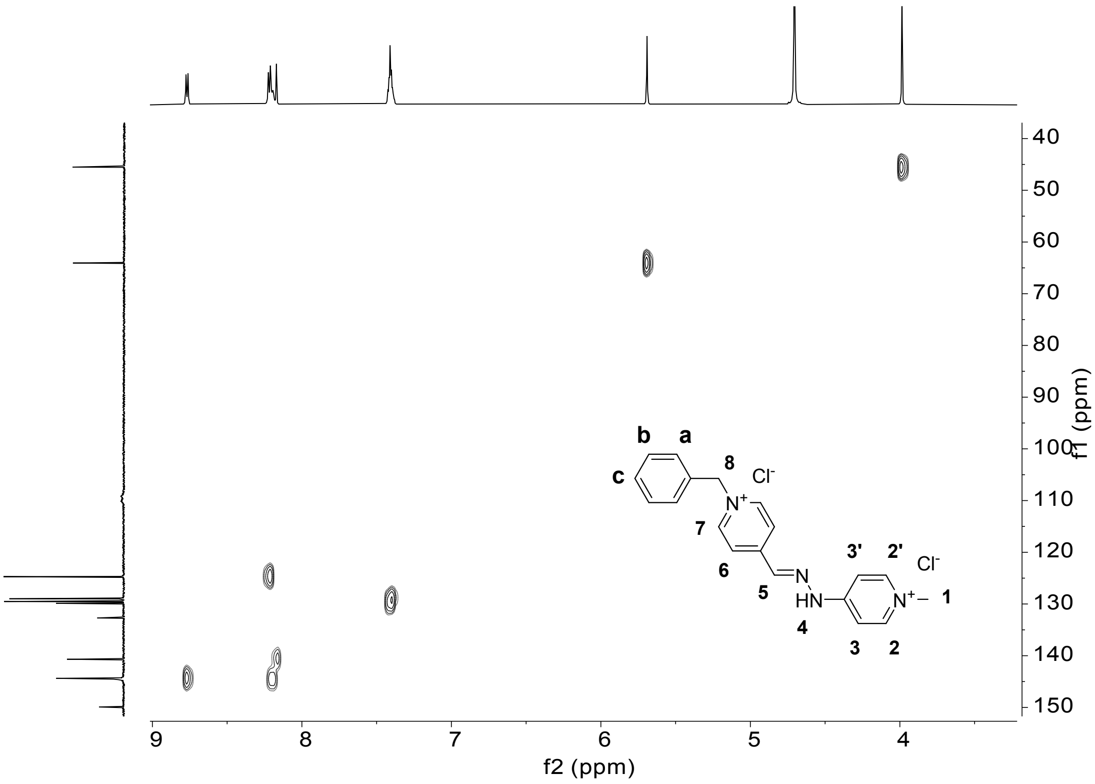

Figure S 47: HSQC (500 and 126 MHz, D_2O) spectrum of $\mathbf{R_dH}\cdot 2Cl$.

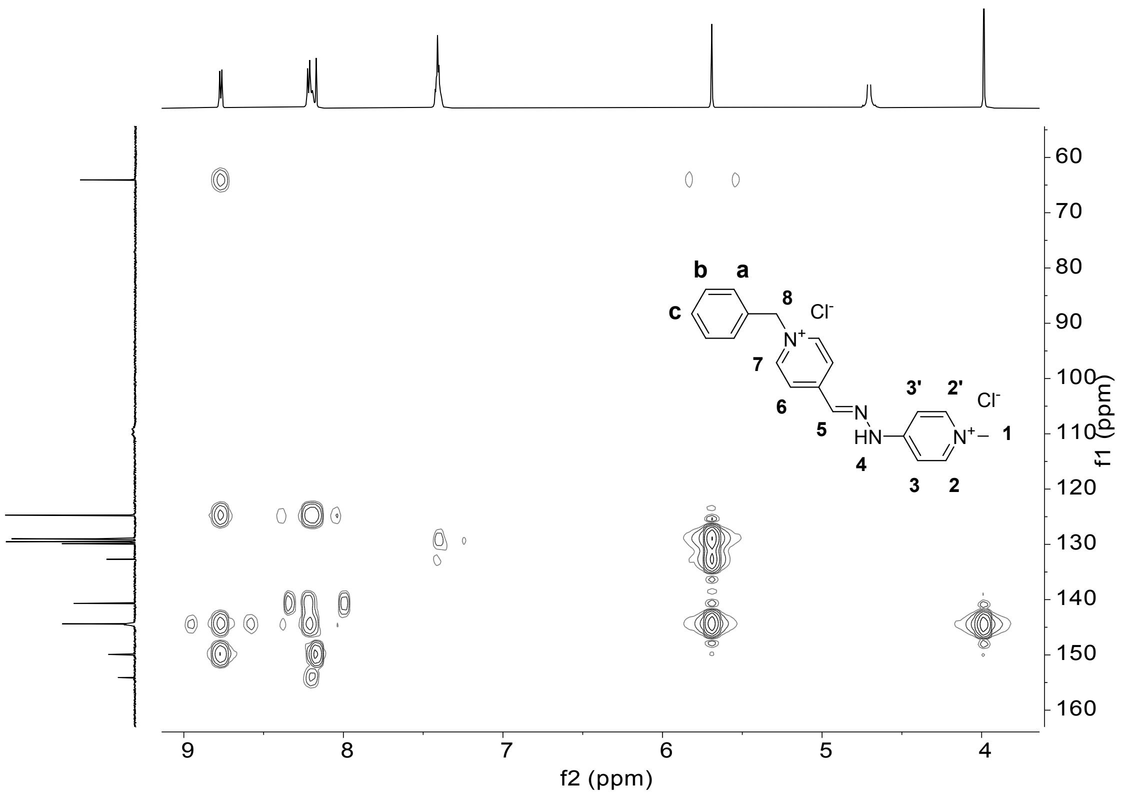

Figure S 48: HMBC (500 and 126 MHz, D_2O) spectrum of $\mathbf{R_dH}\cdot 2Cl$.

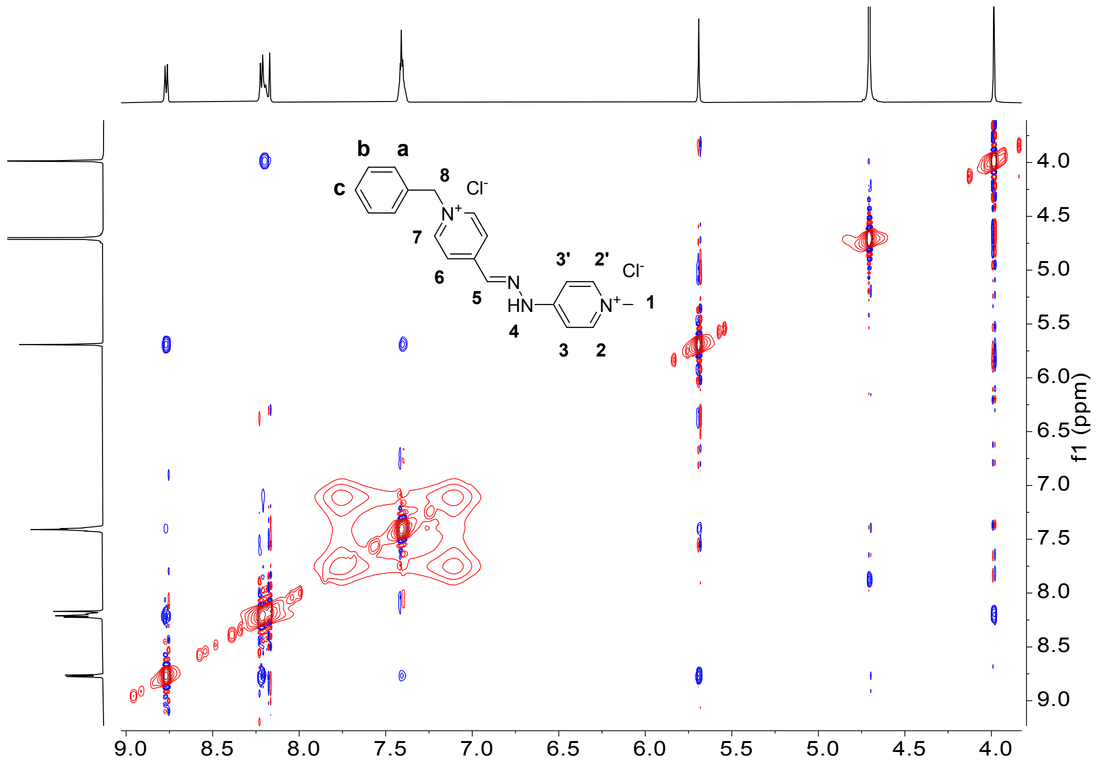

Figure S 49: NOESY (500 MHz, D_2O) spectrum of $\mathbf{R_dH}\cdot 2Cl$.

- **R_d·Cl at pD = 12:**

^{1}H NMR (300 MHz, D_2O) δ 8.59 (d, *J* = 6.6 Hz, 2H), 8.25 – 7.98 (m, 3H), 7.68 (d, *J* = 7.0 Hz, 2H), 7.58 – 7.37 (m, 5H), 6.93 (d, *J* = 165.2 Hz, 2H), 5.64 (s, 2H), 3.79 (s, 3H).

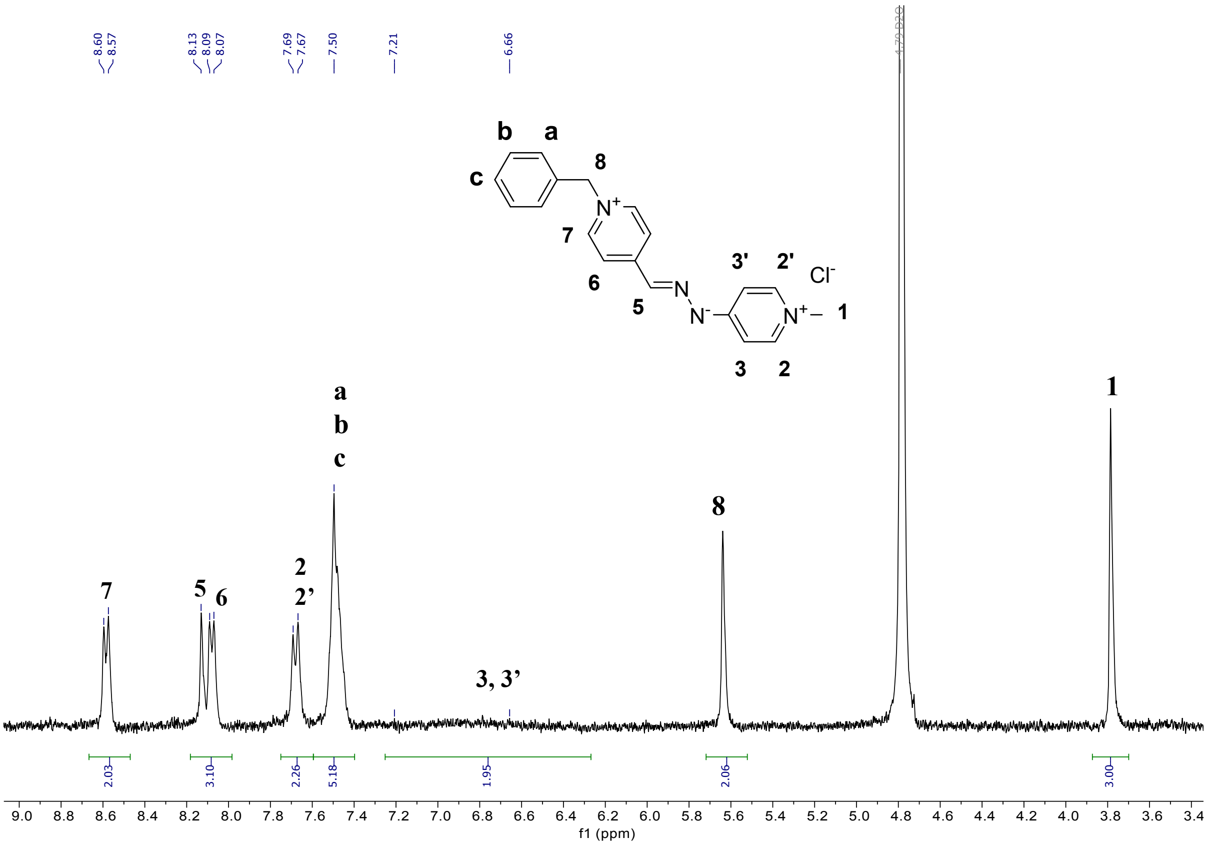

Figure S 50: ^{1}H NMR (300 MHz, D_2O) spectrum of $\mathbf{R_d}$·Cl.

- **R_dH·2PF_6:**

^{1}H NMR (500 MHz, CD_3CN) δ 10.97 (s, 1H), 8.74 (d, 2H), 8.31 (d, 2H), 8.27 (s, 1H), 8.24 (d, 3H), 7.84 (s, 1H), 7.58 – 7.45 (m, 5H), 5.72 (s, 2H), 4.07 (s, 3H) ppm. **^{13}C NMR** (126 MHz, CD_3CN) δ 153.76 (C), 149.54 (C), 144.73 (CH), 144.32 (CH), 140.30 (C=N), 132.62 (C), 129.59 (CH), 129.21 (CH), 128.80 (CH), 124.72 (CH), 110.02 (CH), 108.92 (CH), 63.78 (CH_2), 45.57 (CH_3) ppm.

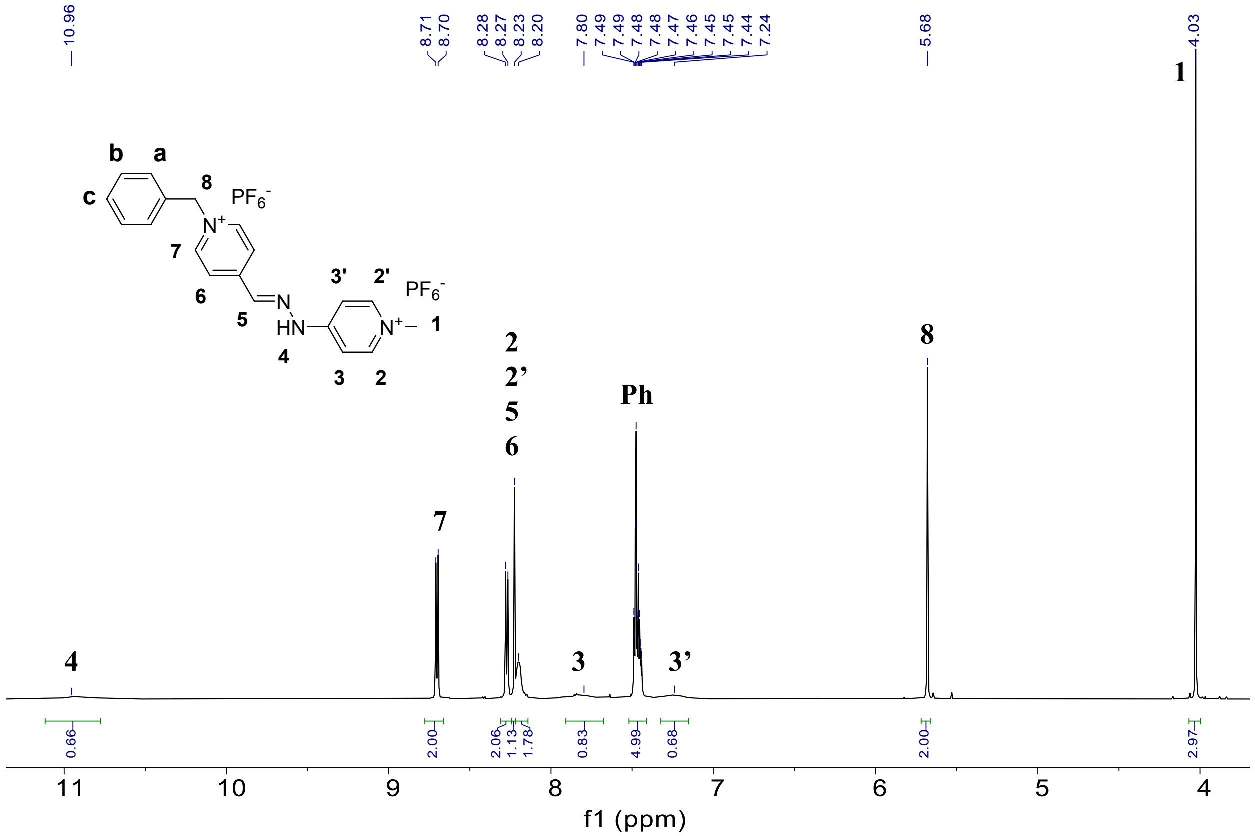

Figure S 51: ^{1}H NMR (500 MHz, CD_3CN) spectrum of **R_dH**·2PF_6.

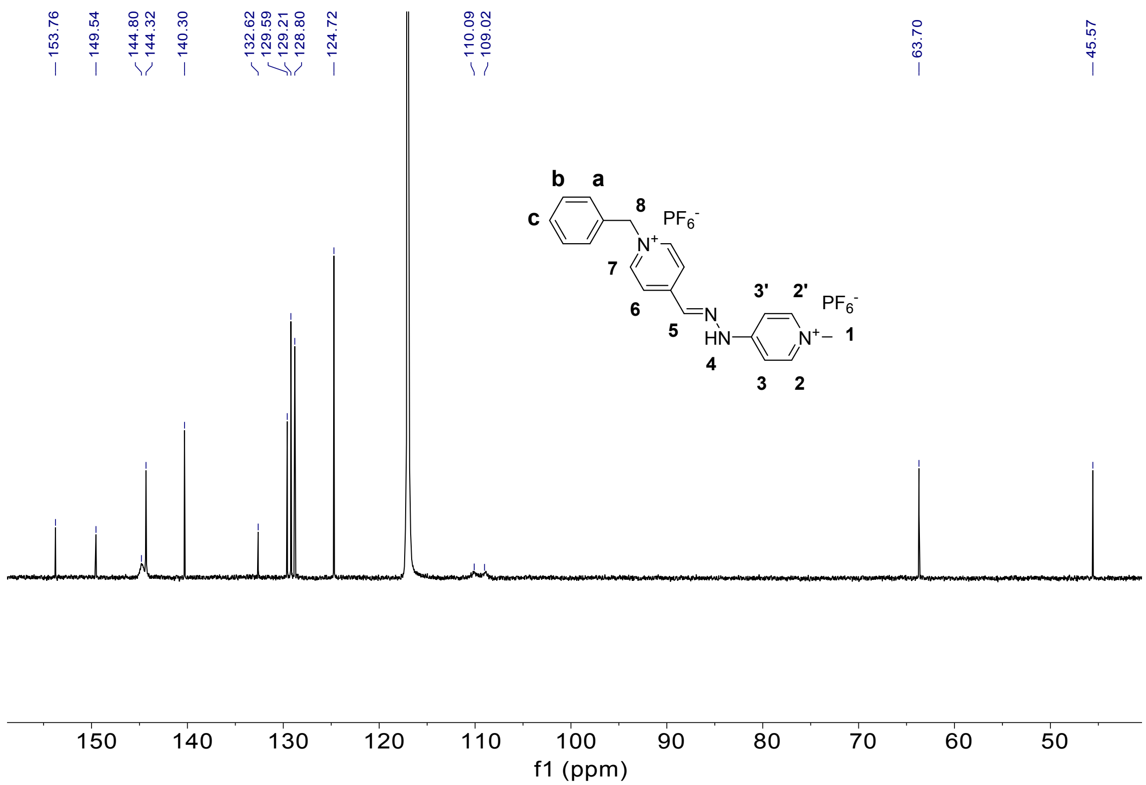

Figure S 52: ^{13}C NMR (126 MHz, CD_3CN) spectrum of $\mathbf{R_dH}\cdot 2PF_6$.

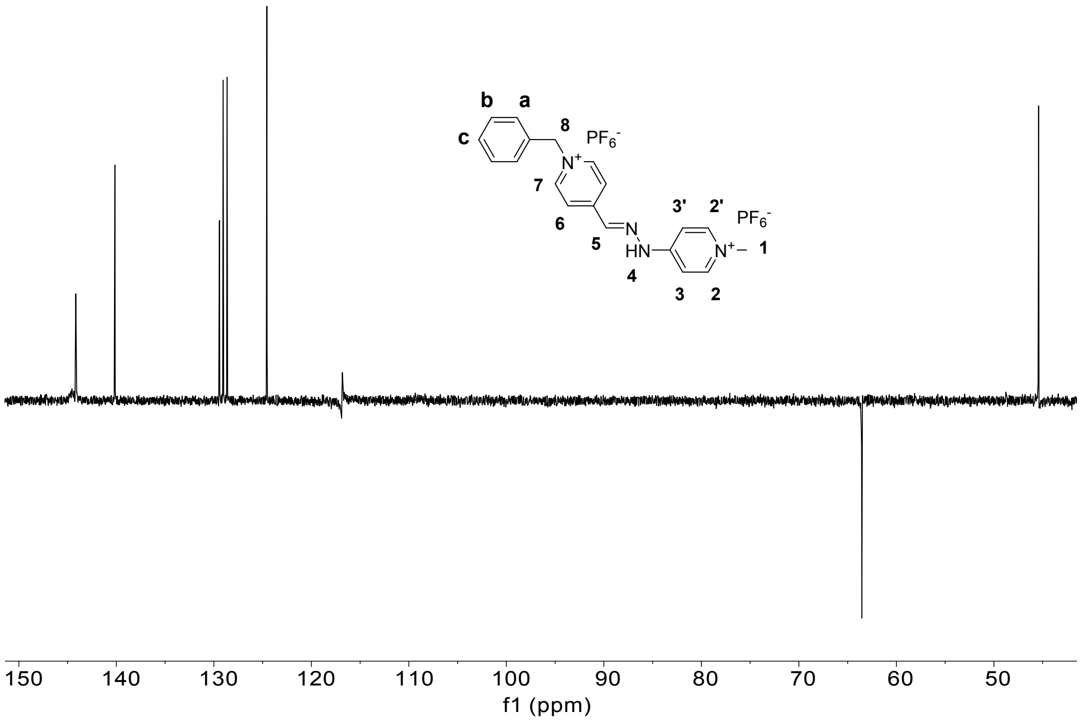

Figure S 53: DEPT-135 (126 MHz, CD_3CN) spectrum of $\mathbf{R_dH}\cdot 2PF_6$.

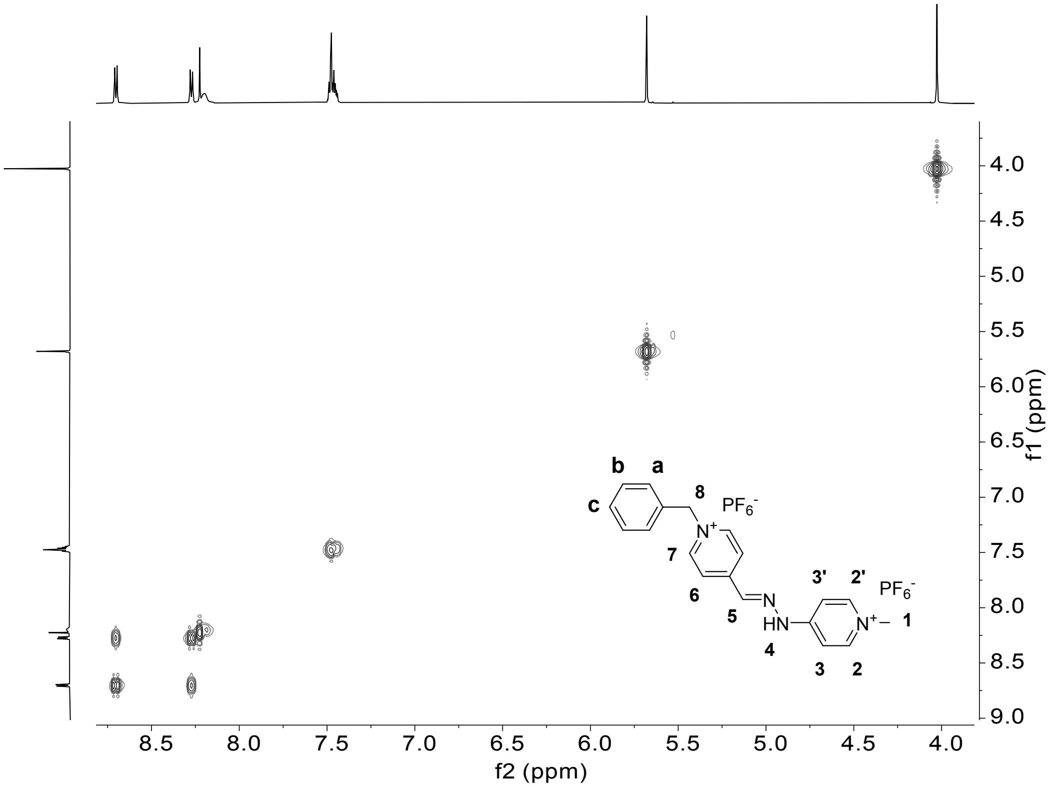

Figure S 54: COSY (500 MHz, CD_3CN) spectrum of $\mathbf{R_dH}\cdot 2PF_6$.

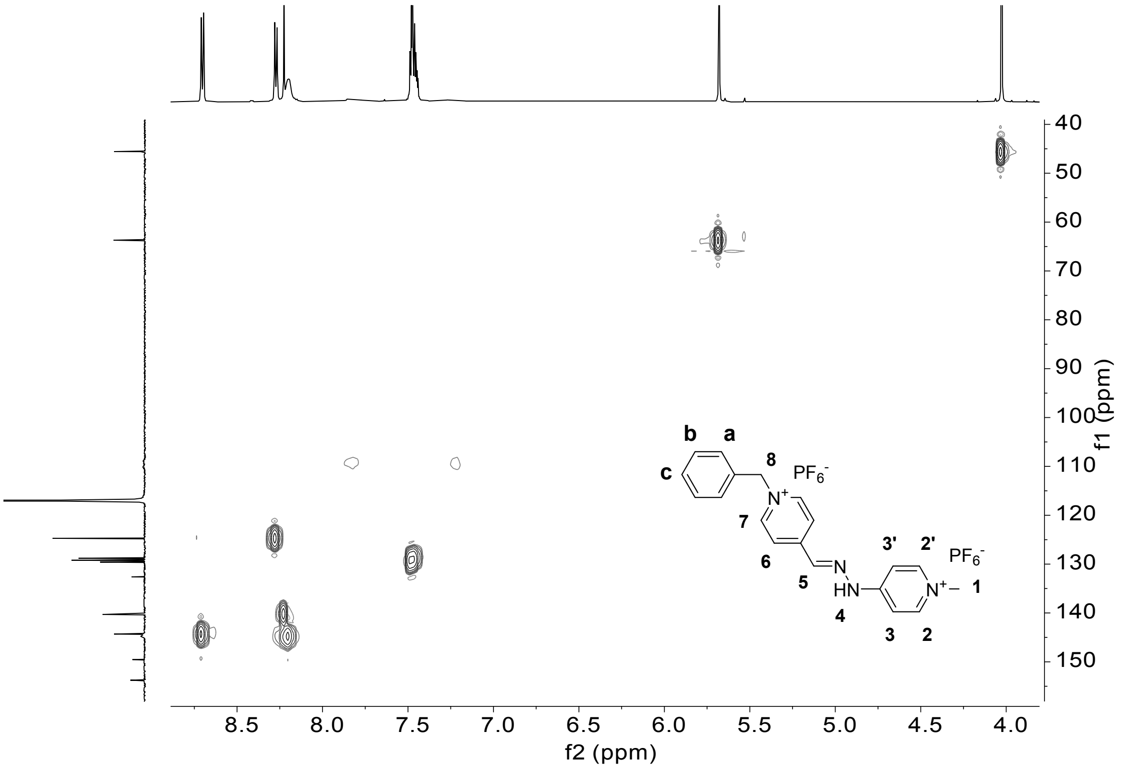

Figure S 55: HSQC (500 and 126 MHz, CD_3CN) spectrum of $\mathbf{R_dH}\cdot 2PF_6$.

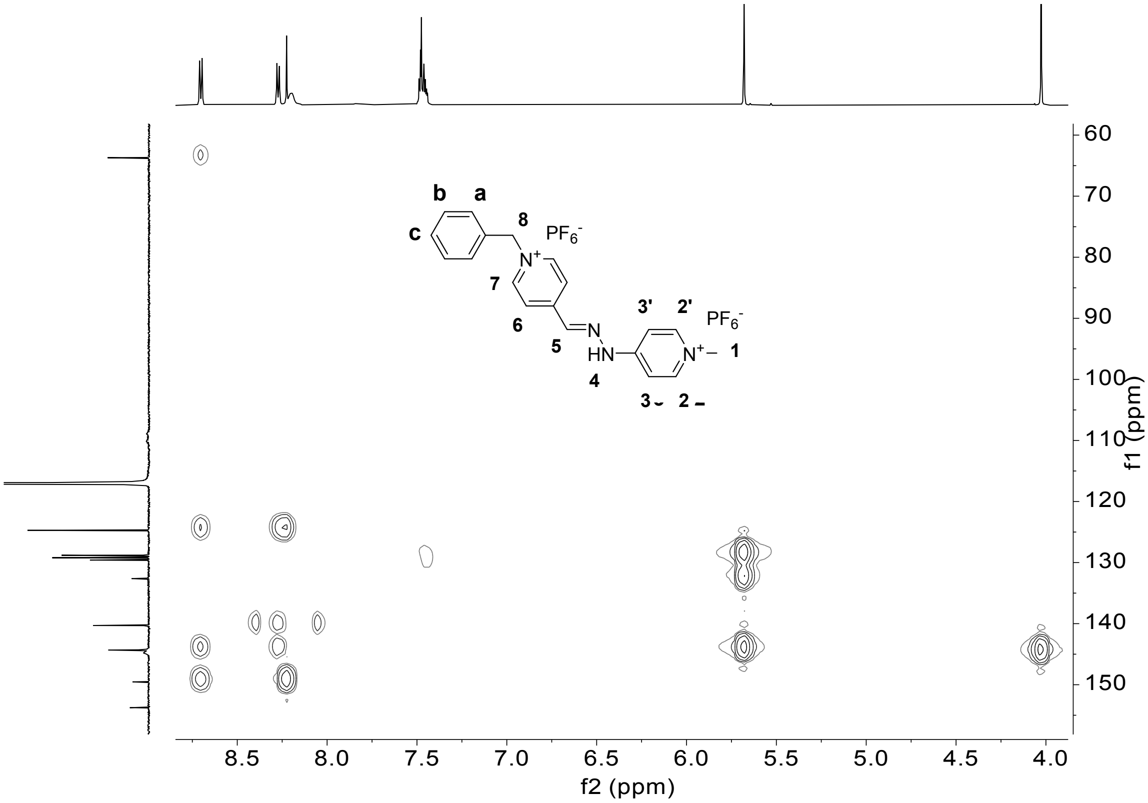

Figure S 56: HMBC (500 and 126 MHz, CD_3CN) spectrum of $\mathbf{R_dH}\cdot 2PF_6$.

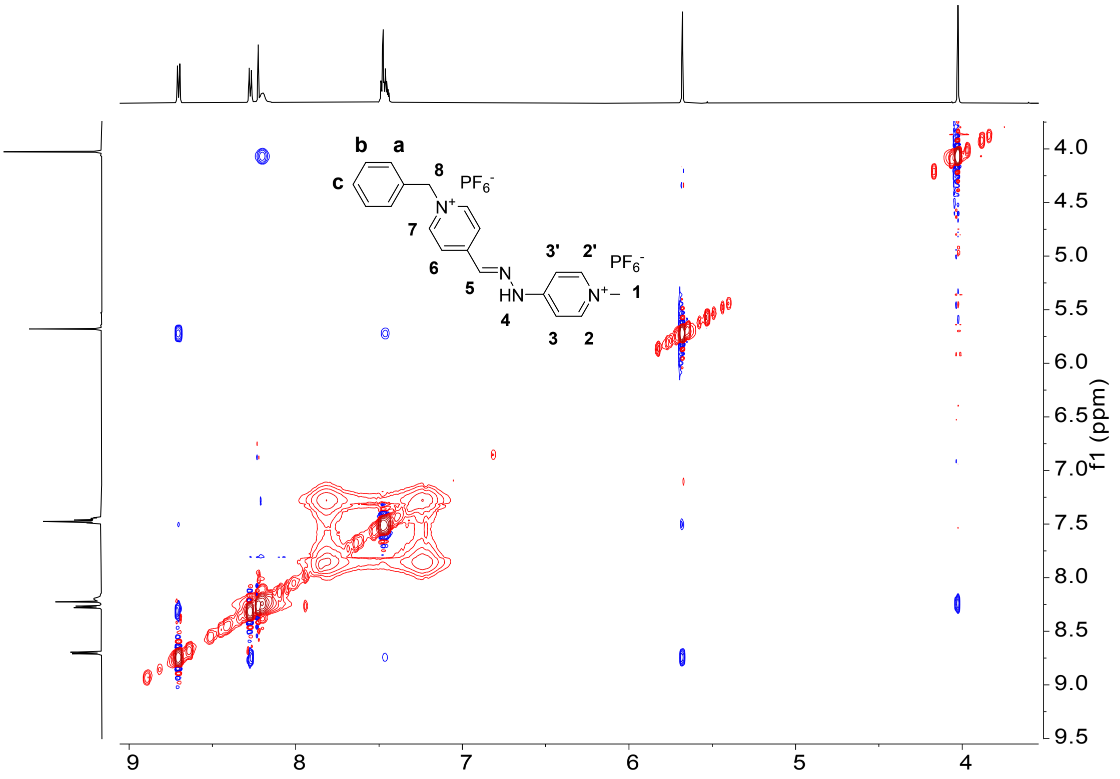

Figure S 57: NOESY (500 MHz, CD_3CN) spectrum of $\mathbf{R_dH}\cdot 2PF_6$.

2.7. Synthesis and characterization data of $\mathbf{R_eH{\cdot}2Cl}$

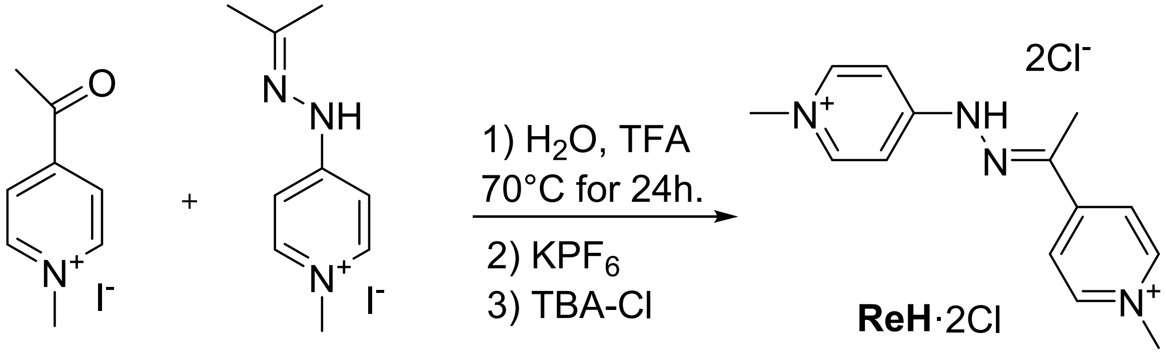

Same synthetic procedure described for $\mathbf{R_bH^{2+}}$.

$\mathbf{R_eH}{\cdot}2PF_6$: orange powder (0.8 g, 74 %). $\mathbf{R_eH}{\cdot}2Cl$: yellowish solid (0.44 g, 96 %).

^{1}H NMR (500 MHz, D_2O) δ 8.79 (d, J = 6.4 Hz, 2H), 8.45 (d, J = 6.3 Hz, 2H), 8.33 (d, J = 7.1 Hz, 2H), 7.82 (s, 1H), 4.40 (s, 3H), 4.12 (s, 3H), 2.50 (t, J = 1.7 Hz, 3H) ppm. **^{13}C NMR** (126 MHz, D_2O) δ 154.66 (C), 152.75 (C), 146.73 (C=N), 144.84 (CH), 144.43 (CH), 124.11 (CH), 47.45 (CH_3), 45.47 (CH_3), 12.55 (CH_3) ppm. **HRMS (ESI)** (*m/z*): calcd for $[C_{14}H_{18}N_4\text{-}H]^+$ 241.1448, found 241.1449.

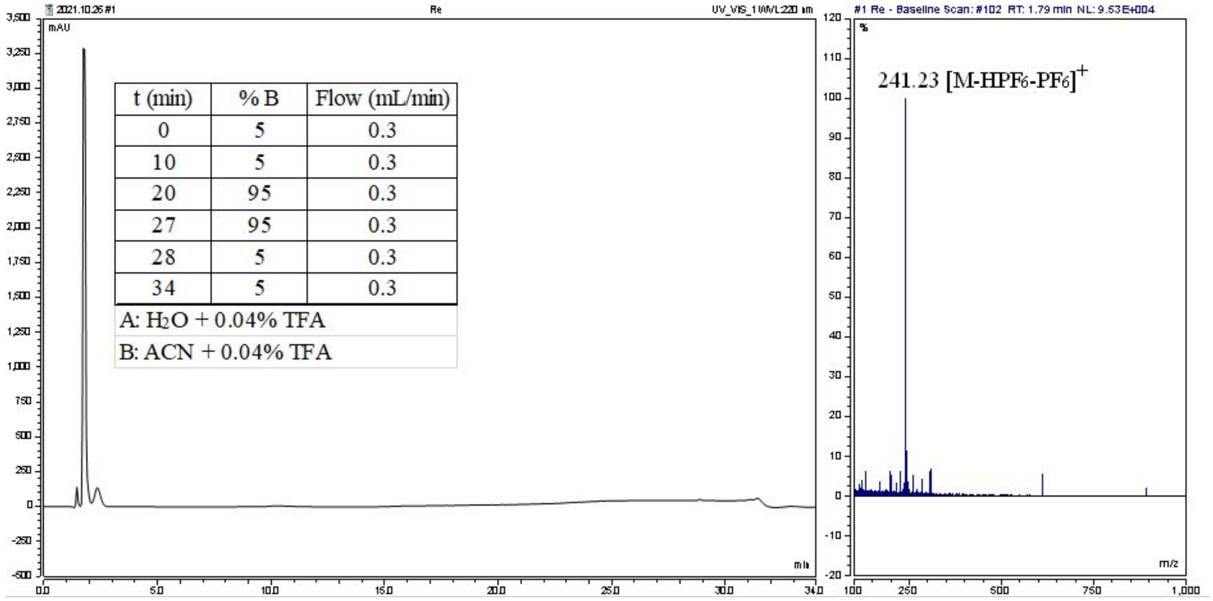

Figure S 58. HPLC-MS chromatogram of $\mathbf{R_eH}{\cdot}2PF_6$. *Inset.* Table of the elution conditions.

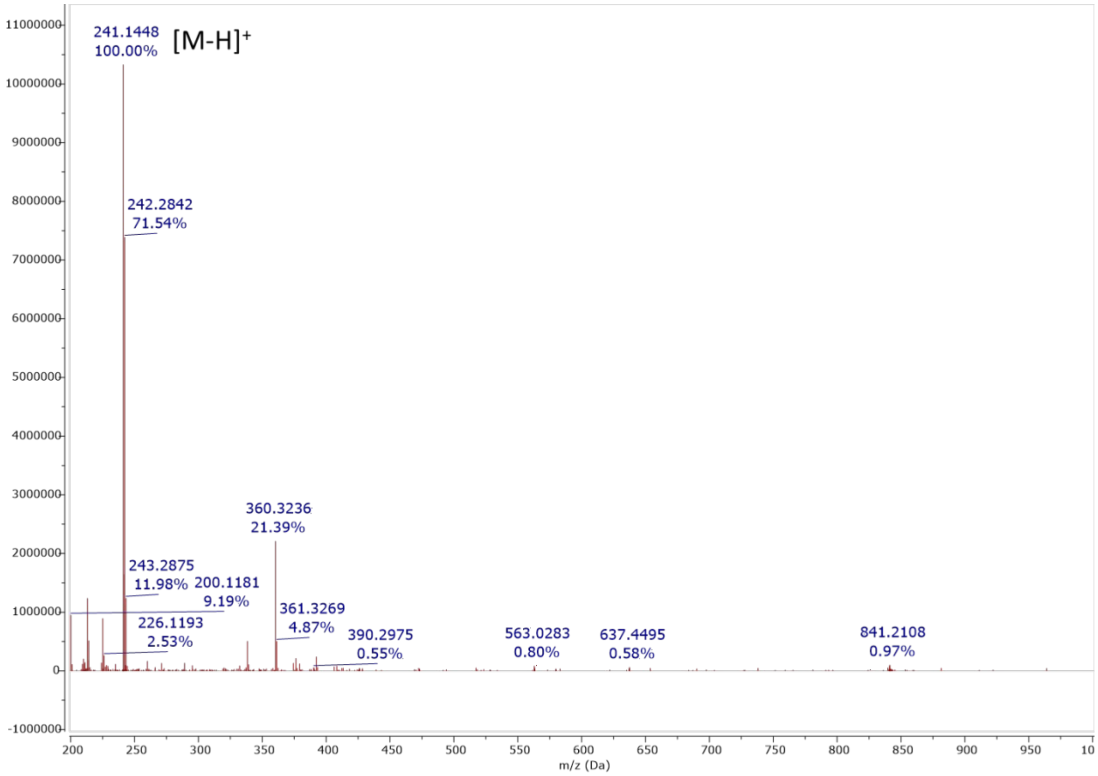

Figure S 59. HR ESI-MS spectrum of $\mathbf{R_cH}\cdot 2PF_6$.

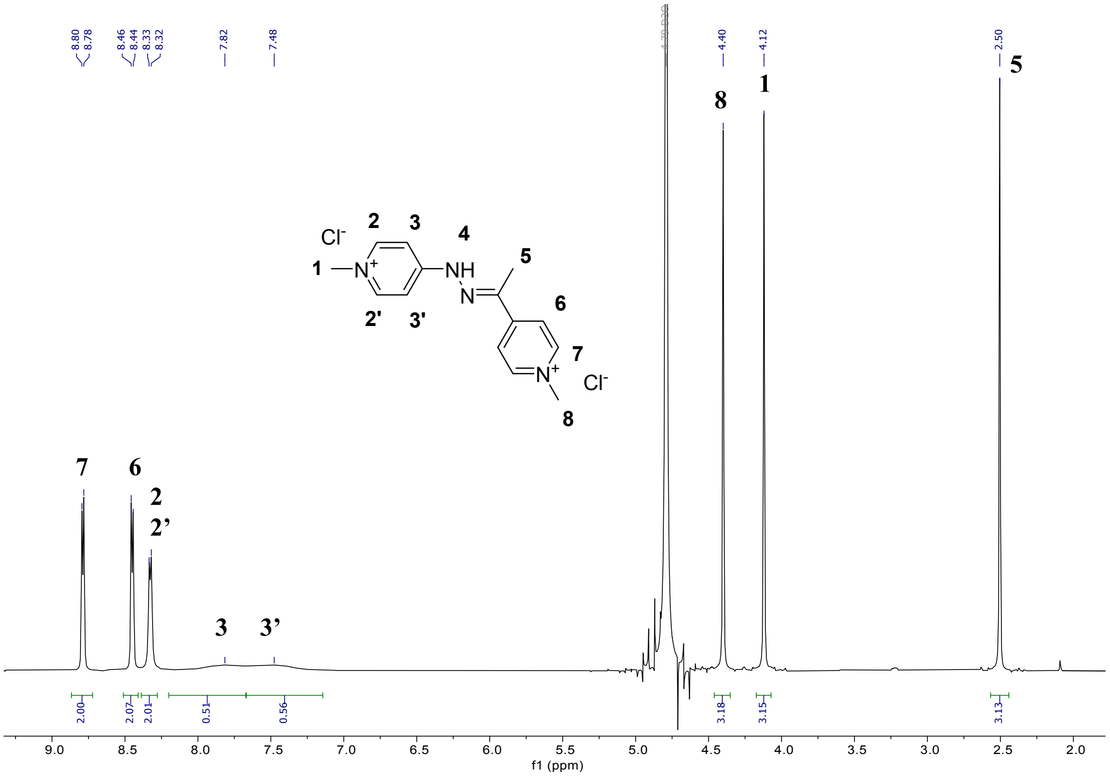

Figure S 60: ^{1}H NMR (500 MHz, D_2O) spectrum of $\mathbf{R_cH}\cdot 2Cl$.

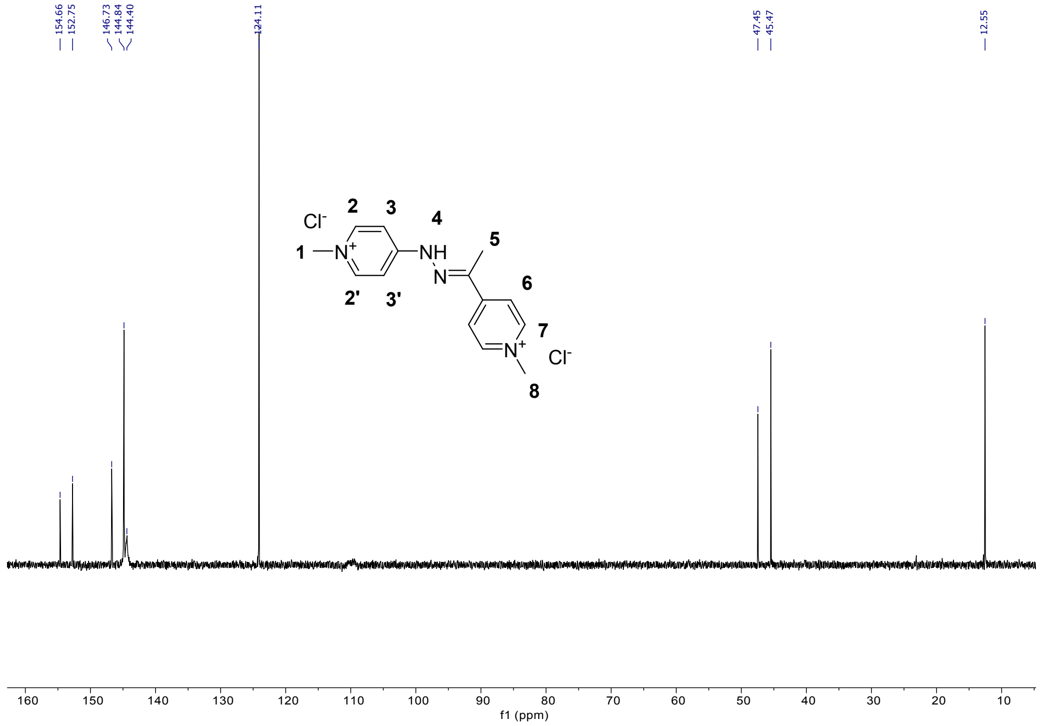

Figure S 61: ^{13}C NMR (126 MHz, D_2O) spectrum of $\mathbf{R_eH}$·2Cl.

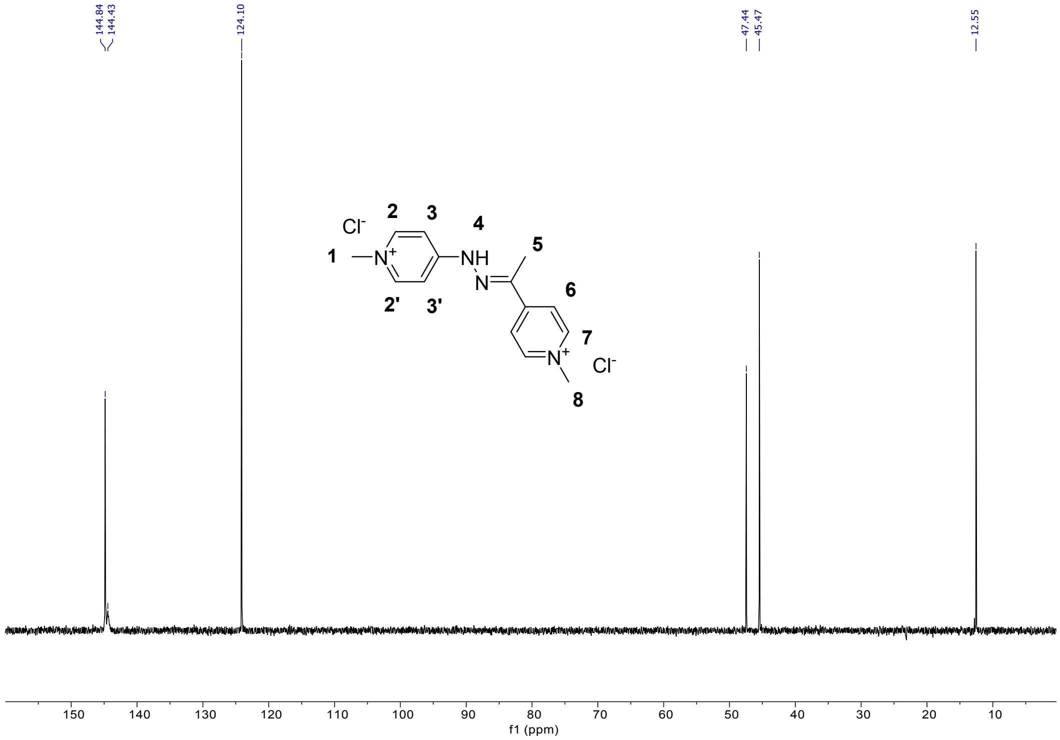

Figure S 62: DEPT-135 (126 MHz, D2O) spectrum of $\mathbf{R_eH}$·2Cl.

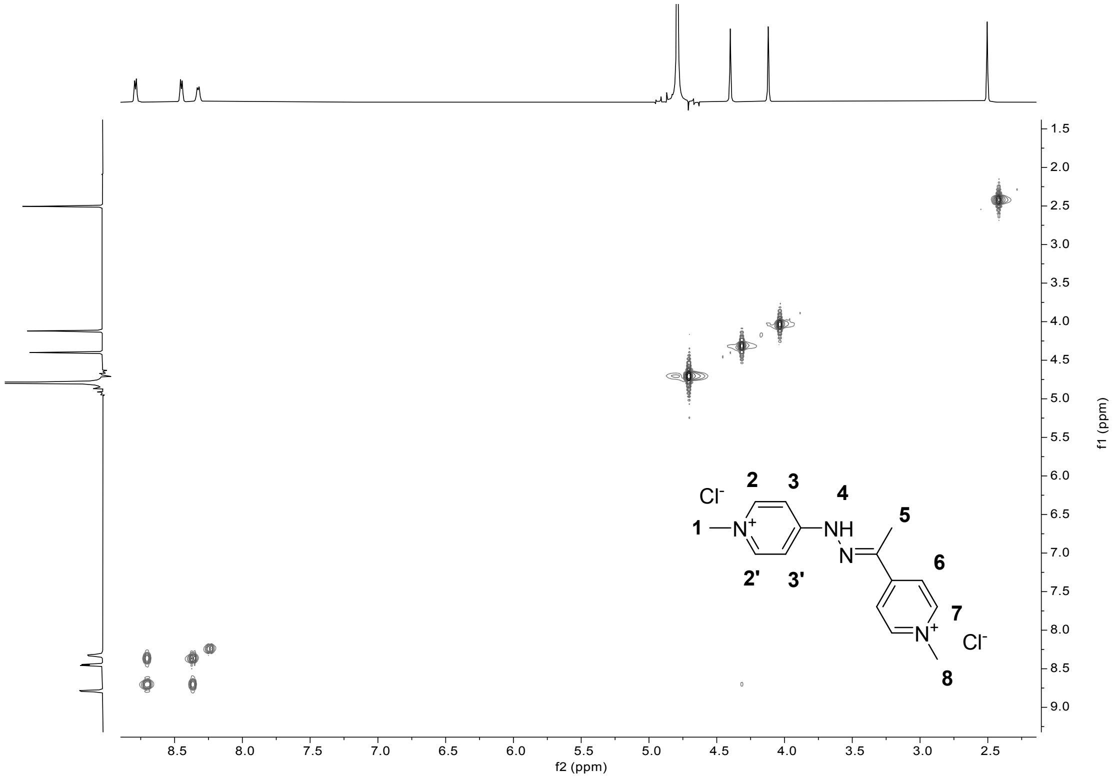

Figure S 63: COSY (500 MHz, D_2O) spectrum of $\mathbf{R_eH}$·2Cl.

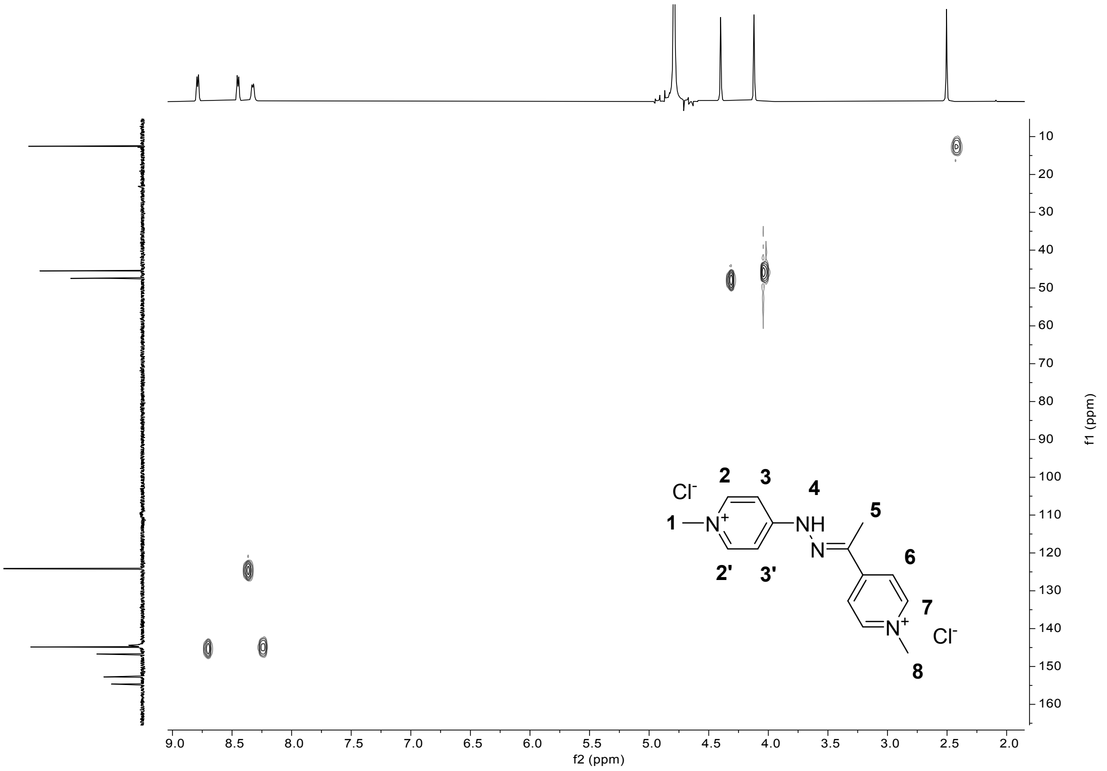

Figure S 64: HSQC (500 and 126 MHz, D_2O) spectrum of $\mathbf{R_eH}$·2Cl.

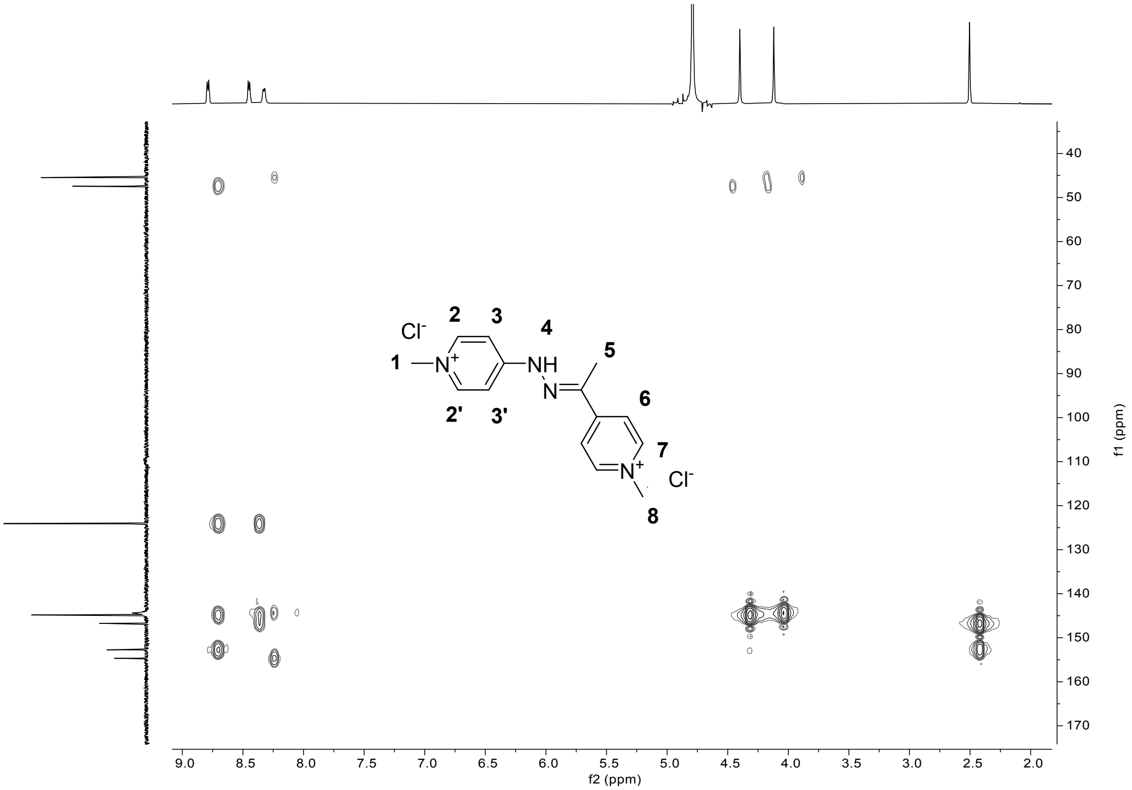

Figure S 65: HMBC (500 and 126 MHz, D_2O) spectrum of $\mathbf{R_eH}$·2Cl.

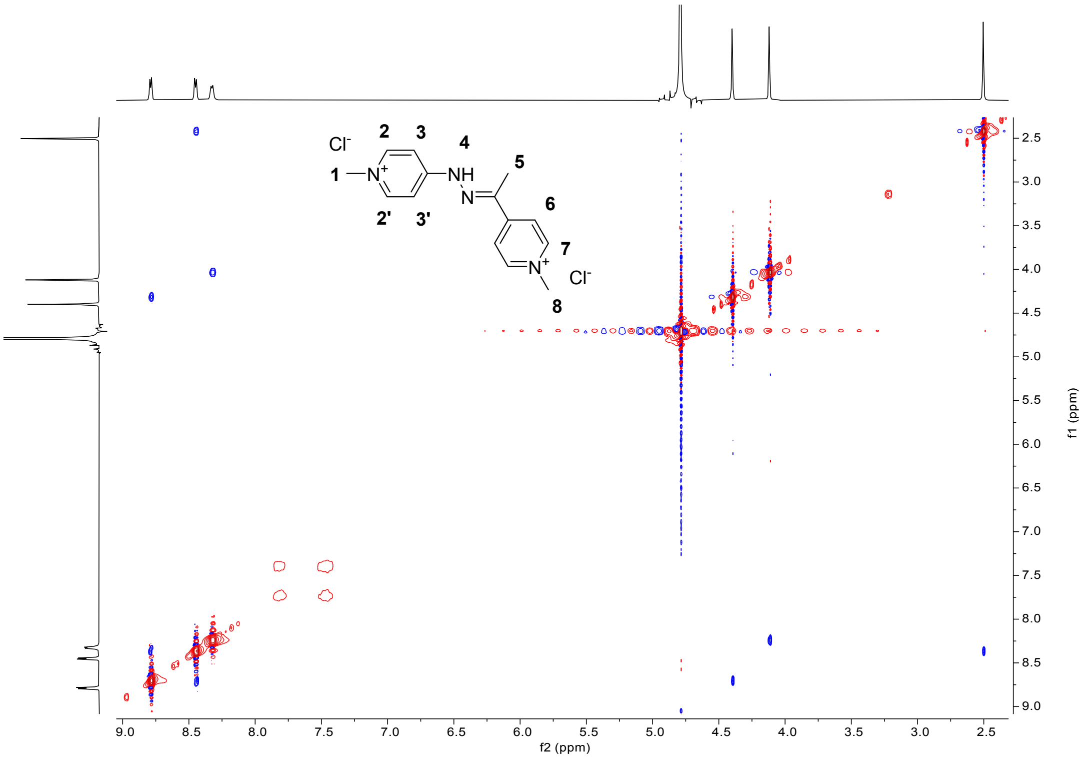

Figure S 66: NOESY (500 MHz, D_2O) spectrum of $\mathbf{R_eH}$·2Cl.

- **R_e·Cl at pD = 12**.

^{1}H NMR (300 MHz, D_2O) δ 8.55 (d, *J* = 6.8 Hz, 2H), 8.28 (d, *J* = 6.8 Hz, 2H), 7.67 (d, *J* = 7.4 Hz, 2H), 7.43 (s, 1H), 6.82 (s, 1H), 4.27 (s, 3H), 3.78 (s, 3H), 2.38 (s, 3H).

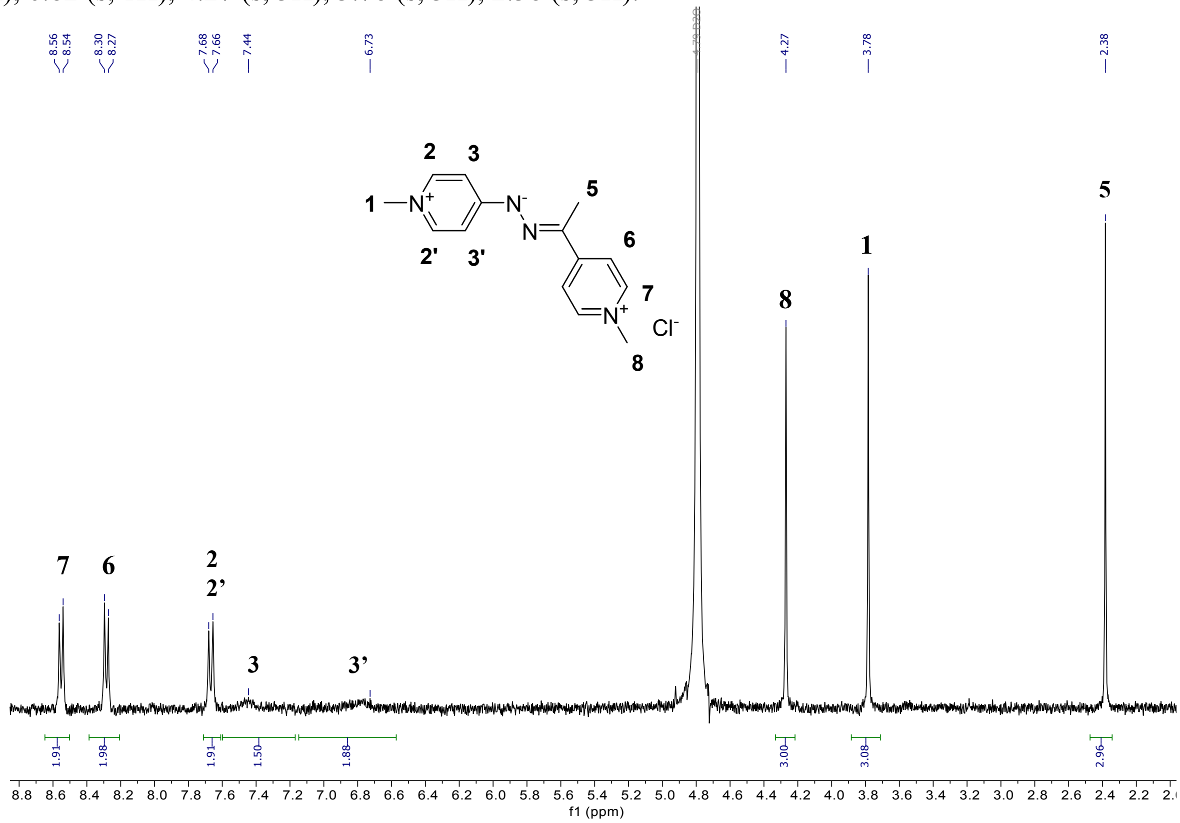

Figure S 67: ^{1}H NMR (300 MHz, D_2O) spectrum of **R_e**·Cl.

- **R$_e$H·2PF$_6$:**

^{1}H NMR (500 MHz, CD_3CN) δ 10.13 (s, 1H), 8.61 (d, 2H), 8.36 (d, 2H), 8.21 (s, 2H), 7.62 (d, *J* = 232.6 Hz, 2H), 4.28 (s, 3H), 4.04 (s, 3H), 2.41 (s, 3H) ppm. **^{13}C NMR** (126 MHz, CD_3CN) δ 154.37 (C), 152.22 (C), 146.27 (C=N), 144.90 (CH) 144.39 (CH), 123.91 (CH), 47.43 (CH_3), 45.51 (CH_3), 12.53 (CH_3) ppm.

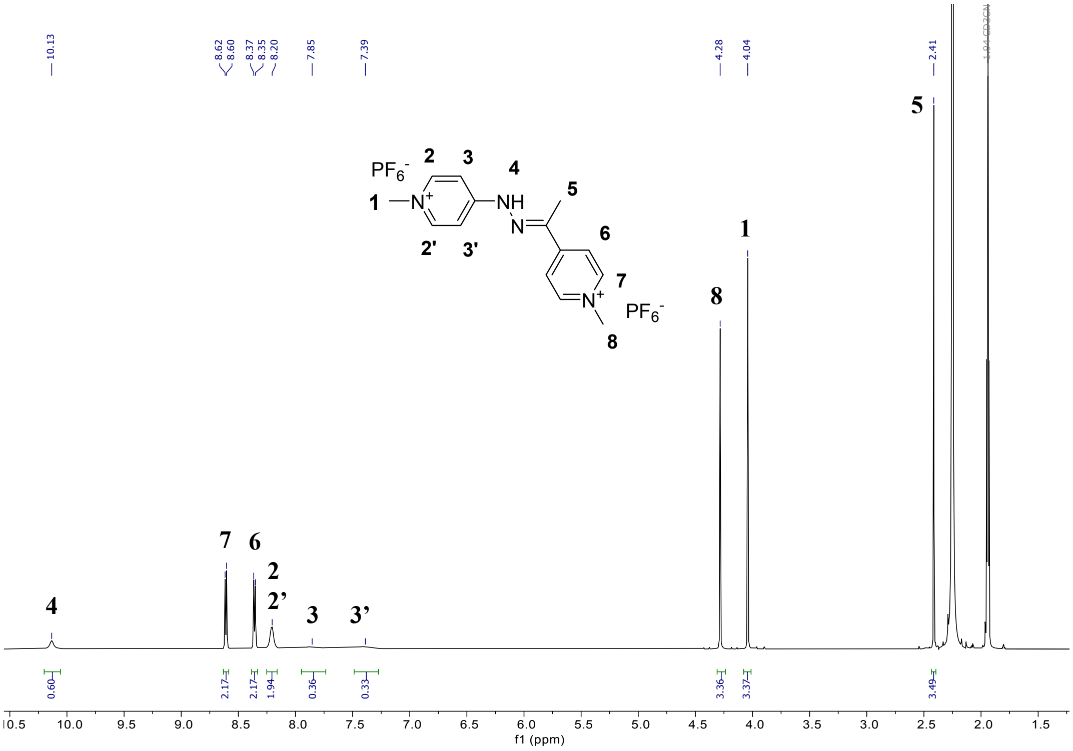

Figure S 68: ^{1}H NMR (500 MHz, CD_3CN) spectrum of **R$_e$H**·2PF$_6$.

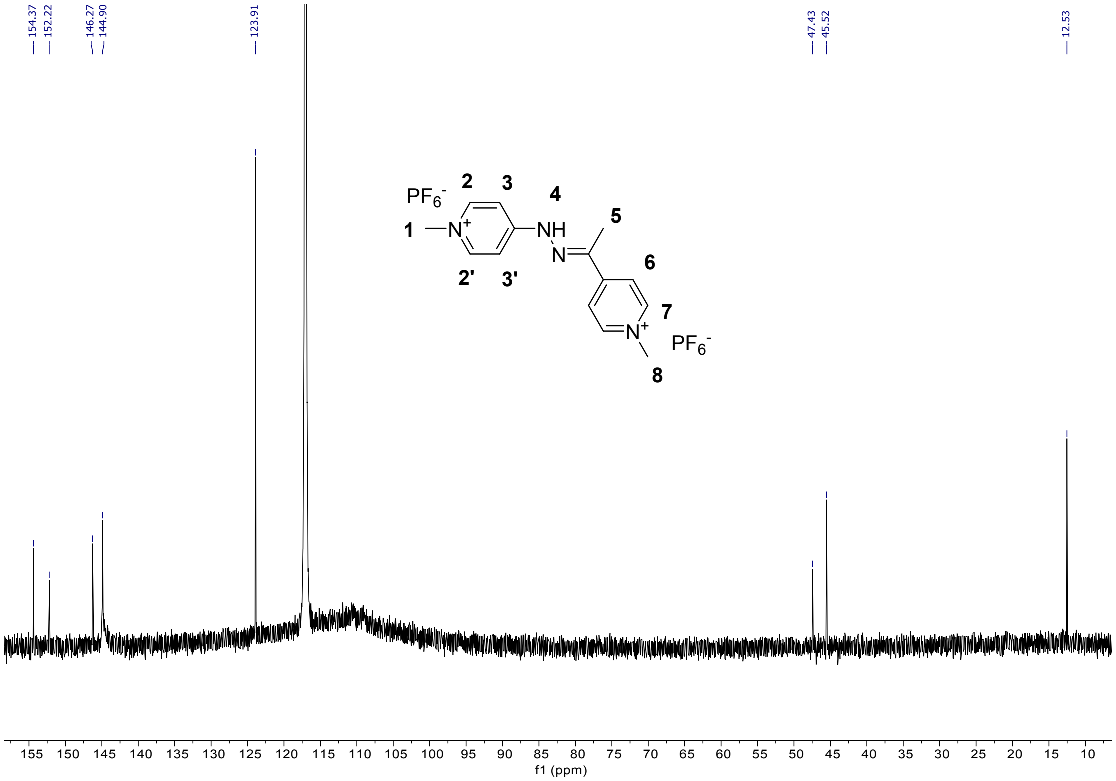

Figure S 69: ^{13}C NMR (126 MHz, CD_3CN) spectrum of **R$_e$H**·2PF$_6$.

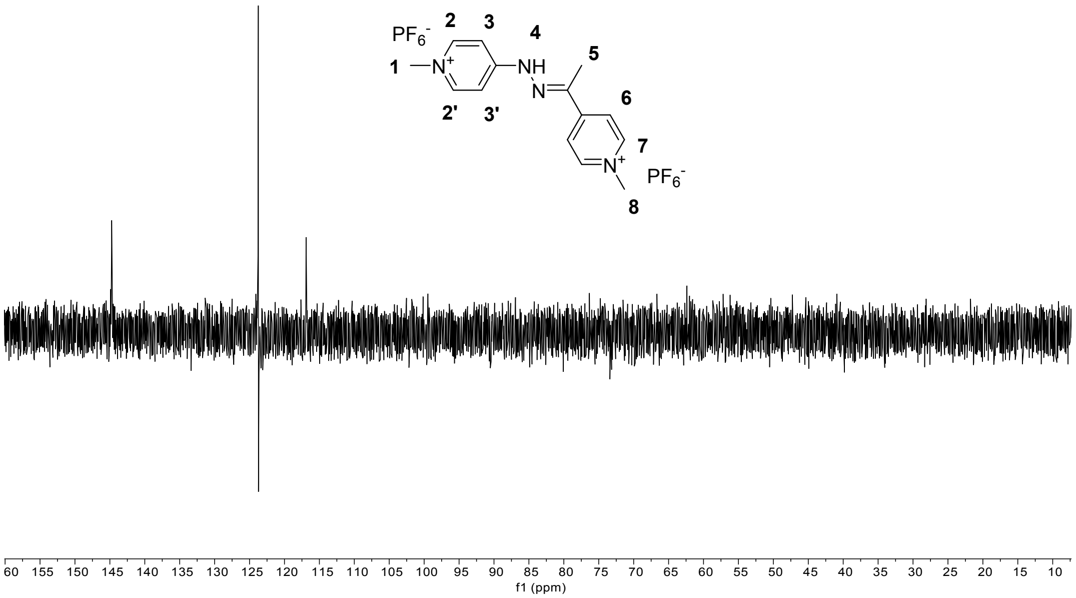

Figure S 70: DEPT-135 (126 MHz, CD3CN) spectrum of **R$_e$H**·2PF$_6$.

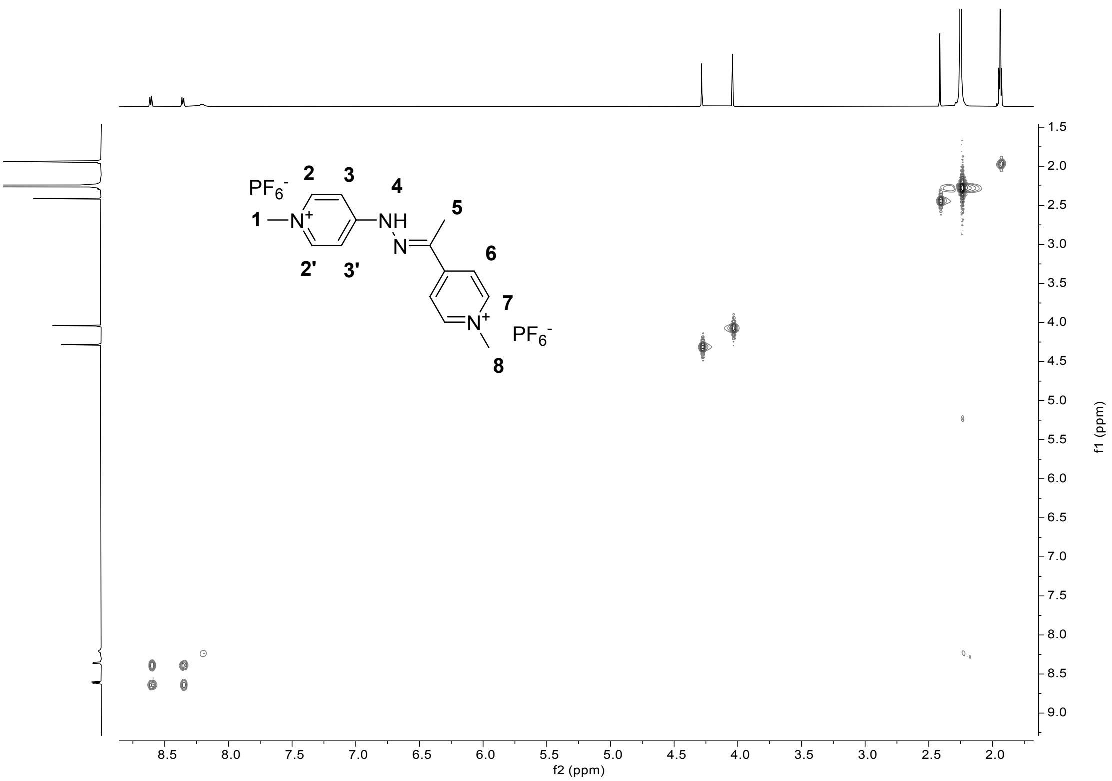

Figure S 71: COSY (500 MHz, CD3CN) spectrum of $\mathbf{R_eH}\cdot 2PF_6$.

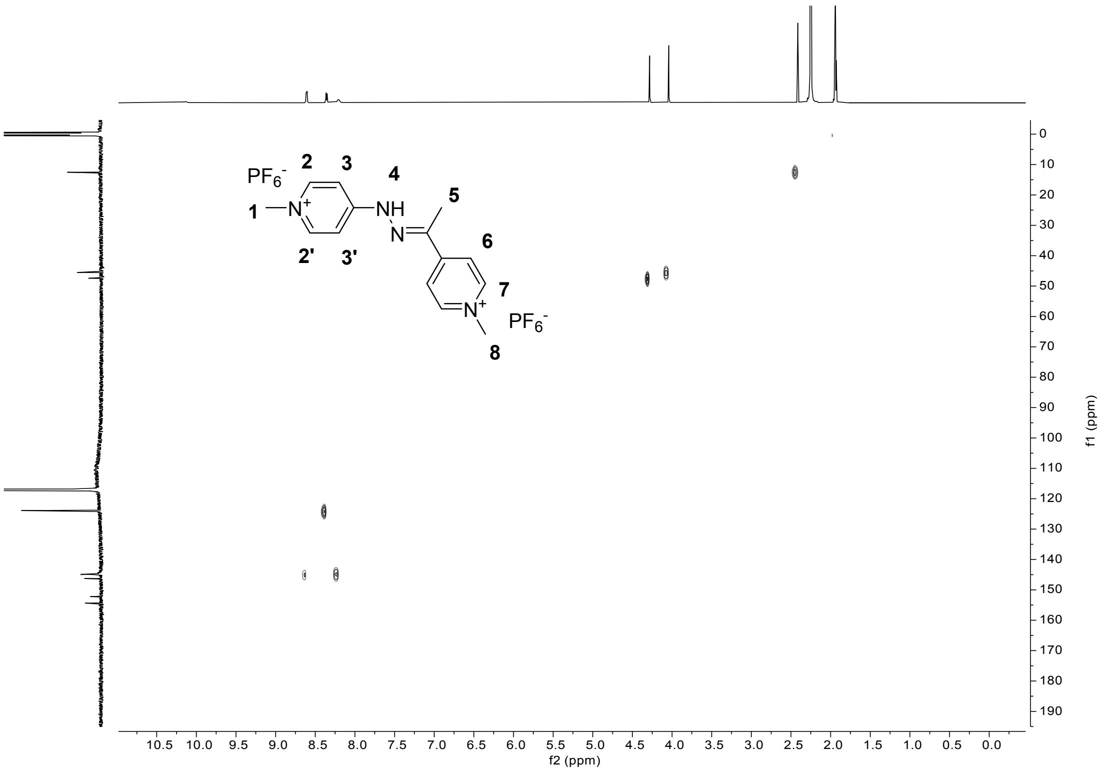

Figure S 72: HSQC (500 and 126 MHz, CD_3CN) spectrum of $\mathbf{R_eH}\cdot 2PF_6$.

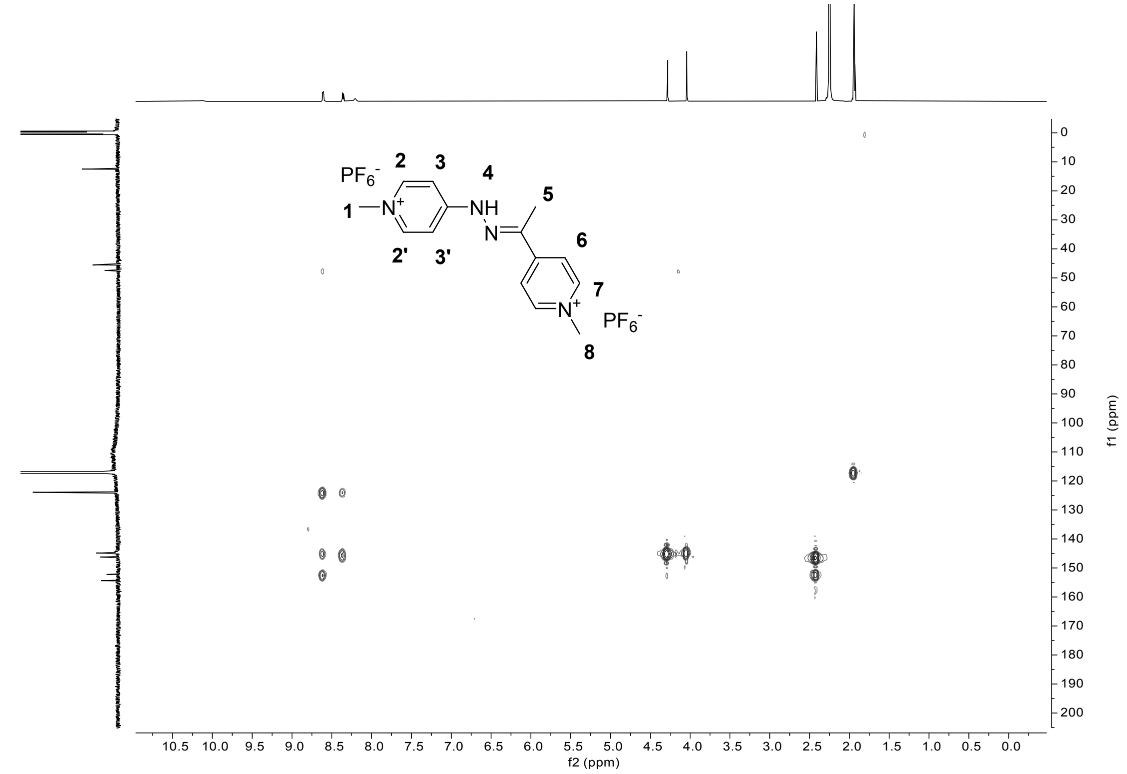

Figure S 73: HMBC (500 and 126 MHz, CD_3CN) spectrum of $\mathbf{R_eH}\cdot 2PF_6$.

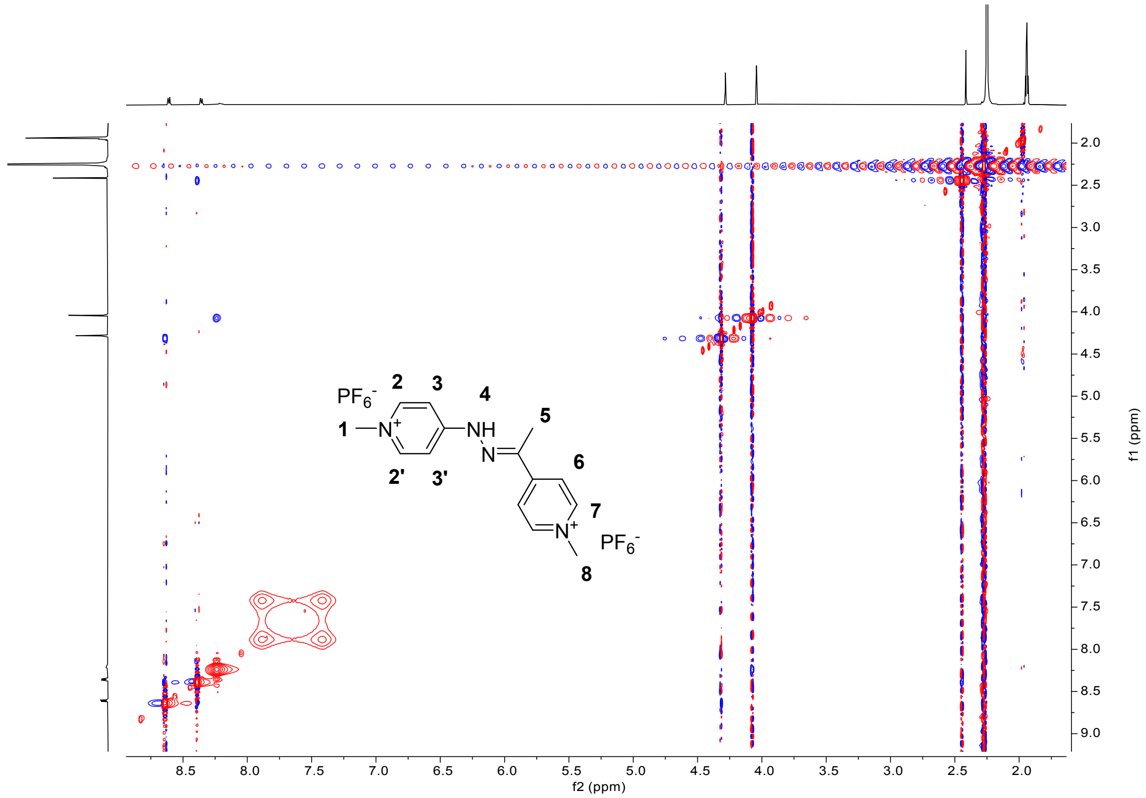

Figure S 74: NOESY (500 MHz, CD_3CN) spectrum of $\mathbf{R_eH}\cdot 2PF_6$.

2.8. Synthesis and characterization data of M_aH

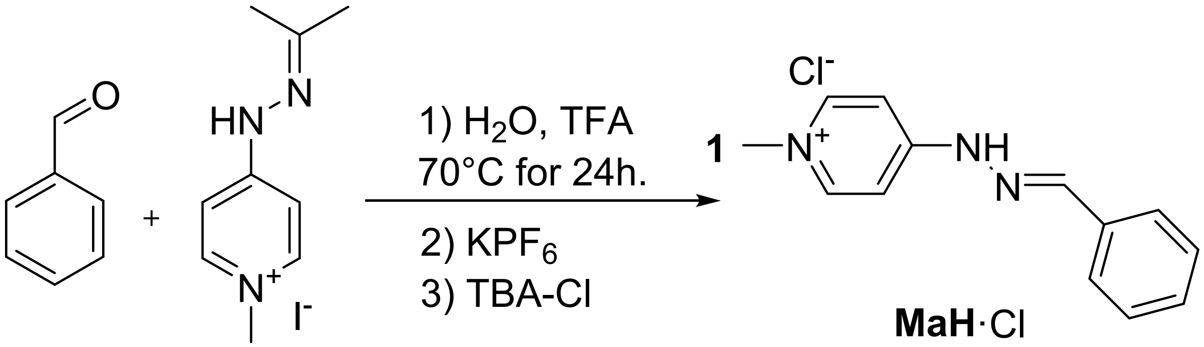

Same synthetic procedure described for $\mathbf{R_bH^{2+}}$.

$\mathbf{M_aH}\cdot PF_6$: orange powder (620 mg, 86%). $\mathbf{M_aH}\cdot$Cl: yellow solid (360 mg, 72%).

^{1}H NMR (400 MHz, D_2O) δ 8.08 – 7.95 (m, 1H), 7.87 (s, 1H), 7.73 – 7.64 (m, 2H), 7.56 – 7.42 (m, 3H), 6.79 (s, 1H), 3.85 (s, 3H) ppm. **^{13}C NMR** (101 MHz, D_2O) δ 153.58 (C), 148.29 (C=N), 144.00 (CH), 143.00 (CH), 133.22 (C), 130.92 (CH), 129.00 (CH), 127.32 (CH), 108.63 (CH), 107.28 (CH), 44.80 (CH_3) ppm. **HRMS (ESI)** (*m/z*): calcd for $[C_{13}H_{14}N_3]^+$ 212.1183, found 212.1182.

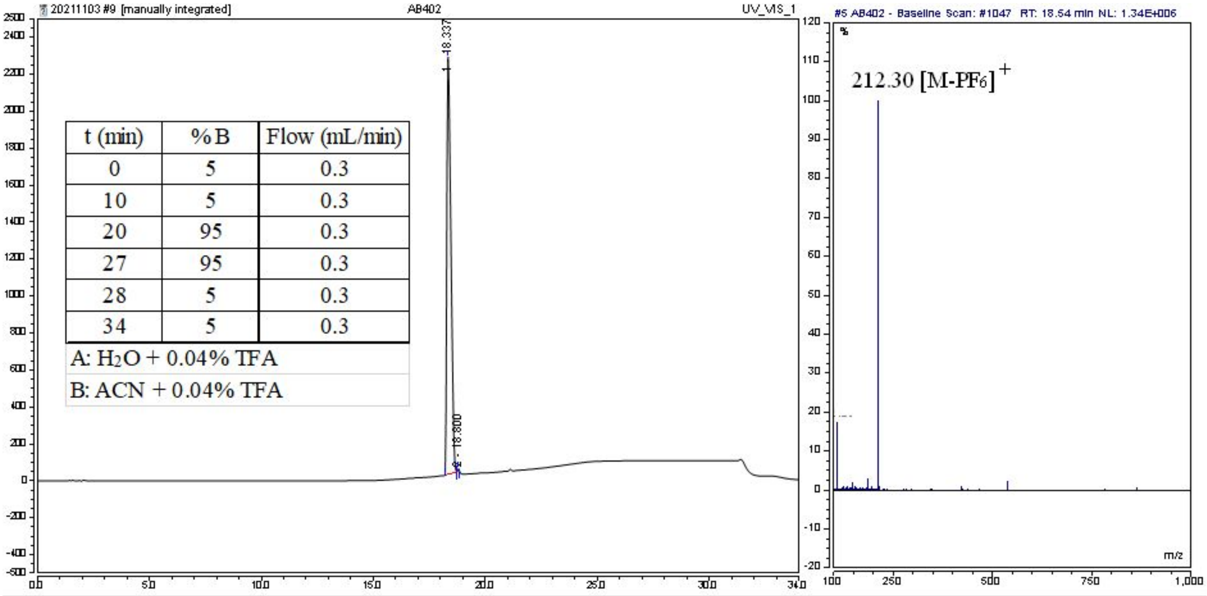

t (min)	%B	Flow (mL/min)
0	5	0.3
10	5	0.3
20	95	0.3
27	95	0.3
28	5	0.3
34	5	0.3
A: H_2O + 0.04% TFA		
B: ACN + 0.04% TFA		

Figure S 75. HPLC-MS chromatogram of $\mathbf{M_aH}\cdot PF_6$. *Inset.* Table of the elution conditions.

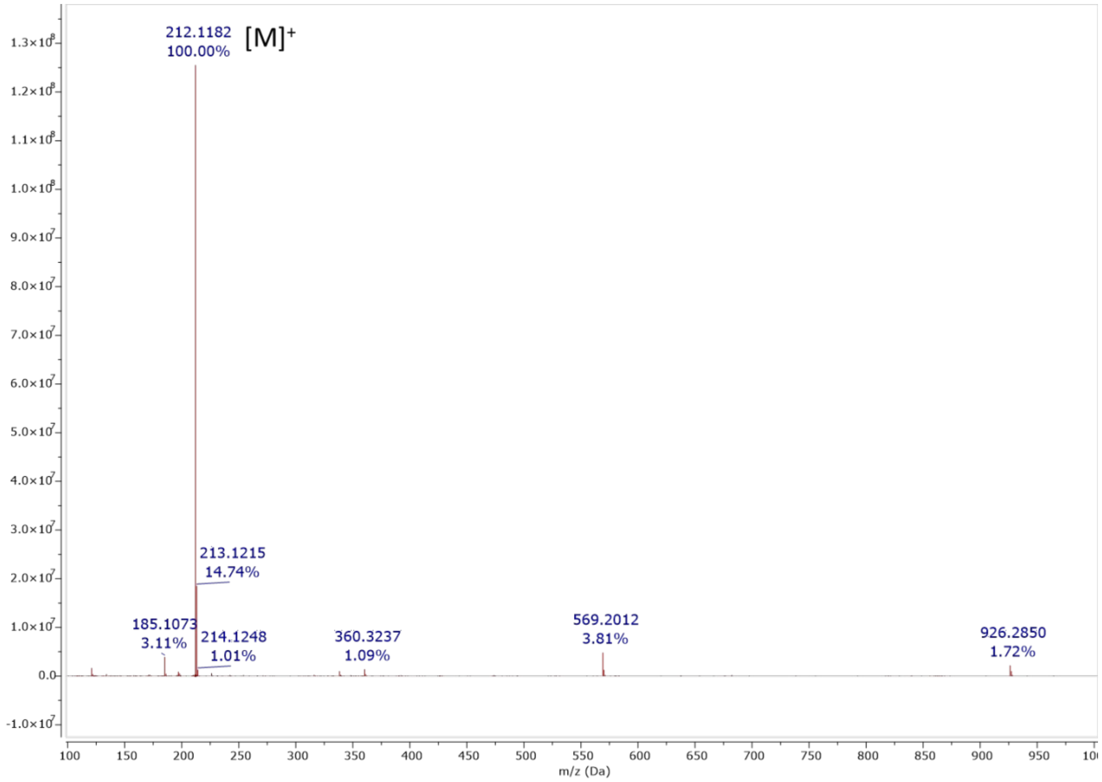

Figure S 76. HR ESI-MS spectrum of $\mathbf{M_a}H \cdot 2PF_6$.

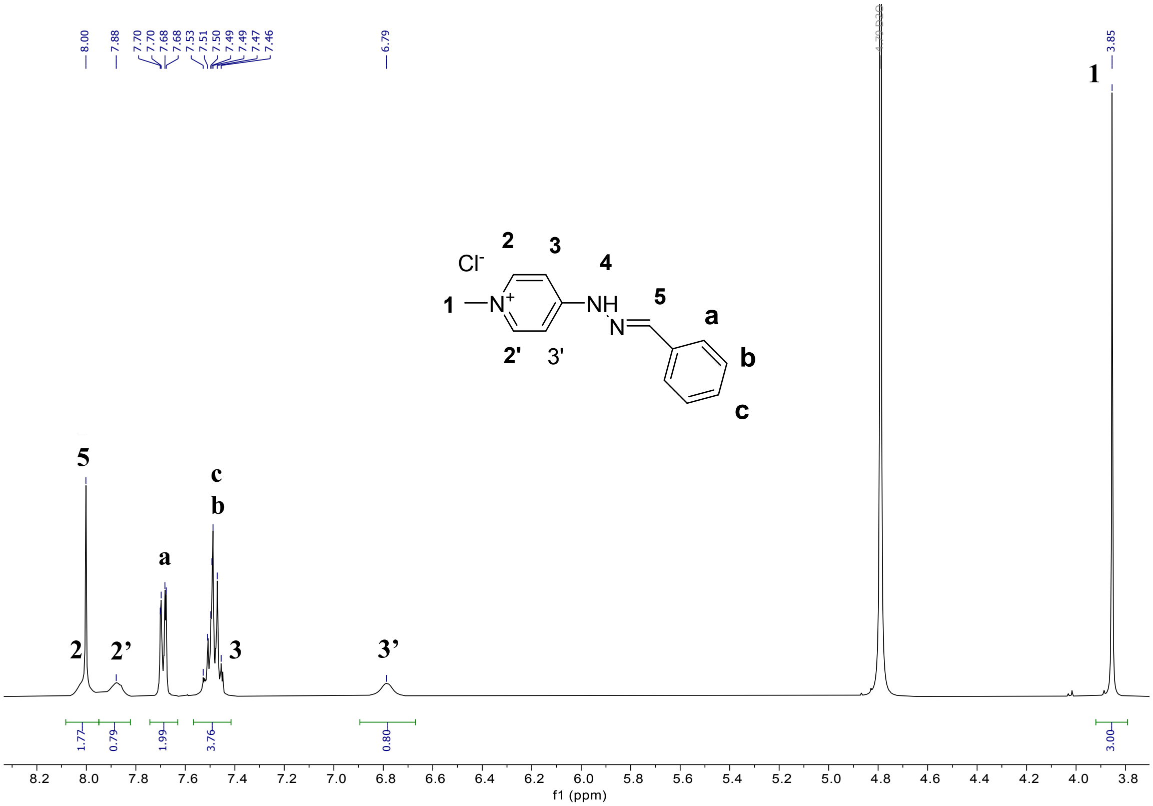

Figure S 77: ^{1}H NMR (400 MHz, D_2O) spectrum of $\mathbf{M_aH}$·Cl.

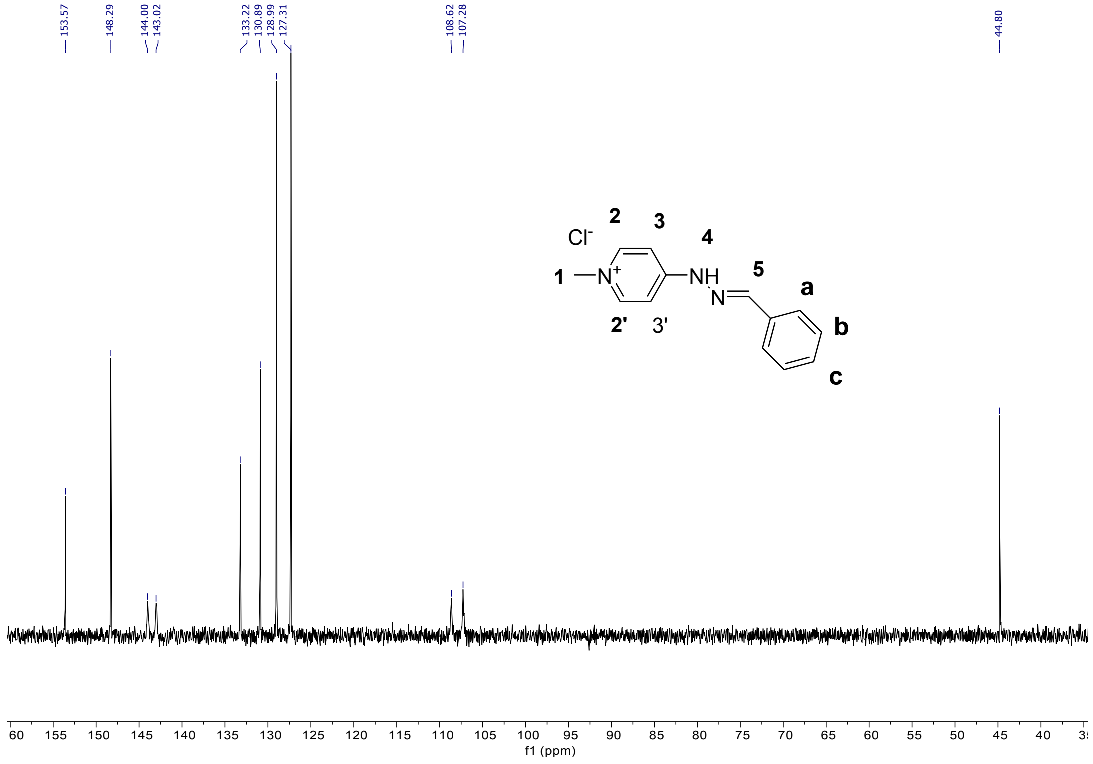

Figure S 78: ^{13}C NMR (101 MHz, D_2O) spectrum of $\mathbf{M_aH}$·Cl.

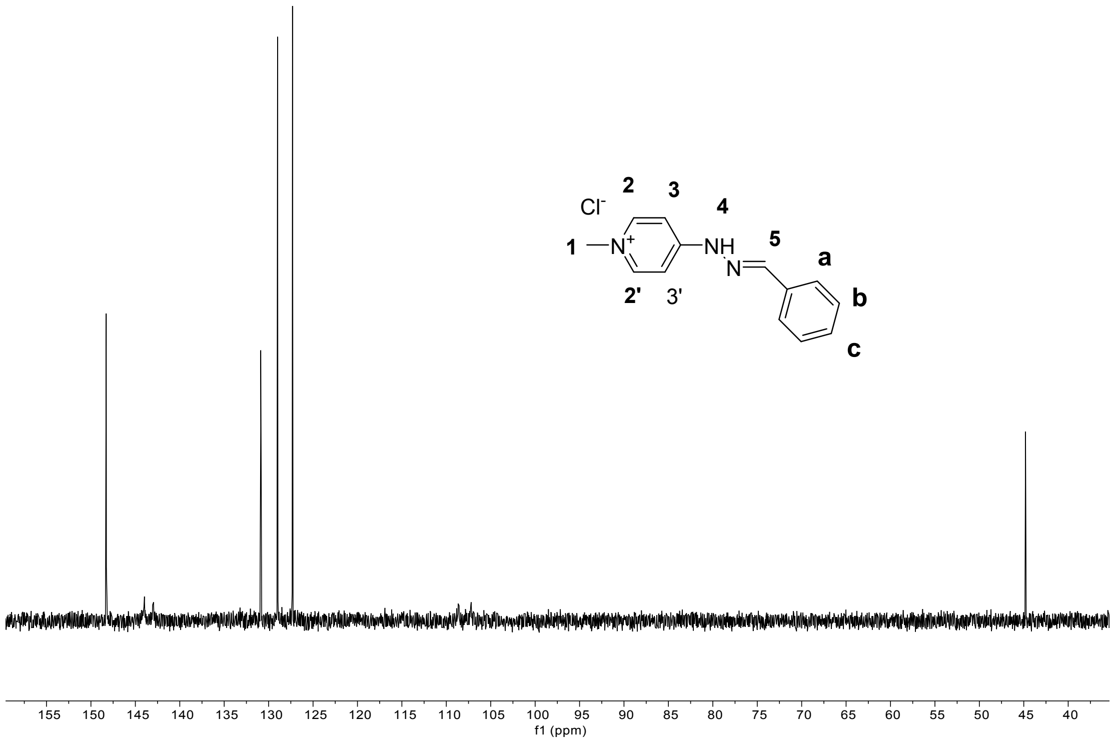

Figure S 79: DEPT-135 (101 MHz, D_2O) spectrum of $\mathbf{M_aH}$·Cl.

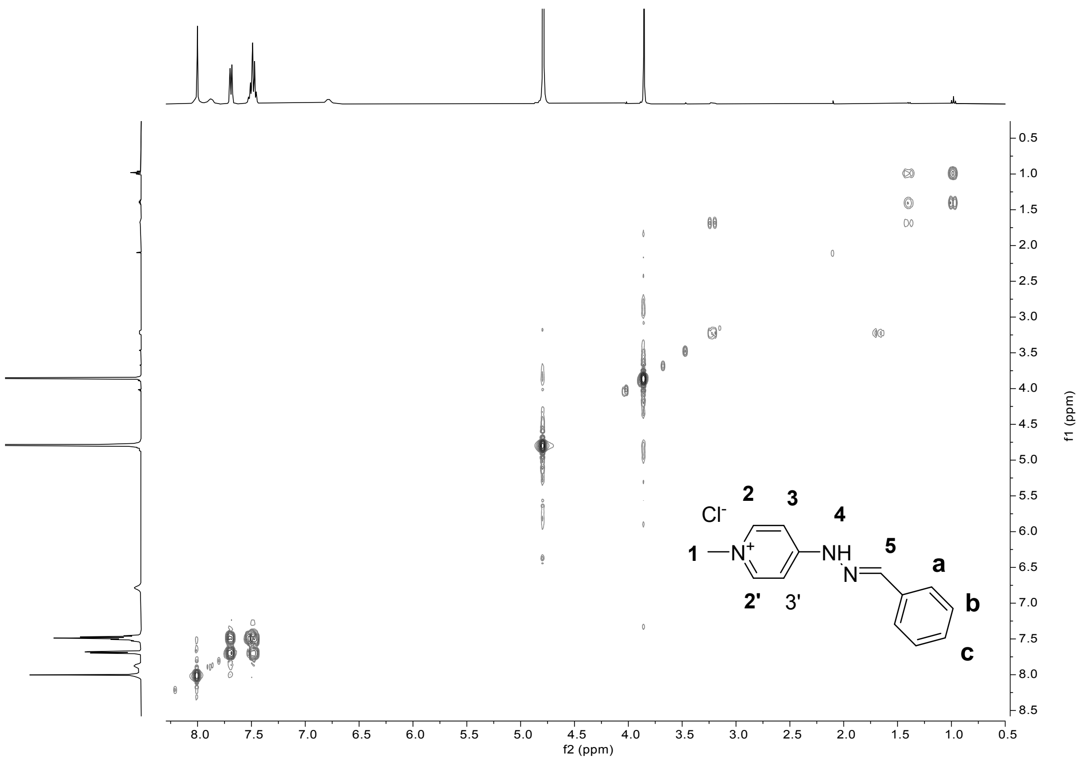

Figure S 80: COSY (400 MHz, D_2O) spectrum of $\mathbf{M_aH}$·Cl.

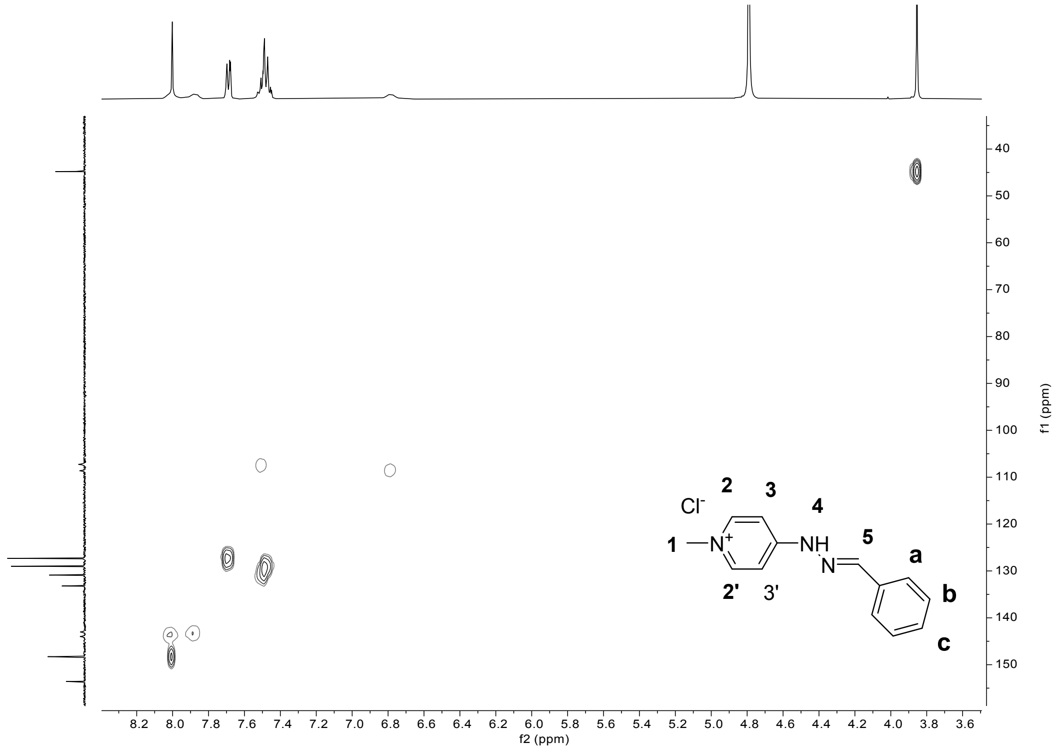

Figure S 81: HSQC (400 and 101 MHz, D_2O) spectrum of $\mathbf{M_aH}$·Cl.

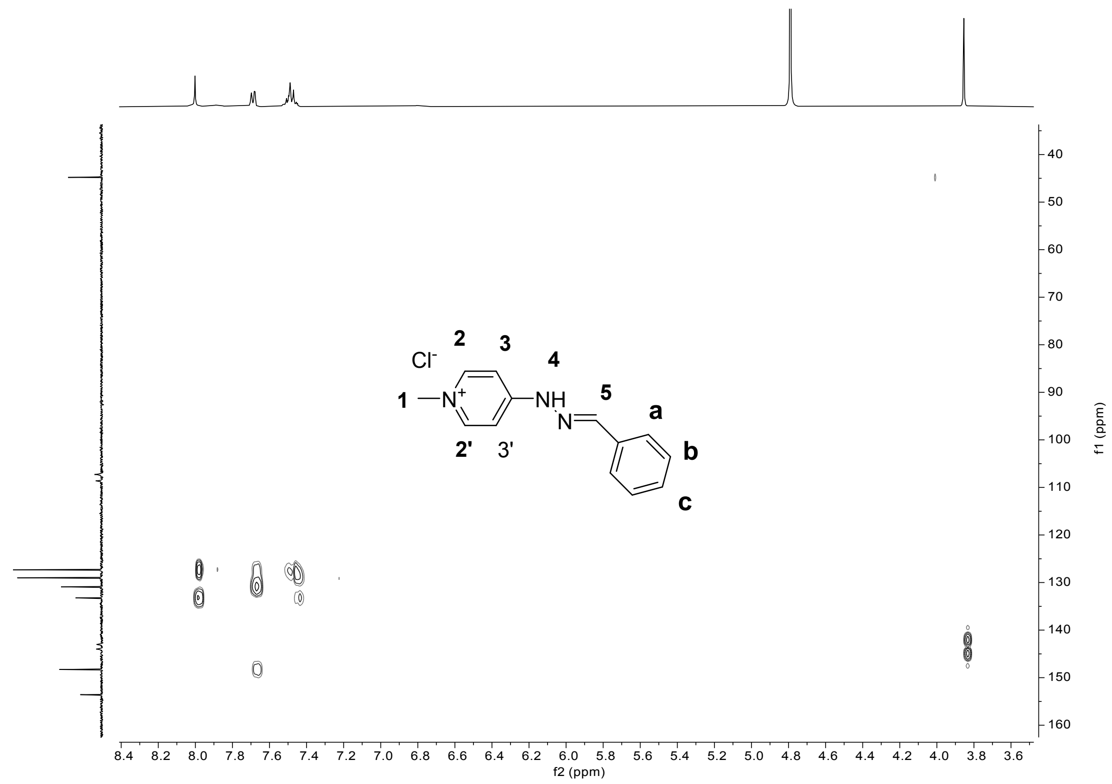

Figure S 82: HMBC (400 and 101 MHz, D_2O) spectrum of $\mathbf{M_aH}\cdot$Cl

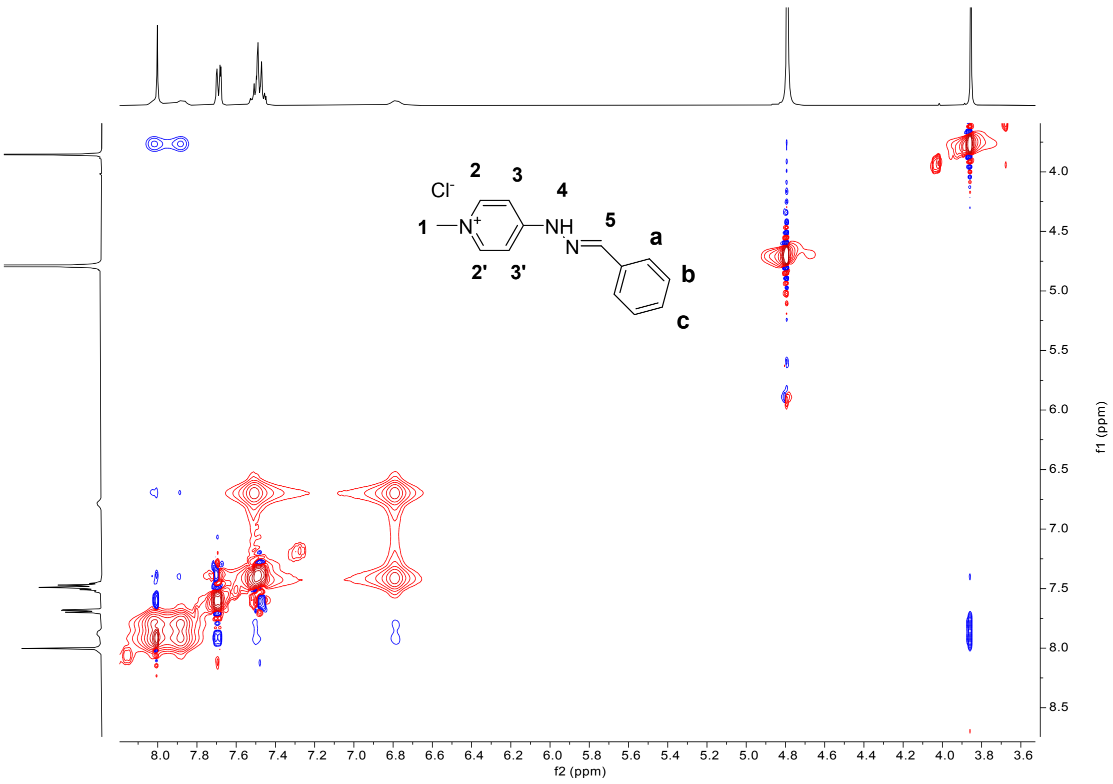

Figure S 83: ^1H-^1H NOESY (400 MHz, D2O) spectrum of $\mathbf{M_aH}\cdot$Cl.

- **M_a at pD = 12.**

^{1}H NMR (300 MHz, D_2O) δ 8.28 (s, 1H), 7.78 (dd, *J* = 7.7, 2.1 Hz, 2H), 7.54 – 7.35 (m, 5H), 7.15 (dd, *J* = 7.6, 2.8 Hz, 1H), 6.40 (dd, *J* = 7.4, 2.8 Hz, 1H), 3.64 (s, 3H).

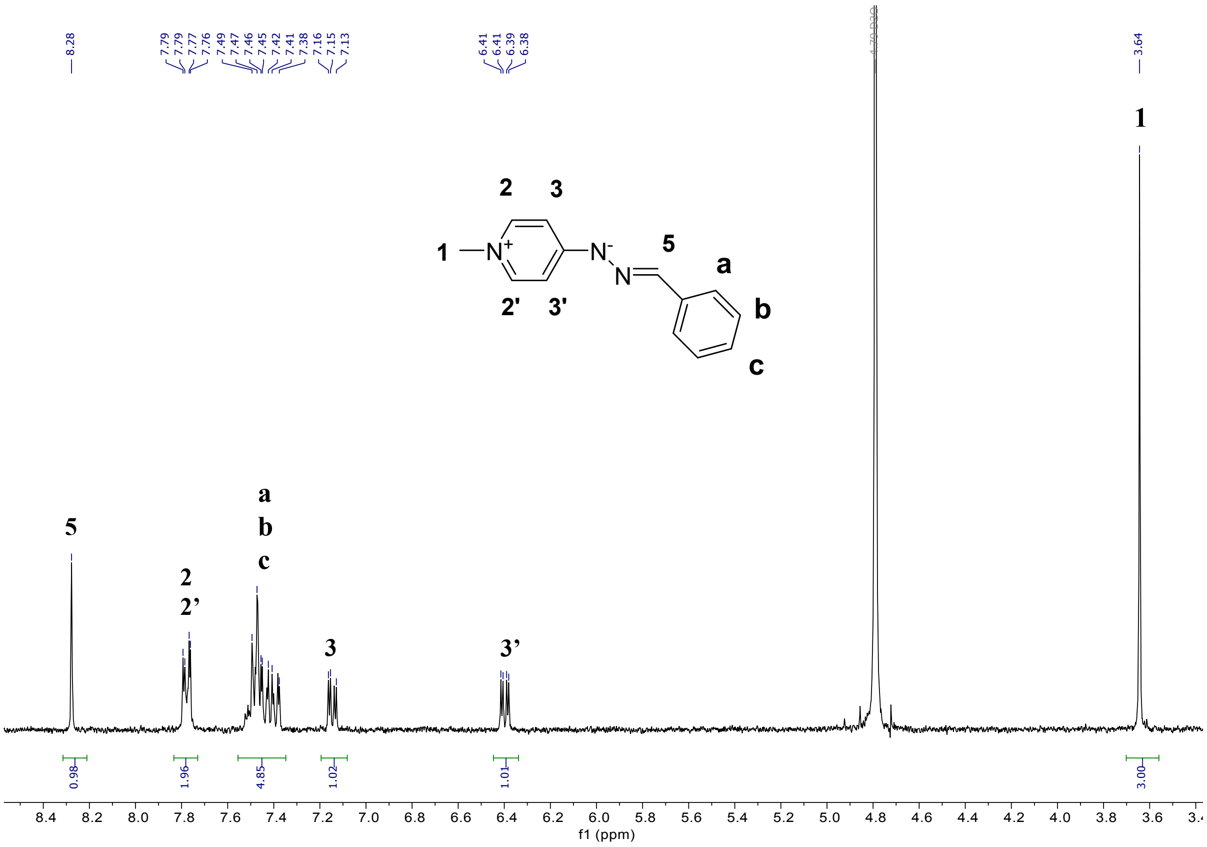

Figure S 84: ^{1}H NMR (300 MHz, D_2O) spectrum of $\mathbf{M_aH}$·Cl.

- **$M_aH{\cdot}PF_6$:**

^{1}H NMR (400 MHz, CD_3CN) δ 10.20 (s, 1H), 8.14 (s, 1H), 8.04 (dd, J = 33.8, 7.3 Hz, 2H), 7.86 – 7.79 (m, 2H), 7.68 (d, J = 7.3 Hz, 1H), 7.53 – 7.46 (m, 3H), 6.99 (d, J = 7.2 Hz, 1H), 3.96 (s, 3H) ppm. **^{13}C NMR** (101 MHz, CD_3CN) δ 154.72 (C), 148.90 (C=N), 145.28 (CH), 144.29 (CH), 134.13 (C), 131.50 (CH), 129.58 (CH), 128.10 (CH), 109.36 (CH), 108.19 (CH), 45.81 (CH_3) ppm.

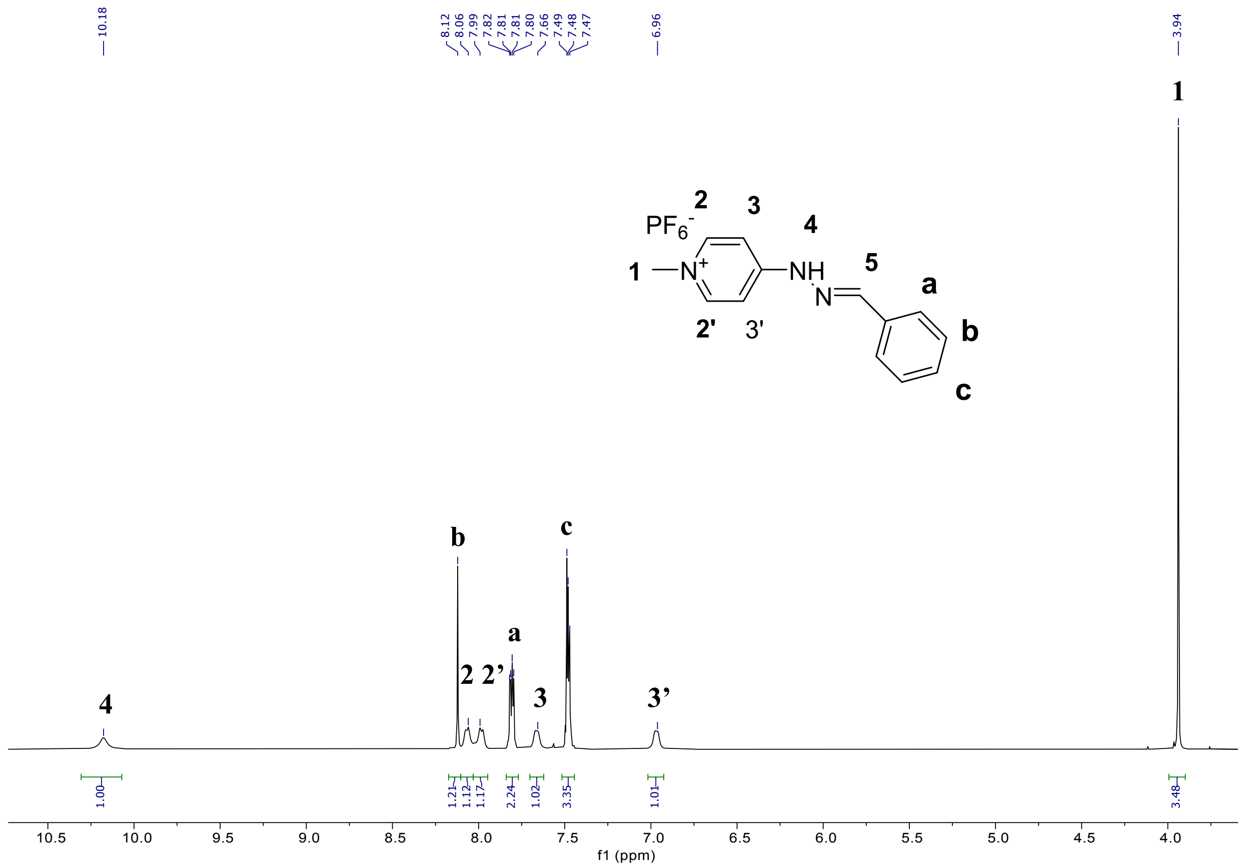

Figure S 85: ^{1}H NMR (400 MHz, CD_3CN) spectrum of **M_aH**$\cdot PF_6$.

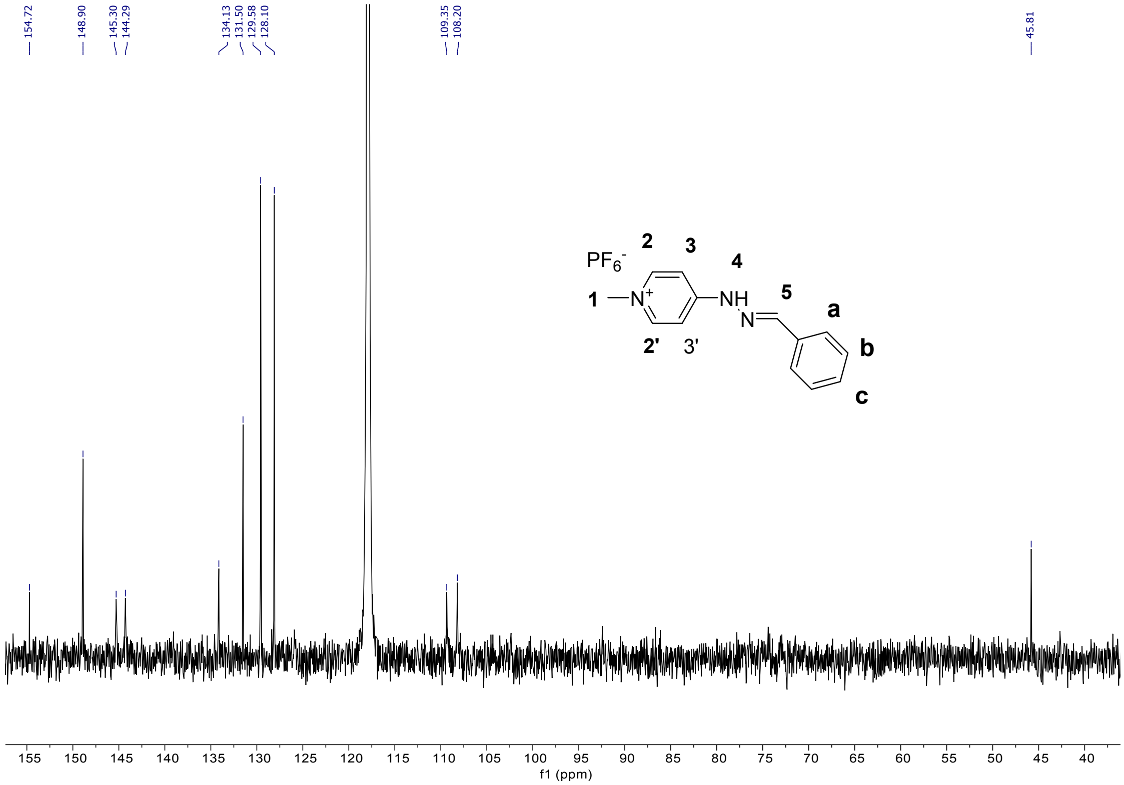

Figure S 86: ^{13}C NMR (101 MHz, CD_3CN) spectrum of $\mathbf{M_aH}\cdot PF_6$.

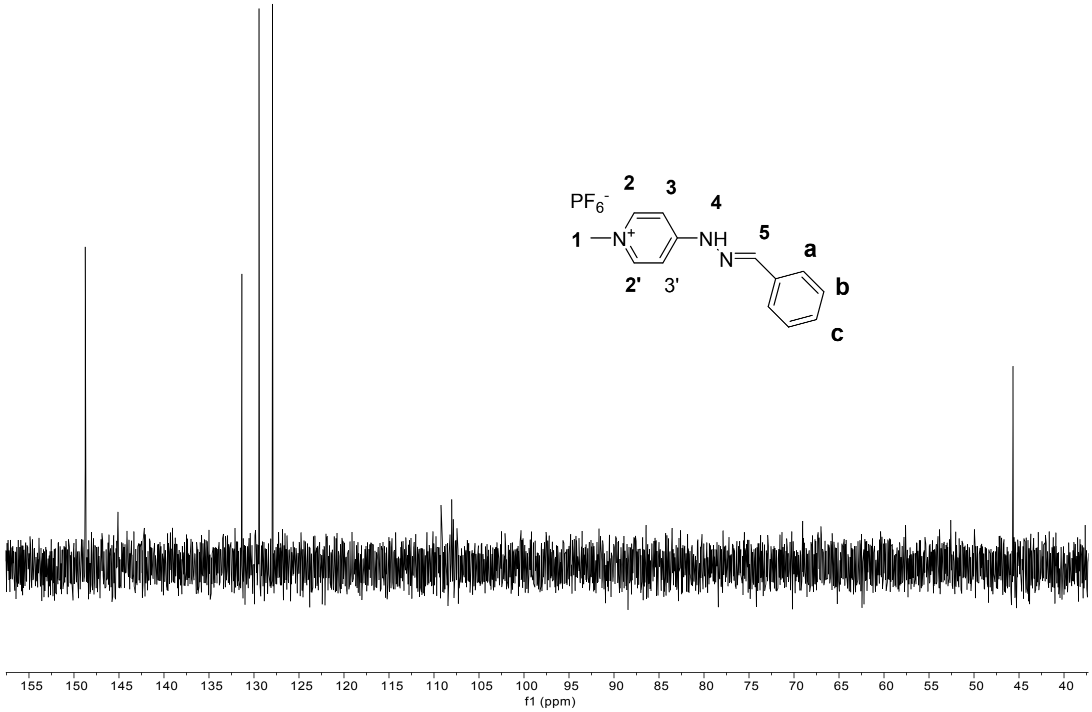

Figure S 87: DEPT-135 (101 MHz, CD_3CN) spectrum of $\mathbf{M_aH}\cdot PF_6$.

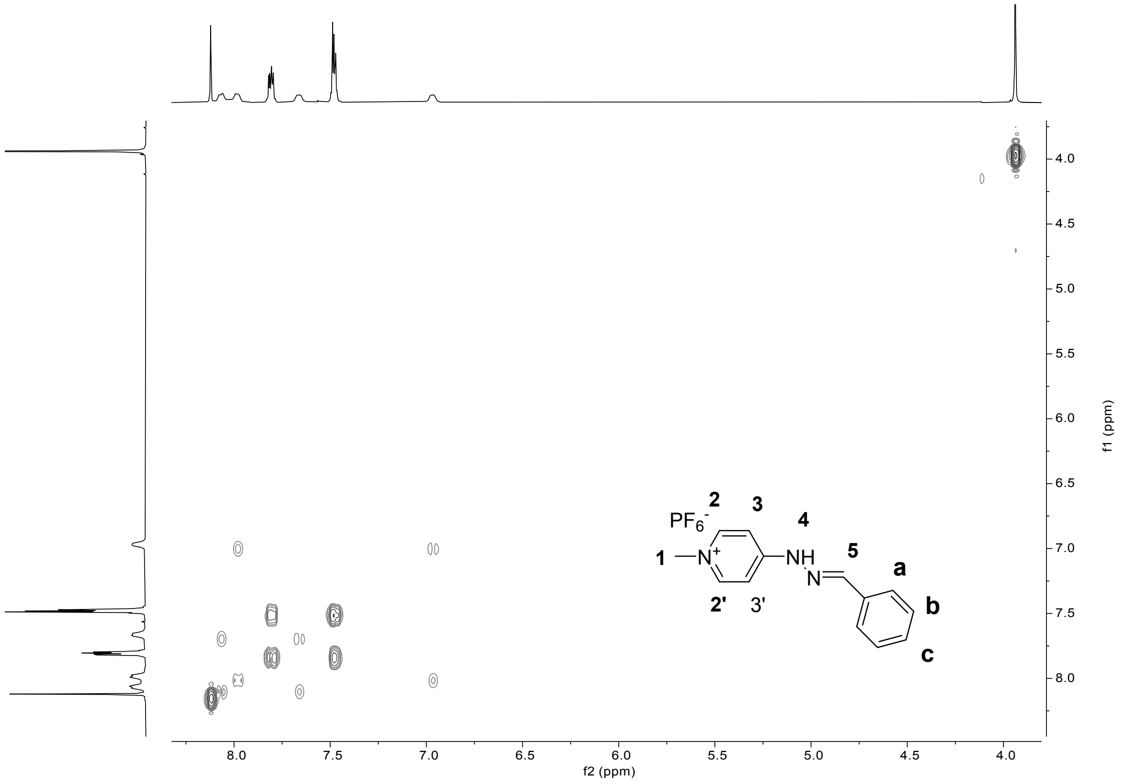

Figure S 88: COSY (400 MHz, CD_3CN) spectrum of $\mathbf{M_aH}\cdot PF_6$.

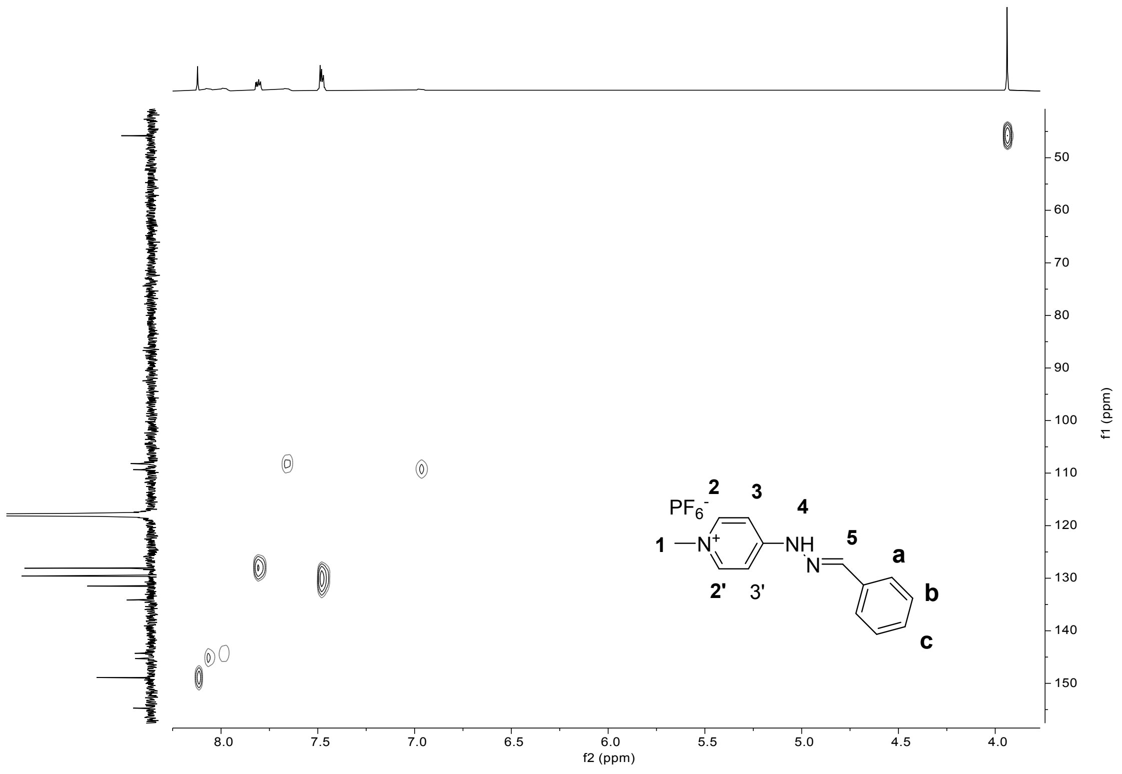

Figure S 89: HSQC (400 and 101 MHz, CD_3CN) spectrum of $\mathbf{M_aH}\cdot PF_6$.

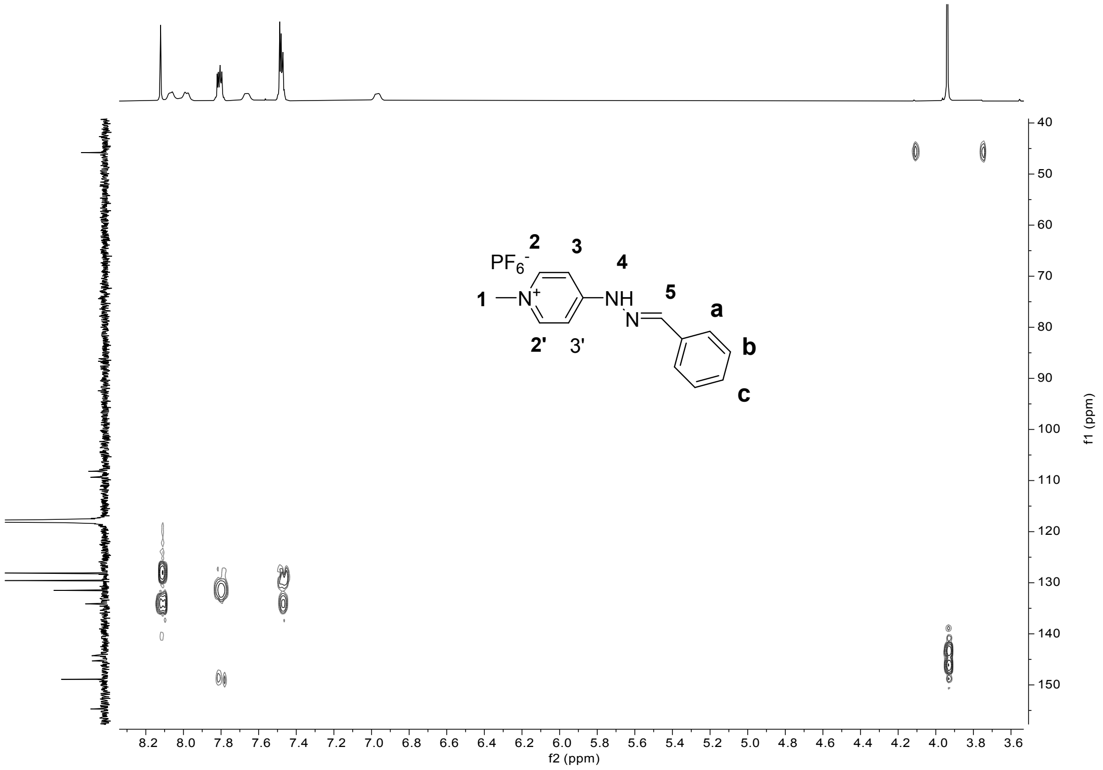

Figure S 90: HMBC (400 and 101 MHz, CD_3CN) spectrum of $\mathbf{M_aH}\cdot PF_6$.

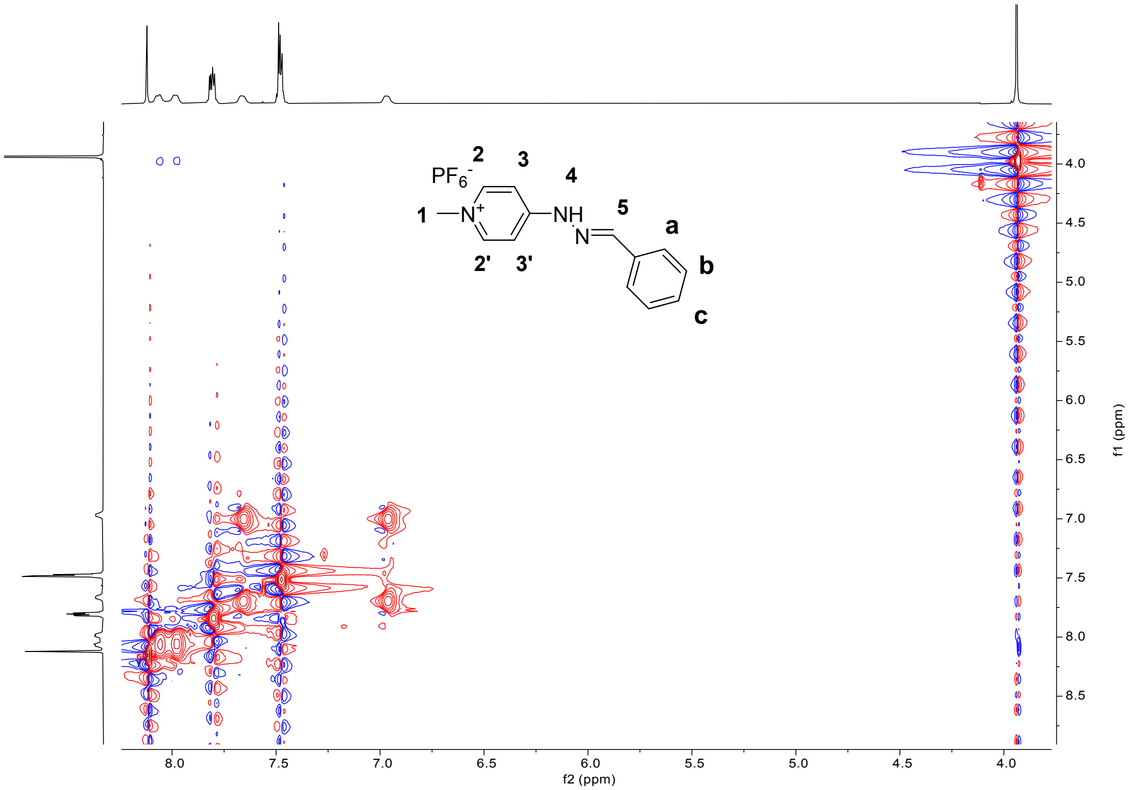

Figure S 91: NOESY (400 MHz, D_2O) spectrum of $\mathbf{M_aH}\cdot PF_6$.

2.9. Synthesis and characterization data of $M_bH{\cdot}Cl$

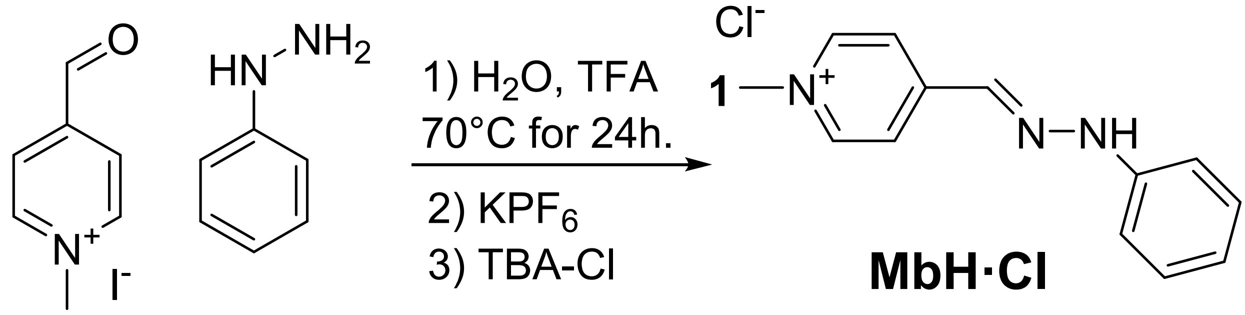

Same synthetic procedure described for $\mathbf{R_bH^{2+}}$.

$\mathbf{M_bH}{\cdot}PF_6$: orange powder (677 mg, 92%). $\mathbf{M_bH}{\cdot}Cl$: yellow solid (435 mg, 87%).

^{1}H NMR (400 MHz, D_2O) δ 8.23 (d, *J* = 6.7 Hz, 2H), 7.82 (d, *J* = 6.8 Hz, 2H), 7.64 (s, 1H), 7.40 – 7.30 (m, 2H), 7.15 – 7.02 (m, 3H), 4.02 (s, 3H) ppm. **^{13}C NMR** (101 MHz, D_2O) δ 150.94 (C), 143.68 (C=N), 142.67 (C), 129.94 (CH), 129.61 (CH), 122.33 (CH), 121.81 (CH), 113.45 (CH), 46.60 (CH_3) ppm. **HRMS (ESI)** (*m/z*): calcd for $[C_{13}H_{14}N_3]^+$ 212.1183, found 212.1182.

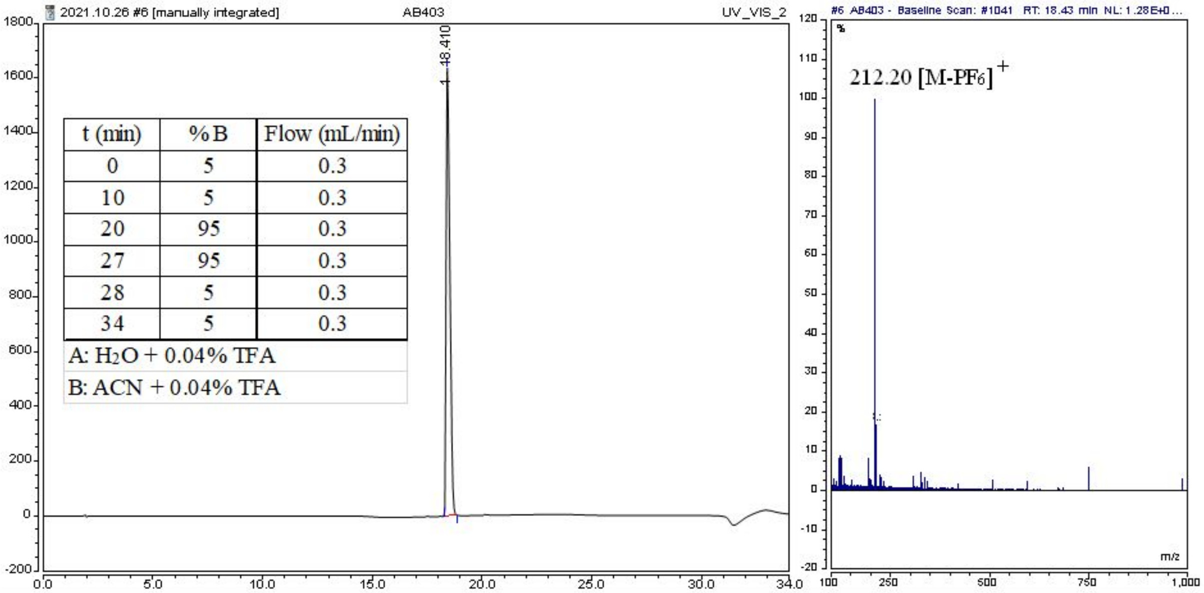

t (min)	%B	Flow (mL/min)
0	5	0.3
10	5	0.3
20	95	0.3
27	95	0.3
28	5	0.3
34	5	0.3

A: H_2O + 0.04% TFA
B: ACN + 0.04% TFA

Figure S 92. HPLC-MS chromatogram of $\mathbf{M_bH}{\cdot}PF_6$. *Inset.* Table of the elution conditions.

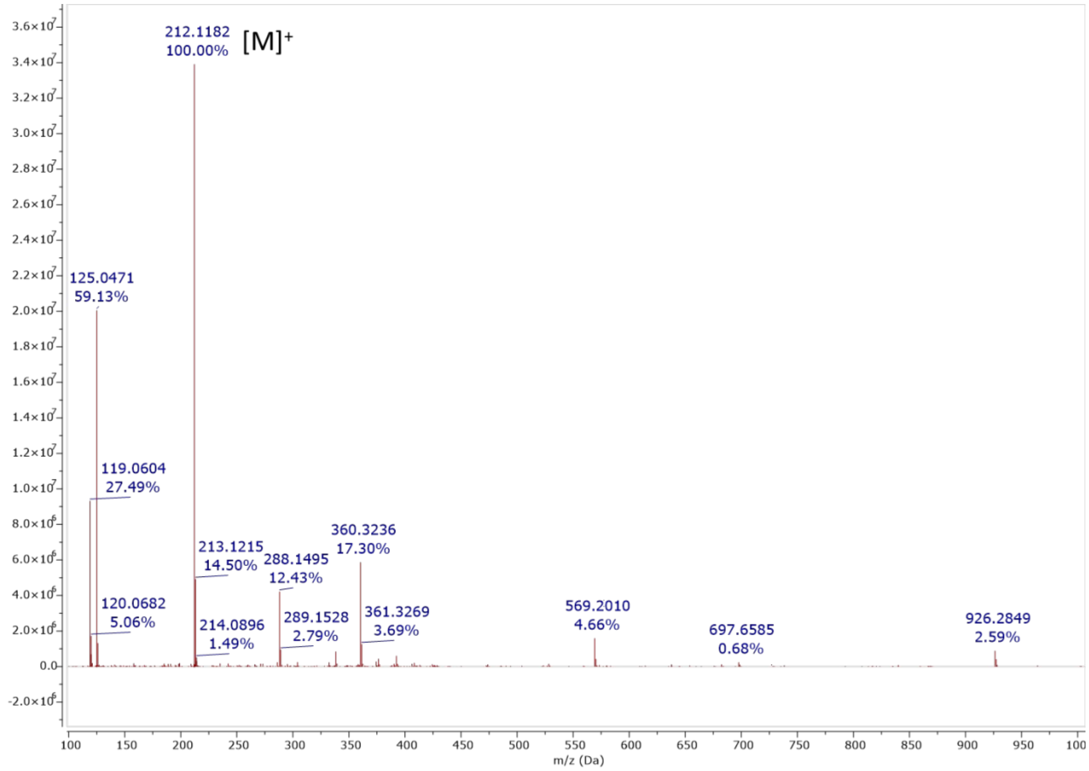

Figure S 93. HR ESI-MS spectrum of $\mathbf{M_b}$H·2PF$_6$.

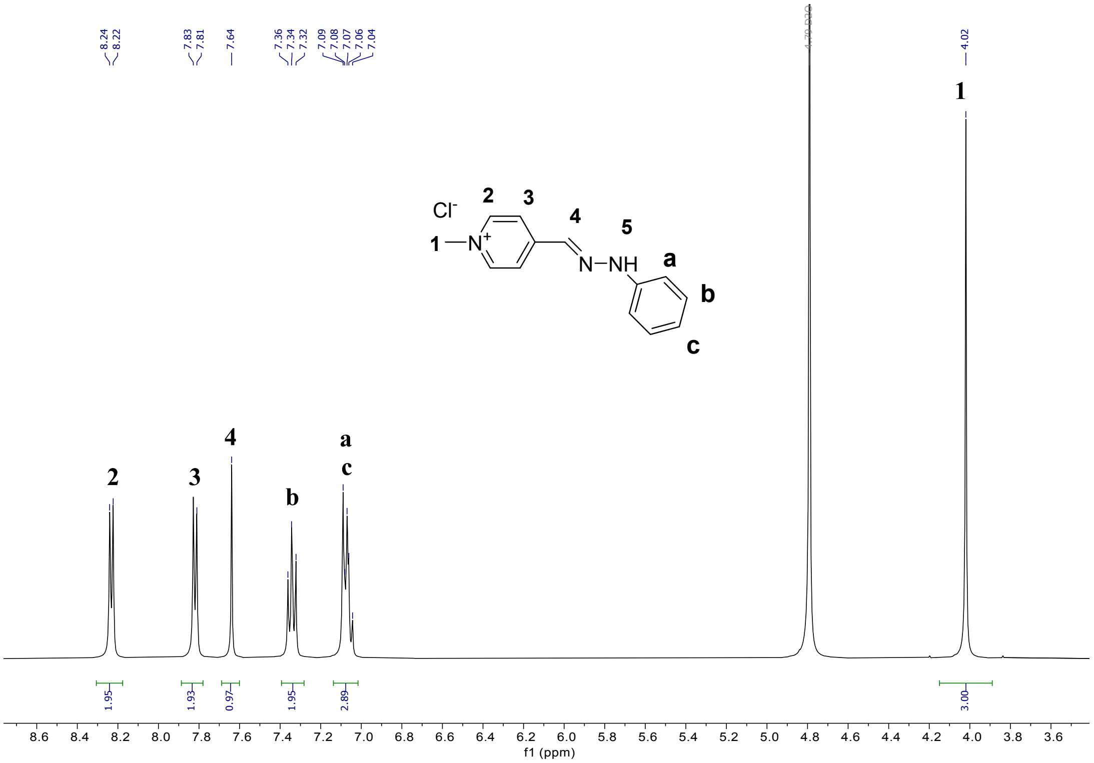

Figure S 94: ^{1}H NMR (400 MHz, D$_2$O) spectrum of $\mathbf{M_bH}$·Cl.

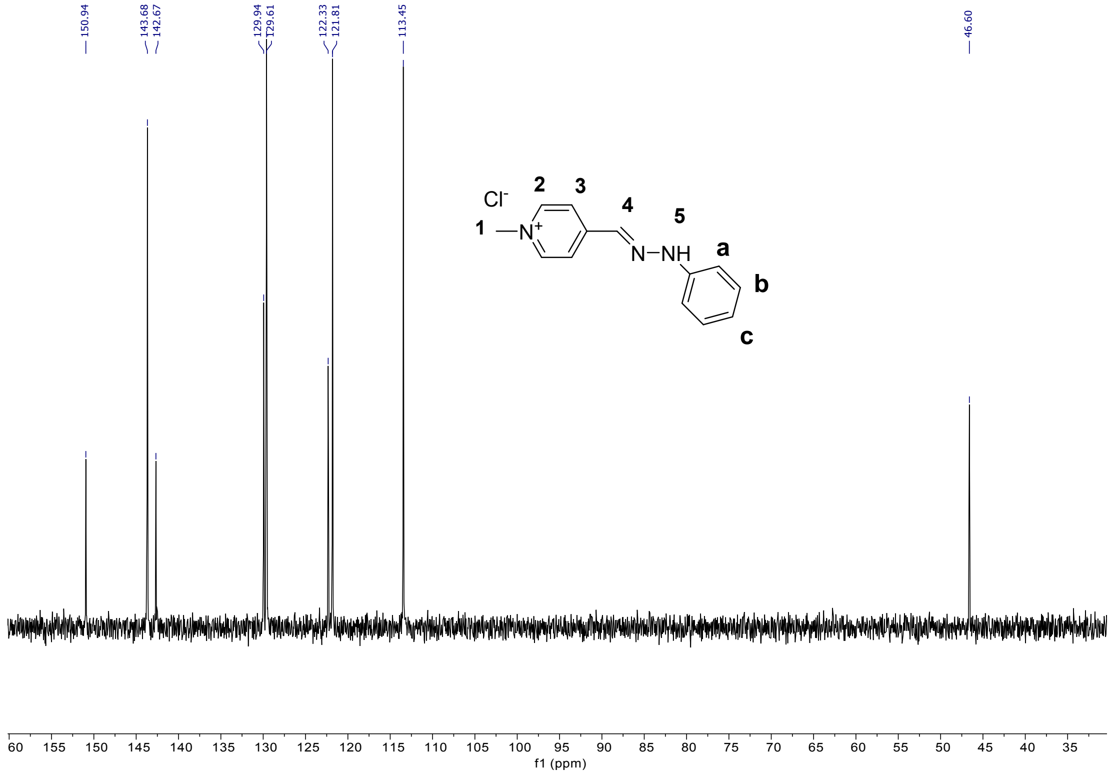

Figure S 95: ^{13}C NMR (101 MHz, D_2O) spectrum of $\mathbf{M_bH}$·Cl.

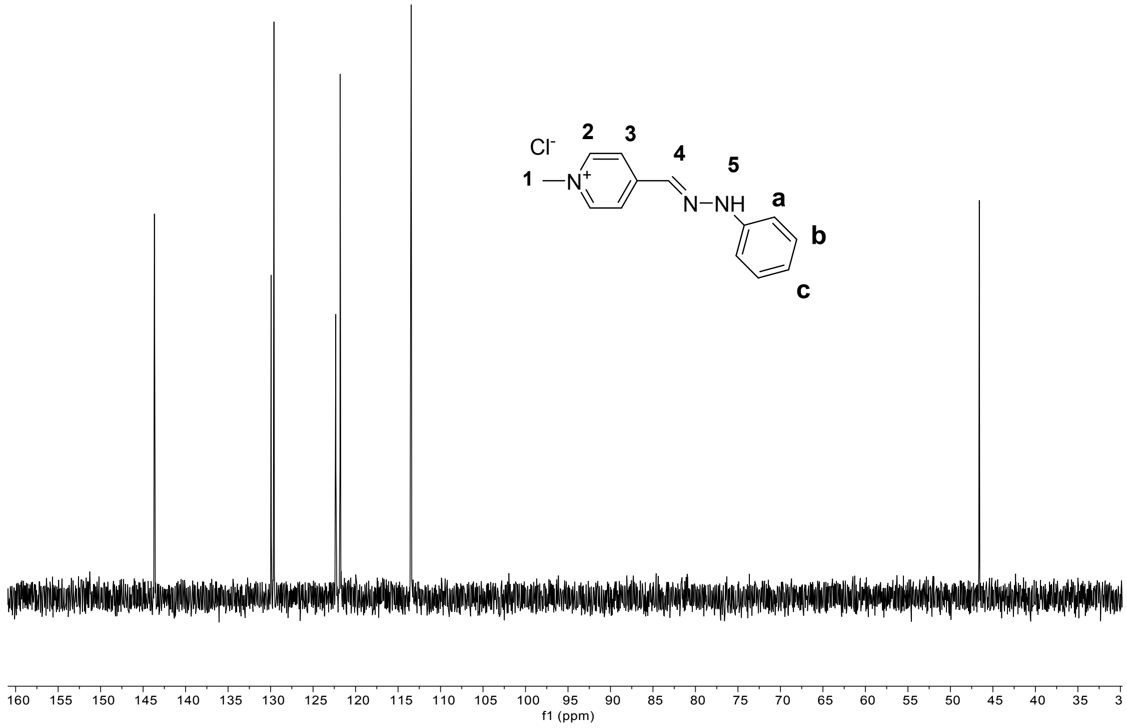

Figure S 96: DEPT-135 (101 MHz, D2O) spectrum of $\mathbf{M_bH}$·Cl.

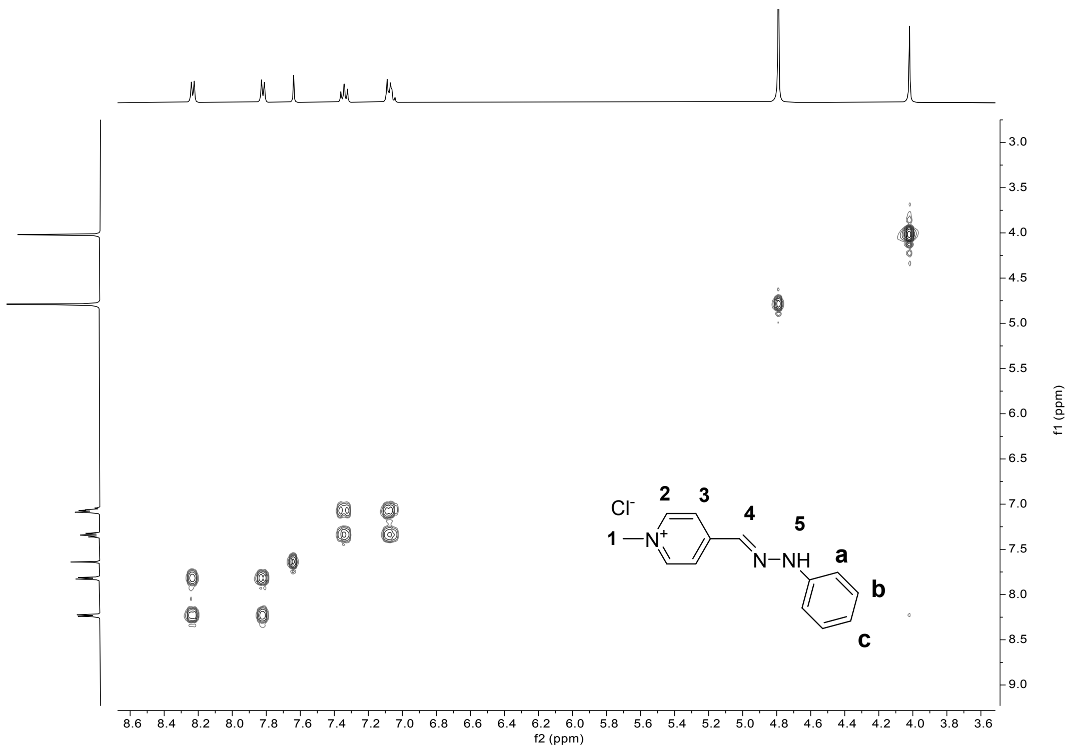

Figure S 97: COSY (400 MHz, D_2O) spectrum of $\mathbf{M_bH}$·Cl.

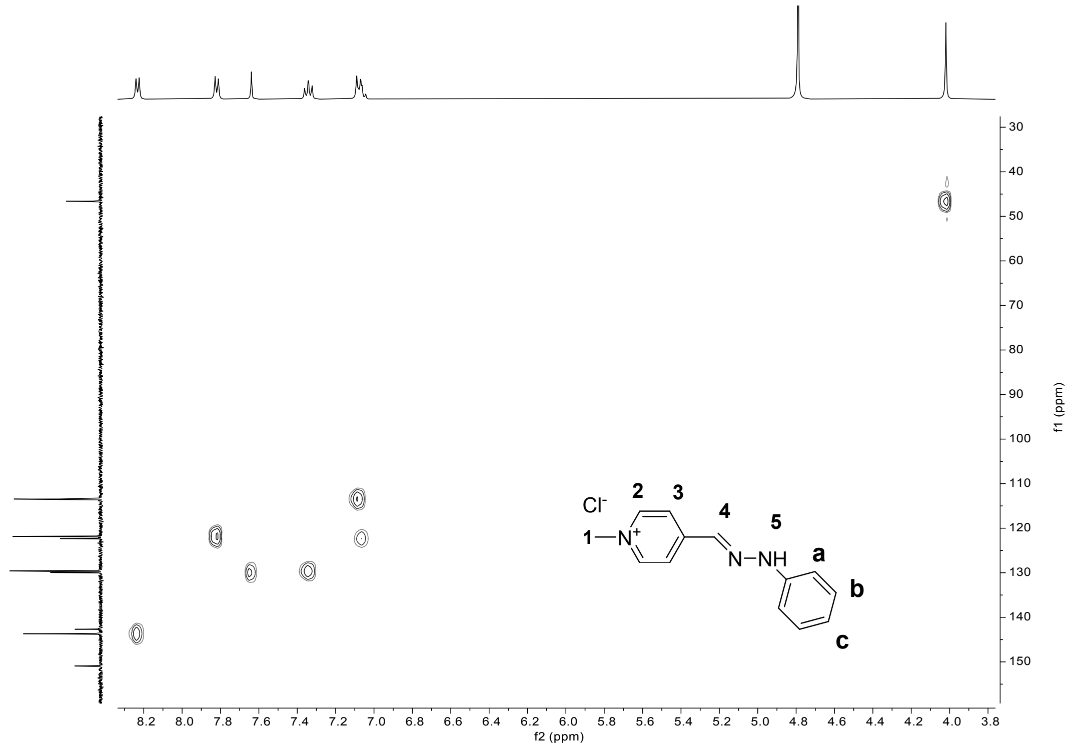

Figure S 98: HSQC (400 and 101 MHz, D_2O) spectrum of $\mathbf{M_bH}$·Cl.

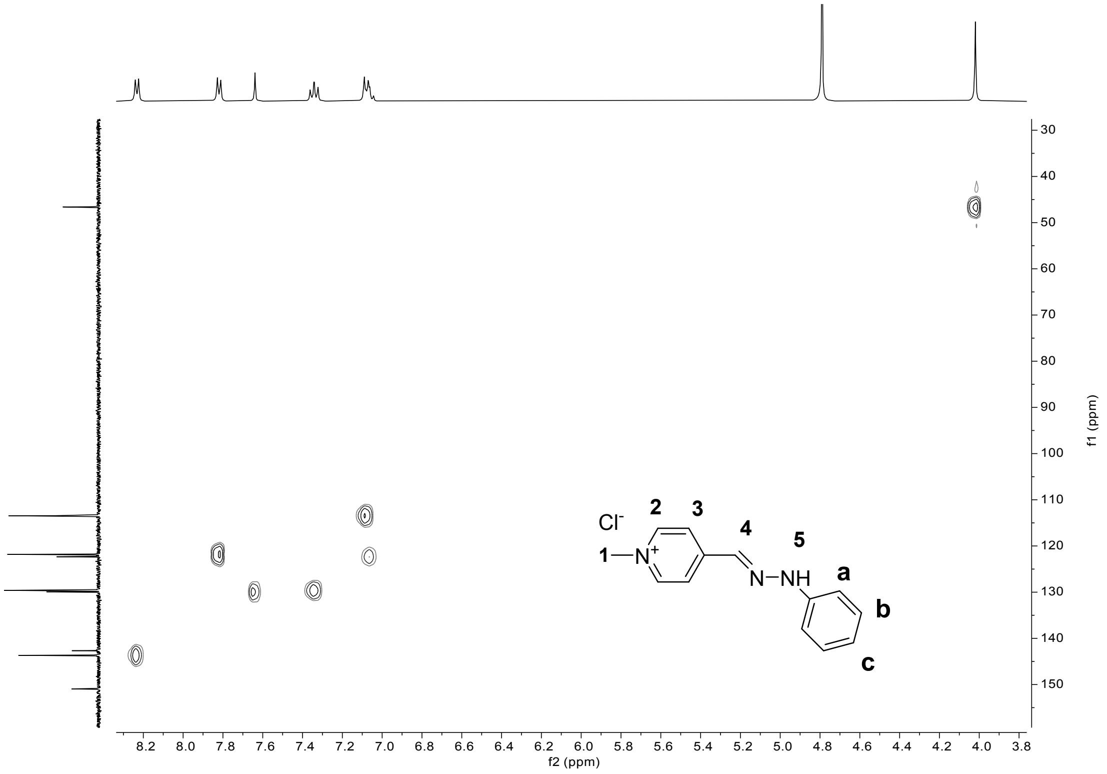

Figure S 99: HMBC (400 and 101 MHz, D_2O) spectrum of $\mathbf{M_bH}$·Cl.

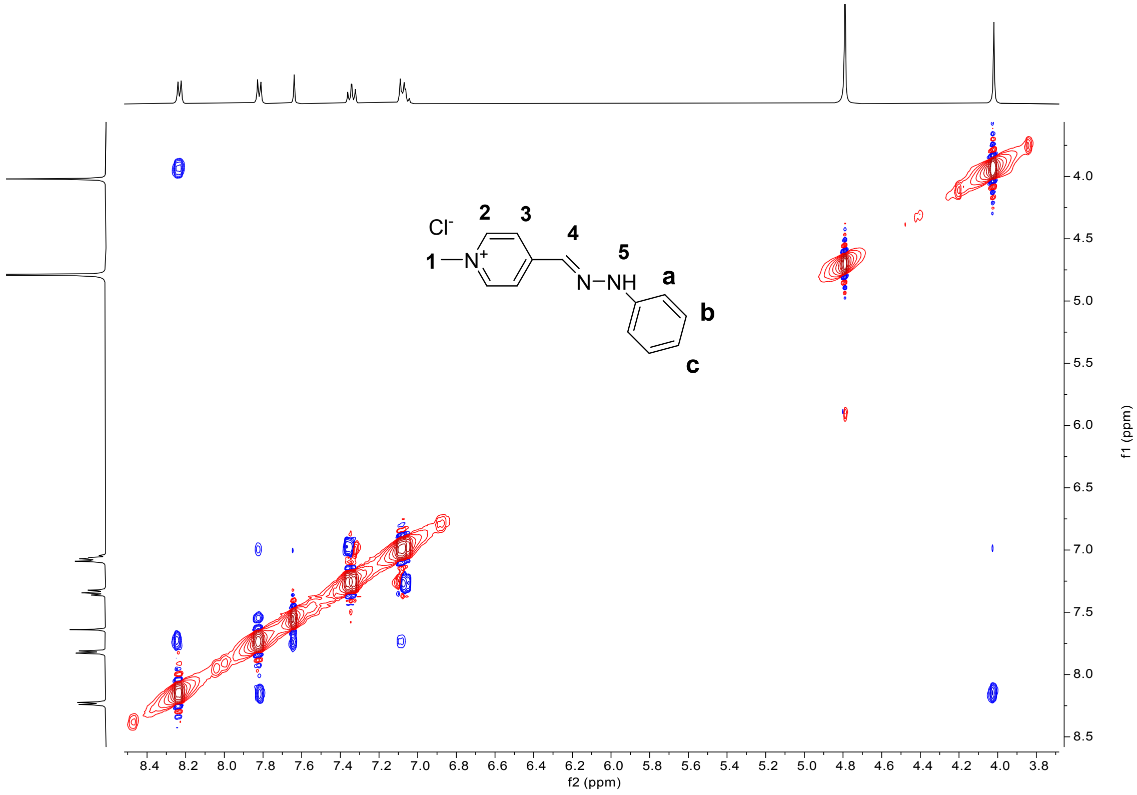

Figure S 100: NOESY (400 MHz, D_2O) spectrum of $\mathbf{M_bH}$·Cl.

- **M_b^+ at pD = 12:**

^{1}H NMR (300 MHz, D_2O) δ 8.10 (d, *J* = 6.7 Hz, 2H), 7.85 – 7.65 (m, 3H), 7.52 – 7.25 (m, 4H), 7.07 (t, *J* = 7.1 Hz, 1H), 4.01 (s, 3H).

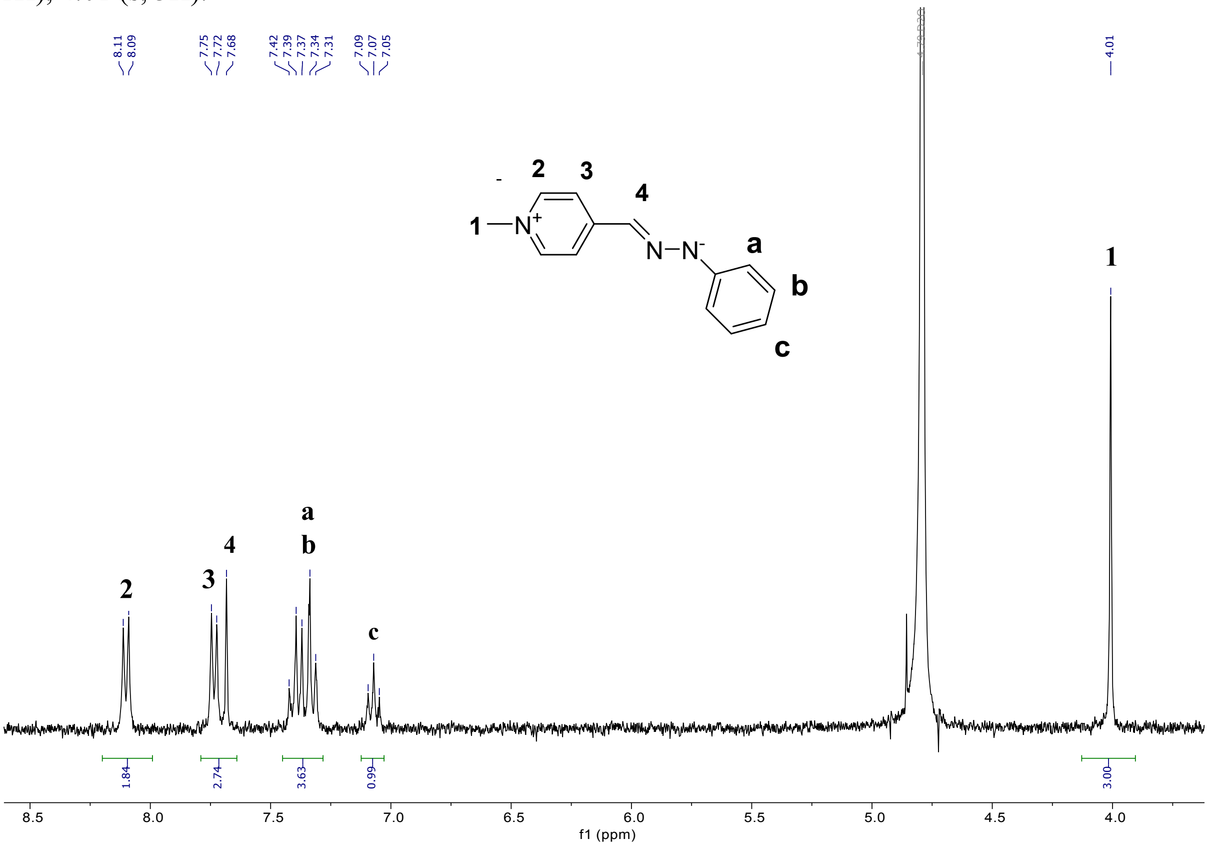

Figure S 101: ^{1}H NMR (300 MHz, D_2O) spectrum of $\mathbf{M_b}$ at pD=12.

- **$M_bH\cdot PF_6$:**

^{1}H NMR (400 MHz, CD_3CN) δ 9.94 (s, 1H), 8.34 (d, 2H), 8.02 (d, 2H), 7.79 (s, 1H), 7.36 (t, 2H), 7.29 (d, 2H), 7.04 (t, J = 1.3 Hz, 1H), 4.15 (s, 3H) ppm. **^{13}C NMR** (101 MHz, CD_3CN) δ 152.29 (C), 144.93 (C=N), 143.59 (C), 130.13 (CH), 130.06 (CH), 123.26 (CH), 122.58 (CH), 114.46 (CH), 47.57 (CH_3) ppm.

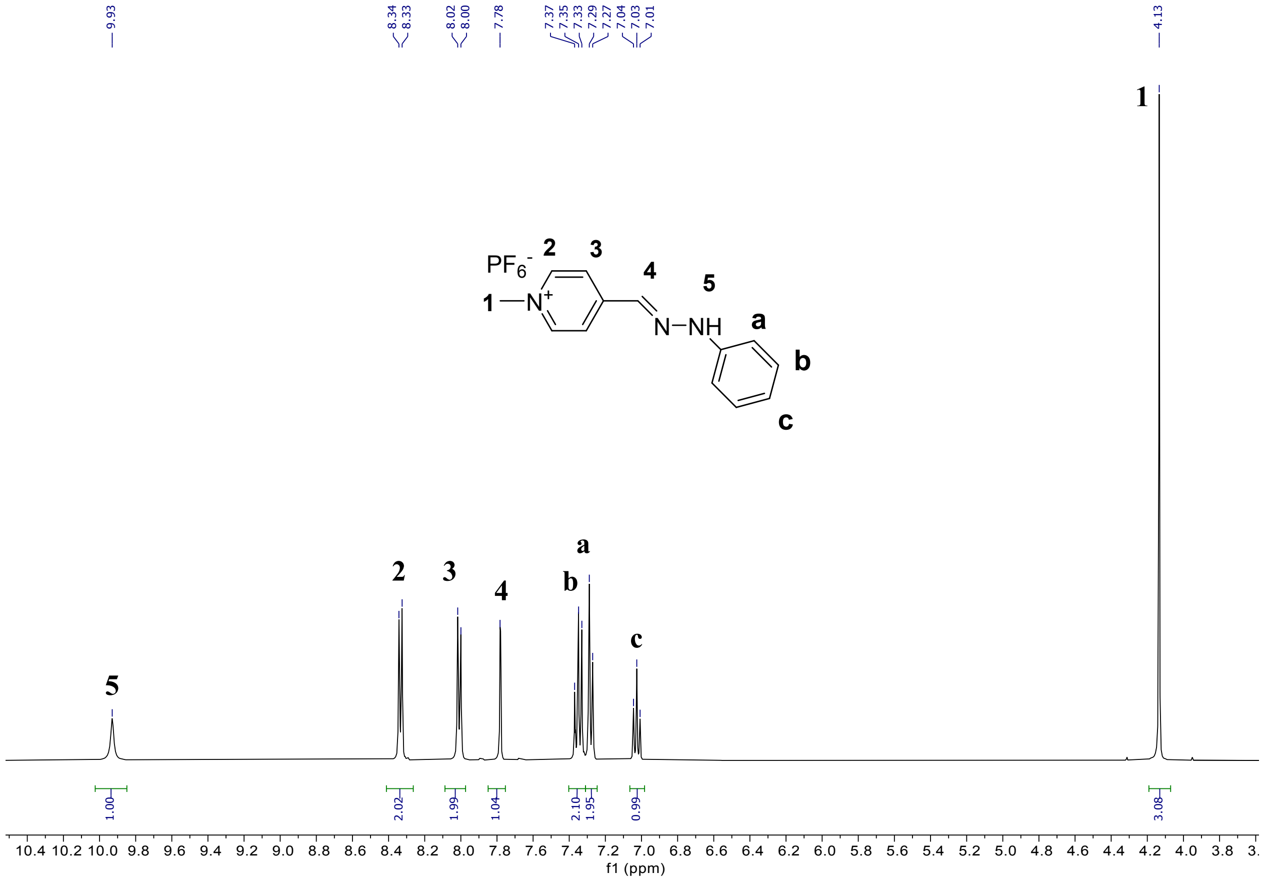

Figure S 102: ^{1}H NMR (400 MHz, CD_3CN) spectrum of $\mathbf{M_bH}\cdot PF_6$.

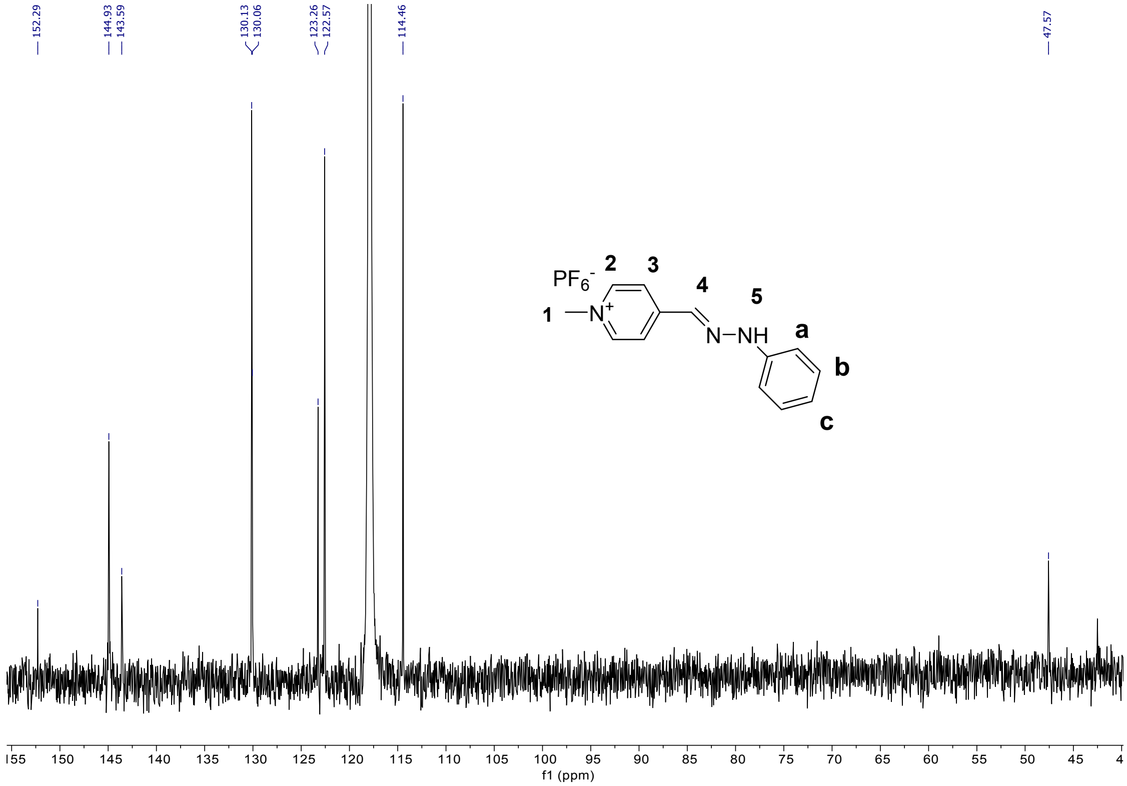

Figure S 103: ^{13}C NMR (101 MHz, CD3CN) spectrum of $\mathbf{M_bH}\cdot PF_6$.

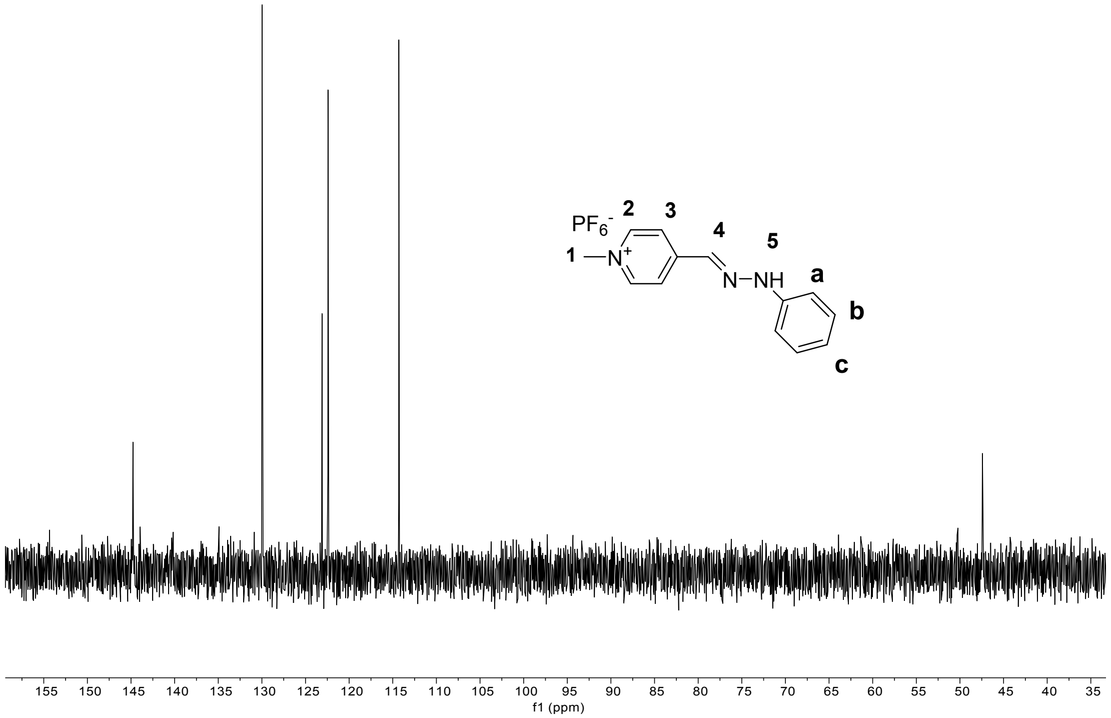

Figure S 104: DEPT-135 (101 MHz, CD_3CN) spectrum of $\mathbf{M_bH}\cdot PF_6$.

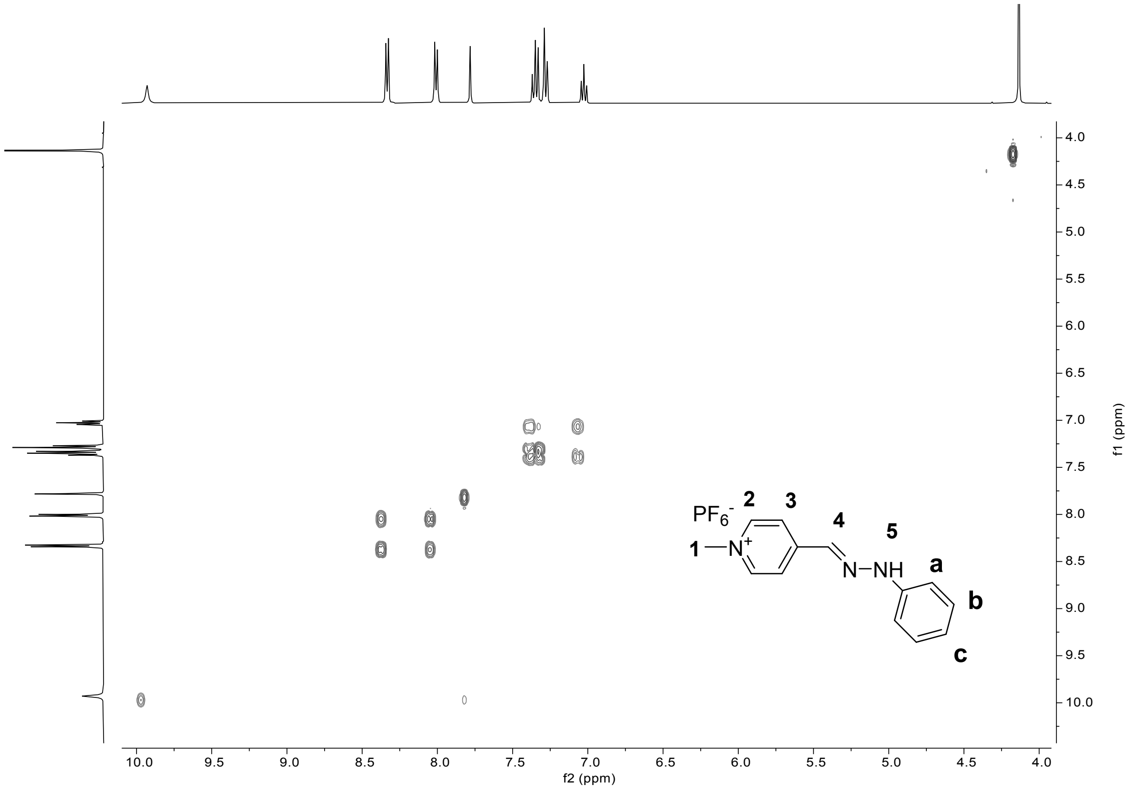

Figure S 105: ^{1}H-^{1}H COSY (400 MHz, CD_3CN) spectrum of $\mathbf{M_bH}\cdot PF_6$.

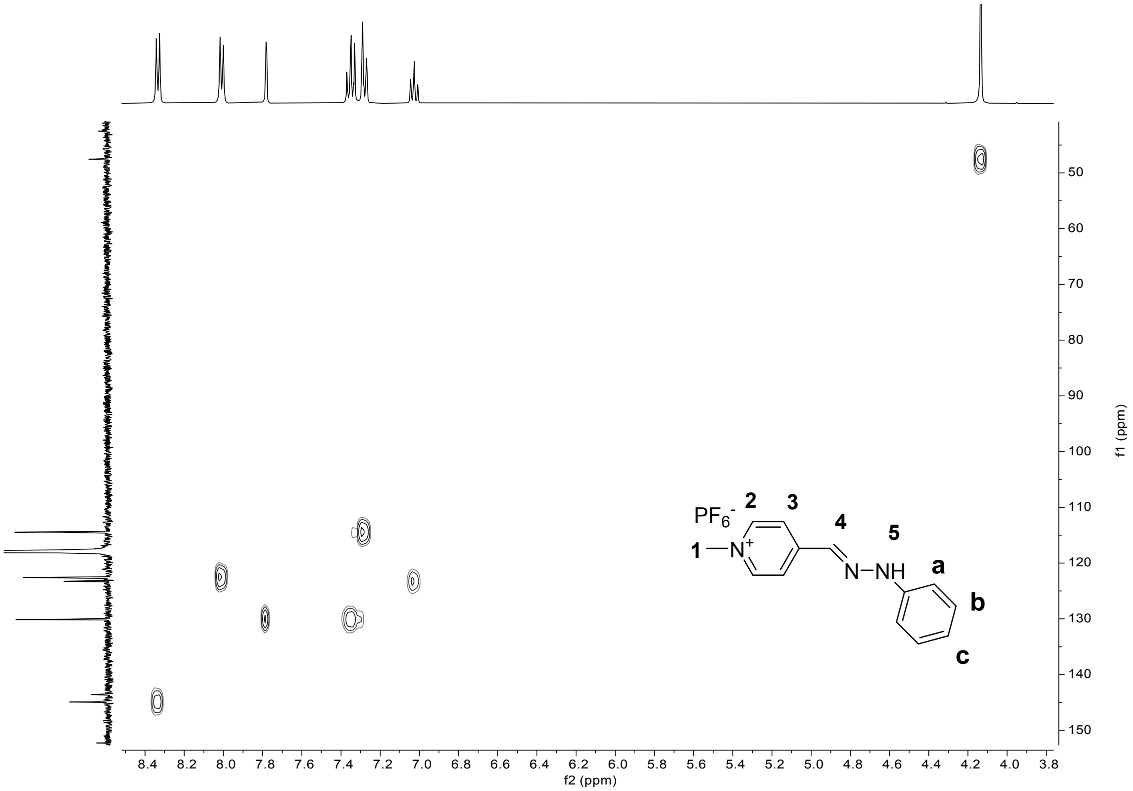

Figure S 106: ^{1}H-^{13}C HSQC (400 and 101 MHz, CD_3CN) spectrum of $\mathbf{M_bH}\cdot PF_6$.

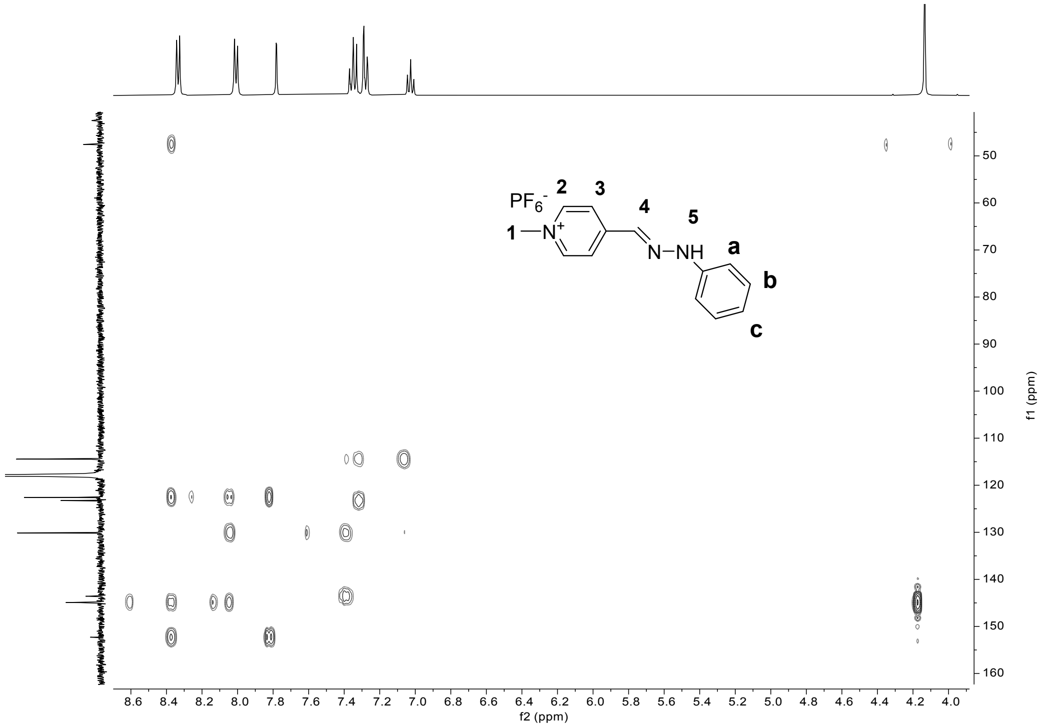

Figure S 107: ^{1}H-^{13}C HMBC (400 and 101 MHz, CD_3CN) spectrum of $\mathbf{M_bH}\cdot PF_6$.

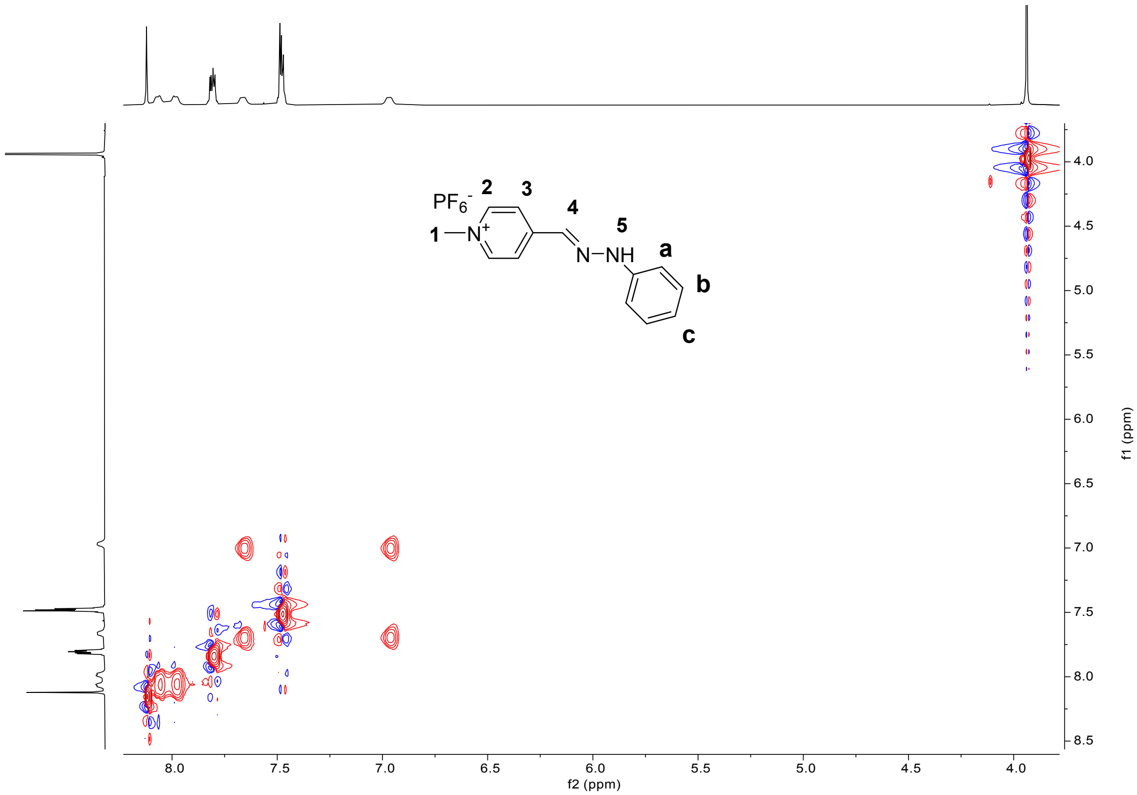

Figure S 108: ^{1}H-^{1}H NOESY (400 MHz, CD_3CN) spectrum of $\mathbf{M_bH}\cdot PF_6$.

3. Anion interaction study with R_aH^{2+}

All alkylammonium salts used were previously dried under vacuum in an inert atmosphere. Spectrophotometric titrations were carried out in ACN by keeping the $\mathbf{R_a}H\cdot 2PF_6$ concentration constant (1-33 x 10^{-6} M) and adding increasing aliquots of the corresponding salt. All UV-vis titration data were fitted with Dynafit software.[3] For the evaluation of goodness-of-fit, Dynafit software employs the analysis of the residuals through the runs-of-sign test.[4] Thus, by analyzing the randomness of the residuals plot against the independent constant (concentration) and the *p* parameter (> 0.01), we can confirm the fit model used.

NMR titrations could not be performed due to the precipitation of $\mathbf{R_a}H^{2+}$ upon 2 eqv. of the corresponding salt. Spectra were recorded at 0, 0.5, 1, 2 and 4 eq. of the corresponding salt to follow the chemical shift of the iminic signal 5.

3.1. 2F□R_aH^{2+} titration by UV-vis in ACN.

The mechanism proposed for the fitting process equilibria, and introduced on the software Dynafit[3] was the following:

$$\mathbf{RaH^{+2} + F^{-} \Leftrightarrow F^{-}\subset RaH^{+2}}$$

$$\mathbf{F^{-}\subset RaH^{+2} + F^{-} \Leftrightarrow 2F^{-}\subset RaH^{+2}}$$

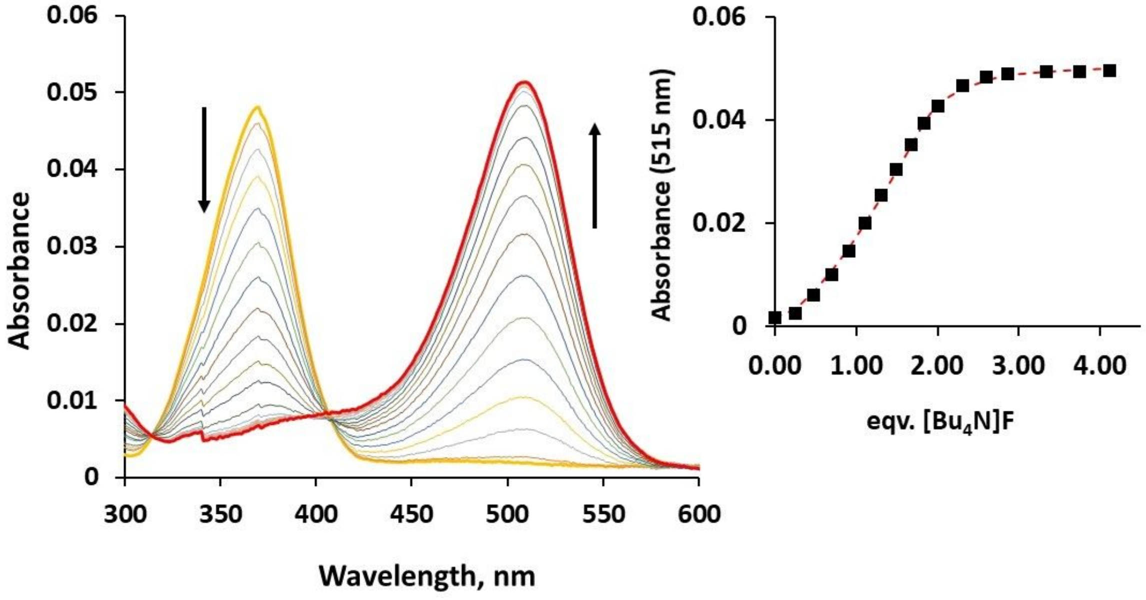

Figure S 109: UV-vis staked spectra of the $\mathbf{R_aH}\cdot 2PF_6$ (1 μM) titration with 100 μM solution of $[Bu_4N]F$. *Inset*: Fitting of the UV-vis titration data at 515 nm.

[3] Kuzmic, P. Program DYNAFIT for the Analysis of Enzyme Kinetic Data: Application to HIV Proteinase. *Anal. Biochem.* **1996**, *237*, 260-273.
[4] Kuzmic, P.; Lorenz, T.; Reinstein, J. Analysis of residuals from enzyme kinetic and protein folding experiments in the presence of correlated experimental noise. *Anal. Biochem.* **2009**, *395*, 1-7.

Concentration (µM)	Absorbance	Residual
0	0.00191943	0.000356065
0.24	0.00261302	-0.000346595
0.48	0.00632244	-0.000092451
0.7	0.0104687	-0.000178457
0.91	0.0152304	-0.000169392
1.11	0.0206256	0.000164765
1.3	0.0260727	0.000405880
1.49	0.0313312	0.000197189
1.67	0.036243	-0.000065244
1.84	0.0403553	-0.000399266
2	0.0438355	-0.000229641
2.3	0.047897	0.000235389
2.6	0.0496515	0.000391607
2.86	0.0502546	0.000266099
3.33	0.050695	-0.000002446
3.75	0.0507708	-0.000272858
4.12	0.0509445	-0.000299685

Table S 1: Experimental data of the titration. The *p* value obtained from "runs-of-sign" was 0.03.

3.2. 2F□R_aH^{2+} study by ^{1}H-NMR.

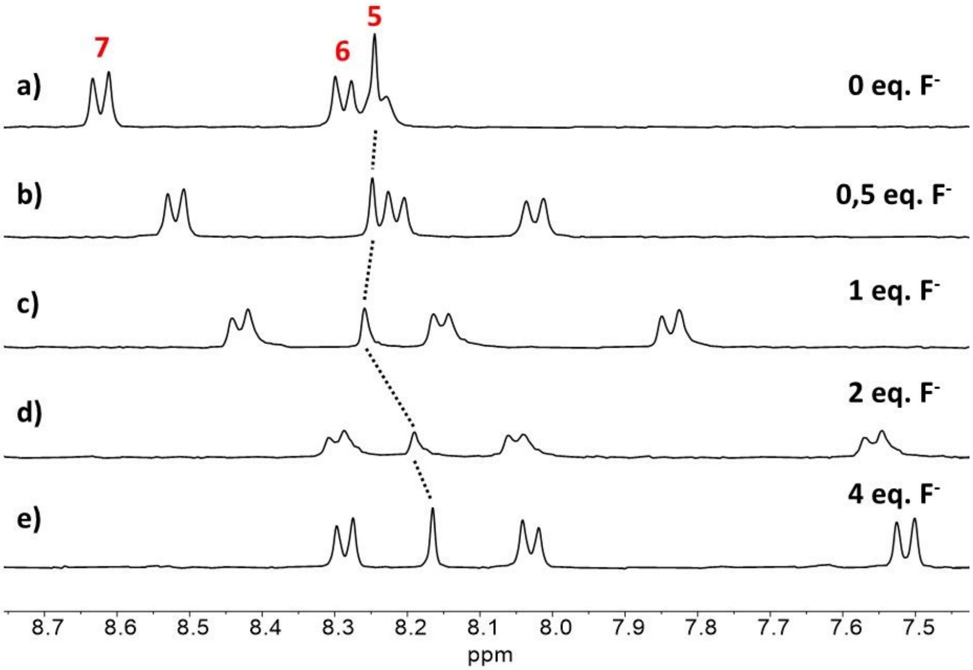

Figure S 110: ^{1}H-NMR (300 MHz, CD_3CN, 295.15 K) stacked spectra of $\mathbf{R_aH^{2+}}$ upon additions of a) 0 eq., b) 0.5 eq., c) 1 eq., d) 2 eq. and e) 4 eq. of $[Bu_4N]F$.

3.3. Cl□R_aH^{2+} titration by UV-vis in ACN.

The mechanism proposed for the fitting process equilibria, and introduced on the software Dynafit[3] was the following:

$$\mathbf{RaH^{+2} + Cl^{-} \Leftrightarrow Cl^{-}{\subset}RaH^{+2}}$$

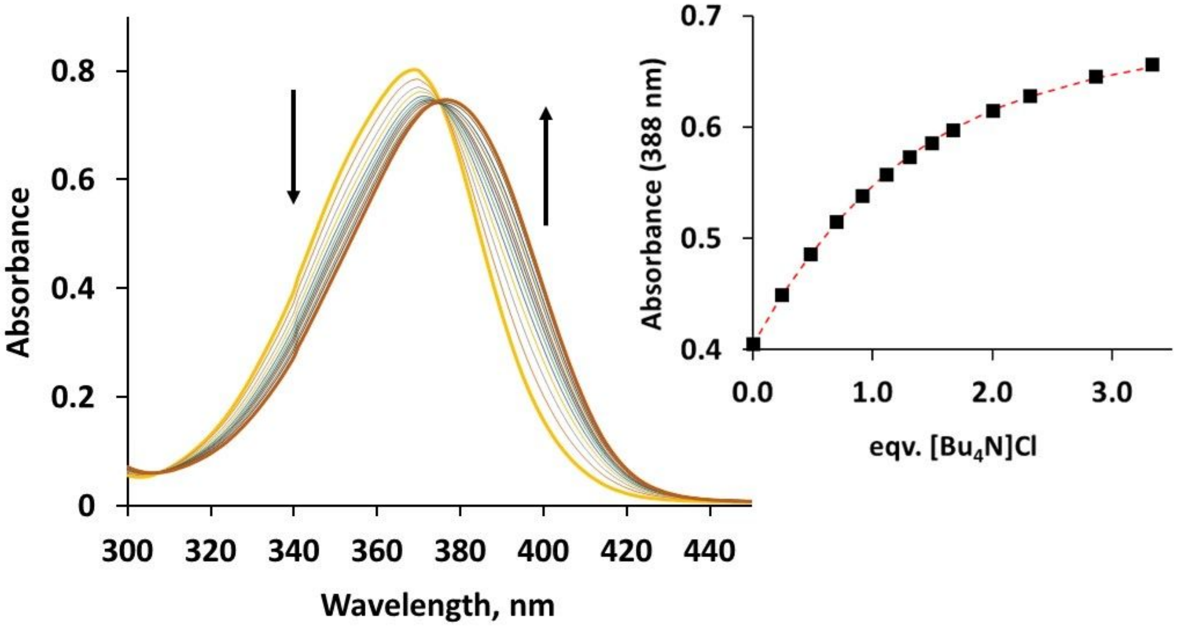

Figure S 111: UV-vis staked spectra of the $\mathbf{R_aH}\cdot 2PF_6$ (20 μM) titration with 200 μM solution of $[Bu_4N]Cl$. *Inset*: Fitting of the UV-vis titration data at 388 nm.

Concentration (µM)	Absorbance	Residual
0	0.4052	-0.00088871
4.9	0.44895	-0.00012566
9.5	0.48559	0.00130346
14	0.51463	0.00064272
18.2	0.53809	0.00034794
22.2	0.5573	0.00014656
26.1	0.57329	-0.00013432
29.8	0.58604	-0.00072056
33.3	0.59702	-0.0007362
40	0.61478	-0.00041809
46.2	0.62785	-0.00016443
57.1	0.64532	0.00026685
66.7	0.65654	0.00048042

Table S 2: Experimental data of the titration. The *p* value obtained from “runs-of-sign” was 0.05.

3.4. Cl□R_aH^{2+} study by ^{1}H-NMR.

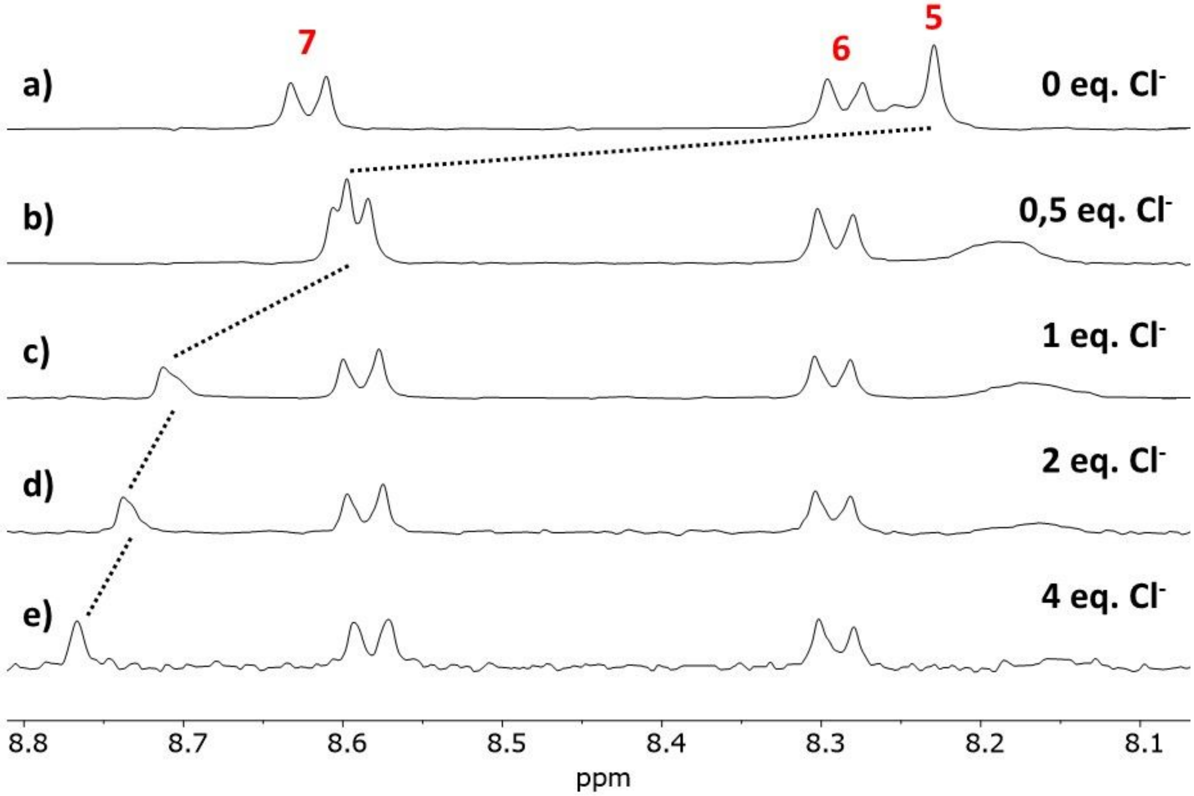

Figure S 112: ^{1}H-NMR (300 MHz, CD_3CN, 295.15 K) stacked spectra of $\mathbf{R_aH}^{2+}$ upon additions of a) 0 eq., b) 0.5 eq., c) 1 eq., d) 2 eq. and e) 4 eq. of $[Bu_4N]Cl$.

3.5. Br□R_aH^{2+} titration by UV-vis in ACN.

The mechanism proposed for the fitting process equilibria, and introduced on the software Dynafit[3] was the following:

$$\mathbf{RaH^{+2} + Br^- \Leftrightarrow Br^-{\subset}RaH^{+2}}$$

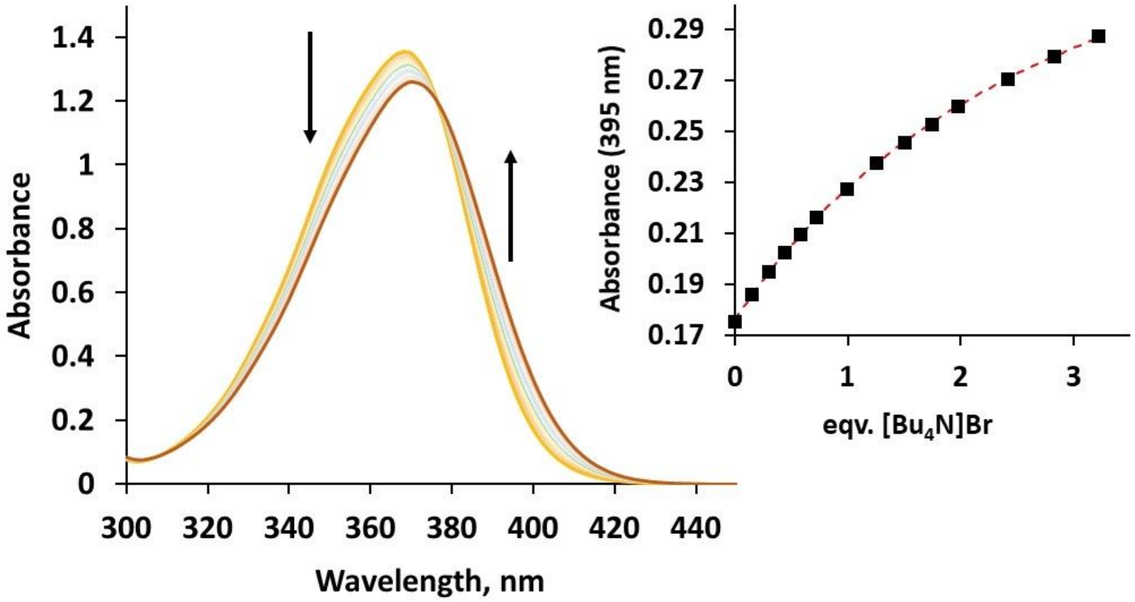

Figure S 113: UV-vis staked spectra of the $\mathbf{R_aH}{\cdot}2PF_6$ (33 μM) titration with 500 μM solution of $[Bu_4N]Br$. *Inset*: Fitting of the UV-vis titration data at 395 nm.

Concentration (µM)	Absorbance	Residual
0	0.1752	-0.00104398
4.95	0.1859	0.00027682
9.8	0.1948	0.00063625
14.56	0.2024	0.00042719
19.23	0.2093	0.00017239
23.81	0.216	0.00030370
32.71	0.2271	-0.00022078
41.28	0.2375	0.00023554
49.55	0.2456	-0.00025969
57.52	0.2528	-0.00053650
65.22	0.2596	-0.00030239
79.83	0.2706	-0.00025129
93.45	0.2795	-0.00008143
106.3	0.2874	0.00064418

Table S 3: Experimental data of the titration. The p value obtained from "runs-of-sign" was 0.3.

3.6. Br□R_aH^{2+} study by ^{1}H-NMR.

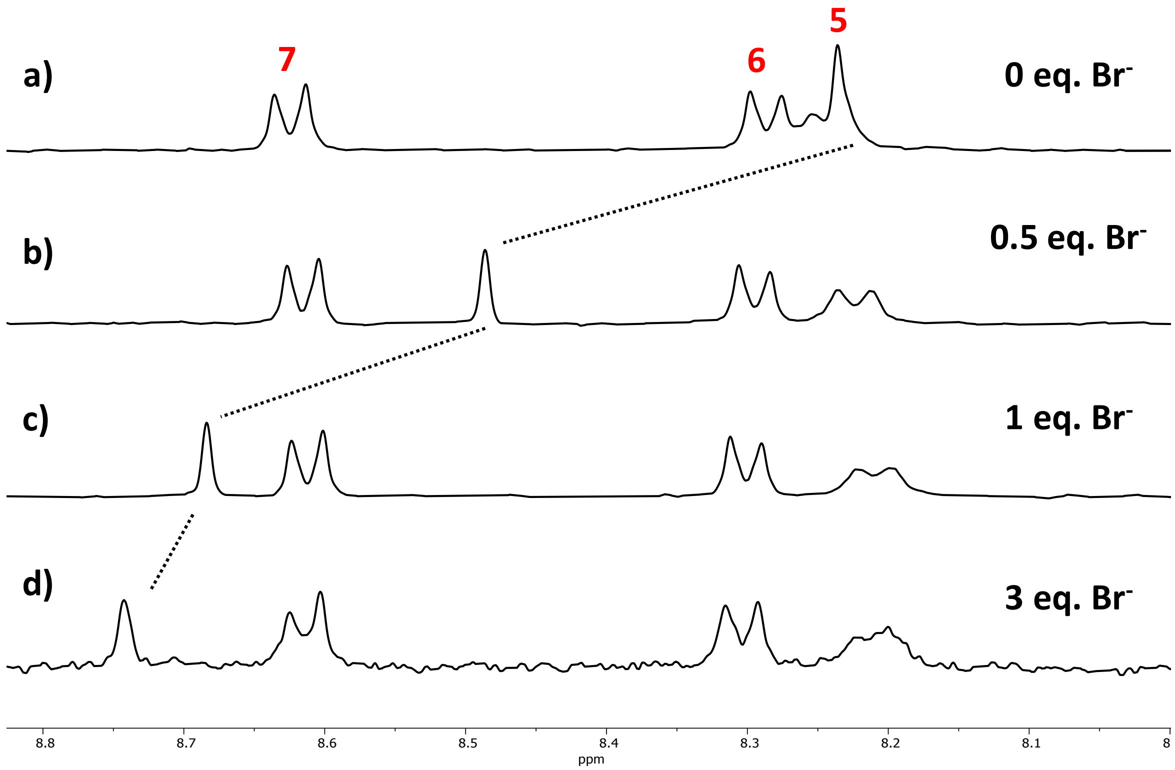

Figure S 114: ^{1}H-NMR (300 MHz, CD_3CN, 295.15 K) stacked spectra of $\mathbf{R_aH^{2+}}$ upon additions of a) 0 eq., b) 0.5 eq., c) 1 eq., and d) 3 eq. of $[Bu_4N]Br$.

4. Acid/base spectroscopy study of synthesized compounds

4.1. Spectroscopy study of R_b·2Cl in H_2O

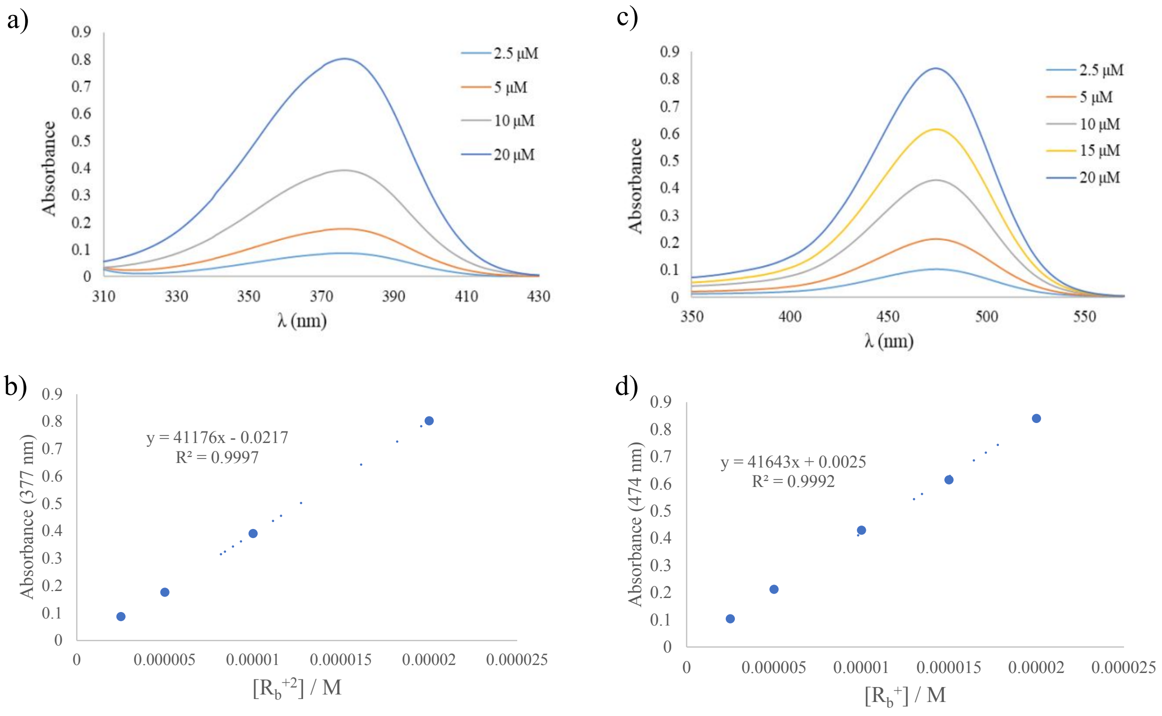

Figure S 115: a) UV-Vis spectra of $\mathbf{R_b}$·2Cl at pH 6.15 from 2.5 µM to 20 µM. b) Linear relationship between absorbance at 377 nm and concentration of $\mathbf{R_b}$·2Cl where ε = 41176 Lmol^{-1}cm^{-1}. c) UV-Vis spectra of $\mathbf{R_b}$·2Cl at pH 12 from 2.5 µM to 20 µM. d) Linear relationship between absorbance at 474 nm and concentration of $\mathbf{R_b}$·2Cl where ε = 41643 Lmol^{-1}cm^{-1}.

4.2. pK_a determination for R_b by UV-Vis

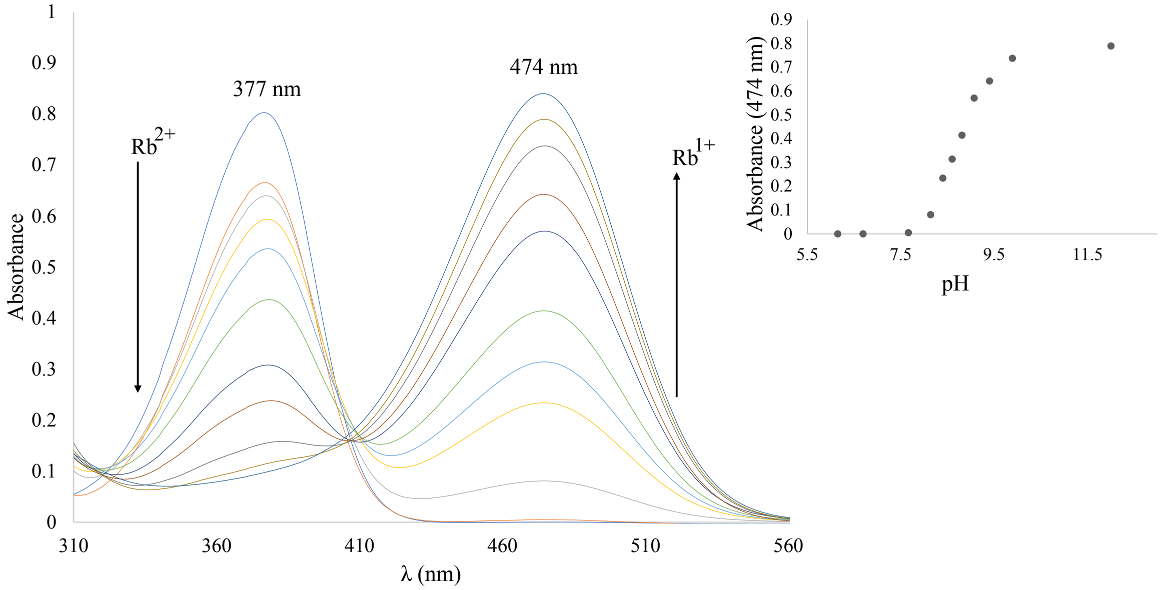

Figure S 116: UV-Vis spectra for the titration of $\mathbf{R_b}\cdot 2Cl$ at 20 μM in NaH_2PO_4/Na_2HPO_4, $KHCO_3/K_2CO_3$ and Na_2HPO_4/Na_2PO_4 buffers. On the top right: Absorption of $\mathbf{R_b}^{+}$ at λ = 474 nm plotted against pH.

pH (measured)	V (mL) of solution A	V (mL) of solution B	V (mL) of solution C	V (mL) of solution D	V (mL) of solution E	ABS (474 nm)	log[A_{474}-AR_b^{2+})/(AR_b^{1+}-A_{474})]
6.15 ($\mathbf{R_b}^{2+}$)	5.2	2.0	-	-	-	0.0001	-
6.69	3.9	4.8	-	-	-	0.0047	-2.2601
7.66	0.9	10.8	-	-	-	0.0807	-0.973
8.14	0.3	15.0	-	-	-	0.2338	-0.413
8.4	0.2	15.0	-	-	-	0.31401	-0.224
8.6	0.1	15.0	-	-	-	0.4141	-0.011
8.81	-	-	-	6.2	0.2	0.5702	0.325
9.07	-	-	-	6.0	0.6	0.6423	0.512
9.4	-	-	-	5.4	1.7	0.7371	0.856
9.89				4.2	4.2	0.7893	1.194
12 ($\mathbf{R_b}^{1+}$)	-	2.2	10.3	-	-	0.8398	-

Table S 4: Experimental data obtained for the UV-Vis titration of $\mathbf{R_b}\cdot 2Cl$ at 20 μM in NaH_2PO_4/Na_2HPO_4, $KHCO_3/K_2CO_3$ and Na_2HPO_4/Na_2PO_4 buffer. **Solution A**: 20 μM of $\mathbf{R_b}\cdot 2Cl$ and 0.05 M of NaH_2PO_4. **Solution B**: 20 μM of $\mathbf{R_b}\cdot 2Cl$ and 0.05 M of $NaHPO_4$. **Solution C**: 20 μM of $\mathbf{R_b}\cdot 2Cl$ and 0.05 M of Na_2PO_4. **Solution D**: 20 μM of $\mathbf{R_b}\cdot 2Cl$ and 0.05 M of $KHCO_3$. **Solution E**: 20 μM of $\mathbf{R_b}\cdot 2Cl$ and 0.05 M of K_2CO_3.

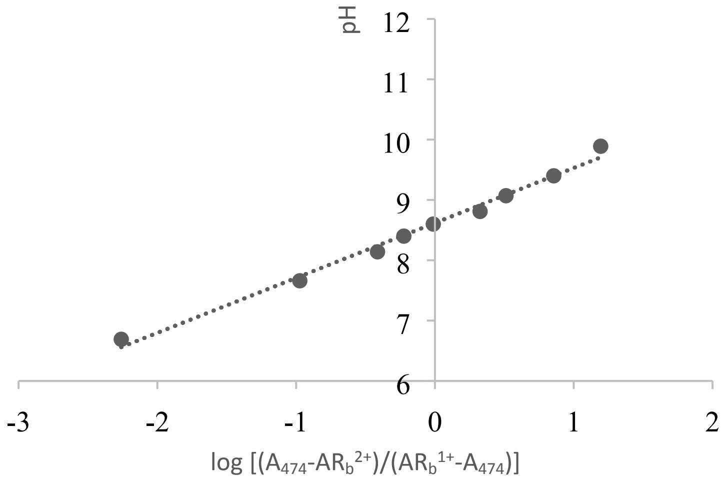

Figure S 117. Linear fitting of pH plotted against log [(A_{474}-AR_b^{2+})/(AR_b^{1+}-A_{474})], where pK_a value is 8.6.

4.3. Spectroscopy study of R_c in H_2O

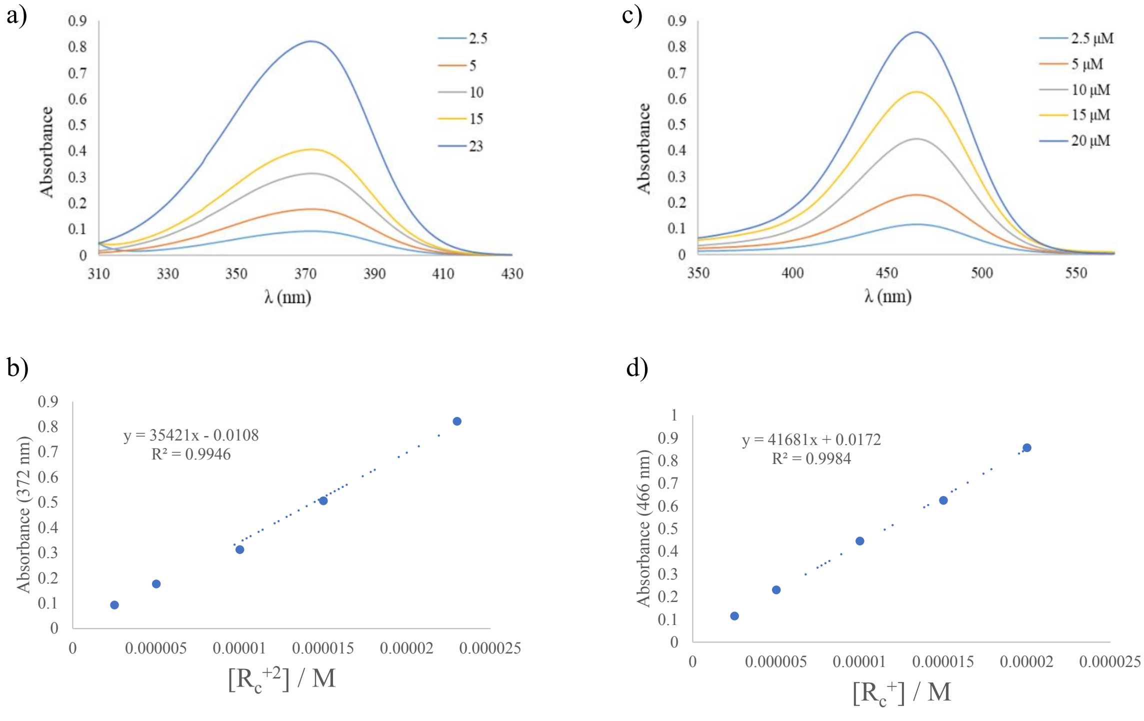

Figure S 118: a) UV-Vis spectra of $\mathbf{R_c}^{+2}$ at pH 5.68 from 2.5 µM to 20 µM. b) Linear relationship between absorbance at 372 nm and concentration of $\mathbf{R_c}^{+2}$ where ε = 35421 $Lmol^{-1}cm^{-1}$. c) UV-Vis spectra of $\mathbf{R_c}^{+}$ at pH 12 from 2.5 µM to 20 µM. d) Linear relationship between absorbance at 466 nm and concentration of $\mathbf{R_c}^{+}$ where ε = 41681 $Lmol^{-1}cm^{-1}$.

4.4. pK_a determination for R_c by UV-Vis

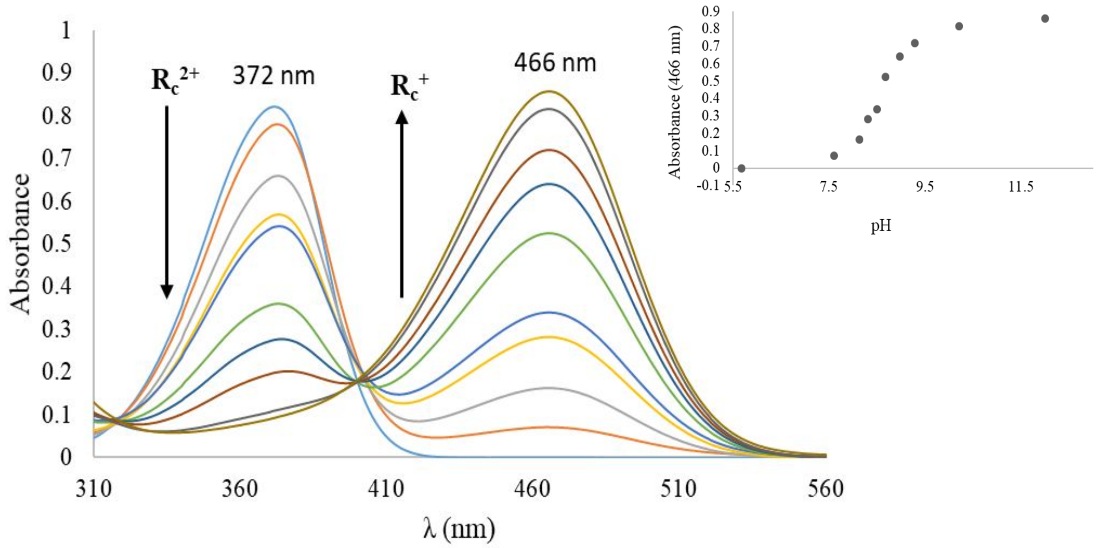

Figure S 119: UV-Vis spectra for the titration of $\mathbf{R_c}\cdot 2Cl$ at 20 µM in NaH_2PO_4/Na_2HPO_4, $KHCO_3/K_2CO_3$ and Na_2HPO_4/Na_2PO_4 buffers. On the top right: Absorption of $\mathbf{R_c}^+$ at $\lambda = 466$ nm plotted against pH.

pH (measured)	V (mL) of solution A	V (mL) of solution B	V (mL) of solution C	V (mL) of solution D	V (mL) of solution E	ABS (466 nm)	log[A_{466}-AR_c^{2+})/(AR_c^{+}-A_{466})]
5.68 ($\mathbf{R_c}^{2+}$)	5.9	0.7	-	-		-0.0011	-
7.61	0.9	10.8	-	-	-	0.0699	-1.044
8.13	0.3	15.0	-	-	-	0.1624	-0.627
8.31	0.2	15.0	-	-	-	0.2833	-0.304
8.5	0.1	15.0	-	-	-	0.3395	-0.181
8.68	-	-	-	6.8	-	0.5239	0.197
8.97	-	-	-	6.2	0.2	0.6399	0.469
9.29	-	-	-	6.0	0.6	0.71905	0.716
10.21	-	-	-	4.2	4.2	0.8153	1.294
12 ($\mathbf{R_c}^+$)	-	2.2	10.3	-	-	0.85735	-

Table S 5: Experimental data obtained for the UV-Vis titration of $\mathbf{R_c}\cdot 2Cl$ at 20 µM in NaH_2PO_4/Na_2HPO_4, $KHCO_3/K_2CO_3$ and Na_2HPO_4/Na_2PO_4 buffer. **Solution A**: 20 µM of **Rc**·2Cl and 0.05 M of NaH_2PO_4. **Solution B**: 20 µM of $\mathbf{R_c}\cdot 2Cl$ and 0.05 M of $NaHPO_4$. **Solution C**: 20 µM of $\mathbf{R_c}\cdot 2Cl$ and 0.05 M of Na_2PO_4. **Solution D**: 20 µM of $\mathbf{R_c}\cdot 2Cl$ and 0.05 M of $KHCO_3$. **Solution E**: 20 µM of $\mathbf{R_c}\cdot 2Cl$ and 0.05 M of K_2CO_3.

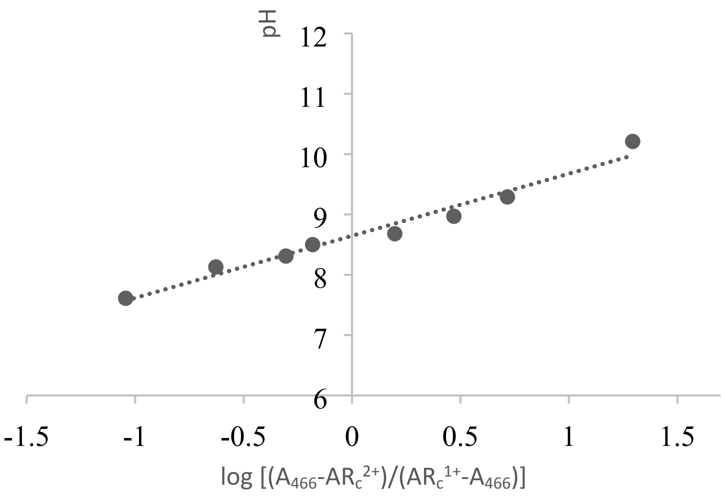

Figure S 120: Linear fitting of pH plotted against log $[(A_{466}\text{-}AR_c^{2+})/(AR_c^{1+}\text{-}A_{466})]$, where p$K_a$ value is 8.6.

4.5. Spectroscopy study of R_d in H_2O

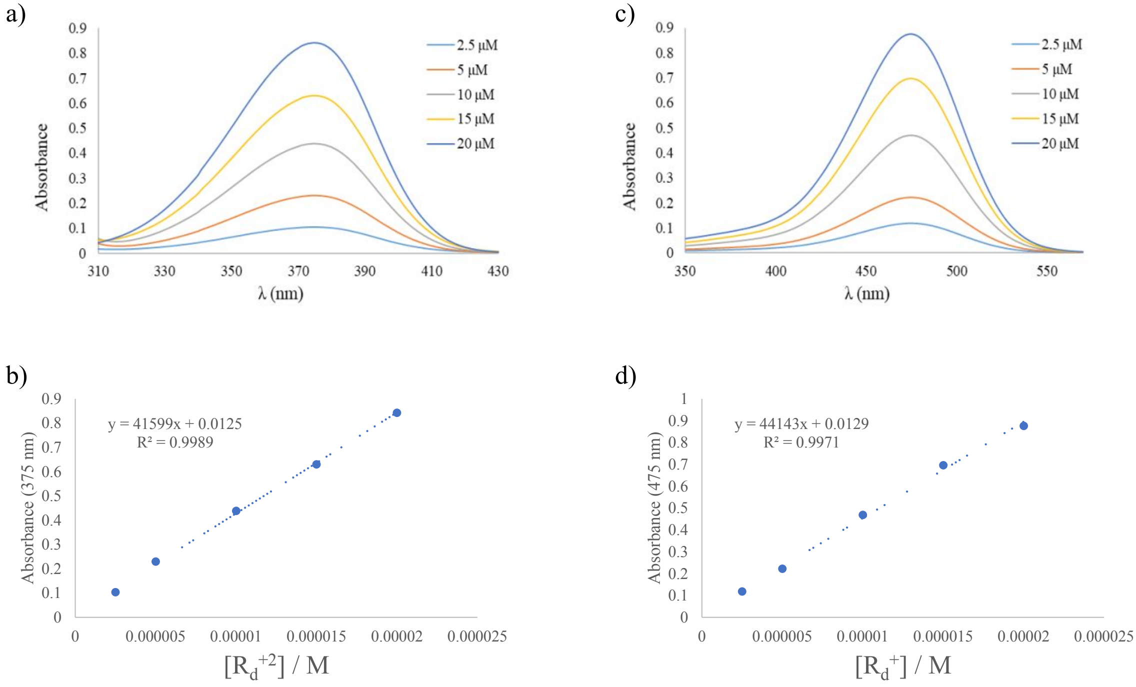

Figure S 121: a) UV-Vis spectra of $\mathbf{R_d}^{+2}$ at pH 5.69 from 2.5 µM to 20 µM. b) Linear relationship between absorbance at 375 nm and concentration of $\mathbf{R_d}^{+2}$ where ε = 41599 Lmol^{-1}cm^{-1}. c) UV-Vis spectra of $\mathbf{R_d}^{+}$ at pH 12.01 from 2.5 µM to 20 µM. d) Linear relationship between absorbance at 475 nm and concentration of $\mathbf{R_d}^{+}$ where ε = 44143 Lmol^{-1}cm^{-1}.

4.6. pK_a determination for R_d by UV-Vis

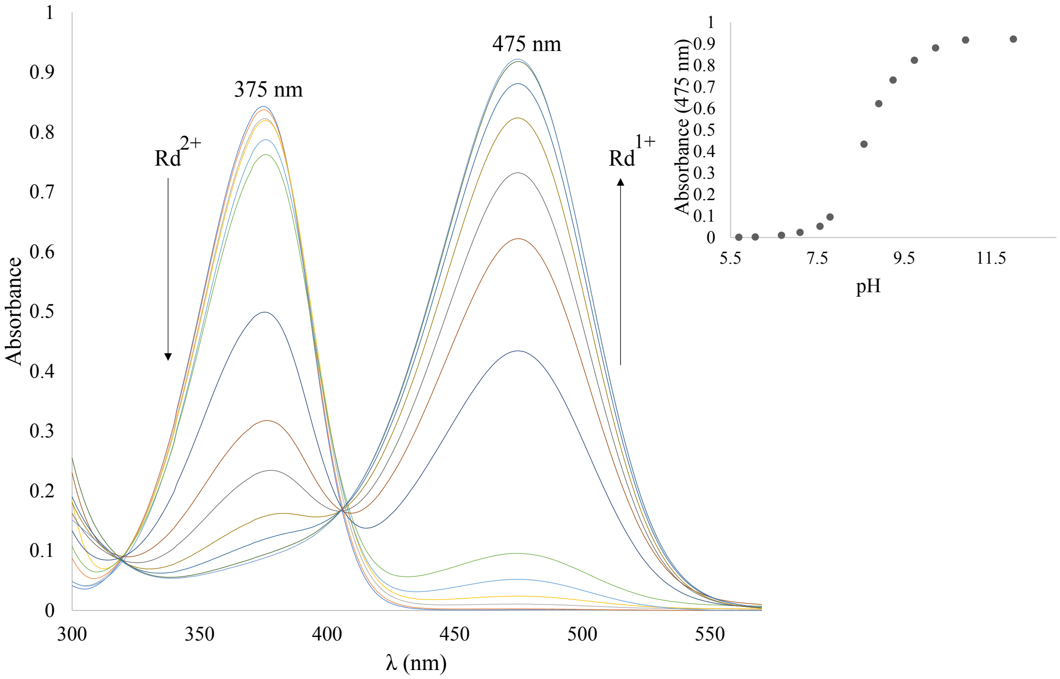

Figure S 122: UV-Vis spectra for the titration of $\mathbf{R_d}$·2Cl at 20 µM in NaH_2PO_4/Na_2HPO_4, $KHCO_3/K_2CO_3$ and Na_2HPO_4/Na_2PO_4 buffers. On the top right: Absorption of $\mathbf{R_d}^+$ at λ = 475 nm plotted against pH.

pH (measured)	V (mL) of solution A	V (mL) of solution B	V (mL) of solution C	V (mL) of solution D	V (mL) of solution E	ABS (475 nm)	$\log[A_{475}-AR_d^{2+})/(AR_d^{1+}-A_{475})]$
5.69 ($\mathbf{R_d}^{2+}$)	5.9	0.7	-	-		0.0013	-
6.07	5.2	2.0	-	-	-	0.0027	-2.917
6.67	3.9	4.8	-	-	-	0.0106	-1.991
7.1	2.1	8.3	-	-	-	0.0239	-1.598
7.56	0.9	10.8	-	-	-	0.0519	-1.234
7.79	0.5	15.0	-	-	-	0.0953	-0.944
8.57	-	-	-	6.6	-	0.4339	-0.052
8.91	-	-	-	6.2	0.2	0.6217	0.315
9.24	-	-	-	6.0	0.6	0.7318	0.584
9.73	-	-	-	5.4	1.7	0.8235	0.922
10.22	-	-	-	4.2	4.2	0.8809	1.332
10.91	-	-	-	1.0	10.4	0.9179	2.371
12.01 ($\mathbf{R_d}^{+}$)	-	2.2	10.3	-	-	0.9218	-

Table S 6: Experimental data obtained for the UV-Vis titration of $\mathbf{R_d}$·2Cl at 20 µM in NaH_2PO_4/Na_2HPO_4, $KHCO_3/K_2CO_3$ and Na_2HPO_4/Na_2PO_4 buffer. **Solution A**: 20 µM of $\mathbf{R_d}$·2Cl and 0.05 M of NaH_2PO_4. **Solution B**: 20 µM of $\mathbf{R_d}$·2Cl and 0.05 M of $NaHPO_4$. **Solution C**: 20 µM of $\mathbf{R_d}$·2Cl and 0.05 M of Na_2PO_4. **Solution D**: 20 µM of $\mathbf{R_d}$·2Cl and 0.05 M of $KHCO_3$. **Solution E**: 20 µM of $\mathbf{R_d}$·2Cl and 0.05 M of K_2CO_3.

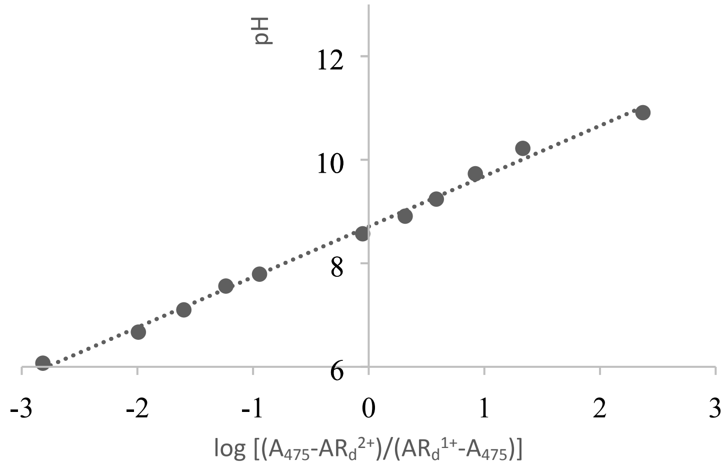

Figure S 123: Linear fitting of pH plotted against log [(A_{475}-AR_d^{2+})/(AR_d^{+}-A_{475})], where pK_a value is 8.7.

4.7. Spectroscopy study of R_e in H_2O

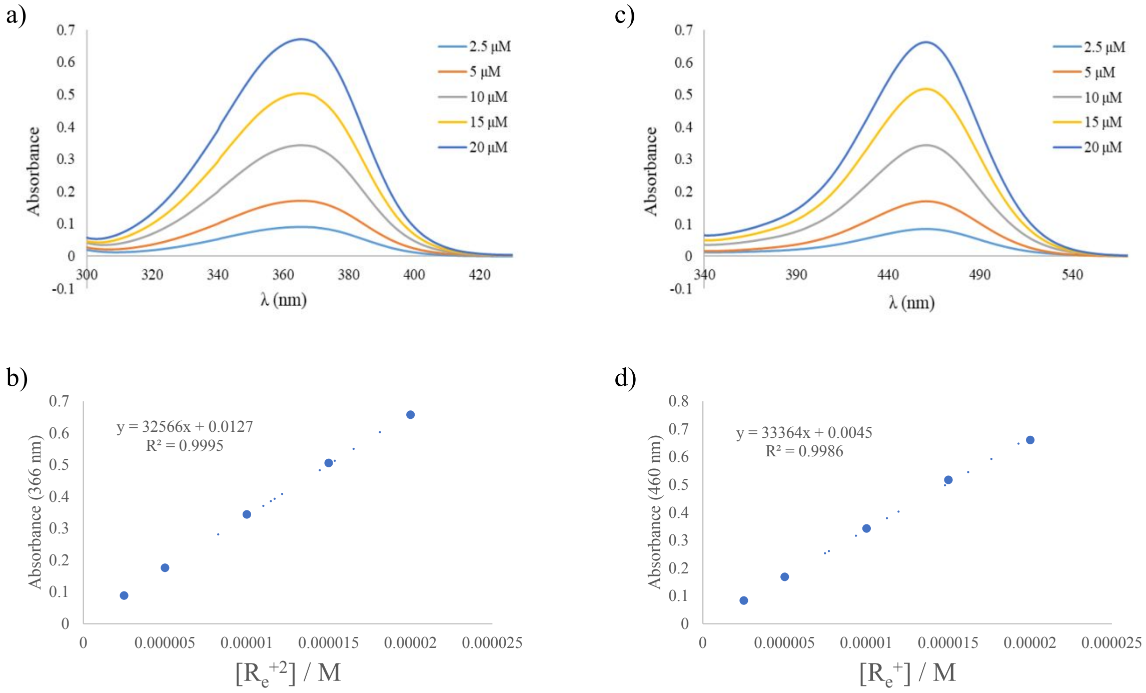

Figure S 124: a) UV-Vis spectra of $\mathbf{R_e}^{+2}$ at pH 6.96 from 2.5 µM to 20 µM. b) Linear relationship between absorbance at 366 nm and concentration of $\mathbf{R_e}^{+2}$ where ε = 32566 Lmol^{-1}cm^{-1}. c) UV-Vis spectra of $\mathbf{R_e}^{+}$ at pH 11.9 from 2.5 µM to 20 µM. d) Linear relationship between absorbance at 460 nm and concentration of $\mathbf{R_e}^{+}$ where ε = 33364 Lmol^{-1}cm^{-1}.

4.8. pK_a determination for R_e by UV-Vis

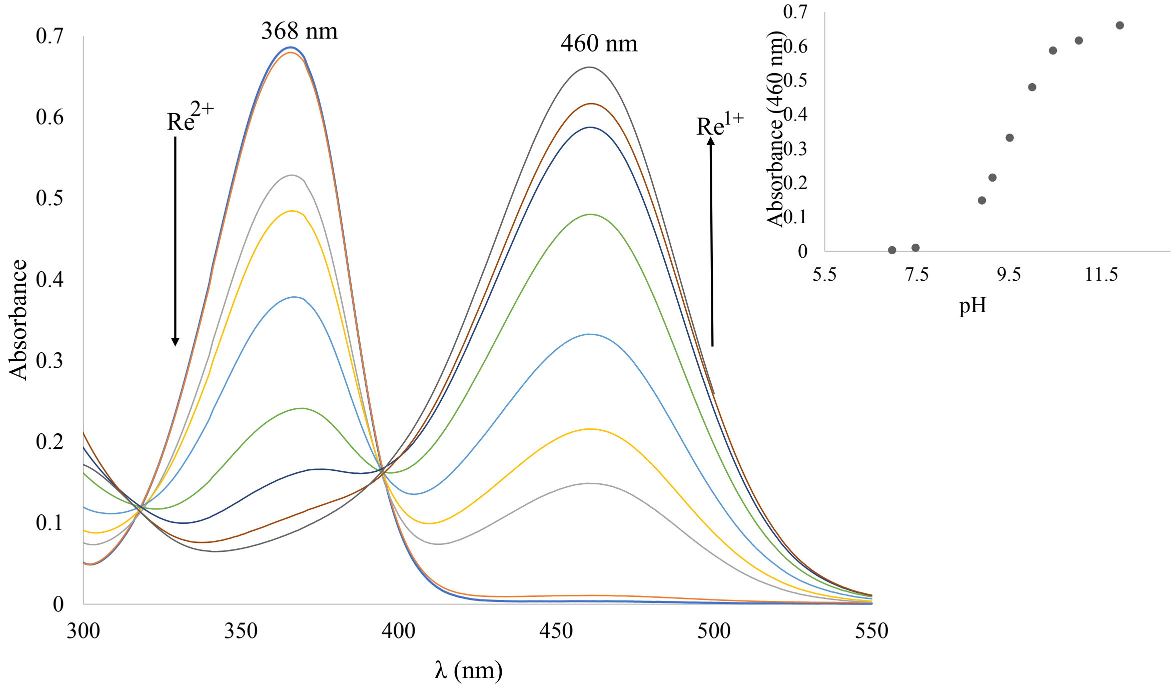

Figure S 125: UV-Vis spectra for the titration of $\mathbf{R_e}$·2Cl at 20 µM in NaH_2PO_4/Na_2HPO_4, $KHCO_3/K_2CO_3$ and Na_2HPO_4/Na_2PO_4 buffers. On the top right: Absorption of $\mathbf{R_e}^+$ at λ = 460 nm plotted against pH.

pH (measured)	V (mL) of solution A	V (mL) of solution B	V (mL) of solution C	V (mL) of solution D	V (mL) of solution E	ABS (460 nm)	log[A_{460}-AR_e^{2+})/(AR_e^{1+}-A_{460})]
6.96 (**R_e^{2+}**)	2.1	8.3	-	-	-	0.0036	-
7.47	0.9	10.8	-	-	-	0.0107	-1.962
8.91	-	-	-	6.1	0.2	0.1488	-0.547
9.14	-	-	-	5.9	0.6	0.2157	-0.322
9.51	-	-	-	5.4	1.7	0.3324	0.0004
10	-	-	-	4.1	4.1	0.4801	0.420
10.45	-	-	-	2.4	7.6	0.5873	0.899
11.01	-	-	-	1.0	10.4	0.6165	1.139
11.9 (**R_e^{1+}**)	-	8.5	4.0	-	-	0.6609	-

Table S 7: Experimental data obtained for the UV-Vis titration of $\mathbf{R_e}$·2Cl at 20 µM in NaH_2PO_4/Na_2HPO_4, $KHCO_3/K_2CO_3$ and Na_2HPO_4/Na_2PO_4 buffer. **Solution A**: 20 µM of $\mathbf{R_e}$·2Cl and 0.05 M of NaH_2PO_4. **Solution B**: 20 µM of $\mathbf{R_e}$·2Cl and 0.05 M of $NaHPO_4$. **Solution C**: 20 µM of $\mathbf{R_e}$·2Cl and 0.05 M of Na_2PO_4. **Solution D**: 20 µM of $\mathbf{R_e}$·2Cl and 0.05 M of $KHCO_3$. **Solution E**: 20 µM of $\mathbf{R_e}$·2Cl and 0.05 M of K_2CO_3.

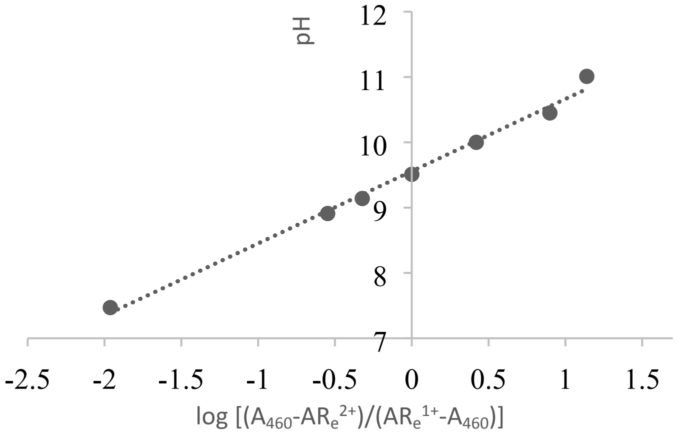

Figure S 126: Linear fitting of pH plotted against log [(A_{460}-AR_e^{2+})/(AR_e^{1+}-A_{460})], where pK_a value is 9.5.

4.9. Spectroscopy study of M_a in H_2O

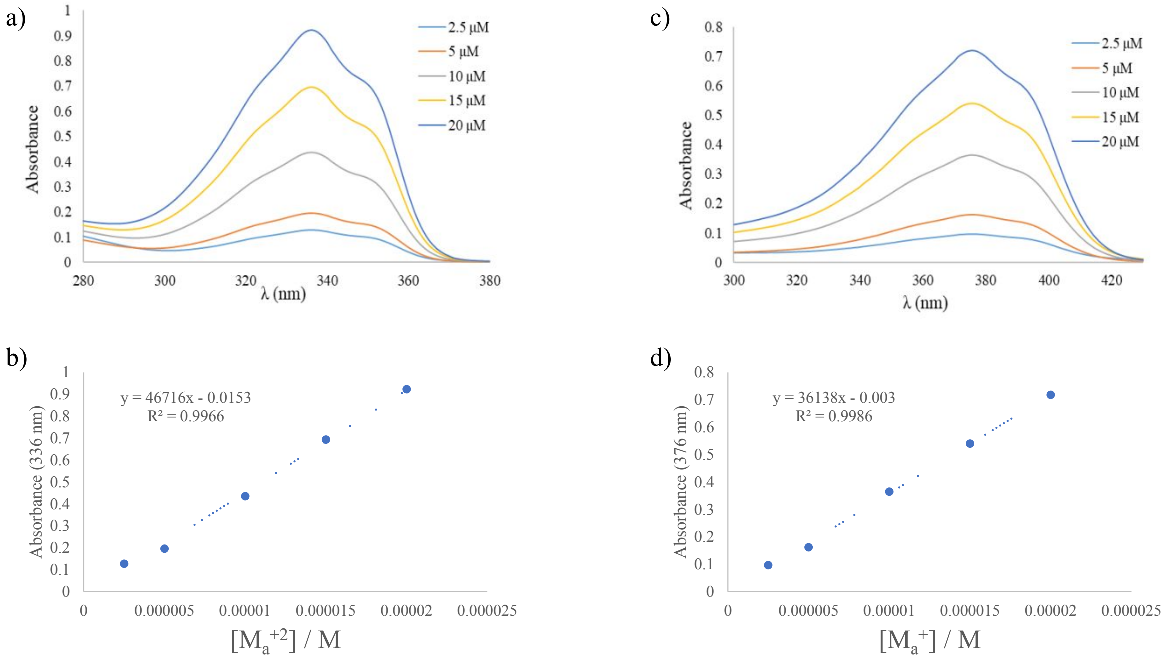

Figure S 127: a) UV-Vis spectra of $\mathbf{M_a}^{+2}$ at pH 6.57 from 2.5 µM to 20 µM. b) Linear relationship between absorbance at 338 nm and concentration of $\mathbf{M_a}^{+2}$ where ε = 46716 $Lmol^{-1}cm^{-1}$. c) UV-Vis spectra of $\mathbf{M_a}^{+}$ at pH 12.28 from 2.5 µM to 20 µM. d) Linear relationship between absorbance at 376 nm and concentration of $\mathbf{M_a}^{+}$ where ε = 36138 $Lmol^{-1}cm^{-1}$.

4.10. pK_a determination for M_a by UV-Vis

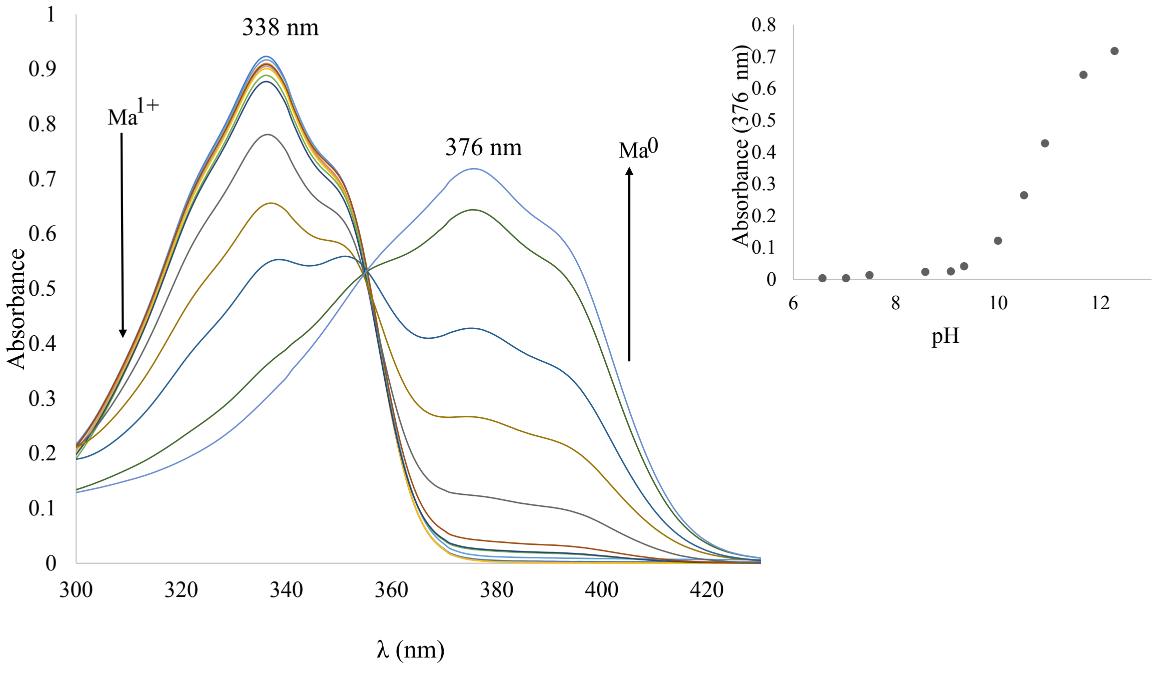

Figure S 128: UV-Vis spectra for the titration of $\mathbf{M_a}$·Cl at 20 µM in NaH_2PO_4/Na_2HPO_4, $KHCO_3/K_2CO_3$ and Na_2HPO_4/Na2PO4 buffers. On the top right: absorption of $\mathbf{M_a}^+$ at λ = 376 nm plotted against pH.

pH (measured)	V (mL) of solution A	V (mL) of solution B	V (mL) of solution C	V (mL) of solution D	V (mL) of solution E	ABS (376 nm)	log[A_{376}-AMa^{1+})/(AMa^0-A_{376})]
6.57 (**M_a $^{1+}$**)	3.8	4.8	-	-	-	0.0043	-
7.03	2.1	8.3	-	-	-	0.0044	-3.908
9.08	-	-	-	6.0	0.6	0.0257	-1.509
9.34	-	-	-	5.4	1.7	0.0417	-1.257
10	-	-	-	4.2	4.2	0.12208	-0.704
10.51	-	-	-	2.4	7.7	0.2649	-0.2406
10.92				1.0	10.4	0.42809	0.1641
11.67		8.5	4.0			0.6435	0.931
12.28 (**M_a^0**)	-	2.2	10.3	-	-	0.7184	-

Table S 8: Experimental data obtained for the UV-Vis titration of $\mathbf{M_a}$·Cl at 20 µM in NaH_2PO_4/Na_2HPO_4, $KHCO_3/K_2CO_3$ and Na_2HPO_4/Na_2PO_4 buffer. **Solution A**: 20 µM of $\mathbf{M_a}$·Cl and 0.05 M of NaH_2PO_4. **Solution B**: 20 µM of $\mathbf{M_a}$·Cl and 0.05 M of $NaHPO_4$. **Solution C**: 20 µM of $\mathbf{M_a}$·Cl and 0.05 M of Na_2PO_4. **Solution D**: 20 µM of $\mathbf{M_a}$·Cl and 0.05 M of $KHCO_3$. **Solution E**: 20 µM of $\mathbf{M_a}$·Cl and 0.05 M of K_2CO_3.

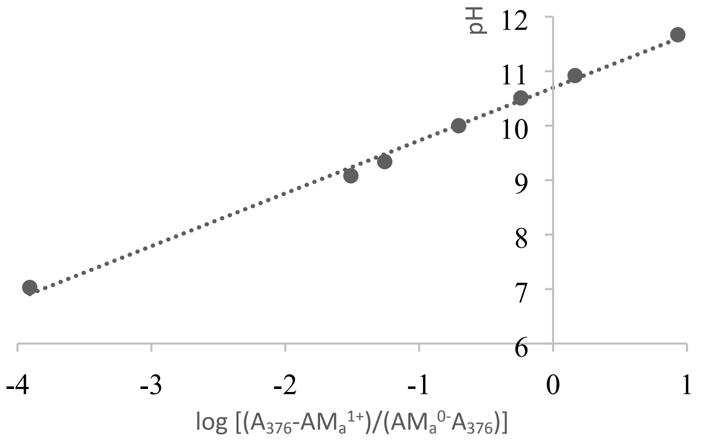

Figure S 129: Linear fitting of pH plotted against log [(A_{376}-AM_a^{1+})/(AM_a^{0}-A_{376})], where pK_a value is 10.6.

4.11. pK_a determination for M_b by UV-Vis

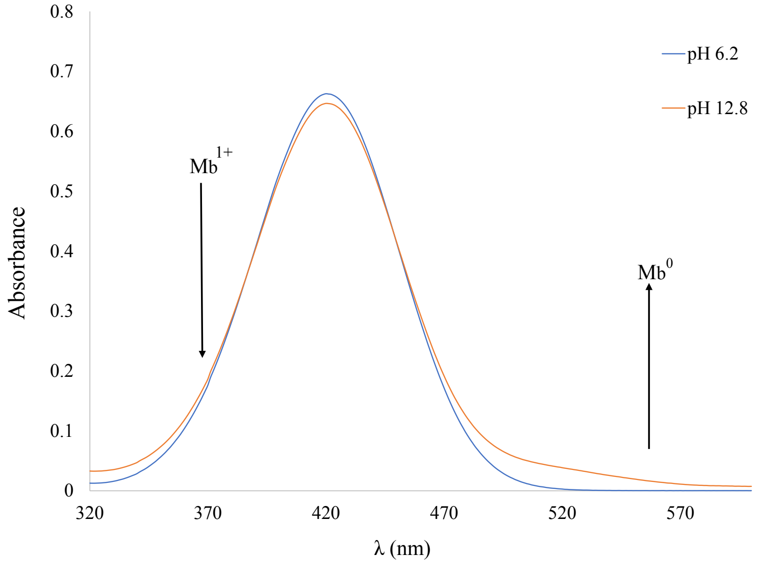

Figure S 130: UV-Vis spectra for the titration of $\mathbf{M_b}$·Cl at 20 μM in NaH_2PO_4/Na_2HPO_4 and Na_2HPO_4/Na_2PO_4 buffers; pH range from 6.2 to 12.8.

**pH (measured)**	**V (mL) of solution A**	**V (mL) of solution B**	**V (mL) of solution C**	**ABS (X nm)**	**log[Ax-AM_b^{1+})/(AM_b^{0}-Ax)]**
6.26 (**M_b $^{1+}$**)	5.2	2	-	-	-
12.8 (**M_b^{0}**)	-	2.2	10.3	-	-

Table S 9: Experimental data obtained for the UV-Vis titration of $\mathbf{M_b}$ Cl at 20 μM in NaH_2PO_4/Na_2HPO_4, $KHCO_3$/K_2CO_3 and Na_2HPO_4/Na_2PO_4 buffer. **Solution A**: 20 μM of $\mathbf{M_b}$·Cl and 0.05 M of NaH_2PO_4. **Solution B**: 20 μM of $\mathbf{M_b}$·Cl and 0.05 M of $NaHPO_4$. **Solution C**: 20 μM of $\mathbf{M_b}$·Cl and 0.05 M of Na_2PO_4.

5. Determination of rotational energy barrier ($\Delta G^{\neq}$)

The coalescence temperature (T_c) could be estimated for different protons on VT NMR experiments. Equation (1) provides the information of the energy of the rotational barrier in association with the maximum peak separation (**Δν** in Hz) at slow exchange between the peaks.[5]

$$\Delta G^{\neq} = 4.57\square 10^{-3} \boldsymbol{T_c}(9.972 + \log \boldsymbol{T_c}/\Delta\nu) \quad (1)$$

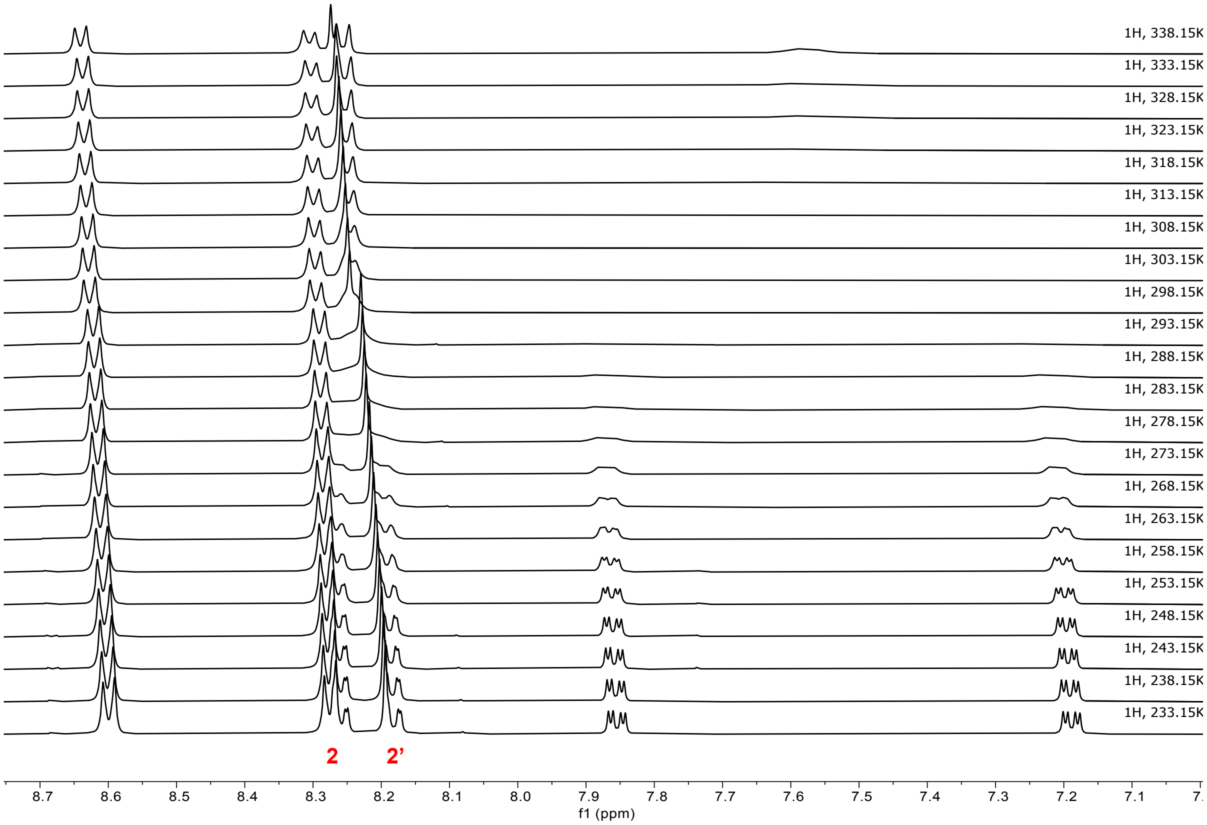

Figure S 131: VT ^{1}H NMR (500 MHz, CD_3CN) stacked spectra for $\mathbf{R_a}^{+2}$.

Signal	$\Delta\nu_c$ (Hz)	T_c (K)	$\Delta G^{\neq}$ (kcal mol^{-1})
2 – 2'	31.16	283.15	14.14

Table S 10: Experimental data attained for the calculation of $\Delta G^{\neq}$ via coalescence temperatures of distinct signals of the VT ^{1}H NMR of $\mathbf{R_a}^{+2}$ in CD_3CN.

[5] a) Sandstrom, J. Dynamic NMR Spectroscopy; Academic Press: New York, NY, USA, **1983**. b) Kessler, H. Detection of Hindered Rotation and Inversion by NMR Spectroscopy. *Angew. Chem. Int. Ed.* **1970**, *9*, 219-235.

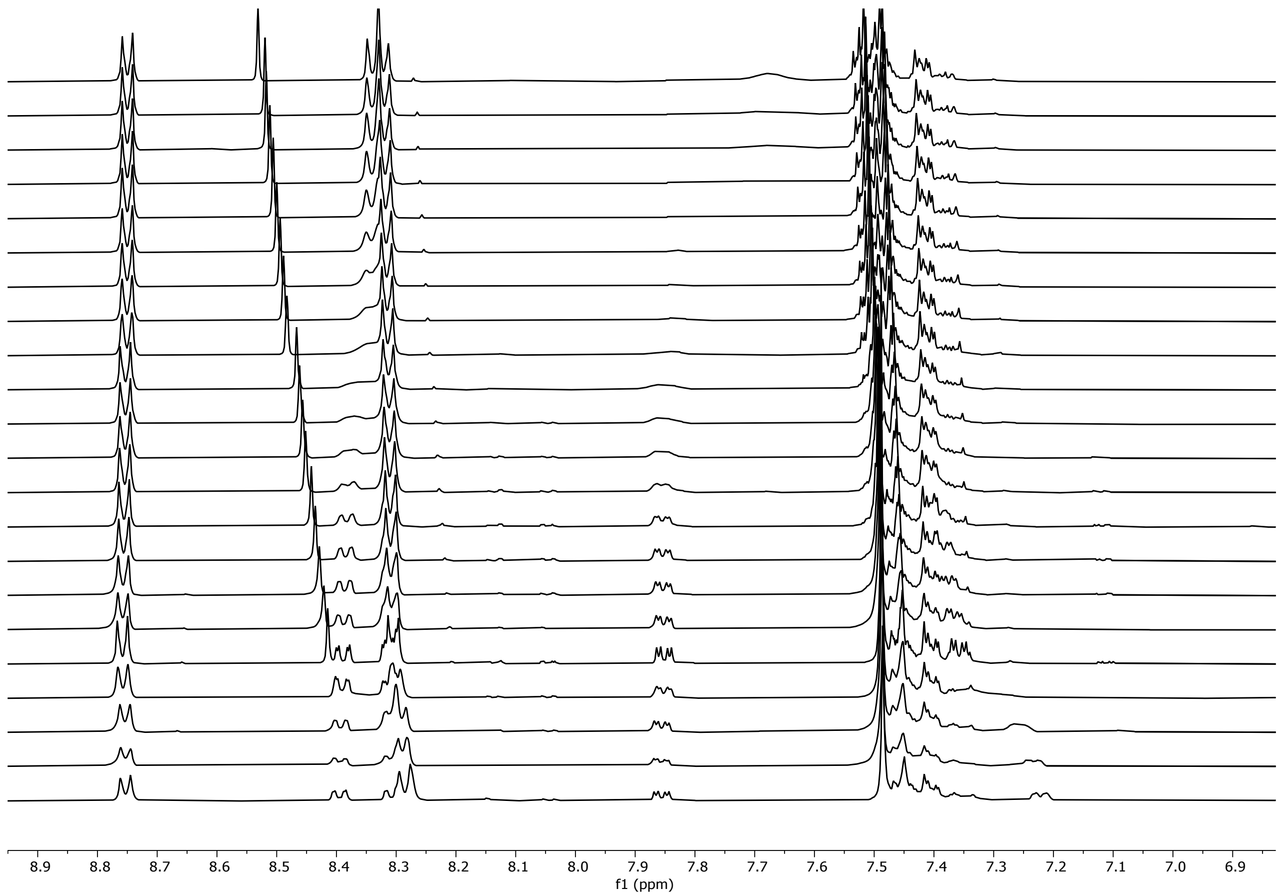

Figure S 132: VT ^{1}H NMR (500 MHz, CD_3CN) stacked spectra for $\mathbf{R_b}^{+2}$.

Signal	$\Delta\nu_c$ (Hz)	T_c (K)	$\Delta G^{\neq}$ (kcal mol^{-1})
2 – 2’	35.37	283.15	14.07

Table S 11: Experimental data attained for the calculation of $\Delta G^{\neq}$ via coalescence temperatures of distinct signals of the VT ^{1}H NMR of $\mathbf{R_b}^{+2}$ in CD_3CN.

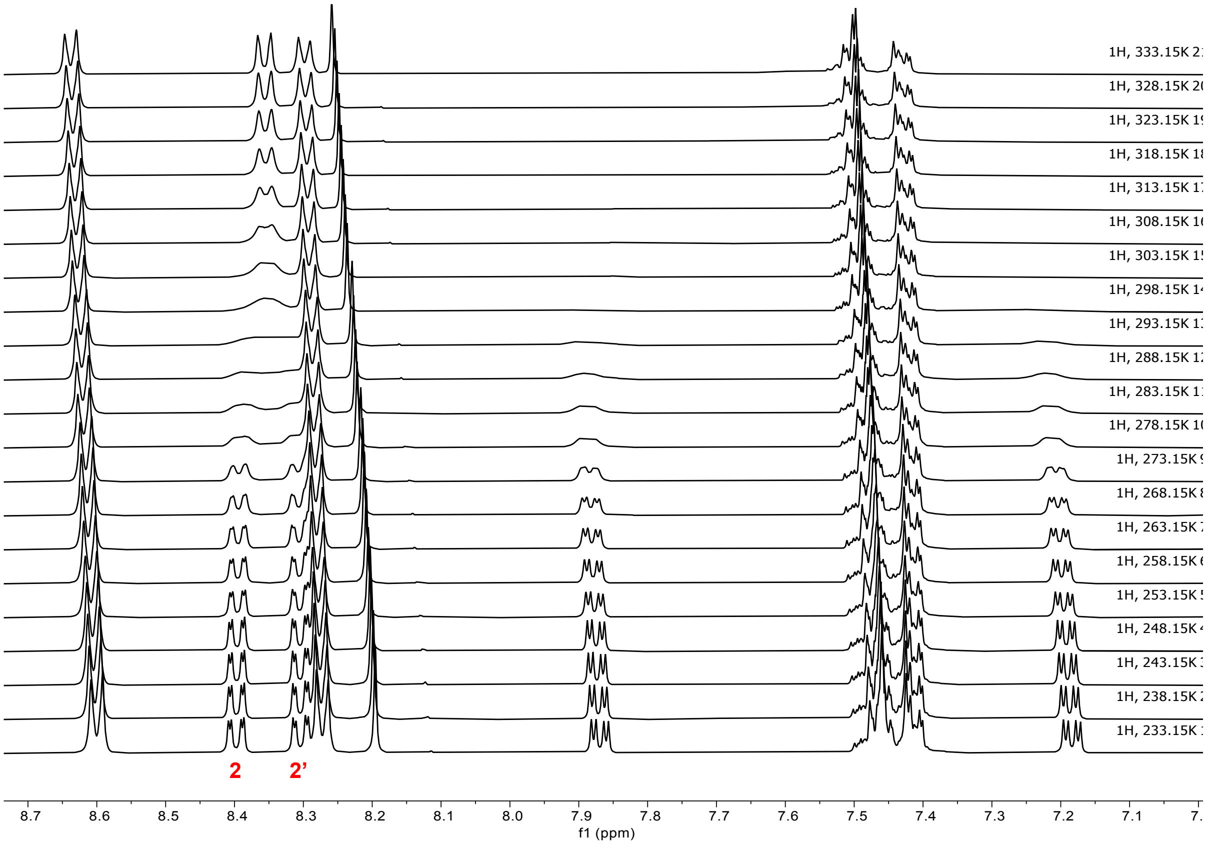

Figure S 133: VT 1H NMR (500 MHz, CD_3CN) stacked spectra for $\mathbf{R_c}^{+2}$.

Signal	$\Delta\nu_c$ (Hz)	T_c (K)	$\Delta G^{\neq}$ (kcal mol^{-1})
2 – 2’	37.67	293.15	14.55

Table S 12: Experimental data attained for the calculation of $\Delta G^{\neq}$ via coalescence temperatures of distinct signals of the VT ^{1}H NMR of $\mathbf{R_c}^{+2}$ in CD_3CN.

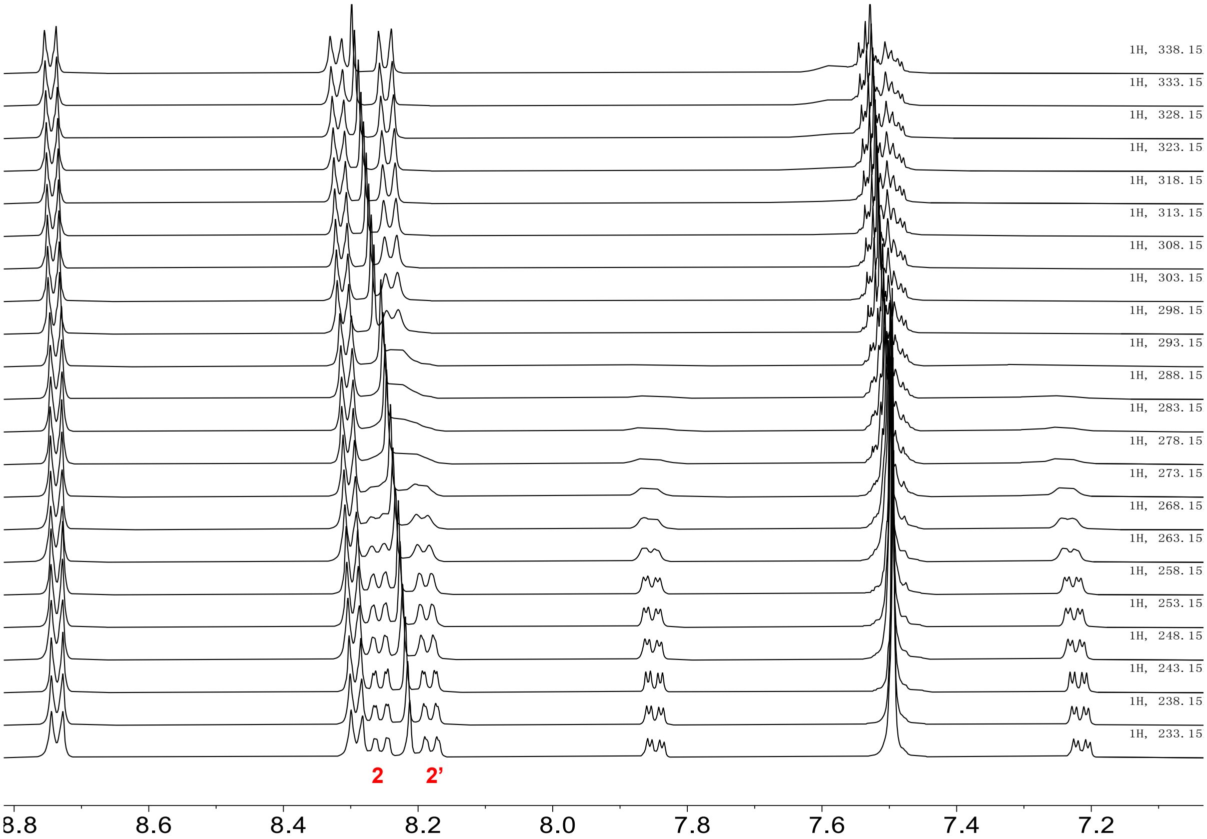

Figure S 134: VT ^{1}H NMR (500 MHz, CD_3CN) stacked spectra for $\mathbf{R_d}^{+2}$.

**Signal**	**$\Delta\nu_c$ (Hz)**	**T_c (K)**	**$\Delta G^{\neq}$ (kcal mol^{-1})**
2 – 2’	30.09	283.15	14.15

Table S 13: Experimental data attained for the calculation of $\Delta G^{\neq}$ via coalescence temperatures of distinct signals of the VT ^{1}H NMR of $\mathbf{R_d}^{+2}$ in CD_3CN.

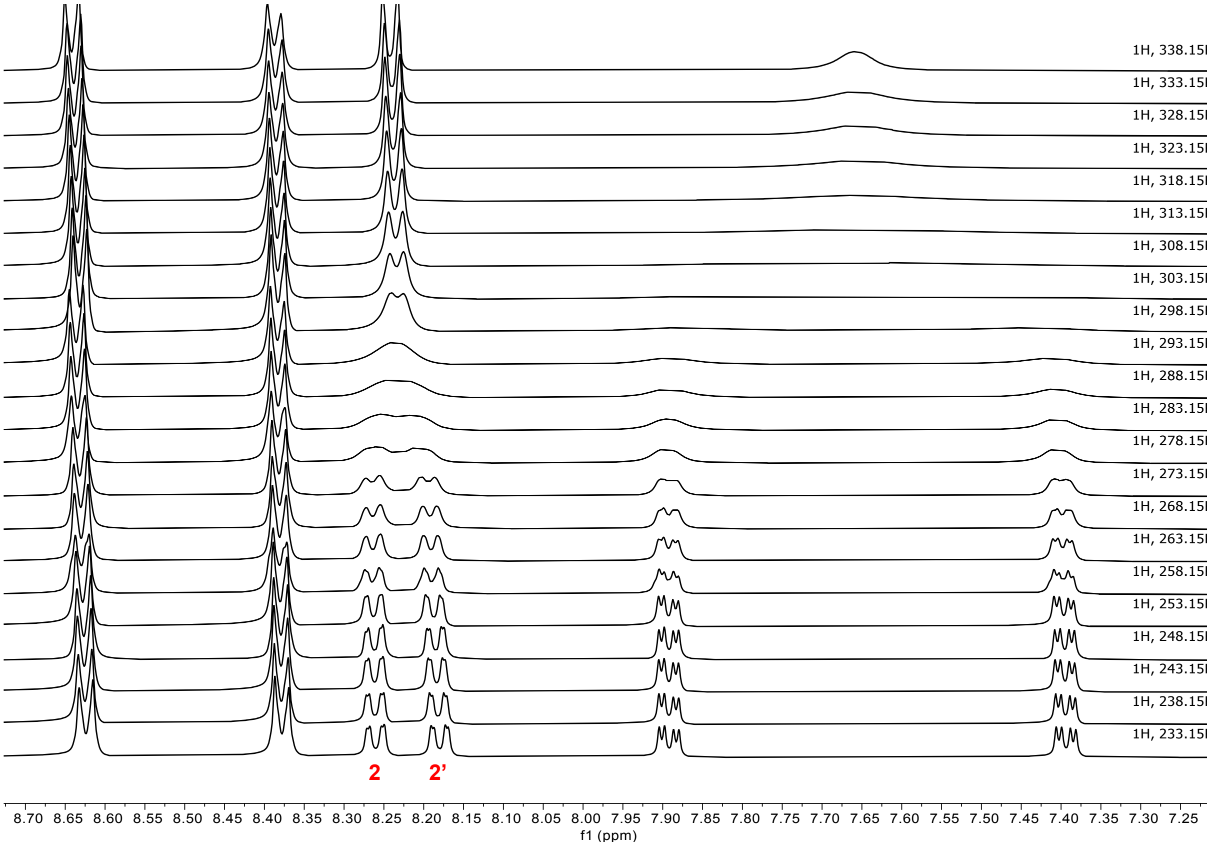

Figure S 135: VT ^{1}H NMR (500 MHz, CD_3CN) stacked spectra for $\mathbf{R_e}^{+2}$.

**Signal**	**$\Delta\nu_c$ (Hz)**	**T_c (K)**	**$\Delta G^{\neq}$ (kcal mol^{-1})**
2 – 2’	32.71	288.15	14.37

Table S 14: Experimental data attained for the calculation of $\Delta G^{\neq}$ via coalescence temperatures of distinct signals of the VT ^{1}H NMR of $\mathbf{R_e}^{+2}$ in CD_3CN.

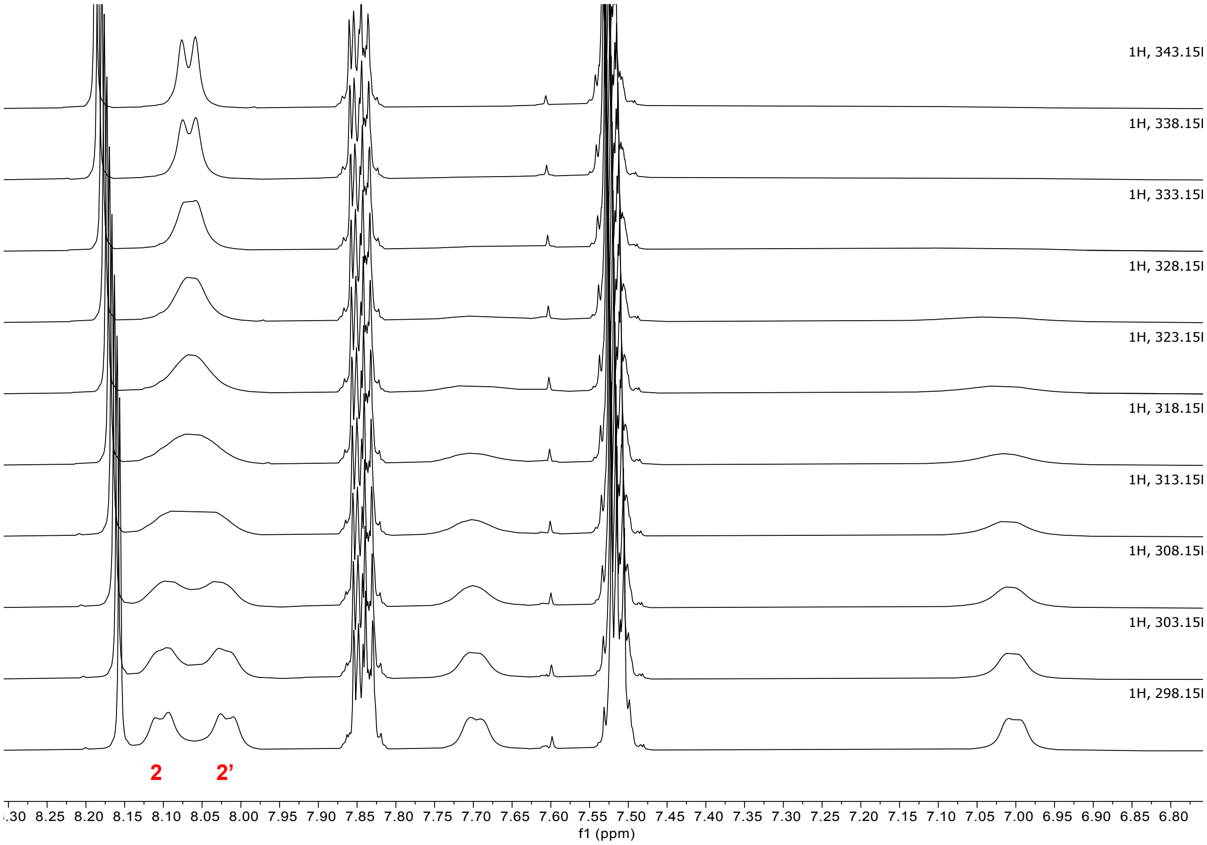

Figure S 136: VT ^{1}H NMR (500 MHz, D_2O) stacked spectra for $\mathbf{M_a}^{+2}$.

Signal	$\Delta\nu_c$ (Hz)	T_c (K)	$\Delta G^{\neq}$ (kcal mol^{-1})
2 – 2’	33.55	318.15	15.91

Table S 15: Experimental data attained for the calculation of $\Delta G^{\neq}$ via coalescence temperatures of distinct signals of the VT ^{1}H NMR of $\mathbf{M_a}^{+2}$ in D_2O.

6. Cyclic voltammetry studies of $\mathbf{R_aH{\cdot}2Cl}$ in water at different pH.

Cyclic voltammetry (CV) experiments were performed using a glassy carbon working electrode, the counter electrode was a Pt coil and the reference electrode was Ag/AgCl. For each pH, $\mathbf{R_a}H{\cdot}2Cl$ (2 mM) was dissolved in an 0.1 M NaCl and 0.05 M aqueous solution of the corresponding buffer (pH = 2, H_3PO_4/NaH_2PO_4; pH 7, NaH_2PO_4/Na_2HPO4; pH 12 Na_2HPO_4/Na_3PO_4). Before each measurement, the aqueous solution was degassed with Ar for 10s. The scan rate was 100 mV/s.

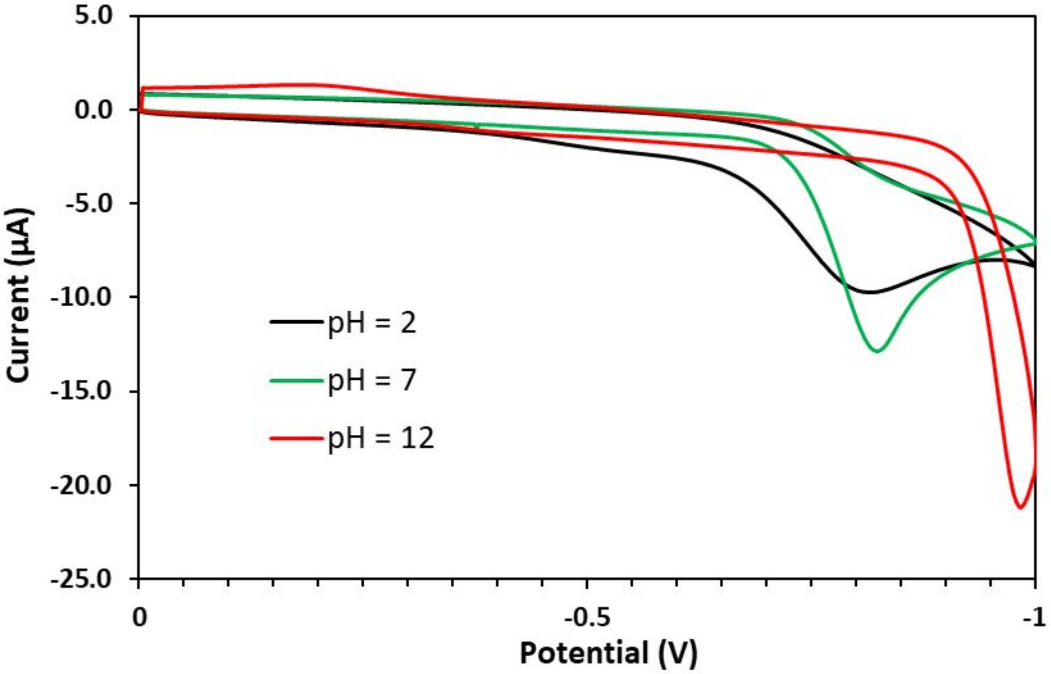

Figure S 137. Cyclic voltamogram for $\mathbf{R_a}H{\cdot}2Cl$ at 2 mM in aqueous solution at pH 2 (black), pH 7 (green) and pH 12 (red).

7. Cell viability assays

Fibroblast cells (HFF-1; ATTC® SCRC-1041TM) were maintained in Dulbecco's modified eagle's medium (DMEM) supplemented with 10% FBS and 1% P/S and kept at 37 °C in a humidified atmosphere containing 5% CO2 until their use. HFF-1 cells were seeded in 96 well-plates at an initial density of 104 cells/well and allowed to attach for 24 h at 37 °C. The following day, culture medium was removed, replenished by fresh medium (90 µl) and cells were exposed to different concentration of **R_aH**·2Cl and **MV** (10 µl; final concentrations 0.01, 0.05, 0.1, 0.5 and 1 mM). Control conditions included cells cultured in in culture medium, but in absence of compound. Cells were further incubated for 24 h at 37°C and cell viability was monitored using the tetrazolium salt (WST-1) method.[6] Absorbance at 450 nm was measured using a Synergy HTX Plate Reader (Biotek, Winooski, VT, USA) and the percent of cell viability (%) was calculated as follows:

$$Viability\ (\%) = \frac{A_{sample}}{A_{negative\ control}}\ x\ 100$$

Where A_{sample} and $A_{negative\ control}$ depict those absorbance values measured at 450 nm in presence and in absence of compound, respectively. All conditions were tested by triplicate in two independent experiments. Data were expressed as mean ± standard deviation (SD). Statistical analysis was performed by two-ways and one-way ANOVA with Tukey's LSD post hoc test using *IBM SPSS Statistics version 23*. A $p \leq 0.05$ was considered statistically significant.

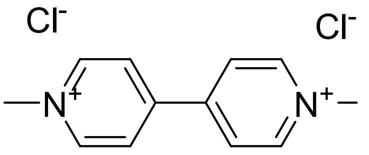

MV

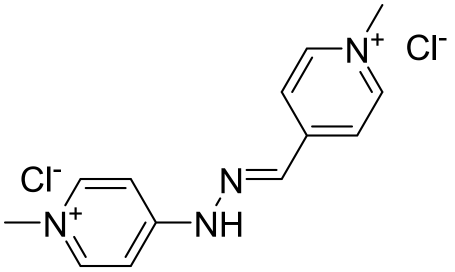

R_aH·2Cl

Figure S 138: Compounds tested in WST-1 assay for cell viability.

[6] Carballo-Pedrares, N.; Kattar, A.; Concheiro, A.; Alvarez-Lorenzo, C.; Rey-Rico, Niosomes-based gene delivery systems for effective transfection of human mesenchymal stem cells. A. *Matter. Sci. Eng.* **2021**, *18*, 112307.

8. Host-guest chemistry

8.1. Self-assembly of $R_aH{\cdot}2Cl$ with cucurbit[7]uril:

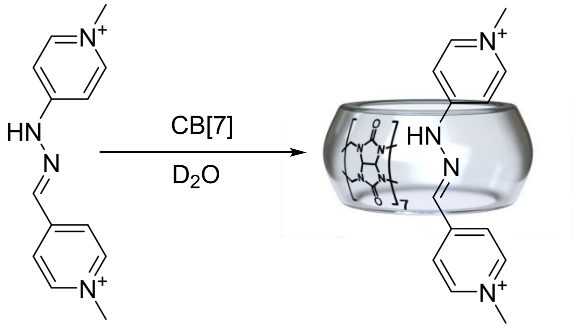

RaH·2Cl **RaH⊂CB[7]**

- **RaH⊂CB[7] at pD = 7**

Firstly, guest **R_aH**·2Cl (1 mM) was dissolved in D_2O with phosphate buffer solution (50 mM, pD7). On the other hand, host CB[7] (6 mM) was dissolved in the previous solution in order to keep constant the concentration of the guest. Then, 100 µL of the second solution was added over 0.5 mL of the **R_aH**·2Cl solution to reach 1 eq of CB[7]. The mixture was sonicated for 10 min. Finally, ^{1}H-NMR was recorded.

^{1}H NMR (500 MHz, D_2O): δ 8,29 (d, J = 6.4 Hz, 2H), 8.18 (d, J = 7.1 Hz, 2H), 7.35 (d, J = 6.3 Hz, 2H), 7.25 (s, 1H), 5.66 (d, J = 15.4 Hz, 14H), 5.46 (s, 14H), 4.35 (s, 3H), 4.17 (d, J = 15.4 Hz, 14H), 4.06 (s, 3H). **HRMS** (ESI) (*m/z*): calcd for $[C_{62}H_{66}N_{35}O_{18}]^{+2}$ 695.2400, found 695.2405.

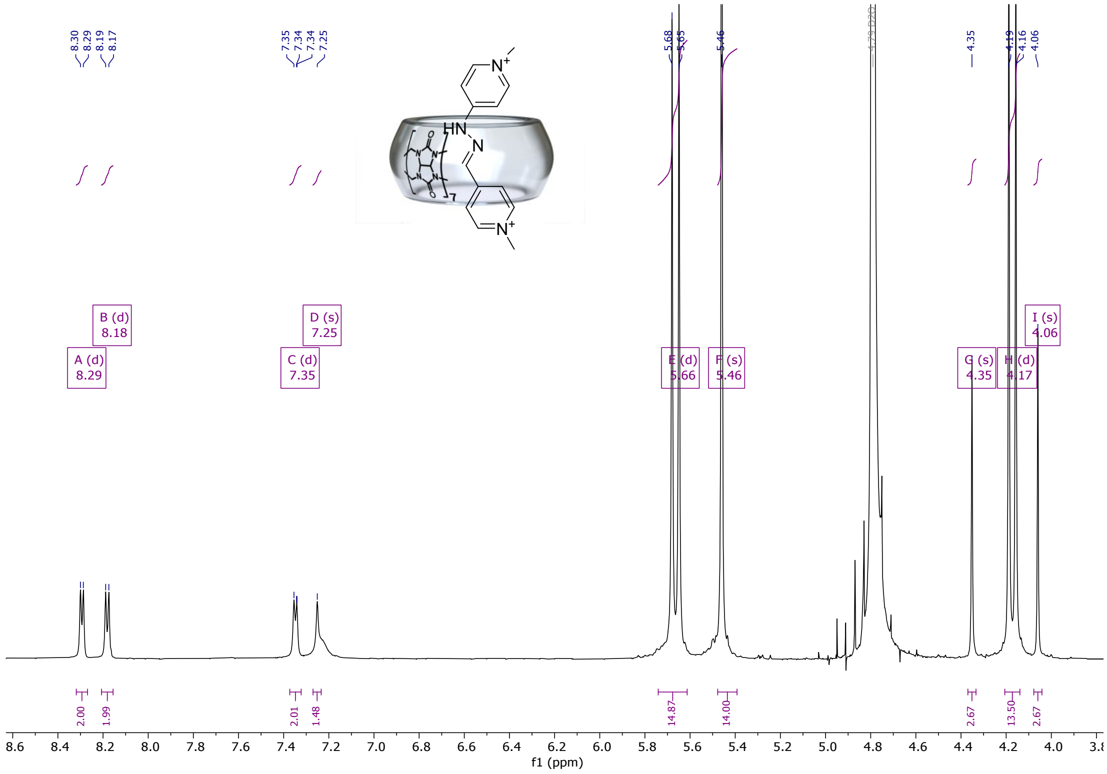

Figure S 139: ^{1}H NMR (500 MHz, D_2O) spectrum of $\mathbf{R_aH}^{+2}$⊂CB[7] at pD=7.

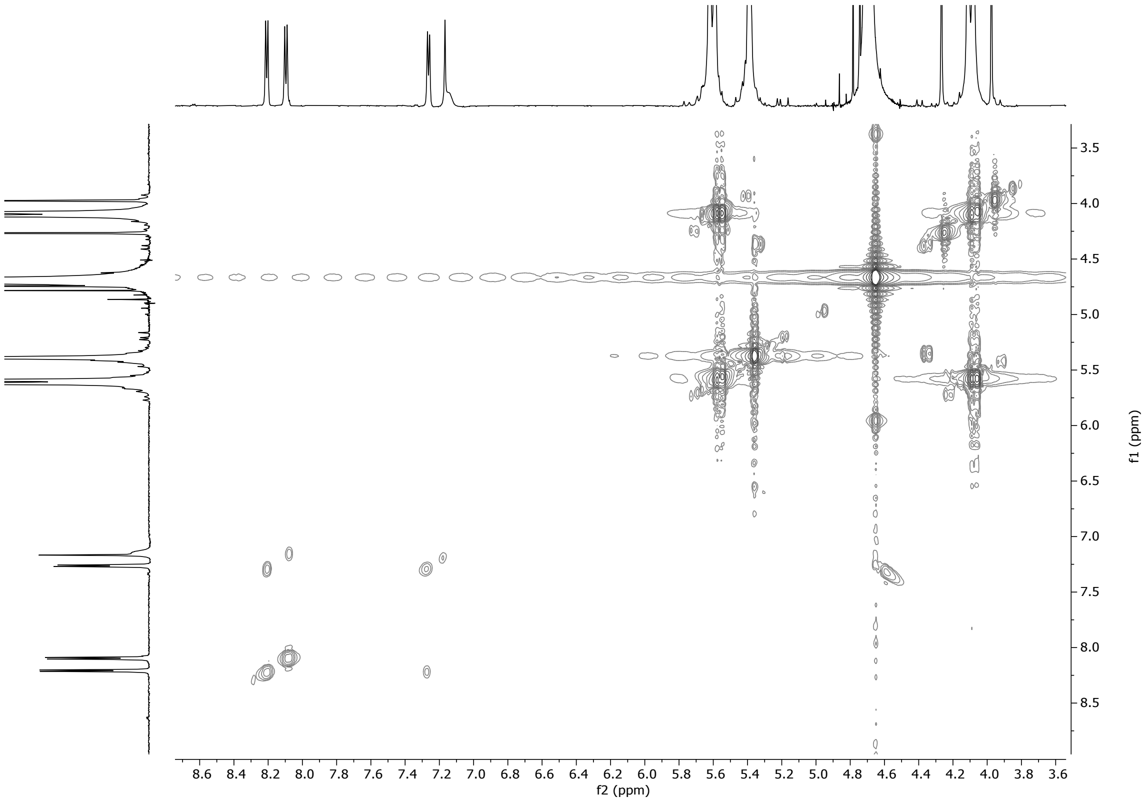

Figure S 140: COSY (500 MHz, D_2O) spectrum of $\mathbf{R_aH}^{+2}$⊂CB[7] at pD=7.

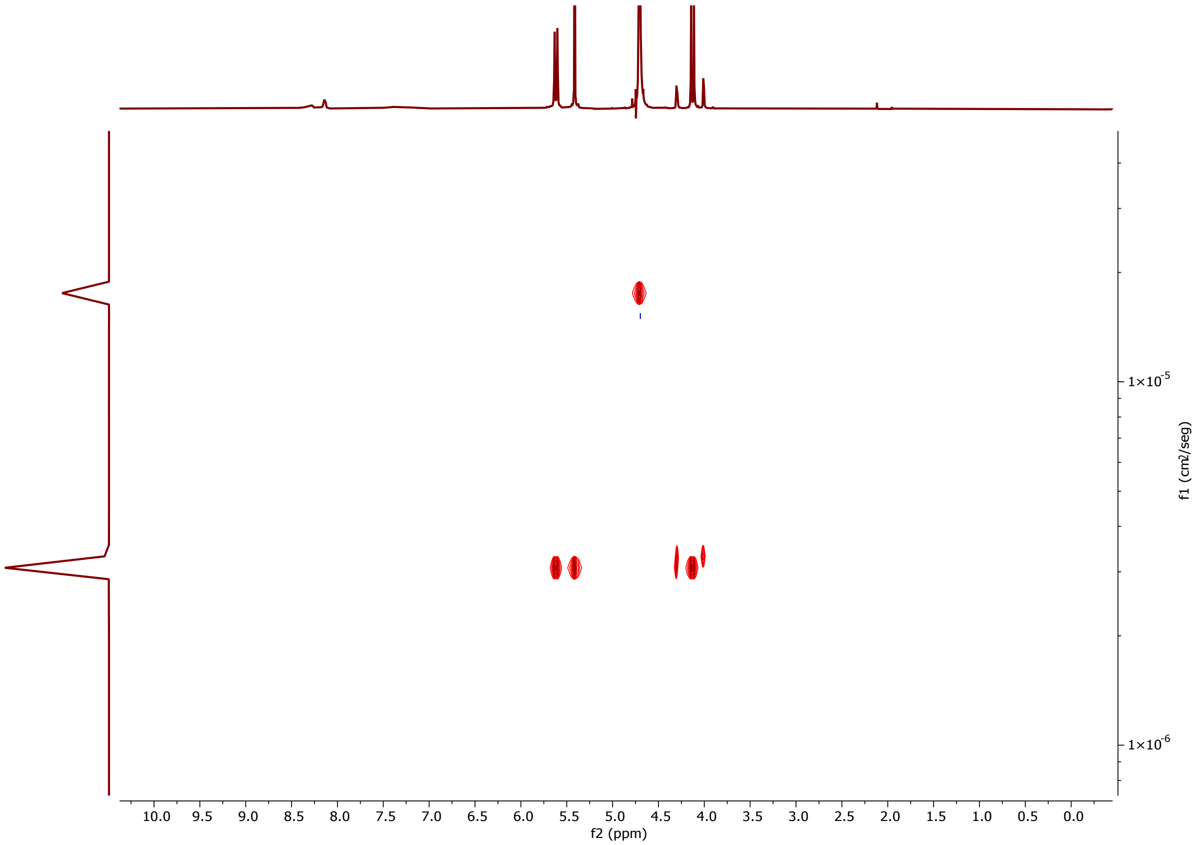

Figure S 141: DOSY (400 MHz, D_2O, 298 K) spectrum of $\mathbf{R_aH}^{+2}$⊂CB[7] at pD=7.

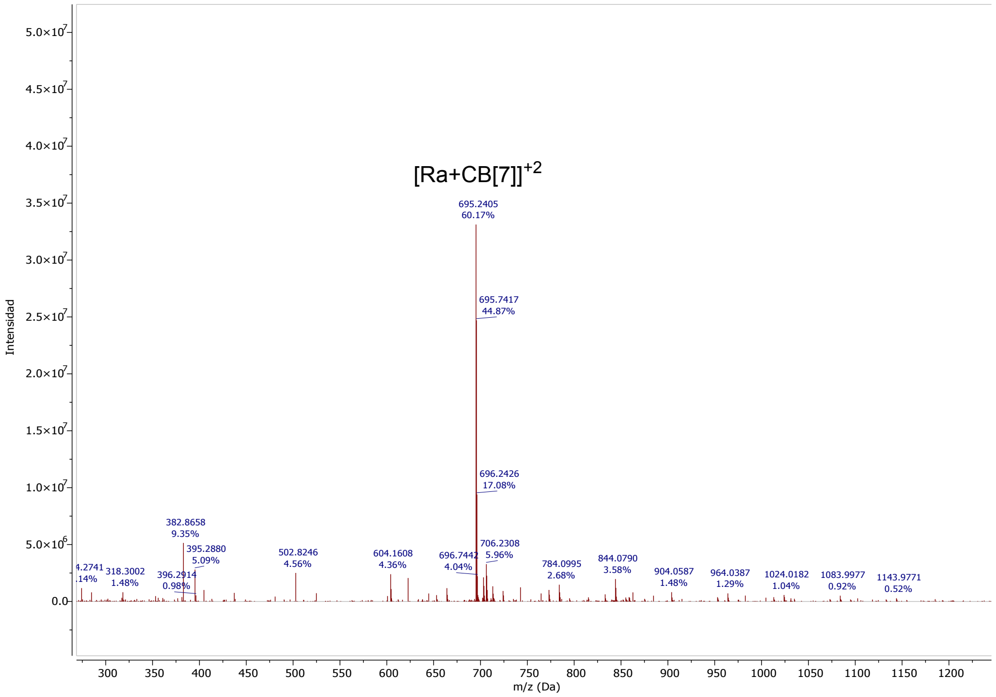

Figure S 142: MS-ESI spectrum of $\mathbf{R_aH}^{+2}$⊂CB[7]at pD=7.

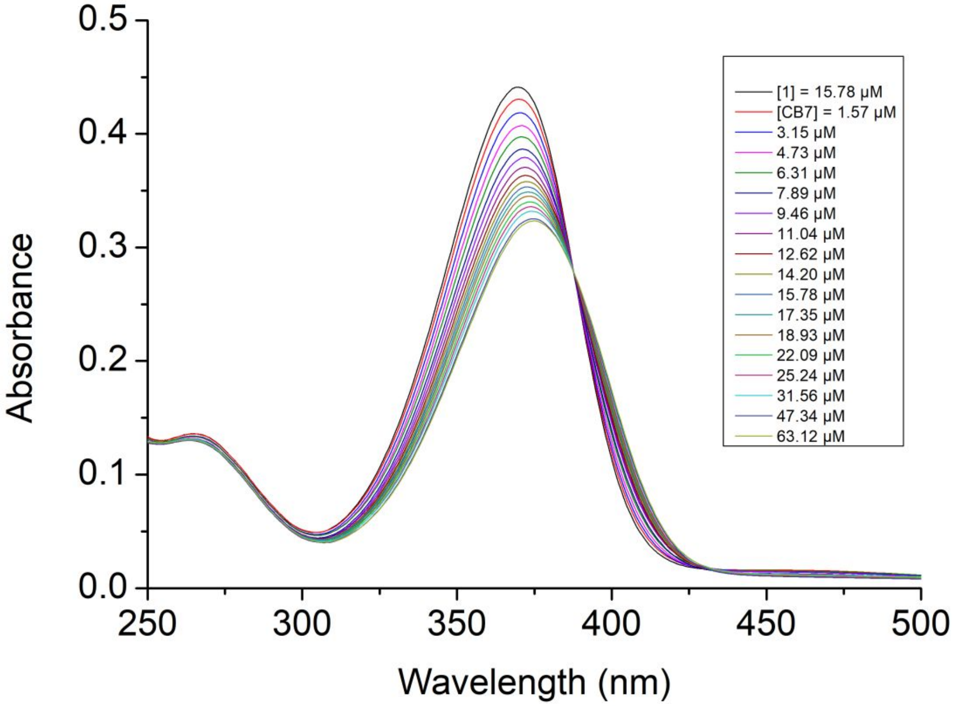

Figure S 143: UV-VIS titration of $\mathbf{R_aH}$·2Cl with CB[7] in buffered aqueous solution at pH=7.

UV-VIS titration were carried out in buffered aqueous solution (50 mM, pH = 7) by keeping the $\mathbf{R_a}$H·$2Cl_2$ concentration constant (15.78 µM) and adding increasing aliquots of CB[7]. The mechanism proposed for the fitting process equilibria, and introduced on the software Dynafit[3] was the following:

$$\mathbf{RaH}^{+2} + \mathrm{CB[7]} \Leftrightarrow \mathbf{RaH}^{+2}\subset \mathrm{CB[7]}.$$

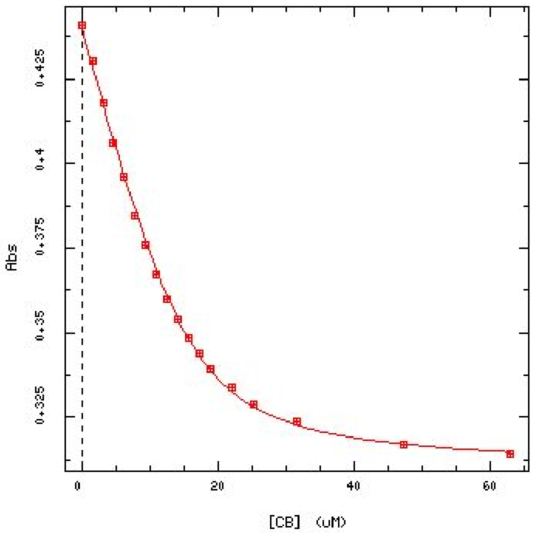

Figure S 144: Fitting of UV-VIS titration of $\mathbf{R_aH}$·2Cl with CB[7] pH=7.

Concentration (µM)	Absorbance	Residual
0	0.41512	-0.0013447
1.49	0.40713	0.0031003
2.97	0.39549	0.00365881
4.46	0.37985	9.50E-05
5.94	0.36684	-0.0011947
7.43	0.35422	-0.0023955
8.92	0.34256	-0.0031653
10.4	0.33303	-0.0026013
11.89	0.32446	-0.0019871
13.37	0.31743	-0.0011193
14.86	0.31219	0.00018937
16.35	0.30818	0.00132455
17.83	0.30503	0.00206916
20.8	0.30016	0.00237133
23.78	0.29672	0.00198053
29.72	0.29266	0.00113009
44.58	0.28827	-0.0003452
59.44	0.28579	-0.0017661

Table S 16: Experimental data for the titration. The *p* value obtained from "runs-of-sign" was 1.

- **RaH⊂CB[7] at pD = 12**

Firstly, guest $\mathbf{R_aH}$·2Cl (1 mM) was dissolved in D_2O with phosphate buffer solution (50 mM, pD12). On the other hand, host CB[7] (6 mM) was dissolved in the previous solution in order to keep constant the concentration of the guest. Then, 100 µL of the second solution was added over 0.5 mL of the $\mathbf{R_aH}$·2Cl solution to reach 1 eq of CB[7]. The mixture was sonicated for 10 min. Finally, ^{1}H-NMR was recorded.

^{1}**H NMR** (500 MHz, D_2O): δ 8.28 (brs, 2H), 8.02 (brs, 1H), 7.91 (brs, 2H), 7.65 (d, J = 7.2 Hz, 2H), 5.68 (d, J = 15.3 Hz, 16H), 5.47 (s, 16H), 4.17 (d, J = 15.3 Hz, 16H), 4.12 (s, 3H), 3.75 (s, 3H). **HRMS** (ESI) (*m/z*): calcd for $[C_{62}H_{66}N_{35}O_{18}]^{+2}$ 794.7674, found 794.7679.

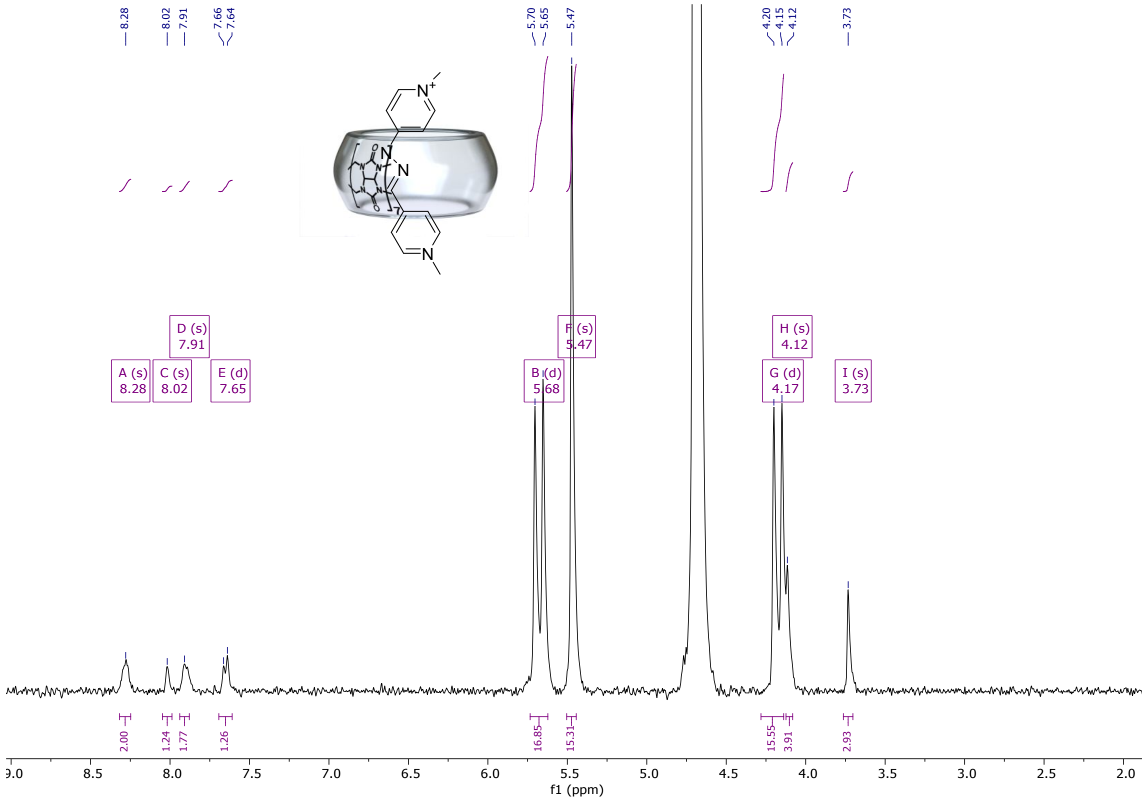

Figure S 145: ^{1}H NMR (400 MHz, D_2O) spectrum of $\mathbf{R_a}^{+}\subset$CB[7] at pD=12.

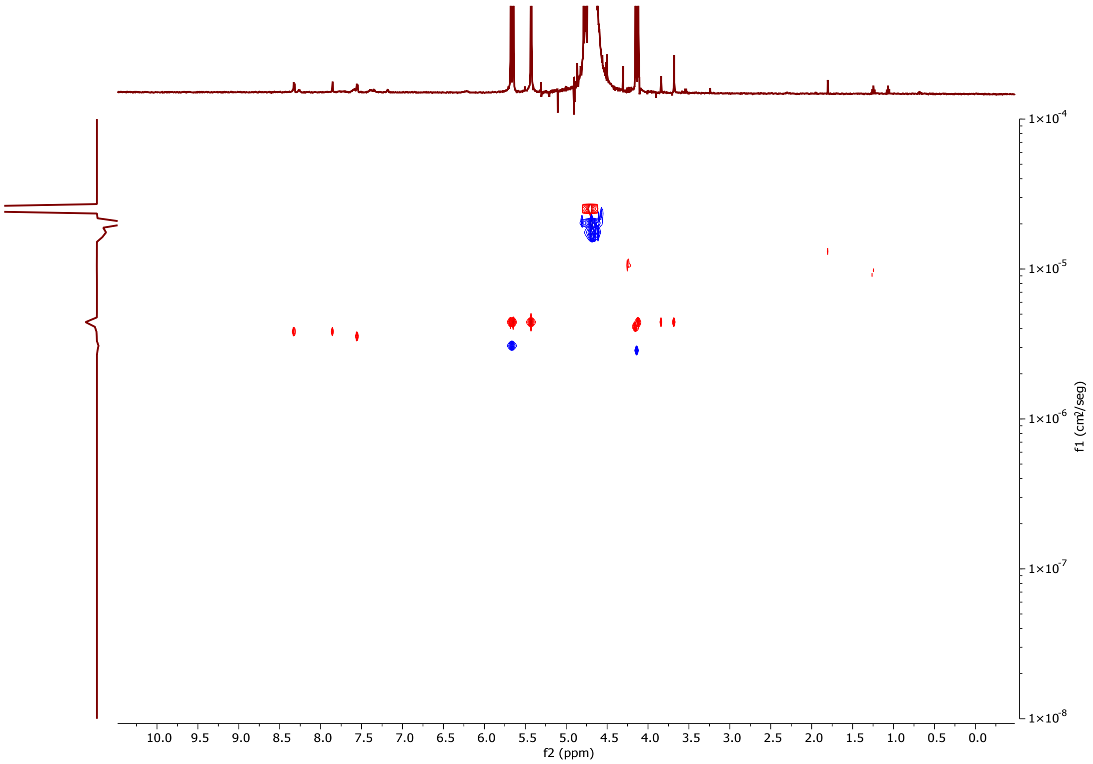

Figure S 146: DOSY (400 MHz, D_2O, 298 K) spectrum of $\mathbf{R_a}\cdot$2Cl and CB[7] at pD=12.

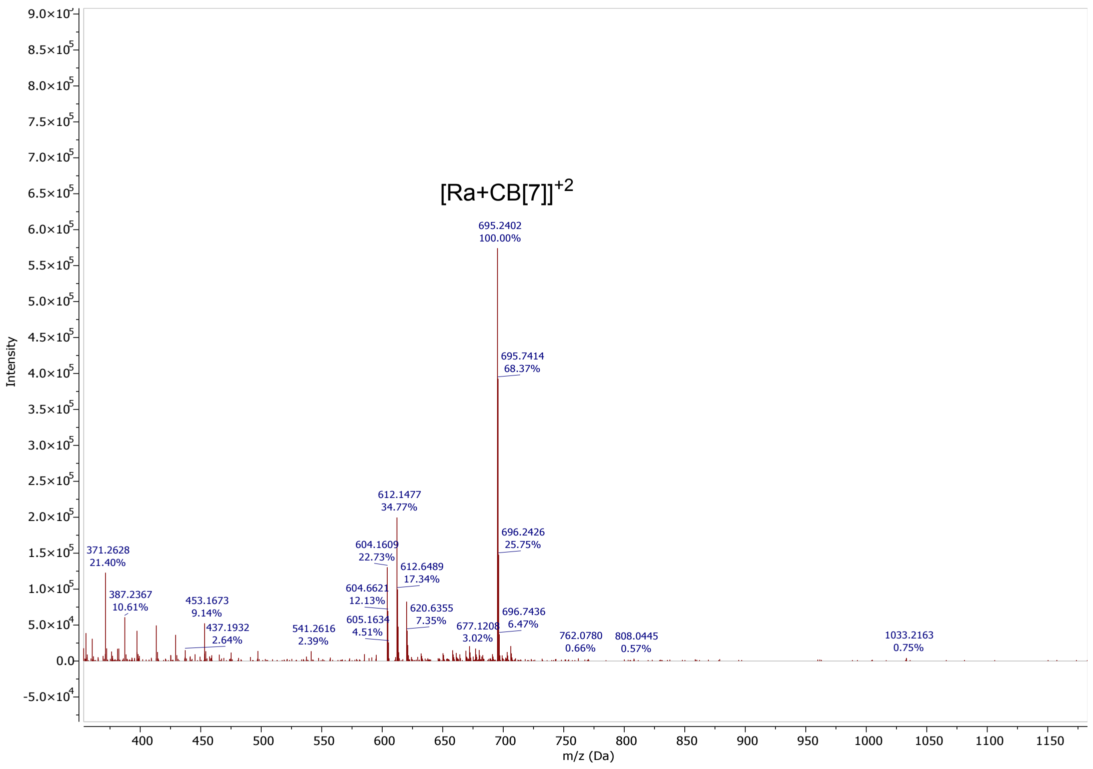

Figure S 147: MS-ESI spectrum of $\mathbf{R_a}\cdot 2Cl$ and CB[7] at pD=12.

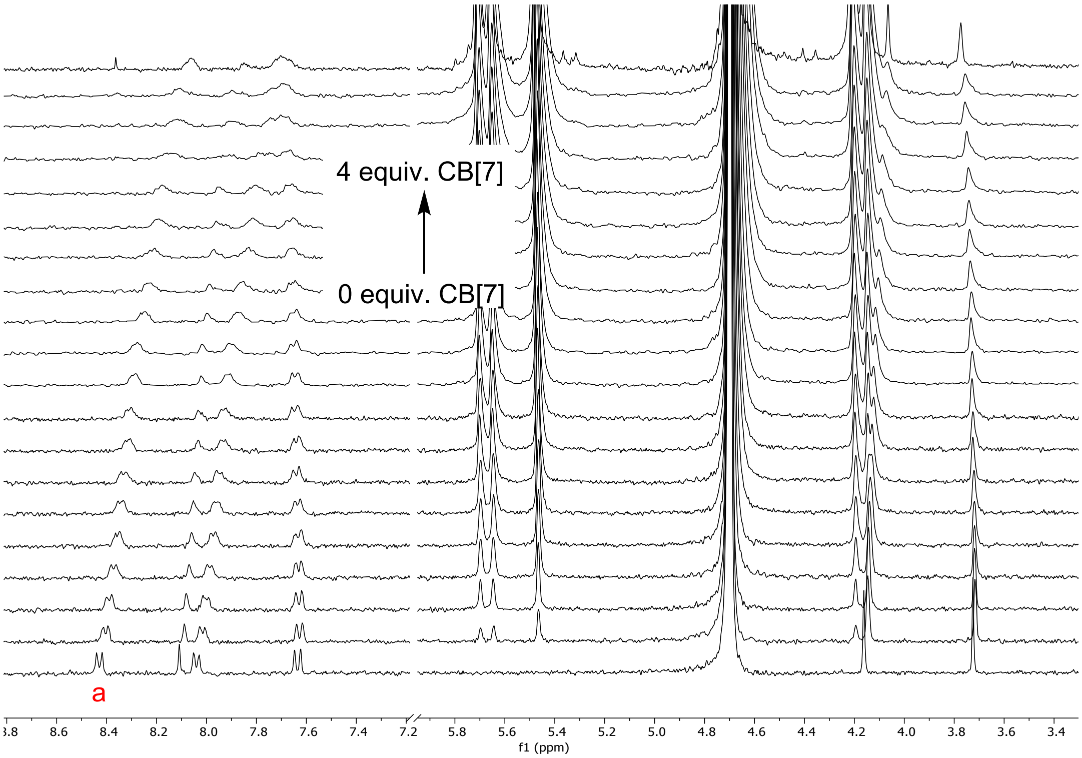

Figure S 148: ^{1}H NMR (400 MHz, D_2O) spectra of $\mathbf{R_aH}\cdot 2Cl$ (1000 uM) upon titration with **CB[7]** (6000 uM) in 50mM phosphate buffer solution pD=12. The chemical shift of signals H_a were used for the fitting.

The chemical shifts (ppm) were selected using the automatic peak picking routine with standard settings implemented in the software Mestrenova (version 14.1.0). The mechanism proposed for the fitting process equilibria, and introduced on the software Dynafit[3] was the following: **Ra**$^{+}$ + CB[7] ⇔ **Ra**$^{+}$⊂CB[7].

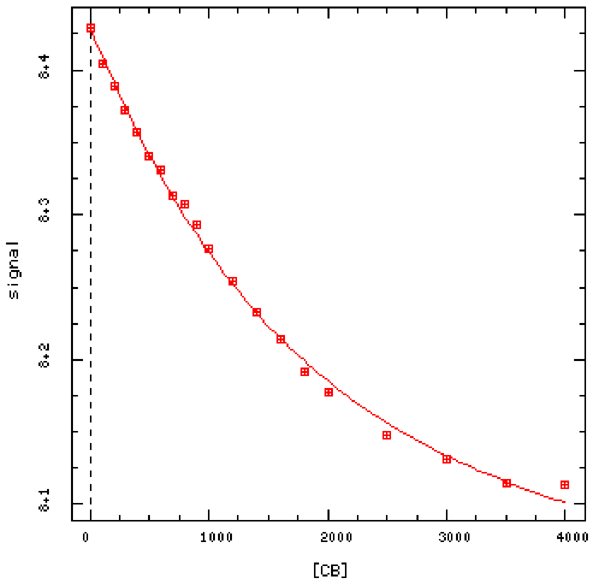

Figure S 149: Fitting of the experimental data of signals H_a.

Equivalents	Chemical shift (ppm)	Residual
0.1	8.40451	-0.0054099
0.2	8.38898	-0.002316
0.3	8.37239	-0.0012353
0.4	8.35642	-0.000435
0.5	8.34069	-0.0002858
0.6	8.33103	0.00508827
0.7	8.31354	0.00181984
0.8	8.30699	0.00871004
0.9	8.29259	0.00701566
1	8.27717	0.00360168
1.2	8.25365	0.00213712
1.4	8.23326	0.00144642
1.6	8.21399	-0.0002191
1.8	8.19163	-0.0068078
2	8.1768	-0.007475
2.5	8.14739	-0.0073074
3	8.11886	-0.0127007
3.5	8.11453	0.00140617
4	8.11326	0.0150875

Table S 17: Experimental data for the titration. The *p* value obtained from "runs-of-sign" was 0.03.

8.2. pK_a determination for R_a⊂CB[7] by UV-Vis

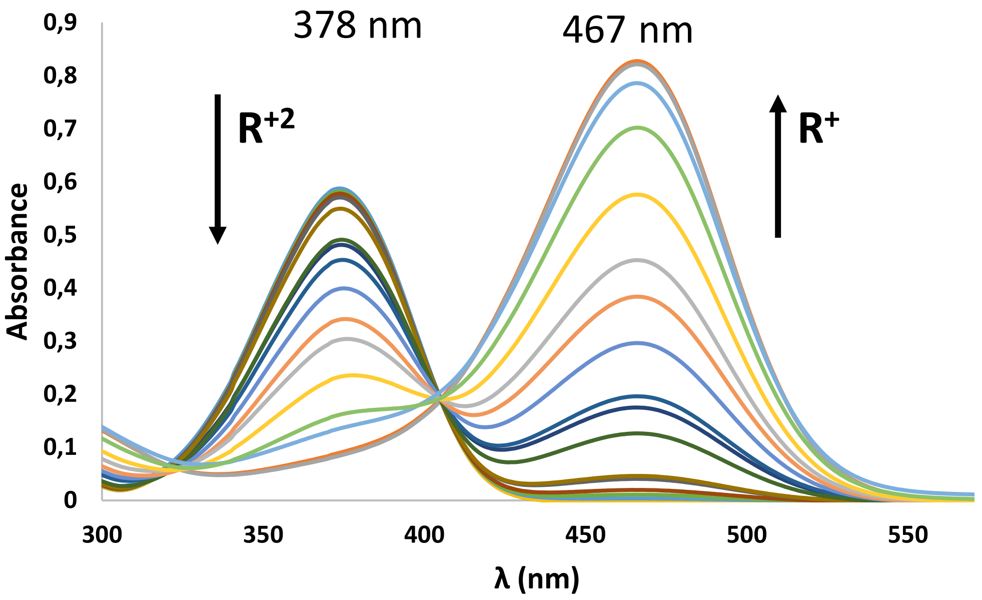

Figure S 150: UV-Vis spectra for the titration of **R_a**⊂CB[7] at 20 µM in NaH_2PO_4/Na_2HPO_4, $KHCO_3/K_2CO_3$ and Na_2HPO_4/Na_2PO_4.

pH (measured)	V (mL) of solution A	V (mL) of solution B	V (mL) of solution C	V (mL) of solution D	V (mL) of solution E	ABS (467 nm)	log[A_{467}-AR_a^{2+})/(AR_a^{+}-A_{467})]
7.11(**R_a^{2+}**)	0.8	1.6	-	-	-	0.00339	-
7.73	0.32	2.08	-	-	-	0.01064	-2.051
8.00	0.2	2.2	-	-	-	0.01962	-1.696
8.31	0.1	2.3	-	-	-	0.04017	-1.330
9.00	-	-	2.3	0.1	-	0.12615	-0.756
9.26	-	-	2.2	0.2	-	0.19604	-0.515
9.43	-	-	2.0	0.4	-	0.29641	-0.257
9.65	-	-	1.8	0.6	-	0.38344	0.067
9.85	-	-	1.6	0.8	-	0.45221	0.078
10.16	-	-	1.2	1.2	-	0.57549	0.357
10.60	-	2.2	-	-	0.2	0.70206	0.748
11.08	-	2.0	-	-	0.4	0.78512	1.274
11.80	-	1.2	-	-	1.2	0.82103	2.157
12.18 (**R_a^{+}**)	-	-	-	-	2.4	0.82672	-

Table S 18: Experimental data obtained for the UV-Vis titration of **R_aH**⊂CB[7] at 20 µM in NaH_2PO_4/Na_2HPO_4, $KHCO_3/K_2CO_3$ and Na_2HPO_4/Na_2PO_4 buffer. **Solution A**: 20 mM of NaH_2PO_4. **Solution B**: 20 mM of $NaHPO_4$. **Solution C**: 20 mM of Na_2PO_4. **Solution D**: 20 mM of $KHCO_3$. **Solution E**: 20 mM of K_2CO_3.

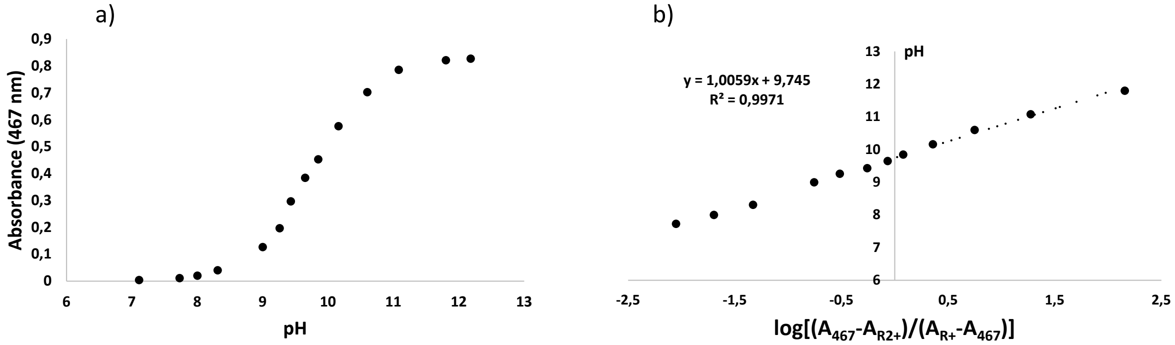

Figure S 151: a) Absorption of $\mathbf{R_aH^{2+}}$⊂CB[7] at λ = 467 nm plotted against pH. b) Linear fitting of pH plotted against log[(A_{467}-A_{R2+})/(A_{R+}-A_{467})], where pK_a value is 9.74.

8.3. Self-assembly of $\mathbf{R_aH}$·2Cl with cucurbit[8]uril:

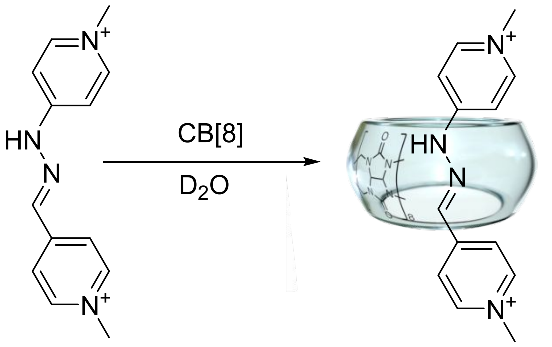

RaH·2Cl **RaH⊂** CB[8]

- **$\mathbf{R_aH}$⊂CB[8] at pD = 7**

A 1mM solution of guest $\mathbf{R_aH}$·2Cl in D_2O with phosphate buffer solution (50 mM) at pD 7 was prepared. Then, CB[8] was added in excess and the mixture was sonicated and heated using an oil bath at 60 °C for 15 min. Finally, the sample was filtered off to remove the excess of CB[8].

^{1}H NMR (500 MHz, D_2O): δ 8.34 (m, 3H), 8.26 (brs, 2H), 7.57 (m, 4H), 5.80 (d, J = 15.3 Hz, 16H), 5.55 (s, 16H), 4.29 (s, 3H), 4.25 (d, J = 15.3 Hz, 16H), 4.10 (s, 3H). **HRMS (ESI)** (*m/z*): calcd for $[C_{56}H_{60}N_{31}O_{16}]^{+2}$ 711.7428, found 711.7434.

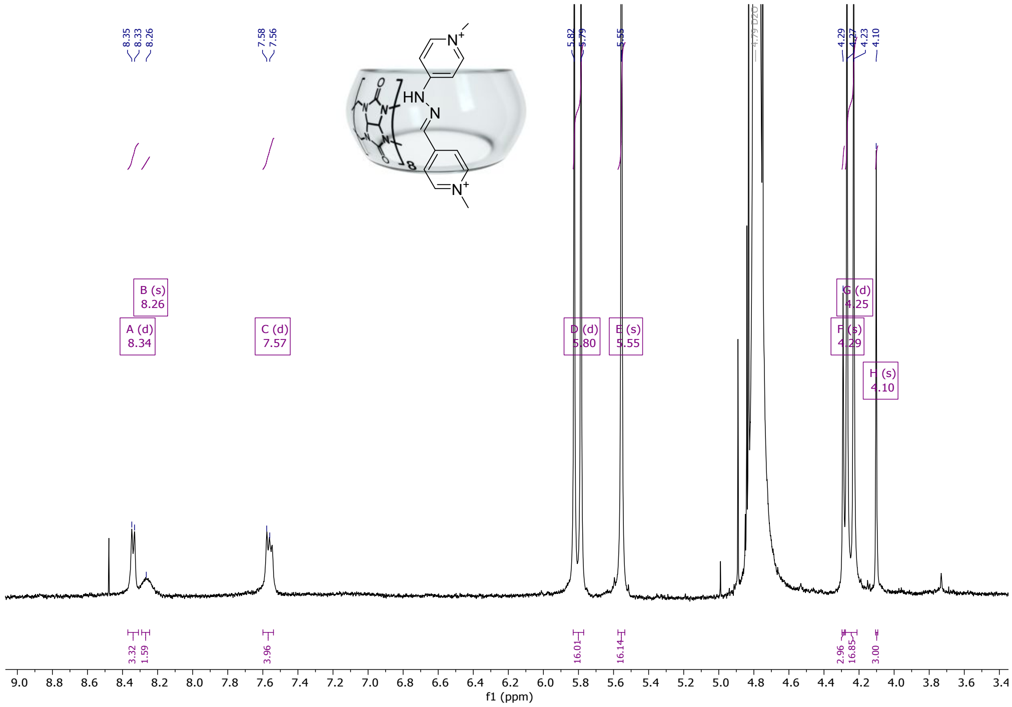

Figure S 152: ^{1}H NMR (500 MHz, D_2O) spectrum of $\mathbf{R_aH^{2+}}\subset$CB[8] at pD=7.

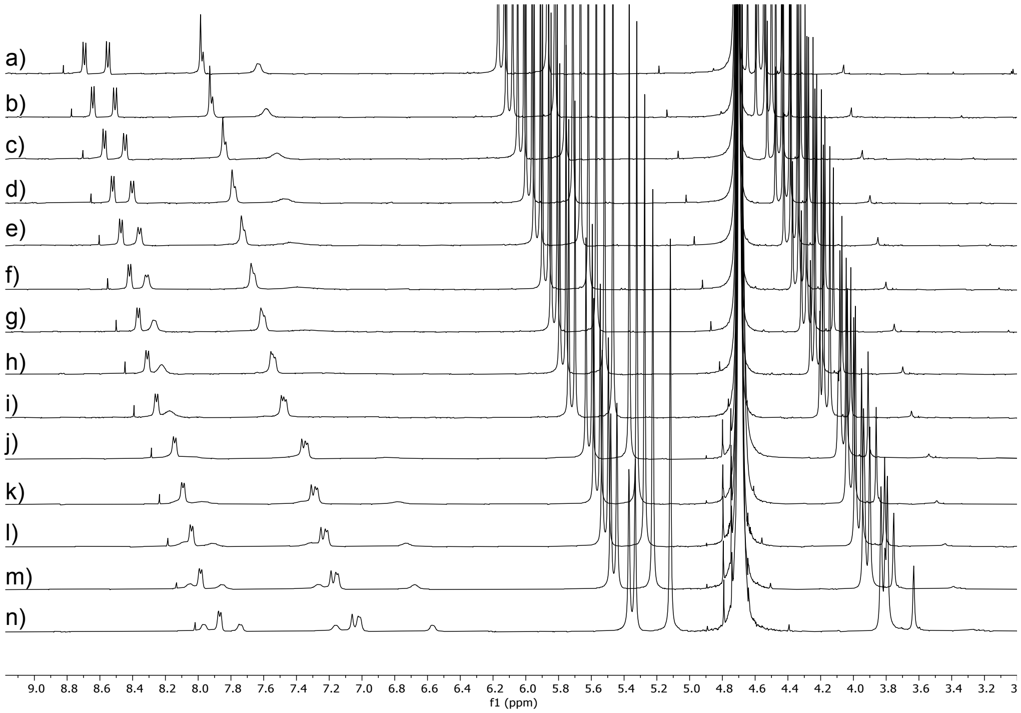

Figure S 153: T. Var. (400 MHz, D_2O, pD=7) spectrum of a mixture of $\mathbf{R_aH^{2+}}\subset$CB[8] at: a) 338.15 K b) 333.15 K c) 328.15 K d) 323.15 K e) 318.15 K f) 313.15 K g) 308.15 K h) 303.15 K i) 298.15 K j) 293.15 K k) 288.15 K l) 283.15 K m) 278.15 K n) 273.15 K.

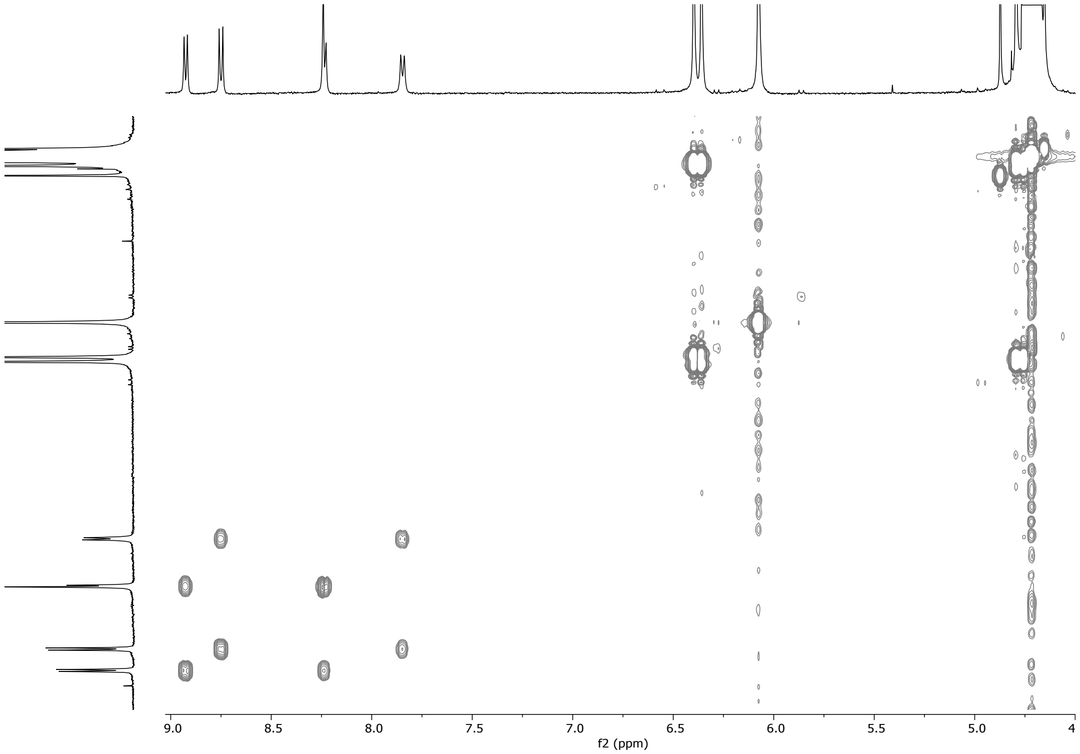

Figure S 154: COSY (500 MHz, D_2O, pD=7) spectrum of $\mathbf{R_aH^{2+}}$⊂CB[8] at 338.15 K.

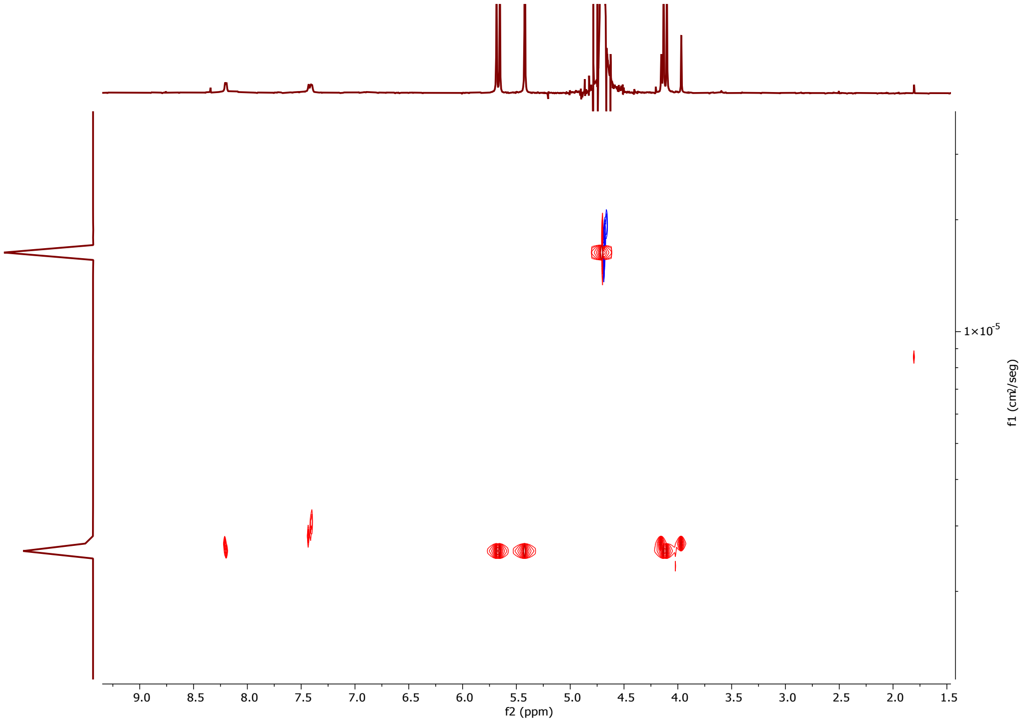

Figure S 155: DOSY (400 MHz, D_2O, 298 K) spectrum of $\mathbf{R_aH^{2+}}$⊂CB[8] at pD=7.

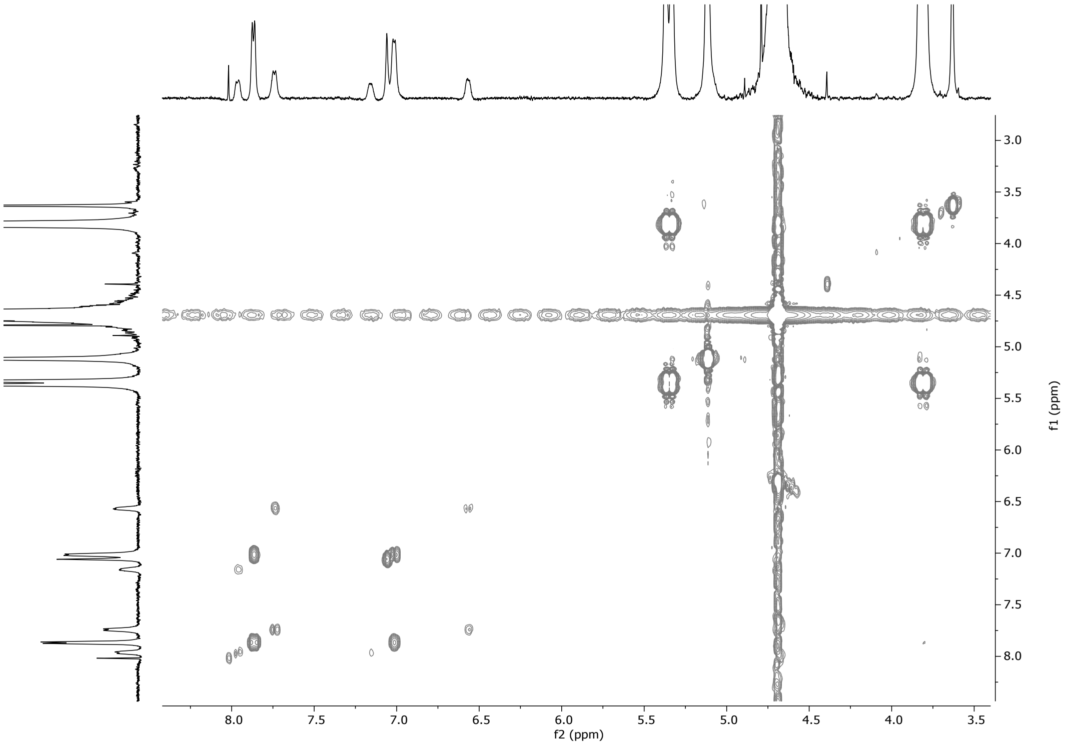

Figure S 156: COSY (500 MHz, D2O, pD=7) spectrum of $\mathbf{R_aH^{2+}}$⊂CB[8] at 273.15 K.

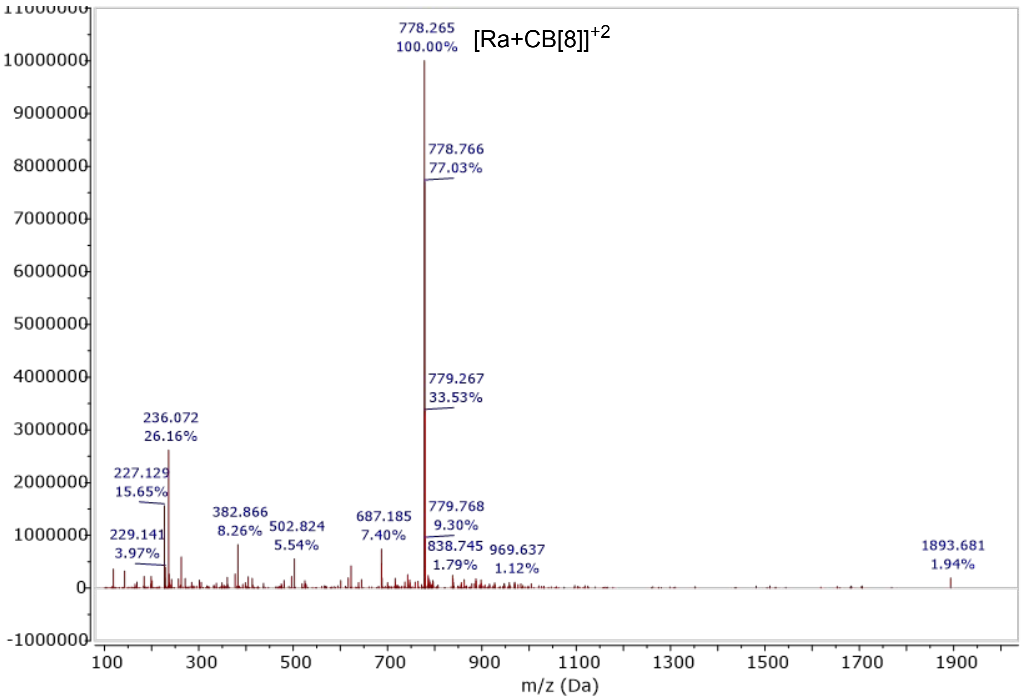

Figure S 157: MS-ESI spectrum of $\mathbf{R_aH^{2+}}$⊂CB[8] at pD=7.

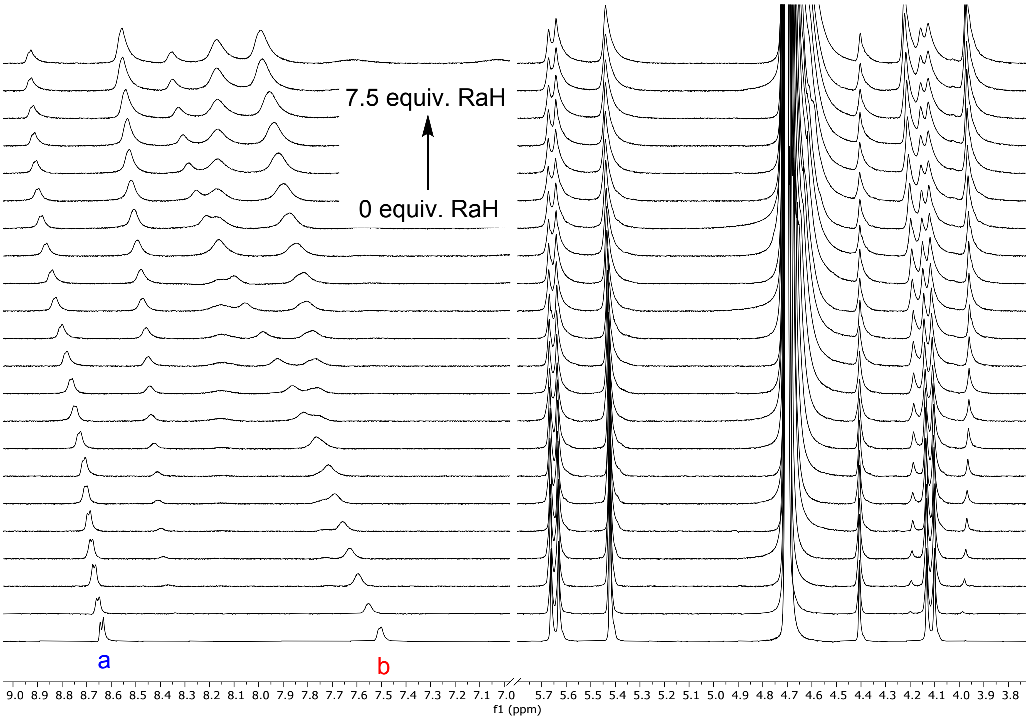

Figure S 158: ^{1}H NMR (400 MHz, D_2O) spectra of **MV**⊂CB[8] (500 uM) upon titration with **RaH**·2Cl (5000 uM) in 50mM phosphate buffer solution pD=7. The chemical shift of signals H_a and H_b were used for the fitting.

The chemical shifts (ppm) were selected using the automatic peak picking routine with standard settings implemented in the software Mestrenova (version 14.1.0). The mechanism proposed for the fitting process equilibria, and introduced on the software Dynafit[3] was the following (the association constant value of CB[8] with the MV is introduced as a fixed parameter)[7]:

MV⊂CB[8] ⇔ **MV** + CB[8]

RaH^{+2} + CB[8] ⇔ **RaH**$^{+2}$⊂CB[8]

[7] Jeon, W. S. Kim, H.-J.; Lee, C.; Kim, K. Control of the stoichiometry in host–guest complexation by redox chemistry of guests: Inclusion of methylviologen in cucurbit[8]uril. *Chem. Commun.* **2002**, 1828–1829.

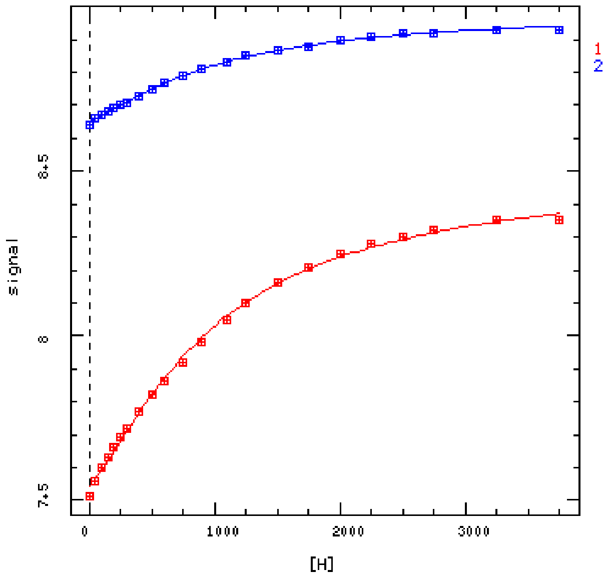

Figure S 159: Fitting of the experimental data of signals H_a and H_b.

Concentration (µM)	Chemical shift (Ha)	Residual (Ha)	Concentration (µM)	Chemical shift (Hb)	Residual (Hb)
0	7.51	-0.0317828	0	8.64	-0.0115706
50	7.56	-0.0038672	50	8.66	0.00071031
100	7.6	0.0104516	100	8.67	0.00173399
150	7.63	0.0122671	150	8.68	0.00188271
200	7.66	0.0126904	200	8.69	0.00154481
250	7.69	0.0126035	250	8.7	0.00102859
300	7.72	0.0126247	300	8.71	0.00055017
400	7.77	0.00447301	400	8.73	0.00022449
500	7.82	-6.51E-06	500	8.75	0.00118235
600	7.86	-0.0102079	600	8.77	0.00363552
750	7.92	-0.0173824	750	8.79	0.00015612
900	7.98	-0.0154346	900	8.81	-0.0001348
1100	8.05	-0.0104981	1100	8.83	-0.0028763
1250	8.1	-0.0014981	1250	8.85	0.00279303
1500	8.16	0.00204016	1500	8.87	0.00305805
1750	8.21	0.00714601	1750	8.88	-0.0026337
2000	8.25	0.010924	2000	8.9	0.00470564
2250	8.28	0.0112652	2250	8.91	0.00433905
2500	8.3	0.00663886	2500	8.92	0.00573145
2750	8.32	0.00592671	2750	8.92	-0.001508
3250	8.35	0.00314798	3250	8.93	-0.0029651
3750	8.35	-0.0215214	3750	8.93	-0.0115878

Table S 19: Experimental data for the titration. The *p* value obtained from “runs-of-sign” was 0.04.

- **$2R_a^+ \subset$CB[8] at pD = 12**

A 1mM solution of guest **R_aH**·2Cl in D_2O with phosphate buffer solution (50 mM) at pD 12 was prepared. Then, CB[8] was added in excess and the mixture was sonicated and heated using an oil bath at 60 °C for 15 min. Finally, the sample was filtered off to remove the excess of CB[8].

^{1}H NMR (500 MHz, D_2O): δ 8.42 (brs, 4H), 8.16 (s, 2H), 8.05 (d, J = 7.3 Hz, 4H), 7.95 (brs, 4H), 6.11 (d, J = 15.3 Hz, 16H), 5.82 (s, 16H), 4.52 (d, J = 15.3 Hz, 16H), 4.33 (s, 6H), 4.13 (s, 6H).

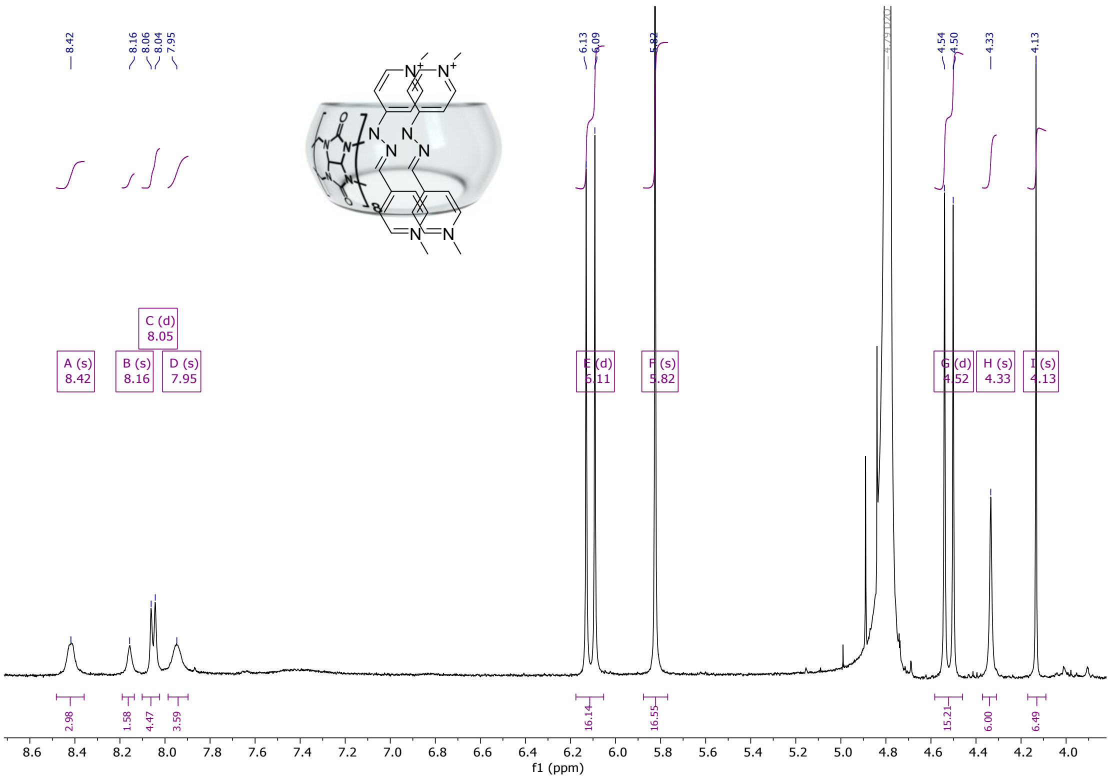

Figure S 160: ^{1}H NMR (400 MHz, D_2O, 328.15 K) spectrum of 2**R_a**$^+ \subset$CB[8] at pD=12.

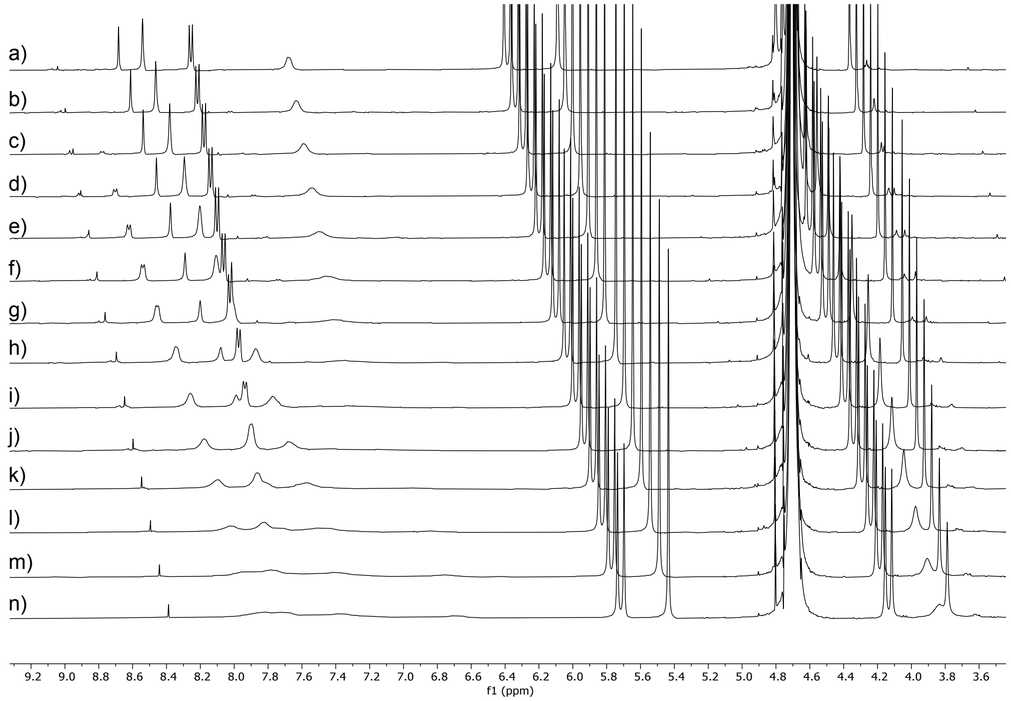

Figure S 161: T. Var. (400 MHz, D2O, pD=12) spectrum of a mixture of 2$\mathbf{R_a}^+$⊂CB[8] at: a) 363.15 K b) 358.15 K c) 353.15 K d) 348.15 K e) 343.15 K f) 338.15 K g) 333.15 K h) 328.15 K i) 323.15 K j) 318.15 K k) 313.15 K l) 308.15 K m) 303.15 K n) 298.15 K.

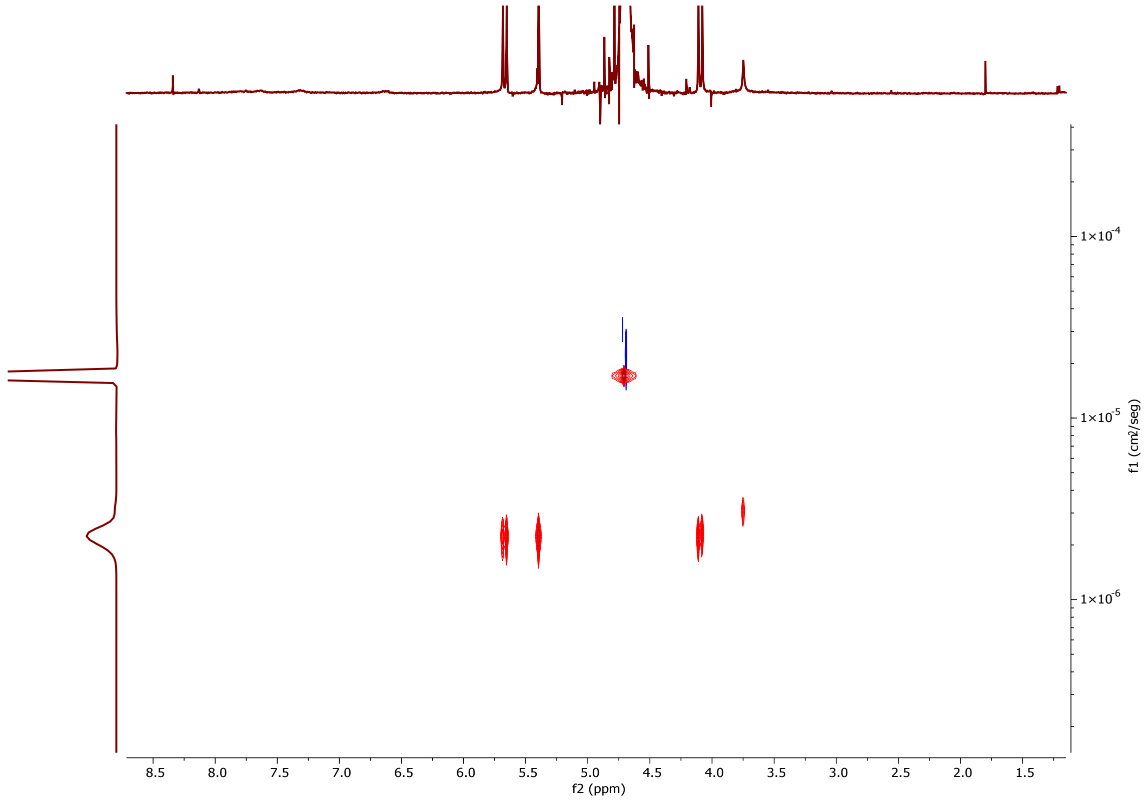

Figure S 162: DOSY (500 MHz, D_2O) spectrum of 2$\mathbf{R_a}^+$⊂CB[8] at pD=12.

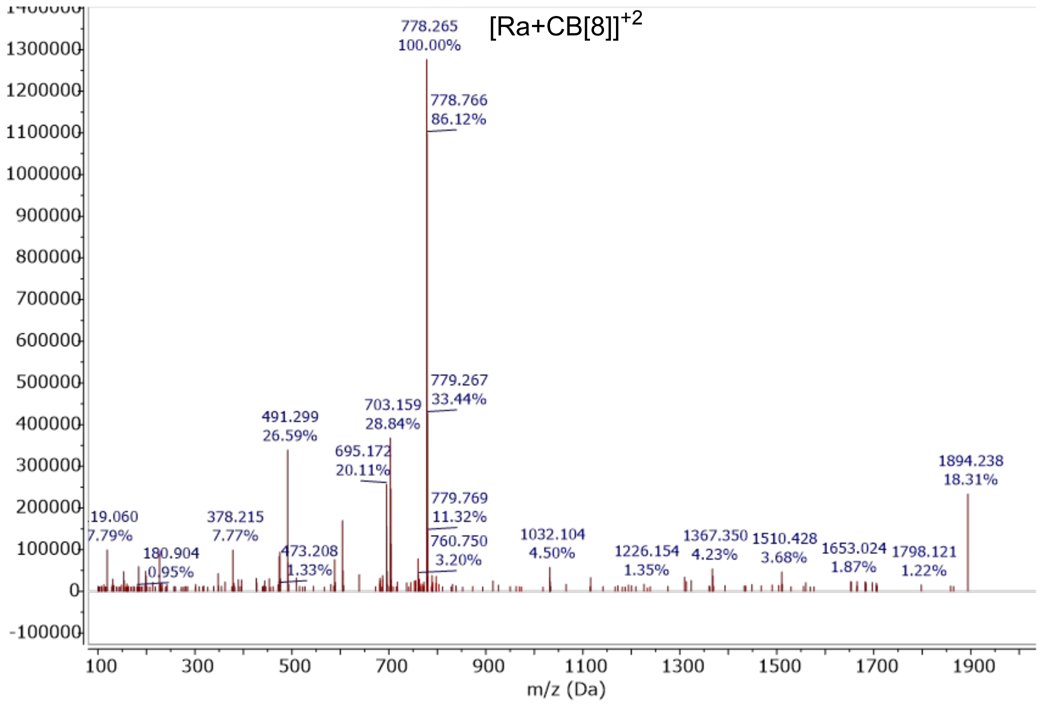

Figure S 163: MS-ESI spectrum of 2$\mathbf{R_a}^+$⊂CB[8] at pD=12.

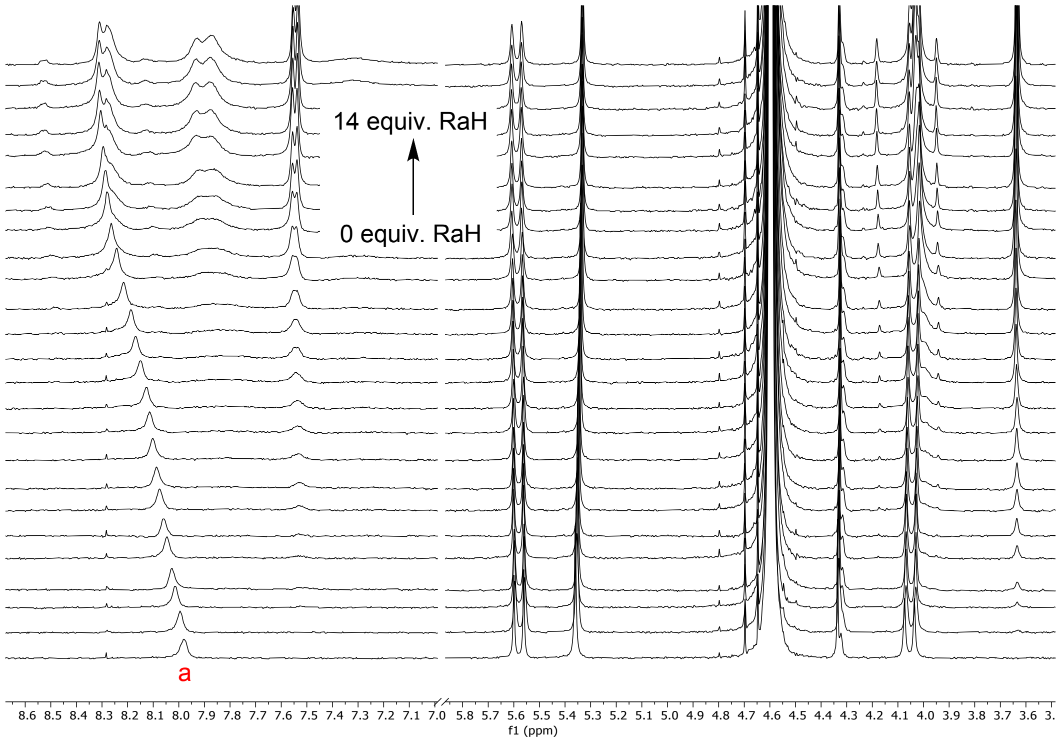

Figure S 164: ^{1}H NMR (400 MHz, D_2O) spectra of **MV**⊂CB[8] (500 uM) upon titration with $\mathbf{R_aH}$·2Cl (5000 uM) in 50mM phosphate buffer solution pD=12. The chemical shift of signals H_a were used for the fitting.

The chemical shifts (ppm) were selected using the automatic peak picking routine with standard settings implemented in the software Mestrenova (version 14.1.0). The mechanism proposed for the fitting process

equilibria, and introduced on the software Dynafit3 was the following (the association constant valuel of CB[8] with the MV is introduced as a fixed parameter)[7]:

$$\mathbf{MV}\subset CB[8] \Leftrightarrow \mathbf{MV} + CB[8]$$

$$\mathbf{R_a}^+ + CB[8] \Leftrightarrow \mathbf{R_a}^+\subset CB[8]$$

$$\mathbf{R_a}^+ + \mathbf{R_a}^+\subset CB[8] \Leftrightarrow 2\mathbf{R_a}^+\subset CB[8]$$

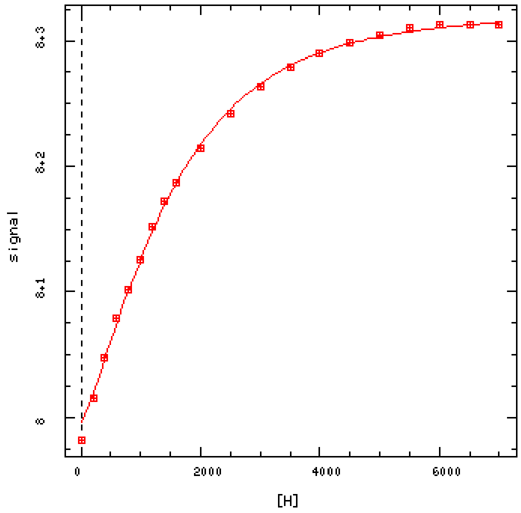

Figure S 165: Fitting of the experimental data of signals H_a.

Concentration (µM)	Chemical shift (ppm)	Residual
0	7.9814	0.00114506
200	8.0149	-0.0024886
400	8.0474	-0.0011375
600	8.0787	0.00182353
800	8.1016	-0.0013255
1000	8.1257	-0.001124
1200	8.1522	0.00356798
1400	8.172	0.00359903
1600	8.1862	3.01E-06
2000	8.2149	-0.0013448
2500	8.242	-0.0026967
3000	8.2634	-0.0017191
3500	8.2786	-0.0010599
4000	8.2899	-0.0001621
4500	8.2978	0.00019965
5000	8.3041	0.00094273
5500	8.3106	0.00327005
6000	8.3125	0.00197893

6500	8.3122	-0.0008042
7000	8.3123	-0.0026675

Table S 20: Experimental data for the titration. The *p* value obtained from “runs-of-sign” was 0.2.

8.4. pK_a determination for R_aH^{2+}⊂CB[8] by UV-Vis

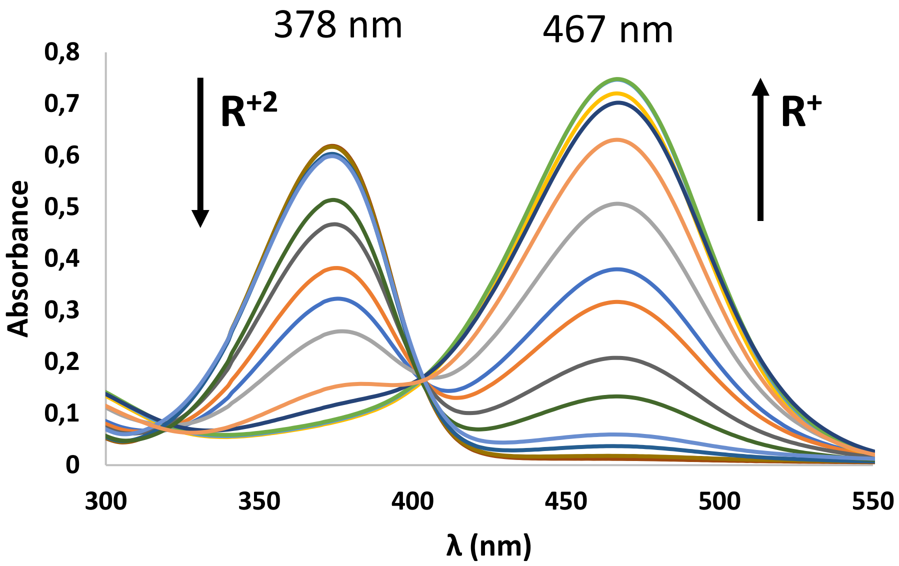

Figure S 166: UV-Vis spectra for the titration of **R_aH^{2+}**⊂CB[8] at 20 μM in NaH_2PO_4/Na_2HPO_4, $KHCO_3$/K_2CO_3 and Na_2HPO_4/Na_2PO_4 buffers.

pH (measured)	V (mL) of solution A	V (mL) of solution B	V (mL) of solution C	V (mL) of solution D	V (mL) of solution E	ABS (474 nm)	log[A_{474}-AR_b^{2+})/(AR_b^{1+}-A_{474})]
6.73 (**R_a^{2+}**)	1.125	1.125	-	-	-	0.01242	-
6.99	0.65	1.6	-	-	-	0.01796	-2.117
7.65	0.25	2.0	-	-	-	0.03661	-1.468
8.00	0.15	2.1	-	-	-	0.05925	-1.167
8.52	-	-	2.25	-	-	0.13337	-0.706
8.74	-	2.25	-	-	-	0.20795	-0.441
9.18	-	-	2.0	0.25	-	0.37957	-0.152
9.48	-	-	2.1	0.15	-	0.31652	0.022
9.72	-	-	1.75	0.50	-	0.50624	0.310
10.19	-	-	1.125	1.125	-	0.63054	0.723
11.00	-	0.80	-	-	1.45	0.70218	1.179
11.70	-	0.45	-	-	1.80	0.73510	1.829
12.27 (**R_a^{+}**)	-	-	-	-	2.25	0.74784	-

Table S 21: Experimental data obtained for the UV-Vis titration of of **R_aH**⊂CB[8] at 20 μM in NaH_2PO_4/Na_2HPO_4, $KHCO_3$/K_2CO_3 and Na_2HPO_4/Na_2PO_4 buffer. **Solution A**: 20 mM of NaH_2PO_4. **Solution B**: 20 mM of $NaHPO_4$. **Solution C**: 20 mM of Na_2PO_4. **Solution D**: 20 mM of $KHCO_3$. **Solution E**: 20 mM of K_2CO_3.

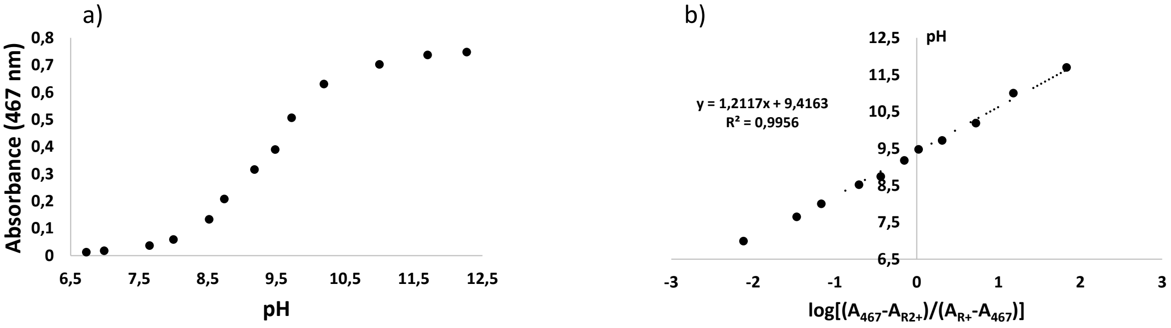

Figure S 167: a) Absorption of $\mathbf{R_a}^{+2}$ at λ = 467 nm plotted against pH. b) Linear fitting of pH plotted against log[(A_{467}-A_{R2+})/(A_{R+}-A467)], where p*Ka* value is 9.41.

8.5. Synthesis and characterization of the heteroternary complex:

To a equimolar mixture (1mM) solution of $\mathbf{R_aH}$·2Cl and HQ in D_2O with phosphate buffer solution different amounts in solid state of CB[8] were added until the solution reach saturation. Then, the mixture was sonicated and heated using an oil bath at 60 °C for 15 min. Finally, the sample was filtered off to remove the excess of CB[8].

- **R_aH^{+2}⊂CB[8]⊂HQ at pD = 7:**

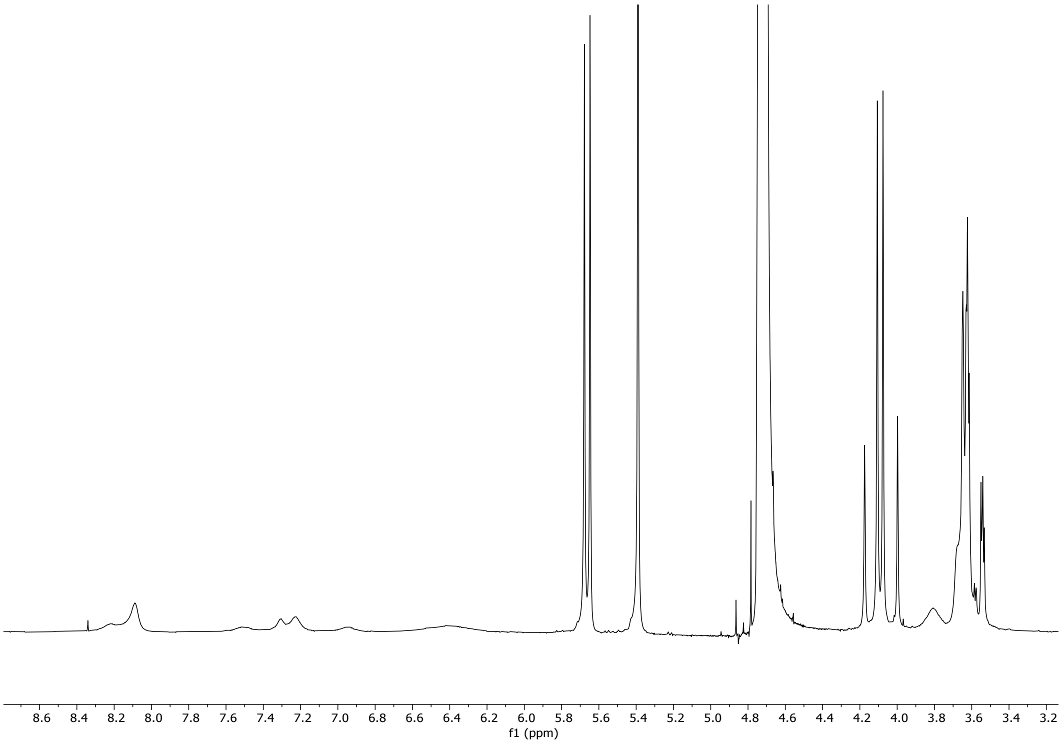

Figure S 168: ^{1}H NMR (500 MHz, D_2O) spectrum of $\mathbf{R_aH}$·2Cl + CB[8] + HQ at pD=7.

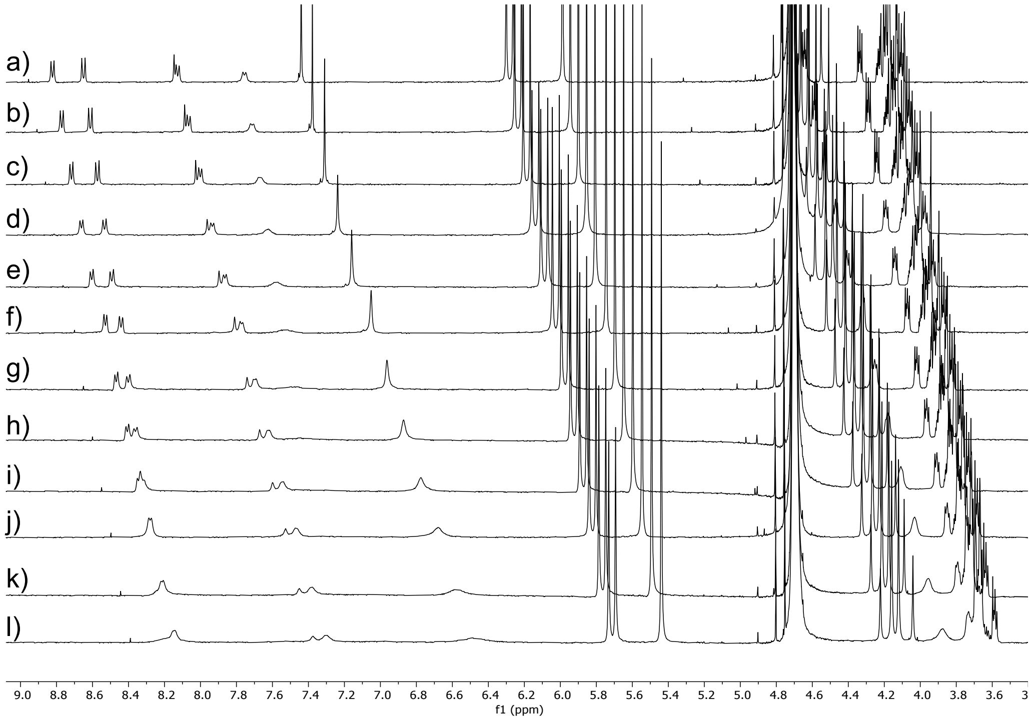

Figure S 169: T. Var. (400 MHz, D_2O, pD=7) spectrum of a mixture of $\mathbf{R_a}$·2Cl, CB[8] and HQ at: a) 353.15 K b) 348.15 K c) 343.15 K d) 338.15 K e) 333.15 K f) 328.15 K g) 323.15 K h) 318.15 K i) 313.15 K j) 308.15 K k) 303.15 K l) 298.15 K.

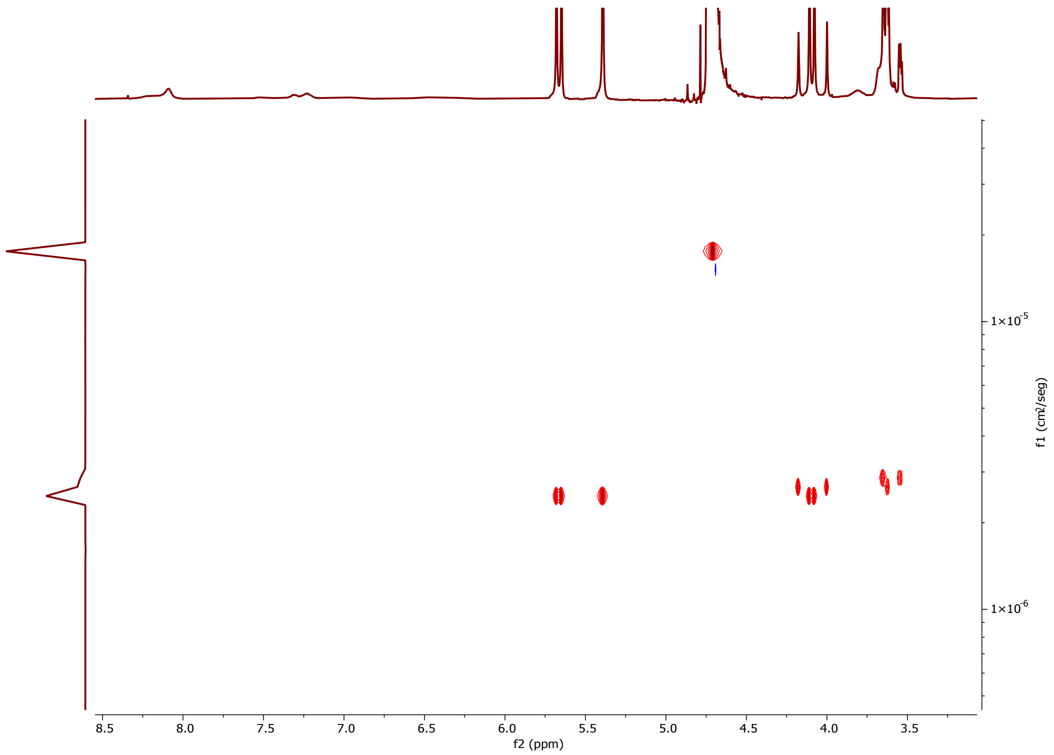

Figure S 170: DOSY (400 MHz, D_2O, 298 K) spectrum of $\mathbf{R_a}$·2Cl, CB[8] and HQ at pD=7.

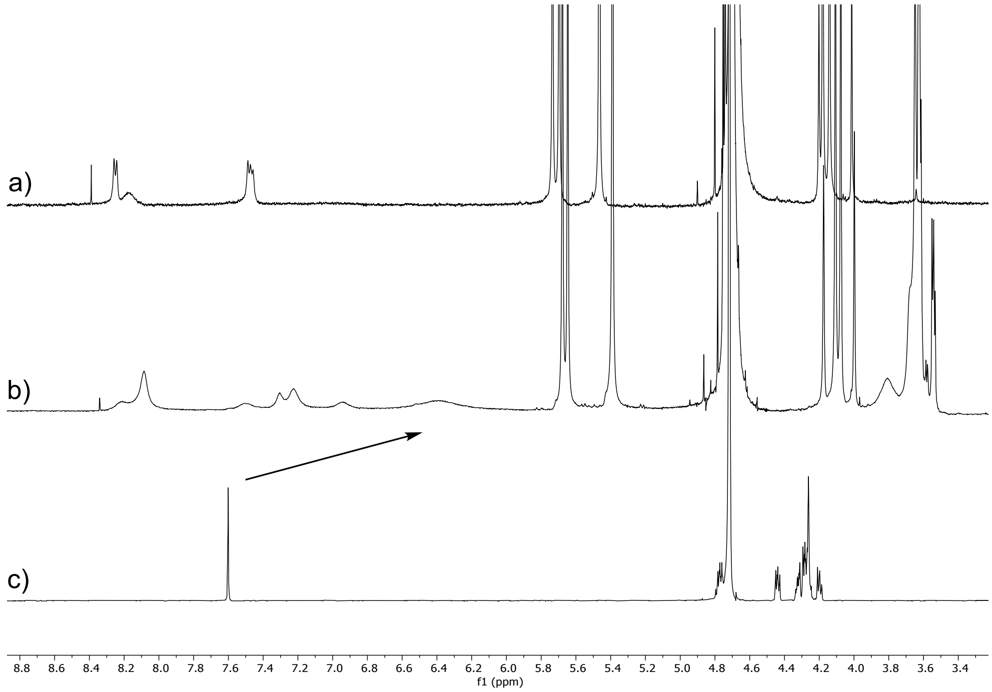

Figure S 171: ^{1}H-NMR (500 MHz, D_2O) of: a) solution of $\mathbf{R_aH}^{+2}$⊂CB[7] at pD=7, b) solution of $\mathbf{R_a}\cdot$2Cl, CB[8] and HQ at pD=7, c) solution of HQ.

- **R_a^+⊂CB[8]⊂HQ at pD = 12:**

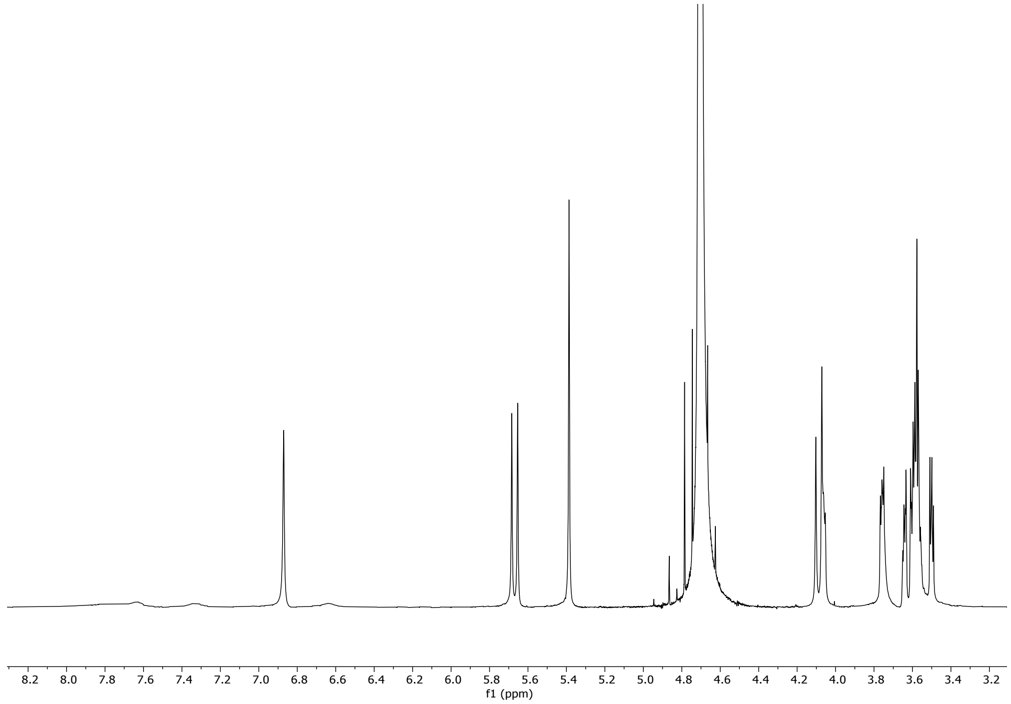

Figure S 172: ^{1}H NMR (500 MHz, D2O) spectrum of $\mathbf{R_a}\cdot$2Cl + CB[8] + HQ at pD=12.

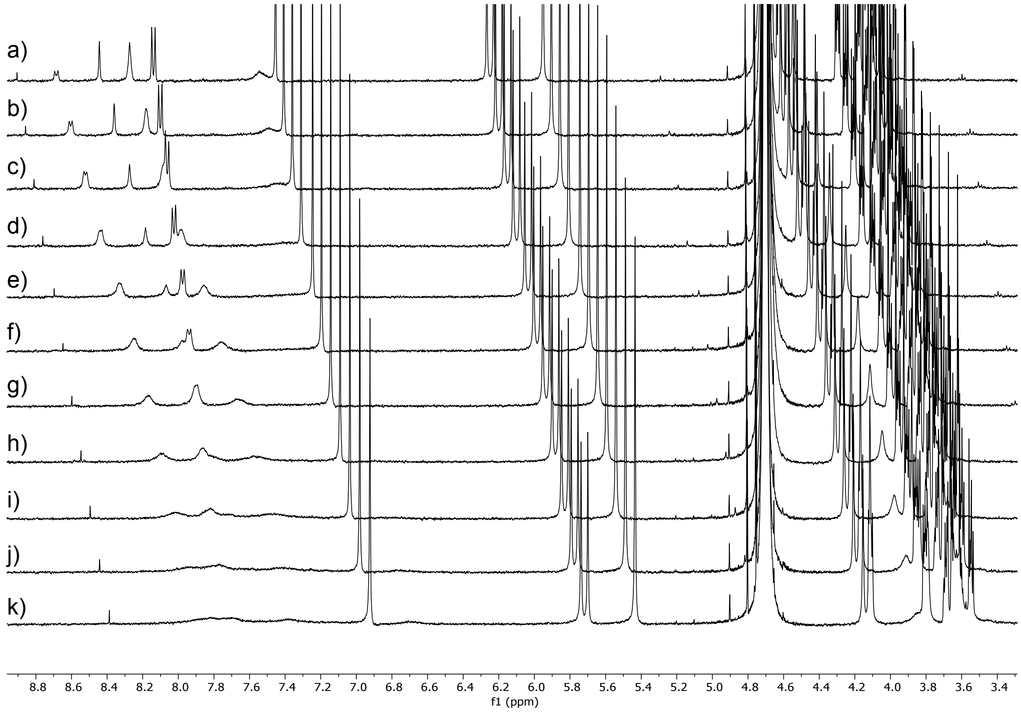

Figure S 173: T. Var. (400 MHz, D_2O, pD=12) spectrum of a mixture of $\mathbf{R_a}$·2Cl, CB[8] and HQ at: a) 348.15 K b) 343.15 K c) 338.15 K d) 333.15 K e) 328.15 K f) 323.15 K g) 318.15 K h) 313.15 K i) 308.15 K j) 303.15 K k) 298.15 K l) 308.15 K m) 303.15 K n) 298.15 K.

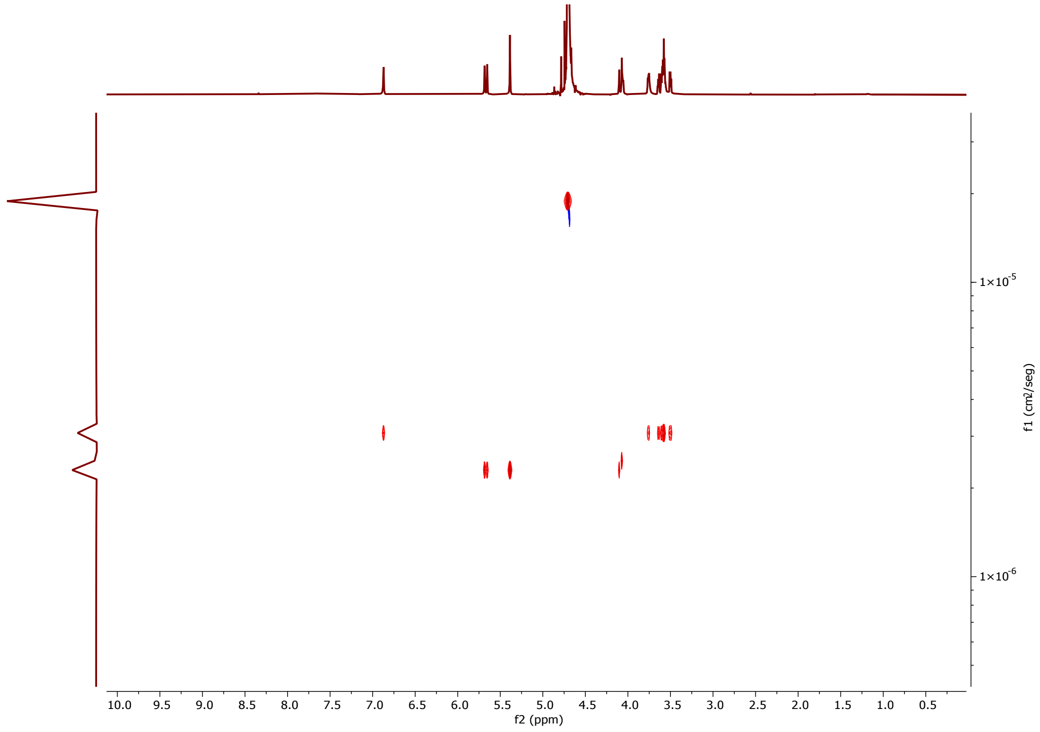

Figure S 174: DOSY (400 MHz, D_2O, 298 K) spectrum of $\mathbf{R_a}$·2Cl, CB[8] and HQ at pD=12.

9. X-ray crystallographic data.

Computing details.

Data collection: APPEX3v2018.7-2 (BRUKER AXS, 2005); cell refinement: APPEX3v2018.7-2 (BRUKER AXS, 2005); data reduction: APPEX3v2018.7-2 (BRUKER AXS, 2005); program(s) used to solve structure: *SHELXT2018*/2;[8] program(s) used to refine structure: *SHELXL2019*/1;[9] molecular graphics: *ORTEP* for *Windows*;[10] software used to prepare material for publication: *WinGX* publication routines.[10]

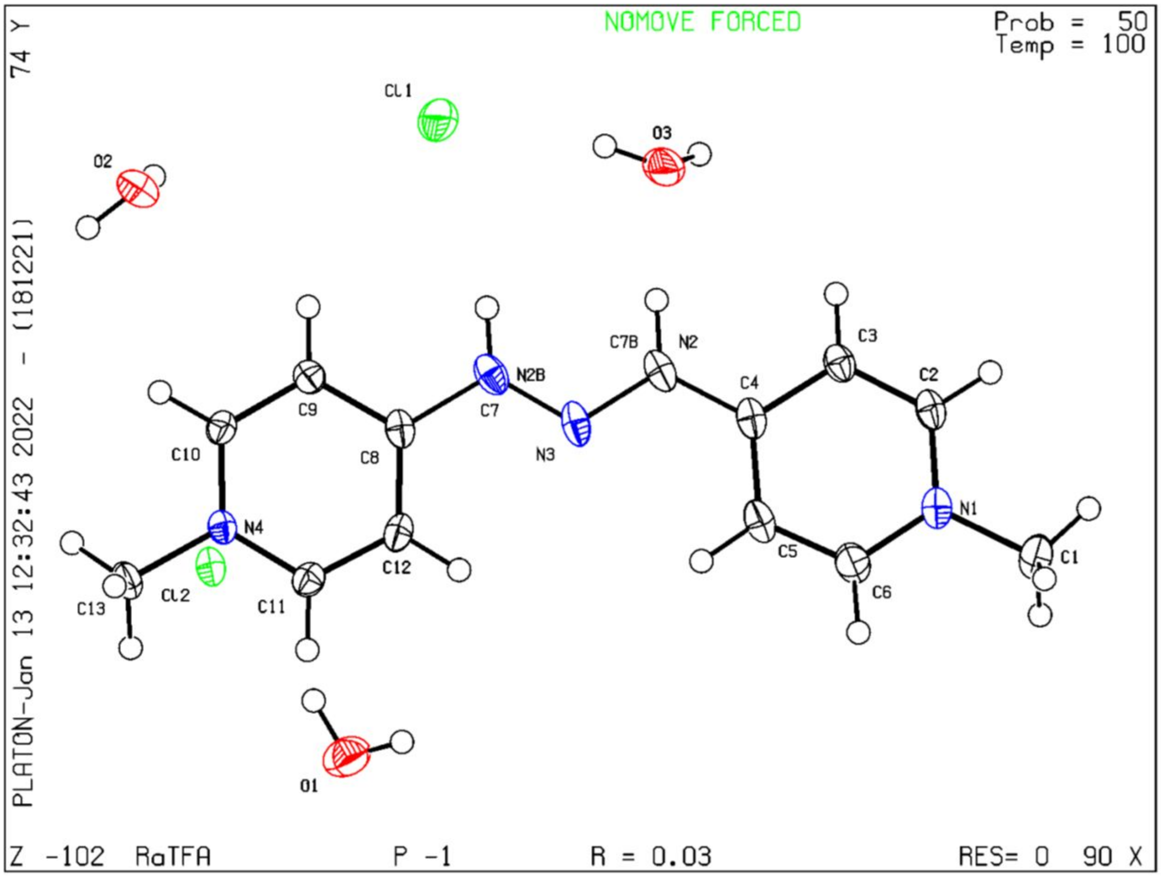

Figure S 175. *ORTEP* plot of the X-ray structure of $\mathbf{R_aH}$·2Cl. The displacement ellipsoids are shown at the 50% probability.

[8] Shelidrick, G.M. Crystal structure refinement with *SHELXL*. *Acta Cryst.* **2015**, *C71*, 3-8.
[9] Shelidrick, G.M. Aspherical scattering factors for *SHELXL* – model, implementation and application. *Acta Cryst.* **2019**, *A75*, 50-62
[10] Farrugia, L.J. *WinGX* and *ORTEP* for *Windows*: an update. *J. Appl. Cryst.* **2012**, *45*, 849-854.

Crystal data

$C_{13}H_{16}N_4 \cdot 2(Cl) \cdot 2.7(H_2O)$	$Z = 2$
$M_r = 347.84$	F(000) = 366
Triclinic, *P*	D_x = 1.354 Mg m−3
Hall symbol: -P 1	Mo *Kα* radiation, *λ* = 0.71073 Å
a = 6.6420 (4) Å	Cell parameters from 9211 reflections
b = 8.4800 (5) Å	*θ* = 2.5–28.3°
c = 16.086 (1) Å	*µ* = 0.40 mm−1
α = 80.356 (2)°	T = 100 K
β = 83.120 (2)°	Plate, yellow
γ = 73.355 (2)°	0.1 × 0.09 × 0.06 mm
V = 853.32 (9) Å3	

Data collection

Bruker D8 VENTURE PHOTON III-14 diffractometer	$T_{min} = 0.722$, $T_{max} = 0.795$
Radiation source: INCOATEC microfocus sealed tube, Incoatec IµS 3.0	54763 measured reflections
Incoatec multilayer mirror monochromator	4240 independent reflections
Detector resolution: 7.3910 pixels mm^{-1}	3685 reflections with $I > 2\sigma(I)$
ω and phi scans	$R_{int} = 0.045$
Absorption correction: multi-scan BRUKER *SADABS2016*/2	$\theta_{max} = 28.3°$, $\theta_{min} = 2.5°$
	$h = -8 \rightarrow 8$
	$k = -11 \rightarrow 11$
	$l = -21 \rightarrow 21$

Refinement

Refinement on F^2	0 constraints
Least-squares matrix: full	Primary atom site location: dual
$R[F^2 > 2\sigma(F^2)] = 0.034$	Secondary atom site location: dual
$wR(F^2) = 0.081$	Hydrogen site location: mixed
$S = 1.08$	H atoms treated by a mixture of independent and constrained refinement
4240 reflections	
226 parameters	$w = 1/[\sigma^2(F_o^2) + (0.0278P)^2 + 0.4691P]$ where $P = (F_o^2 + 2F_c^2)/3$
3 restraints	$(\Delta/\sigma)_{max} = 0.001$

$\Delta\rho_{max} = 0.31$ e Å^{-3} $\Delta\rho_{min} = -0.29$ e Å^{-3}

Special details.

Geometry. All e.s.d.'s (except the e.s.d. in the dihedral angle between two l.s. planes) are estimated using the full covariance matrix. The cell e.s.d.'s are taken into account individually in the estimation of e.s.d.'s in distances, angles and torsion angles; correlations between e.s.d.'s in cell parameters are only used when they are defined by crystal symmetry. An approximate (isotropic) treatment of cell e.s.d.'s is used for estimating e.s.d.'s involving l.s. planes.

10. Computational details:

All quantum mechanical calculations reported in this work were performed using the free-available program packages ORCA 5.0.3 (DFT), [11] and xTB 6.4.1 (semiempirical).[12]

Initial geometries for the different guests, CB[7,8] hosts, and the corresponding complexes were generated by hand using the AVOGADRO software,[13] and further optimized by using the GFN2-xTB semiempirical electronic structure method,[14] and the analytical linearized Poisson-Boltzmann (ALPB)[15] solvation model (water). Initial binding modes were obtained at the same level of theory by performing superquick conformational searches with the utility/driver for the xTB software CREST (conformer-rotamer sampling tool),[16] employing the non-covalent interactions mode (NCI). The lowest lying structures obtained in each case were subsequently employed for the estimation of the free energies at the dispersion-corrected DFT level of theory. Free energies for the H + G ⇌ G⊂H association processes were calculated following the supramolecular approach:

$$\Delta G^{\circ}_{aq} = G^{\circ}_{aq}\text{G⊂H} - G^{\circ}_{aq}\text{G} - G^{\circ}_{aq}\text{H} \text{ (eq. 1)}$$

where for each species X the free energy in aqueous solution was computed as:

$$G^{\circ}_{aq}(X) = \left[E^{DFT}_{gas}(\text{X}) + \delta_{\text{solv}}(X)\right] + G^{\circ}_{gas,\ mrrho}(X) \text{ (eq. 2)}$$

Consequently, each compound X was minimized using the composite electronic structure method r2scan-3c[17] in gas phase, checking the true nature of the structure as local minimum on the potential free energy surfaces by frequency calculations. Then, $E^{DFT}_{gas}(\text{X}) + \delta_{\text{solv}}(X)$ was evaluated together on each minimized structure by single point calculations using the dispersion-corrected ωB97X-D4 functional,[18] in conjunction with the large DEF2-QZVP[19] basis set and Thrular's solvation model SMD[20] to account for solvation effects in water. Energy to free energy thermoestatical contributions $G^{\circ}_{gas,\ mrrho}(X)$ were calculated by a modified rigid rotor harmonic

[11] Neese, F. Software update: The ORCA program system—Version 5.0. *WIREs Comput Mol Sci*. **2022**, 12, e1606.

[12] Bannwarth, C.; Caldeweyher, E.; Ehlert, S.; Hansen, A.; Pracht, P.; Seibert, J.; Spicher, S.; Grimme, S. Extended tight-binding quantum chemistry methods. *WIREs Comput. Mol. Sci.* **2021**, 11, e1493

[13] Hanwell, M. D.; Curtis, D. E.; Lonie, D. C.; Vandermeersch, T.; Zurek, E.; Hutchison, G. R. Avogadro: An advanced semantic chemical editor, visualization, and analysis platform. *J. Cheminform.* **2012**, *4*, 17.

[14] Bannwarth, C.; Ehlert, S.; Grimme, S. GFN2-xTB—An Accurate and Broadly Parametrized Self-Consistent Tight-Binding Quantum Chemical Method with Multipole Electrostatics and Density-Dependent Dispersion Contributions. *J. Chem. Theory Comput.* **2019**, *15*, 1652-1671.

[15] Ehlert, S.; Stahn, M.; Spicher, S.; Grimme, S. Robust and Efficient Implicit Solvation Model for Fast Semiempirical Methods. *J. Chem. Theory Comput.* **2021**, 17, 4250-4261.

[16] Pracht, P.; Bohle, F.; Grimme, S. Automated exploration of the low-energy chemical space with fast quantum chemical methods. *Phys. Chem. Chem. Phys.* **2020**, *22*, 7169-7192.

[17] Grimme, S.; Hansen, A.; Ehlert, S.; Mewes, J.-M. r2SCAN-3c: A "Swiss army knife" composite electronic-structure method" *J. Chem. Phys.* **2021**, *154*, 064103.

[18] Najibi, A.; Goerigk, L. DFT-D4 counterparts of leading meta-generalized-gradient approximation and hybrid density functionals for energetics and geometries. *J Comput Chem*. **2020**; *41*, 2562-2572.

[19] Weigend, F.; Ahlrichs, R. Balanced basis sets of split valence, triple zeta valence and quadruple zeta valence quality for H to Rn: Design and assessment of accuracy. *Phys. Chem. Chem. Phys.* 2005, **7**, 3297-3305.

[20] Marenich, A. V.; Cramer, C. J., Truhlar, D. G. Universal Solvation Model Based on Solute Electron Density and on a Continuum Model of the Solvent Defined by the Bulk Dielectric Constant and Atomic Surface Tensions. *J. Phys. Chem. B* **2009**, *113*, 6378-6396.

oscillator model, through single point hessian calculations at the GFN2-XTB level of theory on the r2scan-3c minimized structures.[21] The obtained results are compiled in **Table S1**

*Optimized structures **r2scan-3c/gas**. Single points at **wB97XD4/def2qzvp/SMD (H_2O)**. Thermostatistical corrections by Single point hessians SPH at GFN2-XTB level.*				
**SPECIES**	$\left[E_{gas}^{DFT}(\mathbf{X})+\delta_{\mathrm{solv}}(X)\right]$ **(ha)**	$G_{gas,\ mrrho}^{\circ}(X)$ **(ha)**	$G_{aq}^{\circ}(X)$ **(ha)**	
**HOSTS**				
**CB7**	-4215.878865	0.825462	-4215.053403	
**CB8**	-4818.144745	0.957695	-4817.187050	
**GUESTS**				
**R_aH^{2+}**	-724.317103	0.226710	-724.090393	
**R_a^+**	-723.842707	0.211737	-723.630971	
**HQ**	-461.678520	0.125548	-461.552972	
**MV^{2+}**	-575.4092327	0.189955	-575.219277	
**COMPLEXES**				ΔG_{aq}° (kcal/mol)
**$R_aH^{2+}\subset$CB7**	-4940.245181	1.088213	-4939.156968	**-8.3**
**$R_a^+\subset$CB7**	-4939.762160	1.073808	-4938.688352	**-2.5**
**$R_aH^{2+}\subset$CB8**	-5542.497729	1.212882	-5541.284847	**-4.6**
**$R_a^+\subset$CB8**	-5542.021217	1.199681	-5540.821536	**-2.2**
**mA_2$R_a^+\subset$CB8**	-6265.914460	1.440923	-6264.473538	-15.4
**mB_2$R_a^+\subset$CB8**	-6265.918424	1.442755	-6264.475670	-16.7
**mC_2$R_a^+\subset$CB8**	-6265.911722	1.439231	-6264.472491	-14.7
**mD_2$R_a^+\subset$CB8**	-6265.906926	1.439839	-6264.467087	-11.4
			**Boltzman Av =**	**-16.6**
**$R_aH^{2+}\cdot$HQ$\subset$CB8**	-6004.215114	1.363536	-6002.851578	**-13.3**
**$R_a^{+}\cdot$HQ$\subset$CB8**	-6003.738280	1.350208	-6002.388073	**-10.7**
**$MV^{2+}\subset$CB7**	-4791.341125	1.054579	-4790.286546	**-8.7**
**$MV^{2+}\subset$CB8**	-5393.593876	1.178462	-5392.415414	**-5.7**

Table S 22: Targeted quantities $\left[E_{gas}^{DFT}(\mathbf{X})+\delta_{\mathrm{solv}}(X)\right]$ and $G_{gas,\ mrrho}^{\circ}(X)$ used for the estimation of the free energies of binding in water for the inclusion complexes discussed in this work

Based on the optimized geometries at the r2scan-3c level of theory, and in order to qualitatively compare the binding modes of the complexes, two structural/geometric parameters were determined: the packing coefficients and displacements of the guest regarding the host. The former has been postulated by Nau *et al*, and is defined as the percentage of the volume occupied by the substrate in the volume of the CB7/8 inner cavity.[22] First, the substrate

[21] Spicher, S.; Grimme, S. Single-Point Hessian Calculations for Improved Vibrational Frequencies and Rigid-Rotor-Harmonic-Oscillator Thermodynamics. *J. Chem. Theory Comput.* **2021**, *17*, 1701-1714

[22] Nau, W. M; Florea, M.; Assaf, K. I. Deep Inside Cucurbiturils: Physical Properties and Volumes of their Inner Cavity Determine the Hydrophobic Driving Force for Host–Guest Complexation. *Isr. J. Chem.* **2011**, *51*, 559–577.

and CB surfaces were generated from the optimized structures using UCSF Chimera2 (version 1.15),[23] and a 0.5 Å radius probe. Next, the inner cavity of the CB was generated and the percentage of that volume occupied by the substrate was determined. These last operations were carried out with the help of Cinema 4D software (version R26.107) and the Vonc Suite plug-in. The results are summarized in **Table S 23**:

Complex	Inner Cavity Available Vol ($Å^3$)	Inner Cavity Occupied Vol ($Å^3$)	Packing Coefficient
mA_2$\mathbf{R_a}^+$@CB8	359	183	50.9%
mB_2$\mathbf{R_a}^+$@CB8	360	181	50.3%
mC_2$\mathbf{R_a}^+$@CB8	365	175	48.0%
mD_2$\mathbf{R_a}^+$@CB8	364	176	48.4%
$\mathbf{MV}^{2+}$@CB7	223	98	44.1%
$\mathbf{MV}^{2+}$@CB8	338	108	31.9%
$\mathbf{R_a}^+$@CB7	238	85	35.9%
$\mathbf{R_a}^+$@CB8	356	94	26.5%
$\mathbf{R_a}H^{2+}$@CB7	226	83	36.6%
$\mathbf{R_a}H^{2+}$@CB8	338	93	27.5%
$\mathbf{R_a}H^{2+}$·HQ@CB8	350	178	50.9%
$\mathbf{R_a}^+$·HQ@CB8	355	183	51.6%
CB7 (ref s12)	242		
CB8 (ref s12)	367		

Table S 23: Targeted quantities for the estimation of the packing coefficients for the inclusion complexes discussed in this work.

Regarding the **displacement** = $d_{(N1-p)}/d_{(N2-p)}$ parameter, that was intended to establish qualitative comparisons between the binding modes of the model vermellogen $\mathbf{R_a}H^{2+}/\mathbf{R_a}^+$ and those well-established for the paraquat⊂CB7/8 complexes, by calculating using the software Mercury 4.0[24] the ratio of the distances between the two nitrogen atoms of each guest (N1/N2) regarding the mean plane of the symmetric cucurbituril host (p):

[23] Pettersen, E.F.; Goddard, T.D.; Huang, C.C.; Couch, G.S.; Greenblatt, D.M.; Meng, E.C.; Ferrin, T. E. UCSF Chimera--a visualization system for exploratory research and analysis. *J. Comput Chem.* **2004**, *25*, 1605-1610.

[24] Macrae, C. F.; Sovago, I.; Cottrell, S. J.; Galek, P. T. A.; McCabe, P.; Pidcock, E.; Platings, M.; Shields, G. P.; Stevens, J. S.; Towler, M.; Wood, P. A. Mercury 4.0: from visualization to analysis, design and prediction. J. *Appl. Cryst.*, **2020**, *53*, 226-235.

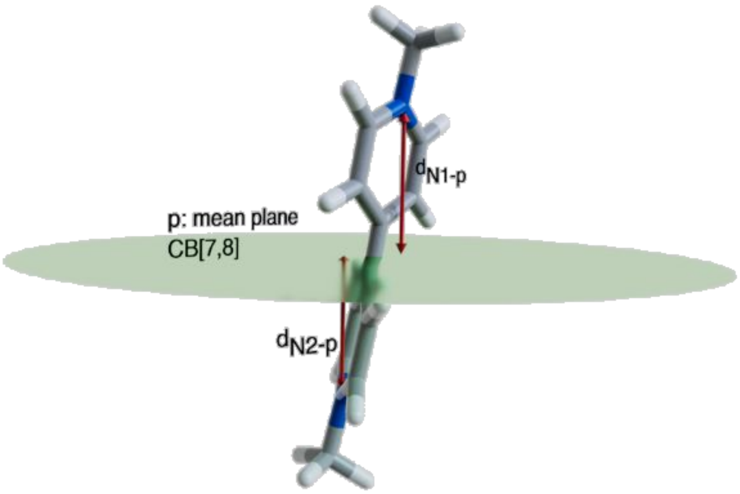

Figure S 176: Schematic representation of the targeted distances in order to obtain the displacement factor.

CARTESIAN COORDINATES

CB7: local minimum on the potential energy surface at the r2scan-3c level of theory

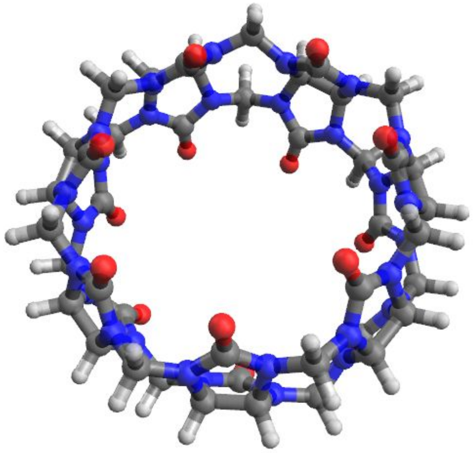

126

C	2.19903	-4.80276	-1.93782
N	0.92951	-4.92481	-1.25580
C	0.74728	-5.77441	-0.08946
C	-0.80067	-5.76688	0.09468
N	-1.28135	-5.25530	-1.16028
C	-0.26027	-4.75963	-1.96388
O	-0.38122	-4.31195	-3.08102
C	-2.66095	-5.27725	-1.59109
N	-3.49636	-4.21753	-1.07264
C	-4.04828	-4.18474	0.25725
C	-5.03924	-2.97838	0.20454
N	-4.72717	-2.34595	-1.06612
C	-3.93733	-3.16963	-1.87129
O	-3.71470	-3.02395	-3.05107
C	-5.60864	-1.37352	-1.68436
N	-5.39504	-0.00809	-1.27206
C	-5.84777	0.56068	-0.03178
C	-5.46128	2.06615	-0.17021
N	-4.54661	2.06284	-1.29895

C	-4.61174	0.86823	-2.01471
O	-4.12169	0.64732	-3.09816
C	-4.12523	3.27774	-1.96146
N	-3.00963	3.93883	-1.32183
C	-3.18245	4.86482	-0.21739
C	-1.78888	5.56237	-0.11974
N	-1.13483	5.16054	-1.33552
C	-1.84905	4.20428	-2.04713
O	-1.54194	3.72928	-3.11622
C	0.15038	5.64019	-1.77984
N	1.29200	5.02834	-1.12533
C	1.84018	5.54559	0.11862
C	3.22736	4.83520	0.21548
N	3.39386	4.25233	-1.09007
C	2.27402	4.40467	-1.89876
O	2.18410	4.09483	-3.06431
C	4.62367	3.67378	-1.58275
N	4.91778	2.33658	-1.11748
C	5.48016	2.01551	0.16754
C	5.85255	0.50656	0.02913
N	5.14363	0.10698	-1.17860
C	4.74358	1.21925	-1.92473
O	4.35943	1.21768	-3.07151
C	5.43393	-1.13761	-1.86831
N	4.69529	-2.28042	-1.39350
C	5.01141	-3.02502	-0.20434
C	4.00952	-4.22243	-0.25484
N	3.10489	-3.85414	-1.32763
C	3.58259	-2.77114	-2.06529
O	3.14089	-2.36370	-3.11493
N	3.45772	-4.24812	1.07531
C	2.61210	-5.29891	1.59529
N	1.23282	-5.26429	1.16421
C	0.21649	-4.75717	1.96665
O	0.34171	-4.30807	3.08274
N	-0.97489	-4.91277	1.25898
C	-2.24300	-4.77821	1.94133
N	-3.14072	-3.82273	1.32976
C	-3.61010	-2.73548	2.06656
O	-3.16567	-2.33096	3.11618
N	-4.71847	-2.23620	1.39393
C	-5.44744	-1.08650	1.86709
N	-5.14477	0.15513	1.17739
C	-4.73522	1.26386	1.92353
O	-4.35309	1.25919	3.07098
N	-4.89711	2.38233	1.11531
C	-4.59051	3.71687	1.58028
N	-3.35501	4.28349	1.08802
C	-2.23369	4.42424	1.89678
O	-2.14685	4.11281	3.06214
N	-1.24552	5.03866	1.12370
C	-0.09832	5.63941	1.77879
N	1.18239	5.14842	1.33394
C	1.88849	4.18601	2.04536
O	1.57736	3.71339	3.11434
N	3.04680	3.91091	1.32007
C	4.15658	3.23979	1.95937
N	4.56645	2.02083	1.29709
C	4.62213	0.82617	2.01359
O	4.13156	0.61024	3.09781
N	5.39683	-0.05742	1.27052
C	5.59859	-1.42460	1.68300
N	4.70739	-2.38917	1.06650
C	3.90988	-3.20418	1.87285

O	3.68963	-3.05541	3.05270	H	5.46154	4.33352	-1.30521
H	1.98490	-4.44800	-2.95361	H	6.35193	2.66209	0.37570
H	2.68999	-5.79330	-1.98404	H	6.93715	0.33505	-0.09333
H	1.16157	-6.78144	-0.27837	H	6.51269	-1.35875	-1.78827
H	-1.22434	-6.76953	0.28611	H	5.16115	-0.98871	-2.92030
H	-3.09679	-6.24771	-1.30450	H	6.06978	-3.34297	-0.22335
H	-2.66233	-5.17240	-2.68270	H	4.49698	-5.18778	-0.48366
H	-4.54468	-5.14522	0.48741	H	3.03844	-6.27406	1.31030
H	-6.10047	-3.28676	0.22182	H	2.61449	-5.19235	2.68673
H	-5.42250	-1.41975	-2.76427	H	-2.74262	-5.76433	1.98978
H	-6.65783	-1.64083	-1.47142	H	-2.02531	-4.42331	2.95631
H	-6.93411	0.39910	0.08882	H	-5.17500	-0.93959	2.91945
H	-6.32682	2.72080	-0.37916	H	-6.52800	-1.29797	1.78541
H	-4.98002	3.97799	-2.02144	H	-4.50176	3.66246	2.67198
H	-3.80279	2.99996	-2.97266	H	-5.42184	4.38452	1.30202
H	-4.00731	5.56773	-0.43477	H	-0.18307	5.40421	2.84672
H	-1.85617	6.66423	-0.06859	H	-0.12332	6.73304	1.63164
H	0.23304	5.40529	-2.84801	H	5.01776	3.93223	2.01868
H	0.18533	6.73338	-1.63159	H	3.83213	2.96522	2.97080
H	1.91763	6.64685	0.06870	H	6.64497	-1.70149	1.46854
H	4.05878	5.53048	0.43238	H	5.41372	-1.46874	2.76322
H	4.53380	3.61992	-2.67439				

CB8: local minimum on the potential energy surface at the r2scan-3c level of theory

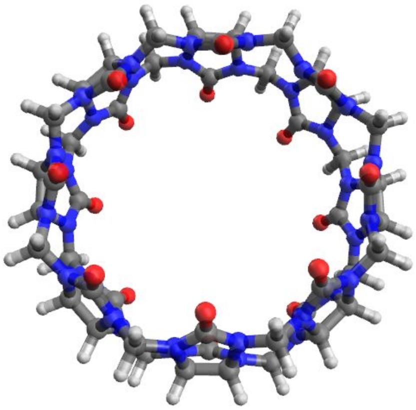

144

C	-6.10717	-2.50282	-0.12290
H	-7.20488	-2.63135	-0.09206
N	-5.72160	-1.80756	-1.32200
C	-4.75967	-2.48676	-2.05875
O	-4.32013	-2.15993	-3.13760
N	-4.43261	-3.63787	-1.34296
C	-3.74529	-4.72590	-2.00252
N	-2.53003	-5.14499	-1.33879
C	-1.33468	-5.20689	-2.05495
O	-1.11446	-4.70717	-3.13460
N	-0.45783	-5.99273	-1.31819
C	0.85874	-6.32810	-1.79921
N	1.94400	-5.63505	-1.13201
C	2.89268	-4.94940	-1.89305
O	2.79474	-4.64253	-3.05932
N	3.99072	-4.72484	-1.07213
C	5.21293	-4.13801	-1.57192
N	5.50943	-2.81369	-1.07525
C	5.47244	-1.69378	-1.89635
O	5.14707	-1.66942	-3.06156
N	5.92343	-0.61237	-1.13730
C	6.34595	0.60267	-1.80715
N	5.72103	1.80767	-1.32235
C	4.75895	2.48710	-2.05866
O	4.31938	2.16072	-3.13764
N	4.43229	3.63819	-1.34263
C	3.74601	4.72680	-2.00241
N	2.53064	5.14646	-1.33944
C	1.33524	5.20782	-2.05542
O	1.11527	4.70853	-3.13533
N	0.45804	5.99292	-1.31825
C	-0.85851	6.32820	-1.79929
N	-1.94370	5.63495	-1.13211
C	-2.89248	4.94979	-1.89349
O	-2.79403	4.64225	-3.05955
N	-3.99084	4.72568	-1.07293
C	-5.21226	4.13709	-1.57248
N	-5.50701	2.81267	-1.07499
C	-5.47233	1.69292	-1.89649
O	-5.14791	1.66839	-3.06196
N	-5.92597	0.61243	-1.13797
C	-6.51763	1.04723	0.11591
H	-7.61566	0.92303	0.08066
N	-5.98937	0.45742	1.31701
C	-6.32648	-0.85829	1.79938
N	-5.63512	-1.94483	1.13285
C	-4.94873	-2.89309	1.89350
O	-4.64176	-2.79523	3.05977
N	-4.72259	-3.99032	1.07188
C	-4.13751	-5.21327	1.57195
N	-2.81343	-5.51139	1.07570
C	-1.69319	-5.47301	1.89622
O	-1.66879	-5.14834	3.06161
N	-0.61165	-5.92350	1.13683
C	0.60284	-6.34726	1.80709
N	1.80851	-5.72318	1.32293
C	2.48706	-4.75995	2.05855
O	2.16068	-4.32051	3.13758
N	3.63814	-4.43315	1.34253
C	4.72667	-3.74687	2.00249
N	5.14636	-2.53143	1.33977
C	5.20704	-1.33594	2.05558

O	4.70718	-1.11590	3.13521	H	5.99103	4.71927	-0.42965
N	5.99060	-0.45798	1.31769	C	6.10666	2.50238	-0.12298
C	6.32662	0.85823	1.79934	H	7.20440	2.63052	-0.09184
N	5.63413	1.94400	1.13248	C	6.51738	-1.04721	0.11576
C	4.94828	2.89254	1.89339	H	7.61524	-0.92218	0.07866
O	4.64128	2.79459	3.05963	C	6.07206	-2.54021	0.22201
N	4.72323	3.99034	1.07223	H	6.91067	-3.23123	0.42639
C	4.13710	5.21282	1.57218	C	3.85511	-5.33393	0.22550
N	2.81299	5.51020	1.07546	H	4.71959	-5.99209	0.43023
C	1.69274	5.47261	1.89606	C	2.50283	-6.10809	0.12319
O	1.66814	5.14762	3.06136	H	2.63123	-7.20577	0.09138
N	0.61148	5.92386	1.13676	C	-1.04701	-6.51829	-0.11577
C	-0.60299	6.34777	1.80693	H	-0.92266	-7.61620	-0.07787
N	-1.80879	5.72386	1.32286	C	-2.53969	-6.07227	-0.22216
C	-2.48698	4.76020	2.05821	H	-3.23089	-6.91035	-0.42804
O	-2.16046	4.32064	3.13714	C	-5.33261	-3.85496	-0.22544
N	-3.63754	4.43237	1.34164	H	-5.99068	-4.71964	-0.42960
C	-4.72627	3.74686	2.00236	C	-6.34773	-0.60317	-1.80700
N	-5.14681	2.53149	1.34030	H	-3.46586	-4.37523	-3.00353
C	-5.20668	1.33569	2.05548	H	-4.42988	-5.59156	-2.08854
O	-4.70692	1.11563	3.13517	H	0.90272	-6.04413	-2.85769
C	-6.07138	2.54012	0.22179	H	1.00297	-7.41874	-1.69798
H	-6.90978	3.23175	0.42489	H	6.05458	-4.80615	-1.32229
C	-3.85501	5.33384	0.22510	H	5.10869	-4.05670	-2.66062
H	-4.71950	5.99176	0.43050	H	6.07235	0.49494	-2.86378
C	-2.50292	6.10827	0.12283	H	7.44142	0.71130	-1.71305
H	-2.63155	7.20591	0.09071	H	3.46700	4.37652	-3.00368
C	1.04708	6.51854	-0.11580	H	4.43118	5.59206	-2.08784
H	0.92267	7.61644	-0.07792	H	-0.90244	6.04426	-2.85778
C	2.53980	6.07253	-0.22193	H	-1.00294	7.41882	-1.69802
H	3.23117	6.91078	-0.42658	H	-5.10779	4.05535	-2.66113
C	5.33272	3.85483	-0.22527	H	-6.05474	4.80442	-1.32343

H	-7.41738	-1.00117	1.69868
H	-6.04225	-0.90182	2.85781
H	-4.80655	-6.05417	1.32223
H	-4.05635	-5.10911	2.66068
H	0.71067	-7.44278	1.71262
H	0.49494	-6.07381	2.86374
H	5.59197	-4.43200	2.08791
H	4.37625	-3.46799	3.00376
H	7.41733	1.00201	1.69805
H	6.04257	0.90196	2.85780
H	4.80572	6.05414	1.32272
H	4.05563	5.10846	2.66086
H	-0.49518	6.07441	2.86361
H	-0.71064	7.44330	1.71247
H	-5.59113	4.43251	2.08793
H	-4.37562	3.46818	3.00360
H	-6.07475	-0.49555	-2.86380
H	-7.44304	-0.71270	-1.71217

RaH^{2+}: local minimum on the potential energy surface at the r2scan-3c level of theory

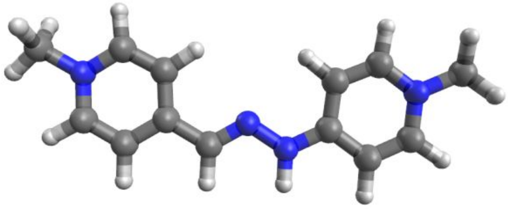

33

C	-6.17891	-1.26399	-0.00974
C	6.21678	-1.23825	0.03707
C	3.55031	1.37378	-0.04697
C	4.78409	0.76158	-0.03657
N	4.88630	-0.58383	0.00324
C	3.77139	-1.35691	0.03760
C	2.51983	-0.79588	0.02907
C	2.37969	0.60248	-0.01499
C	1.08963	1.26815	-0.02950
N	-0.00049	0.57692	-0.01090
N	-1.15455	1.24984	-0.02483
C	-2.47447	-0.79490	0.01667
C	-2.36312	0.60564	-0.01145
C	-3.54304	1.37115	-0.02362
C	-4.75832	0.73972	-0.00732
N	-4.84810	-0.61189	0.02357
C	-3.72149	-1.36415	0.03159
H	1.09030	2.36519	-0.05705
H	-6.89877	-0.63632	0.51736
H	-6.11630	-2.23297	0.48682
H	-6.49234	-1.39786	-1.04934
H	6.98748	-0.49741	-0.17407
H	6.24844	-2.02391	-0.72090
H	6.37998	-1.66795	1.02947
H	3.51165	2.45784	-0.08216
H	5.71218	1.32241	-0.06062
H	3.93214	-2.42969	0.07101
H	1.65079	-1.44215	0.05545
H	-1.15491	2.26984	-0.04672
H	-1.59777	-1.42871	0.02907

H	-3.51958	2.45621	-0.04283
H	-5.69354	1.28948	-0.01645
H	-3.85987	-2.43998	0.05228

Ra^{+}: local minimum on the potential energy surface at the r2scan-3c level of theory

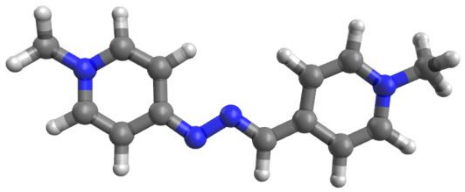

32			
C	6.10410	1.27057	0.07739
C	-6.12729	1.26875	-0.00609
C	-3.52074	-1.38555	-0.00934
C	-4.73068	-0.75275	0.01151
N	-4.81563	0.60459	0.03889
C	-3.67317	1.35147	0.03459
C	-2.44155	0.76869	0.01411
C	-2.31161	-0.64517	-0.00931
C	-1.06155	-1.31466	-0.02935
N	0.05899	-0.62331	-0.02387
N	1.14767	-1.36838	-0.05010
C	2.41787	0.76774	0.02351
C	2.29674	-0.65064	-0.03174
C	3.50903	-1.38842	-0.07189
C	4.71348	-0.75107	-0.05696
N	4.79419	0.60905	-0.00951
C	3.65010	1.34923	0.03599
H	-1.03767	-2.40846	-0.04960
H	6.85514	0.65374	-0.41904
H	6.38813	1.41577	1.12509
H	6.05733	2.23910	-0.42421
H	-6.88211	0.60937	0.42516
H	-6.39685	1.50290	-1.04130
H	-6.08856	2.19042	0.57783
H	-3.49980	-2.47014	-0.02333
H	-5.67171	-1.29203	0.01075
H	-3.81004	2.42749	0.05138
H	-1.55508	1.39076	0.01780
H	1.53191	1.38843	0.05368
H	3.46727	-2.47078	-0.11776
H	5.65725	-1.28491	-0.08541
H	3.78587	2.42518	0.07916

HQ: local minimum on the potential energy surface at the r2scan-3c level of theory

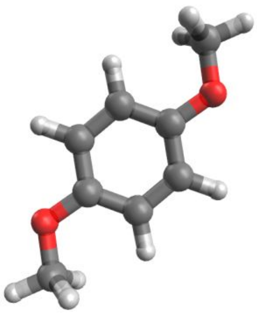

20			
C	1.20042	0.08064	0.68376
O	2.23645	-0.05035	-0.27952
C	2.74445	1.10333	-0.81585
C	2.31388	2.39115	-0.48614
C	2.90156	3.49390	-1.09445
C	3.91899	3.33270	-2.03268
O	4.42718	4.48639	-2.56882
C	5.46126	4.35526	-3.53417
C	4.34959	2.04488	-2.36237
C	3.76185	0.94213	-1.75412
H	0.93849	-0.93585	0.98299
H	0.31420	0.57243	0.25859
H	1.53923	0.64174	1.56607
H	1.52517	2.55364	0.23929
H	2.57479	4.49880	-0.84598
H	5.72231	5.37168	-3.83438
H	5.12078	3.79363	-4.41549
H	6.34854	3.86395	-3.11062
H	5.13836	1.88239	-3.08773
H	4.08861	-0.06278	-2.00260

MV^{2+}: local minimum on the potential energy surface at the r2scan-3c level of theory

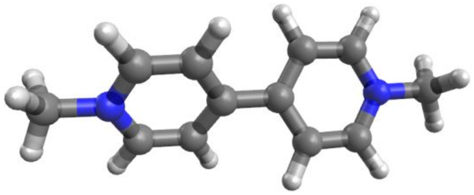

28			
C	-4.99568	-0.01237	0.01824
N	-3.50845	0.00493	-0.01108
C	-2.84034	1.09993	0.41041
C	-1.46082	1.12489	0.41930
C	-2.83899	-1.09378	-0.42326
C	-1.46023	-1.12133	-0.42315
C	-0.74024	0.00189	-0.00058
C	0.74021	0.00234	-0.00050
C	1.46011	1.12495	-0.42108
C	2.84018	1.10075	-0.41218
N	3.50839	0.00667	0.00973
C	4.99578	-0.01449	-0.00979
C	2.83907	-1.09233	0.42315

C	1.46087	-1.12065	0.42308
H	-5.36670	1.01010	-0.05618
H	-5.32495	-0.46299	0.95948
H	-5.36190	-0.59822	-0.82603
H	-3.44001	1.94307	0.73784
H	-0.96499	2.01905	0.78285
H	-3.43922	-1.93566	-0.75356
H	-0.96347	-2.01504	-0.78659
H	0.96408	2.01857	-0.78566
H	3.43857	1.94438	-0.74027
H	5.36790	1.00848	-0.06236
H	5.32969	-0.57882	-0.88560
H	5.35619	-0.49083	0.90375
H	3.44064	-1.93319	0.75414
H	0.96430	-2.01430	0.78691

RaH^{2+}⊂CB7: local minimum on the potential energy surface at the r2scan-3c level of theory

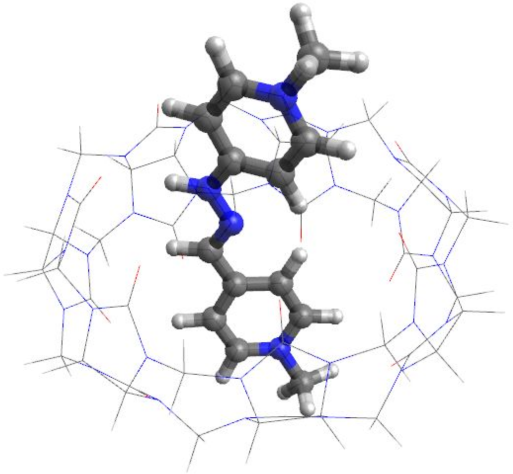

159

C	-1.88704	-0.39633	6.52785
C	-1.24031	1.01435	-5.16303
C	1.45649	0.29438	-2.70260
C	0.79518	0.67643	-3.84534
N	-0.54251	0.52593	-3.95729
C	-1.26748	-0.00781	-2.94861
C	-0.65809	-0.37297	-1.77490
C	0.72688	-0.20909	-1.61849
C	1.39031	-0.50443	-0.35784
N	0.65203	-0.59034	0.69579
N	1.20245	-0.82313	1.88457
C	0.97715	-1.06885	4.25116
C	0.42428	-0.72529	3.00395
C	-0.90434	-0.27887	2.95034
C	-1.63034	-0.19328	4.10553
N	-1.09060	-0.54337	5.29646
C	0.19734	-0.97116	5.36785
H	2.48608	-0.58185	-0.34185
H	-1.72987	0.59873	6.95539
H	-1.58464	-1.15861	7.24773
H	-2.94219	-0.52925	6.28552
H	-0.61082	0.84023	-6.03721
H	-1.43740	2.08219	-5.03534

H	-2.18327	0.47710	-5.26683
H	2.53416	0.41667	-2.67213
H	1.31214	1.12016	-4.68842
H	-2.33077	-0.12933	-3.12702
H	-1.25717	-0.79859	-0.97840
H	1.99649	-1.43090	4.32521
H	-1.36776	0.01160	2.01802
H	-2.65677	0.15523	4.10134
H	0.56324	-1.23389	6.35401
C	-3.45318	4.24154	-2.00473
N	-2.16508	4.56317	-1.44703
C	-1.97860	5.27569	-0.20774
C	-0.43960	5.52657	-0.17284
N	0.05685	4.71955	-1.27879
C	-0.97467	4.19541	-2.04709
O	-0.85361	3.55977	-3.07825
C	1.36472	4.93845	-1.86095
N	2.43915	4.25770	-1.17053
C	3.04590	4.79302	0.04246
C	4.32315	3.91523	0.19323
N	4.44892	3.27717	-1.09283
C	3.32472	3.45864	-1.88674
O	3.15518	3.02240	-3.00651
C	5.61817	2.55255	-1.54263
N	5.71937	1.17270	-1.10226
C	6.13415	0.73453	0.20743
C	6.18092	-0.82112	0.06433
N	5.42896	-1.04915	-1.16370
C	5.27282	0.12861	-1.89460
O	4.84362	0.21637	-3.02480
C	5.41025	-2.32706	-1.86186
N	4.33818	-3.21307	-1.47498
C	4.33431	-4.01740	-0.28305
C	2.99051	-4.80262	-0.38502
N	2.28547	-4.11048	-1.45202
C	3.10976	-3.20741	-2.12016
O	2.81740	-2.55918	-3.10186
C	1.17015	-4.71230	-2.15609
N	-0.10330	-4.54298	-1.49277
C	-0.50894	-5.38960	-0.38113
C	-2.03661	-5.11135	-0.27417
N	-2.33725	-4.43259	-1.50655
C	-1.19857	-4.06396	-2.20911
O	-1.16417	-3.46226	-3.26077
C	-3.67117	-4.20298	-2.01118
N	-4.37427	-3.08238	-1.42288
C	-5.02468	-3.09142	-0.13583
C	-5.82925	-1.75182	-0.13405
N	-5.30756	-1.05086	-1.29937
C	-4.53239	-1.88704	-2.09835
O	-4.09864	-1.62242	-3.20145
C	-5.99228	0.07912	-1.91011
N	-5.52075	1.37311	-1.47915
C	-5.80963	1.95051	-0.18904
C	-4.99903	3.28344	-0.21079
N	-4.13231	3.12385	-1.37176
C	-4.48667	2.01980	-2.13533
O	-4.00296	1.70224	-3.20468
N	-4.33376	3.28247	1.06795
C	-3.54589	4.37906	1.58298

N	-2.22206	4.50743	1.00239	N	-5.25905	1.24607	0.96202
C	-1.10397	4.49207	1.84098	C	-4.44683	2.07621	1.73351
O	-1.06709	4.07488	2.97668	O	-3.95922	1.79998	2.81169
N	-0.05955	5.07747	1.14111	H	-3.29802	3.97231	-3.05533
C	1.22254	5.34602	1.75230	H	-4.09468	5.13566	-1.94381
N	2.32287	4.54510	1.26320	H	-2.57434	6.20404	-0.20414
C	2.83840	3.47786	1.98147	H	-0.16457	6.58495	-0.31859
O	2.41175	3.02996	3.02301	H	1.57031	6.02396	-1.88074
N	3.96856	3.03622	1.29405	H	1.34354	4.55284	-2.88637
C	4.93145	2.18744	1.94698	H	3.26039	5.86890	-0.07078
N	5.15317	0.92137	1.27112	H	5.23135	4.49521	0.42842
C	4.81903	-0.26753	1.88290	H	5.58528	2.53082	-2.63763
O	4.03584	-0.40750	2.81148	H	6.51568	3.09127	-1.20843
N	5.54070	-1.27641	1.27683	H	7.10100	1.18281	0.48469
C	5.31756	-2.66040	1.63437	H	7.20127	-1.22711	-0.01357
N	4.18946	-3.28605	0.97005	H	6.36670	-2.84789	-1.70314
C	3.11206	-3.75561	1.72096	H	5.28238	-2.10559	-2.92775
O	2.84767	-3.44411	2.86554	H	5.22224	-4.66925	-0.24730
N	2.41903	-4.65207	0.93324	H	3.12435	-5.86922	-0.63051
C	1.28037	-5.39391	1.43305	H	1.09415	-4.22397	-3.13462
N	-0.00380	-4.99231	0.90798	H	1.37295	-5.78970	-2.29777
C	-0.94901	-4.31881	1.66836	H	-0.26849	-6.44704	-0.58429
O	-0.78460	-3.85945	2.78002	H	-2.64591	-6.02531	-0.17486
N	-2.13051	-4.29833	0.93040	H	-3.58217	-3.99611	-3.08360
C	-3.39658	-4.04827	1.58547	H	-4.26747	-5.11419	-1.85544
N	-4.14621	-2.94692	1.01672	H	-5.66039	-3.98555	-0.02791
C	-4.49035	-1.85643	1.80098	H	-6.91762	-1.89584	-0.22859
O	-4.01460	-1.57011	2.88266	H	-7.06931	0.00840	-1.69729
N	-5.50657	-1.17781	1.14990	H	-5.82468	0.01998	-2.99139
C	-5.94452	0.12571	1.59045	H	-6.89439	2.09899	-0.06687

H	-5.62970	4.18056	-0.32019	H	6.23754	-3.22283	1.41695
H	-4.10918	5.31286	1.42466	H	1.25377	-5.24317	2.51798
H	-3.41074	4.20658	2.65684	H	1.43207	-6.46126	1.21003
H	1.47389	6.40778	1.60503	H	-4.01362	-4.96370	1.55187
H	1.11465	5.13550	2.82251	H	-3.17963	-3.79108	2.62865
H	4.54165	1.96525	2.94728	H	-7.02526	0.21559	1.40445
H	5.89795	2.71315	2.03405	H	-5.75062	0.18009	2.66730
H	5.11600	-2.69147	2.71076	H	2.21255	-0.94974	2.01780

$\mathbf{R_a}^+ \subset$CB7: local minimum on the potential energy surface at the r2scan-3c level of theory.

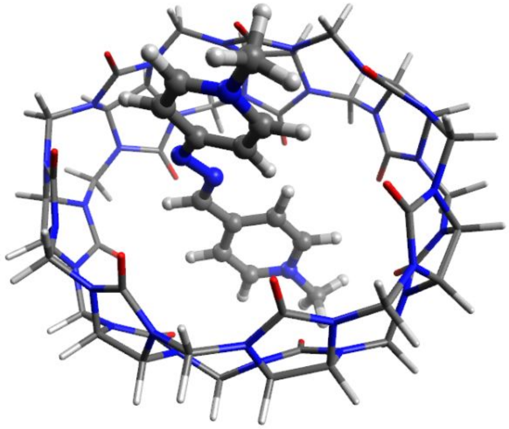

158

				N	1.75231	-0.24098	1.13733
				C	1.59768	-0.47272	3.50653
C	-1.27402	-0.97414	5.80837	C	0.97039	-0.42458	2.23567
C	-1.88928	0.77694	-5.49160	C	-0.45116	-0.50513	2.22422
C	1.17782	0.88850	-3.39205	C	-1.14740	-0.65969	3.38472
C	0.31008	1.05290	-4.43529	N	-0.50969	-0.73388	4.58191
N	-0.92276	0.48164	-4.42705	C	0.85110	-0.63622	4.63232
C	-1.31012	-0.28991	-3.37703	H	2.60599	0.53795	-1.10033
C	-0.49047	-0.46585	-2.30210	H	-2.32216	-0.73023	5.62847
C	0.77909	0.15819	-2.25097	H	-0.89178	-0.33948	6.61216
C	1.59275	0.12180	-1.07829	H	-1.19582	-2.02649	6.10225
N	1.03762	-0.30326	0.02735	H	-1.35540	0.97389	-6.42344

H	-2.48547	1.64918	-5.20567	O	4.27426	2.27081	-2.94583
H	-2.54819	-0.08310	-5.62440	C	5.94834	0.25605	-1.80263
H	2.15133	1.36569	-3.43235	N	5.38176	-1.01149	-1.41943
H	0.55428	1.65527	-5.30308	C	5.75659	-1.75540	-0.24294
H	-2.29230	-0.74318	-3.44831	C	4.85616	-3.03163	-0.33032
H	-0.82108	-1.08510	-1.47728	N	3.97274	-2.74469	-1.44551
H	2.67407	-0.38256	3.57615	C	4.33260	-1.58479	-2.12027
H	-0.99382	-0.42526	1.29176	O	3.85222	-1.17361	-3.15529
H	-2.22947	-0.72653	3.41107	C	3.20793	-3.76566	-2.12140
H	1.29488	-0.69360	5.62047	N	1.95543	-4.09182	-1.47399
C	-5.12978	2.39657	-1.79942	C	1.89398	-4.98291	-0.32892
N	-4.07396	3.19861	-1.23983	C	0.38110	-5.34421	-0.24225
C	-4.16952	3.88385	0.02451	N	-0.15912	-4.81928	-1.46823
C	-2.84261	4.70378	0.08527	C	0.77396	-4.09834	-2.20618
N	-2.10506	4.24314	-1.07930	O	0.59285	-3.60483	-3.29799
C	-2.86559	3.40705	-1.88645	C	-1.44932	-5.17603	-1.99954
O	-2.55164	2.97163	-2.97696	N	-2.57561	-4.49942	-1.38940
C	-1.00314	4.99933	-1.63727	C	-3.15027	-4.84753	-0.11354
N	0.25927	4.78895	-0.96603	C	-4.49006	-4.04488	-0.09574
C	0.59432	5.46823	0.27777	N	-4.35910	-3.15262	-1.23931
C	2.13609	5.26253	0.38299	C	-3.28845	-3.52086	-2.05519
N	2.49642	4.76970	-0.92196	O	-3.05220	-3.08457	-3.16271
C	1.39582	4.50135	-1.72058	C	-5.49941	-2.47478	-1.83332
O	1.41516	4.12483	-2.87346	N	-5.70097	-1.11892	-1.37910
C	3.85479	4.65652	-1.40664	C	-6.20640	-0.76778	-0.07425
N	4.57327	3.47519	-0.98711	C	-6.09889	0.78943	-0.06247
C	5.18417	3.27891	0.30532	N	-5.25989	1.06971	-1.22060
C	5.95341	1.93164	0.13184	C	-5.10613	-0.04849	-2.02928
N	5.39167	1.39930	-1.10280	O	-4.58812	-0.07430	-3.12792
C	4.68537	2.36470	-1.80714	N	-5.52145	1.06461	1.22767

C	-5.27078	2.38630	1.75665	C	-5.47938	-2.49204	1.66391
N	-4.10635	3.05111	1.20959	N	-5.39360	-1.16637	1.06637
C	-3.05500	3.41976	2.05300	C	-5.07836	-0.07558	1.87553
O	-2.87623	3.02774	3.18413	O	-4.55370	-0.12119	2.96845
N	-2.28566	4.34586	1.36579	H	-4.90847	2.26315	-2.86459
C	-1.22550	5.08651	2.01715	H	-6.08434	2.93566	-1.68035
N	0.09293	4.86163	1.48118	H	-5.07531	4.51498	0.04820
C	1.07815	4.18904	2.19817	H	-3.00143	5.79510	0.02489
O	0.93301	3.63005	3.26181	H	-1.26017	6.07526	-1.61219
N	2.26322	4.32544	1.48266	H	-0.87390	4.67763	-2.67701
C	3.53478	4.01998	2.09967	H	0.29702	6.53101	0.22666
N	4.28749	2.99488	1.41251	H	2.68806	6.19331	0.60357
C	4.65134	1.83454	2.09004	H	3.80372	4.62601	-2.50106
O	4.22499	1.46360	3.16094	H	4.41818	5.54432	-1.08207
N	5.65093	1.22246	1.34627	H	5.84313	4.13012	0.54894
C	6.12574	-0.10349	1.67186	H	7.04376	2.06261	0.02559
N	5.39834	-1.18419	1.04030	H	7.03718	0.22189	-1.63801
C	4.61247	-2.04346	1.79698	H	5.73764	0.39743	-2.86937
O	4.35488	-1.93521	2.97666	H	6.83687	-1.98129	-0.26803
N	4.23879	-3.09281	0.97182	H	5.43023	-3.95465	-0.52743
C	3.51165	-4.22890	1.48202	H	2.96589	-3.38801	-3.12182
N	2.17429	-4.38931	0.95255	H	3.82209	-4.68167	-2.20774
C	1.04009	-4.23756	1.73565	H	2.54445	-5.86087	-0.49266
O	0.99739	-3.83665	2.87849	H	0.19381	-6.43109	-0.17358
N	-0.04601	-4.68058	0.97616	H	-1.44998	-4.90531	-3.06184
C	-1.29126	-5.01559	1.62523	H	-1.59560	-6.26277	-1.88937
N	-2.45046	-4.35067	1.06066	H	-3.29369	-5.93945	-0.04175
C	-3.27426	-3.58326	1.87171	H	-5.38208	-4.68295	-0.21385
O	-3.01092	-3.16177	2.97855	H	-6.41324	-3.05389	-1.62950
N	-4.48428	-3.42866	1.20608	H	-5.32572	-2.42469	-2.91443

H	-7.23952	-1.13362	0.04654
H	-7.07282	1.29674	-0.16520
H	-6.16542	3.00475	1.57907
H	-5.10224	2.27531	2.83424
H	-1.45539	6.16389	1.95765
H	-1.21159	4.76678	3.06577
H	3.32398	3.64592	3.10903
H	4.14085	4.94238	2.16307

H	6.00984	-0.23022	2.75466
H	7.19105	-0.16876	1.40112
H	3.41984	-4.08717	2.56523
H	4.08198	-5.14983	1.27247
H	-1.44829	-6.10962	1.58293
H	-1.21007	-4.69577	2.67070
H	-6.47885	-2.91015	1.46740
H	-5.33170	-2.37506	2.74375

$\mathbf{R_a}H^{2+}{\subset}CB8$: local minimum on the potential energy surface at the r2scan-3c level of theory.

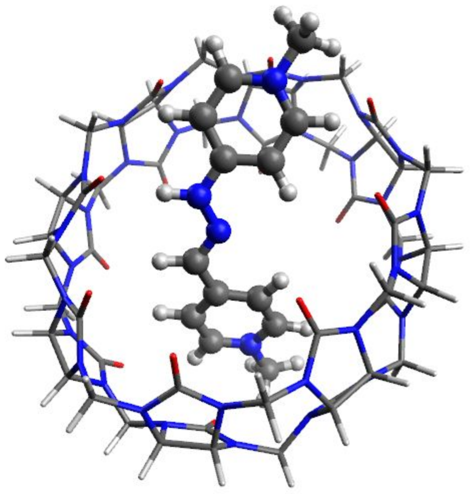

177

C	1.66381	-5.99996	-0.30539
H	2.17731	-6.95587	-0.50075
N	2.17754	-5.37913	0.88728
C	1.17238	-4.84642	1.67556
O	1.32617	-4.19903	2.69425
N	-0.04438	-5.20989	1.10150
C	-1.24490	-5.24046	1.91560
N	-2.40199	-4.63028	1.28769
C	-3.15791	-3.71876	2.04090
O	-2.75287	-3.05816	2.97065

N	-4.45132	-3.74672	1.54615
C	-5.52638	-2.97641	2.12506
N	-5.83159	-1.74154	1.42577
C	-5.89594	-0.55899	2.16930
O	-5.44200	-0.37941	3.27363
N	-6.62181	0.35607	1.41454
C	-6.79513	1.72273	1.84514
N	-5.84807	2.67336	1.29438
C	-4.70101	3.03914	1.97608
O	-4.33924	2.63798	3.05815
N	-4.03842	3.99656	1.19389
C	-3.15597	4.91552	1.88535

N	-1.88327	5.11259	1.23704
C	-0.68824	4.72180	1.79873
O	-0.55412	3.96595	2.75487
N	0.33140	5.34818	1.11106
C	1.62553	5.56614	1.72876
N	2.72387	4.95954	1.02496
C	3.59746	4.09891	1.67037
O	3.42107	3.58133	2.75923
N	4.71293	3.96921	0.87089
C	5.85012	3.17264	1.26136
N	5.96798	1.90489	0.56598
C	5.96087	0.71243	1.27744
O	5.55190	0.55091	2.41404
N	6.54353	-0.25170	0.47614
C	6.71516	-1.61669	0.90533
N	5.71215	-2.54650	0.41473
C	4.92590	-3.26226	1.30153
O	4.78188	-3.02886	2.48995
N	4.36067	-4.31398	0.60806
C	4.78478	-4.37360	-0.77299
H	5.20312	-5.36864	-1.00282
N	3.77992	-4.00121	-1.74975
C	2.84832	-4.95943	-2.30091
N	1.65709	-5.15625	-1.49537
C	0.39836	-5.10622	-2.08164
O	0.10820	-4.59267	-3.14468
N	-0.47726	-5.77151	-1.23592
C	-1.82692	-6.11412	-1.62003
N	-2.86412	-5.30843	-1.01834
C	-3.65664	-4.44433	-1.75045
O	-3.53852	-4.19190	-2.93483
N	-4.62129	-3.93213	-0.89233
C	-5.83586	-3.35477	-1.44357
N	-6.12965	-2.01665	-0.99523
C	-5.77228	-0.89437	-1.71758
O	-5.20639	-0.88689	-2.79644
N	-6.20899	0.21833	-1.00986
C	-6.38454	1.48901	-1.68953
N	-5.57838	2.55953	-1.14440
C	-4.65785	3.23737	-1.93040
O	-4.27506	2.91003	-3.03783
N	-4.25405	4.35714	-1.22164
C	-3.34346	5.34357	-1.76133
N	-2.00738	5.32307	-1.18680
C	-0.88755	5.29275	-2.03015
O	-0.87964	4.94515	-3.18765
N	0.19537	5.75612	-1.29437
C	1.48201	6.03790	-1.90135
N	2.57883	5.23748	-1.40064
C	3.30412	4.40513	-2.25373
O	2.99656	4.09775	-3.38025
N	4.46074	4.02289	-1.57297
C	5.57685	3.44197	-2.29960
N	5.93912	2.10876	-1.88436
C	5.53847	0.98882	-2.60081
O	4.91925	0.98340	-3.63779
N	6.02787	-0.12910	-1.92004
C	6.14305	-1.39306	-2.62086
N	5.40229	-2.47911	-2.02237
C	4.19824	-2.92628	-2.54276

O	3.62889	-2.49873	-3.51946	H	-1.03237	-4.68119	2.83371
C	5.83579	-3.22274	-0.87294	H	-1.48212	-6.28969	2.17383
H	6.86938	-3.58353	-1.00866	H	-5.22482	-2.69546	3.14099
C	6.91057	0.24462	-0.83100	H	-6.42328	-3.61655	2.16922
H	7.95450	-0.02608	-1.06219	H	-7.81051	2.05483	1.58757
C	6.68546	1.78338	-0.69997	H	-6.66601	1.73092	2.93345
H	7.62418	2.36087	-0.65825	H	-2.96789	4.49393	2.87920
C	4.64074	4.75927	-0.34063	H	-3.64769	5.89885	1.99039
H	5.51989	5.42246	-0.41034	H	1.59921	5.12868	2.73242
C	3.29252	5.53306	-0.18271	H	1.79090	6.65522	1.80866
H	3.42660	6.62238	-0.06255	H	5.74068	2.95415	2.32939
C	-0.16547	6.18809	0.03037	H	6.76796	3.76027	1.09562
H	0.13256	7.23666	0.19914	H	6.65918	-1.62773	1.99921
C	-1.70941	5.96204	0.07919	H	7.71164	-1.95990	0.58412
H	-2.28720	6.89427	0.19664	H	3.35759	-5.93160	-2.43379
C	-4.87258	4.43513	0.07933	H	2.52300	-4.58051	-3.27688
H	-5.27240	5.44681	0.26176	H	-2.00189	-7.17015	-1.36278
C	-5.97525	3.33544	0.02420	H	-1.90736	-5.98195	-2.70467
H	-6.99399	3.73658	-0.10491	H	-6.69497	-3.99755	-1.19283
C	-7.02633	-0.16870	0.14123	H	-5.70905	-3.32263	-2.53117
H	-8.08852	0.05164	-0.05515	H	-7.45009	1.77440	-1.65041
C	-6.72469	-1.69072	0.27635	H	-6.08200	1.35471	-2.73458
H	-7.62607	-2.30213	0.44857	H	-3.78844	6.34231	-1.61806
C	-4.59695	-4.60353	0.40445	H	-3.23525	5.13972	-2.83286
H	-5.47911	-5.25607	0.51473	H	1.71679	7.10530	-1.75138
C	-3.24787	-5.38210	0.36863	H	1.38568	5.82768	-2.97316
H	-3.34074	-6.43259	0.69274	H	5.28041	3.38217	-3.35364
C	0.14839	-6.15175	0.00015	H	6.45664	4.09699	-2.19491
H	-0.14551	-7.17244	0.29619	H	7.20423	-1.68513	-2.68493
C	3.57548	-5.33762	1.25712	H	5.73820	-1.24114	-3.62872

H	3.63065	-5.14839	2.33450	C	1.33776	-0.87331	3.06898
H	4.01328	-6.32290	1.03444	C	2.47885	-1.28345	3.70475
C	4.48835	-0.87870	5.04234	H	4.27190	-1.19893	6.06641
N	3.24644	-0.40929	4.39921	H	5.21181	-0.06327	5.04384
C	2.86933	0.88504	4.52044	H	4.88722	-1.70736	4.45730
C	1.72430	1.35128	3.93351	H	3.53775	1.53673	5.07040
C	0.95904	0.47712	3.14761	H	1.47954	2.40454	4.00590
N	-0.10597	0.97447	2.44044	H	-2.57734	1.88127	-1.56773
N	-0.55563	0.27190	1.40298	H	-3.01546	0.75440	-3.71600
C	-1.39524	0.82336	0.59468	H	-2.15733	-0.98171	-5.19406
H	-1.78787	1.83889	0.73880	H	-3.50699	-1.92010	-4.46686
C	-1.73107	0.12154	-0.62860	H	-1.85624	-2.59700	-4.48276
C	-2.32574	0.83367	-1.67597	H	-1.34607	-2.79812	-2.35062
C	-2.55155	0.22869	-2.88937	H	-0.93961	-1.82506	-0.08208
N	-2.18142	-1.05292	-3.09435	H	0.75372	-1.59940	2.51953
C	-2.44254	-1.68155	-4.40602	H	2.82255	-2.31030	3.65264
C	-1.62131	-1.77927	-2.09639	H	-0.36939	1.96041	2.57365
C	-1.39967	-1.22593	-0.85967				

R$_a$$^+$⊂CB8: local minimum on the potential energy surface at the r2scan-3c level of theory.

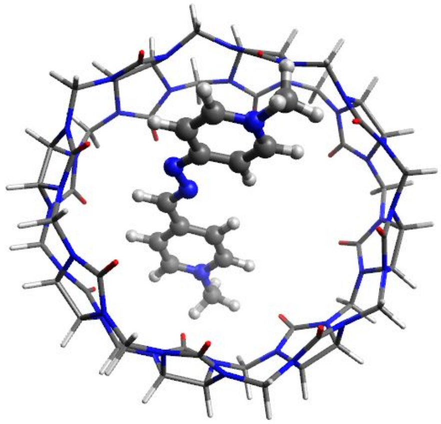

176

C	1.83843	-6.62409	-0.04790
H	2.37834	-7.57959	0.07080

N	2.09002	-5.75784	1.09560
C	0.96082	-5.64513	1.91219
O	0.92125	-5.14269	3.01304
N	-0.09103	-6.25445	1.24502
C	-1.40431	-6.38435	1.82717
N	-2.40934	-5.49685	1.27097
C	-3.16585	-4.69667	2.13156
O	-2.90630	-4.43736	3.28327
N	-4.29237	-4.28857	1.42763
C	-5.36966	-3.56551	2.06751
N	-5.54440	-2.20460	1.61702
C	-5.22385	-1.11606	2.42103
O	-4.72819	-1.16201	3.52252
N	-5.62318	0.03175	1.73459
C	-5.78854	1.27541	2.46438
N	-5.11972	2.40695	1.87542
C	-3.99784	2.98705	2.45716
O	-3.41274	2.58796	3.43706
N	-3.70779	4.13933	1.72626
C	-2.85274	5.15666	2.29950
N	-1.70598	5.49045	1.48240
C	-0.43113	5.46978	2.04492
O	-0.11725	4.97684	3.10661
N	0.40290	6.18226	1.19625
C	1.79499	6.42014	1.50427
N	2.72622	5.50456	0.88596
C	3.52943	4.65656	1.62999
O	3.54495	4.55332	2.83951
N	4.34854	3.97511	0.73605
C	5.51270	3.25540	1.20284
N	5.61701	1.89856	0.71452
C	5.47579	0.79362	1.53569
O	5.14774	0.80220	2.70613
N	5.82310	-0.32856	0.78608
C	6.11926	-1.58079	1.44808
N	5.37822	-2.71204	0.93154
C	4.52879	-3.43908	1.75206
O	4.12865	-3.10242	2.85158
N	4.25390	-4.63396	1.11504
C	4.93972	-4.77389	-0.14739
H	5.52989	-5.70706	-0.15703
N	4.11599	-4.68719	-1.33819
C	3.41659	-5.83363	-1.88761
N	2.09907	-6.05820	-1.34440
C	0.94642	-5.67732	-2.01356
O	0.88342	-5.18245	-3.11686
N	-0.13617	-6.01498	-1.19694
C	-1.44066	-6.19737	-1.80292
N	-2.50279	-5.44333	-1.18152
C	-3.13695	-4.39131	-1.81381
O	-2.84373	-3.92130	-2.89892
N	-4.19319	-3.99399	-1.00815
C	-5.25202	-3.15872	-1.53009
N	-5.42755	-1.91147	-0.81946
C	-5.40560	-0.70328	-1.50723
O	-4.99298	-0.52862	-2.63865
N	-5.97738	0.25163	-0.68703
C	-6.24777	1.59782	-1.13373
N	-5.38718	2.61098	-0.55744
C	-4.75608	3.53250	-1.39023

O	-4.61415	3.43538	-2.59233	H	3.43307	6.58843	-0.76504
N	-4.35741	4.59697	-0.59889	C	-0.28647	6.68230	0.03920
C	-3.73922	5.78465	-1.14547	H	-0.03804	7.74462	-0.12904
N	-2.33324	5.94536	-0.85472	C	-1.78704	6.44131	0.38338
C	-1.34818	5.60880	-1.76967	H	-2.31960	7.35612	0.69696
O	-1.52953	5.15366	-2.88205	C	-4.75923	4.45691	0.77894
N	-0.12873	5.92158	-1.19276	H	-5.31329	5.35527	1.10520
C	1.08100	5.93748	-1.98480	C	-5.63233	3.16416	0.76828
N	2.13552	5.09881	-1.46649	H	-6.71063	3.36417	0.90192
C	2.57114	3.98435	-2.17449	C	-6.43011	-0.29003	0.57253
O	2.01798	3.48062	-3.12665	H	-7.48595	-0.01507	0.74507
N	3.78508	3.58881	-1.61824	C	-6.21375	-1.82573	0.40359
C	4.68279	2.73619	-2.35785	H	-7.15594	-2.39046	0.28788
N	5.06722	1.52858	-1.65277	C	-4.38778	-4.88708	0.12315
C	5.09681	0.32621	-2.36230	H	-5.34442	-5.42848	0.01452
O	4.53892	0.09078	-3.40923	C	-3.13741	-5.82115	0.05939
N	5.92778	-0.54105	-1.66256	H	-3.39551	-6.89500	0.04159
C	6.23942	-1.86533	-2.14515	C	0.28714	-6.78646	-0.03706
N	5.50735	-2.93950	-1.51178	H	-0.04275	-7.83591	-0.13265
C	4.55246	-3.67673	-2.19863	C	3.40743	-5.64747	1.69656
O	4.19265	-3.50200	-3.33928	H	-1.31588	-6.13870	2.89209
C	5.82159	-3.48517	-0.21825	H	-1.73565	-7.43092	1.71176
H	6.90341	-3.69150	-0.13762	H	-5.13547	-3.52380	3.13805
C	6.46384	0.04757	-0.46938	H	-6.31211	-4.11572	1.91023
H	7.55319	-0.12334	-0.40763	H	-6.86466	1.50785	2.55451
C	6.07821	1.54934	-0.60602	H	-5.35908	1.11912	3.46116
H	6.92716	2.19385	-0.89665	H	-2.46972	4.76598	3.24997
C	4.25386	4.50844	-0.60774	H	-3.44945	6.06904	2.48742
H	5.22229	4.94694	-0.91161	H	1.92127	6.31086	2.58762
C	3.10748	5.56450	-0.50590	H	2.03991	7.45217	1.20481

H	5.44666	3.21366	2.29583	N	0.59799	2.16973	0.87453
H	6.42750	3.79924	0.90727	N	0.16783	1.39894	-0.10803
H	5.84883	-1.46181	2.50365	C	-0.48822	1.99852	-1.07041
H	7.20094	-1.79447	1.36728	H	-0.64851	3.08123	-1.06064
H	4.01953	-6.74275	-1.72620	C	-1.05663	1.21037	-2.11047
H	3.29748	-5.65052	-2.96220	C	-1.88906	1.80090	-3.08904
H	-1.70972	-7.26827	-1.77773	C	-2.51951	1.02287	-4.01807
H	-1.36335	-5.86033	-2.84307	N	-2.33879	-0.32255	-4.05344
H	-6.20068	-3.72611	-1.50978	C	-3.03617	-1.13321	-5.05887
H	-4.99720	-2.91173	-2.56677	C	-1.50860	-0.92073	-3.15627
H	-7.30181	1.83449	-0.90404	C	-0.87179	-0.19243	-2.19496
H	-6.09239	1.62591	-2.21820	C	1.49077	0.05341	1.81231
H	-4.27473	6.66917	-0.76725	C	2.11954	-0.55379	2.85880
H	-3.84152	5.72456	-2.23495	H	3.24890	-1.58413	4.87173
H	1.45771	6.97307	-2.06335	H	2.81606	-0.26848	5.99717
H	0.81289	5.56504	-2.98076	H	4.30140	-0.15524	4.99925
H	4.16839	2.42419	-3.27399	H	2.60197	1.98987	4.92050
H	5.59055	3.31153	-2.62391	H	1.41022	3.20038	3.11378
H	7.31928	-2.03970	-2.01441	H	-2.06794	2.87070	-3.09426
H	5.98244	-1.89432	-3.21076	H	-3.20595	1.43578	-4.74859
H	3.26321	-5.38434	2.75100	H	-2.48350	-1.11709	-6.00474
H	3.92258	-6.62043	1.62241	H	-4.04030	-0.73124	-5.20453
C	3.26502	-0.50521	5.02826	H	-3.11312	-2.15793	-4.69216
N	2.50279	0.14671	3.95845	H	-1.41004	-1.99731	-3.24292
C	2.25818	1.48460	4.02440	H	-0.22796	-0.69519	-1.48457
C	1.61986	2.14251	3.01743	H	1.19415	-0.53683	0.95578
C	1.21692	1.44989	1.84893	H	2.35331	-1.61262	2.86947

mA_2R$_a^+$⊂CB8: local minimum on the potential energy surface at the r2scan-3c level of theory.

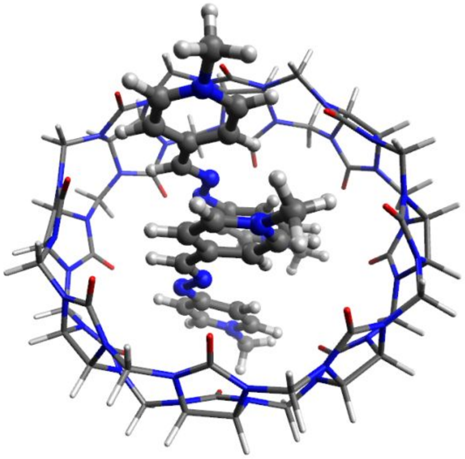

208

C	-6.82096	0.97780	-0.12501
H	-7.80709	1.43210	-0.31467
N	-5.92223	1.19663	-1.25443
C	-5.73366	0.03325	-1.99049
O	-5.15685	-0.05957	-3.05691
N	-6.35366	-0.99695	-1.30561
C	-6.35752	-2.35677	-1.80087
N	-5.29899	-3.19826	-1.29002
C	-4.32541	-3.73189	-2.12633
O	-4.14287	-3.43390	-3.28967
N	-3.62421	-4.67427	-1.39528
C	-2.72746	-5.60956	-2.04871
N	-1.37777	-5.59185	-1.55006
C	-0.31270	-5.11681	-2.30607
O	-0.39843	-4.55737	-3.37947
N	0.85489	-5.44149	-1.62401
C	2.12669	-5.45669	-2.32553
N	3.16594	-4.68761	-1.68568
C	3.78397	-3.63507	-2.34726
O	3.43115	-3.14080	-3.39901
N	4.91140	-3.29361	-1.62210
C	5.85914	-2.31589	-2.09927
N	5.89547	-1.08727	-1.32749
C	5.69972	0.13641	-1.96342
O	5.21439	0.30642	-3.06266
N	6.21721	1.11871	-1.13490
C	6.22991	2.51609	-1.49863
N	5.20149	3.32309	-0.87645
C	4.25244	3.98359	-1.64387
O	4.06903	3.85072	-2.83457
N	3.59302	4.87692	-0.80724
C	2.73082	5.90131	-1.34796
N	1.35884	5.83161	-0.89501
C	0.31753	5.48103	-1.73545
O	0.40723	5.13349	-2.89404
N	-0.86684	5.64268	-1.01358
C	-2.13068	5.71599	-1.70897
N	-3.15472	4.84319	-1.17065
C	-3.68538	3.80081	-1.91138
O	-3.24761	3.35843	-2.95566

N	-4.83349	3.36882	-1.26036	N	4.99843	3.00605	1.55036
C	-5.17701	4.19829	-0.12640	C	3.89972	3.43512	2.26969
H	-6.14698	4.69792	-0.29324	O	3.48645	2.95162	3.30712
N	-5.16845	3.53407	1.15814	N	3.37729	4.53701	1.61509
C	-6.26717	2.73655	1.65655	C	2.37470	5.36936	2.23235
N	-6.31557	1.37625	1.16381	N	1.11277	5.41225	1.51660
C	-5.75804	0.31812	1.85910	C	-0.07595	5.14280	2.18491
O	-5.19875	0.38422	2.93814	O	-0.19774	4.57699	3.25243
N	-5.98408	-0.83514	1.11883	N	-1.10790	5.67813	1.42550
C	-5.90513	-2.13385	1.76481	C	-2.45402	5.77984	1.92635
N	-4.99389	-3.05680	1.13292	N	-3.40688	4.90401	1.27077
C	-3.86603	-3.53304	1.78689	C	-4.17050	4.00882	1.99841
O	-3.41699	-3.10971	2.83575	O	-4.01362	3.70320	3.16479
N	-3.37038	-4.59174	1.04528	C	-3.97579	5.19230	-0.03196
C	-2.39374	-5.51166	1.59205	H	-4.27902	6.25180	-0.09101
N	-1.13173	-5.52342	0.88801	C	-0.64017	6.34555	0.23846
C	0.05963	-5.44674	1.61090	H	-1.04978	7.36990	0.18657
O	0.17558	-5.08792	2.76465	C	0.91015	6.30842	0.38771
N	1.07068	-5.90033	0.78401	H	1.35276	7.29653	0.60426
C	2.42928	-6.07429	1.24416	C	4.19973	4.96388	0.50030
N	3.36924	-5.10808	0.72734	H	4.58012	5.98497	0.68070
C	4.03414	-4.22891	1.56557	C	5.32948	3.88397	0.44889
O	3.82098	-4.06396	2.75005	H	6.34125	4.30309	0.59111
N	5.01264	-3.59770	0.81157	C	6.73387	0.59285	0.10026
C	6.07041	-2.84705	1.45398	H	7.74183	0.99371	0.30122
N	6.13445	-1.45238	1.08953	C	6.71178	-0.94841	-0.13095
C	5.58940	-0.45513	1.87765	H	7.71238	-1.38427	-0.29142
O	4.99901	-0.61208	2.92958	C	5.15269	-4.17791	-0.50983
N	5.88021	0.76021	1.26959	H	6.14209	-4.65831	-0.60890
C	5.87966	1.98042	2.05237	C	3.96443	-5.19121	-0.58753

H	4.28768	-6.22701	-0.79360
C	0.59315	-6.33303	-0.50350
H	0.96767	-7.34718	-0.72770
C	-0.95497	-6.26518	-0.35268
H	-1.43089	-7.25917	-0.27877
C	-4.20704	-4.91671	-0.09137
H	-4.55984	-5.95988	-0.01700
C	-5.35946	-3.86568	-0.00981
H	-6.35517	-4.31716	0.14221
C	-6.85695	-0.57638	-0.02436
H	-7.86473	-0.98574	0.15320
C	-5.77853	2.48781	-1.90227
H	-6.23219	-2.31072	-2.88846
H	-7.33368	-2.80492	-1.55991
H	-2.69519	-5.33360	-3.10866
H	-3.13351	-6.62994	-1.94335
H	2.46350	-6.50362	-2.43638
H	1.95818	-5.01972	-3.31664
H	5.56460	-2.04872	-3.12072
H	6.86614	-2.76805	-2.10976
H	6.07147	2.57456	-2.58149
H	7.21954	2.93032	-1.24555
H	2.72321	5.77730	-2.43707
H	3.13489	6.89468	-1.08928
H	-1.94632	5.41454	-2.74642
H	-2.51106	6.75308	-1.69028
H	-7.22008	3.22548	1.40280
H	-6.15774	2.68759	2.74564
H	-6.91213	-2.58535	1.79375
H	-5.54897	-1.96532	2.78746
H	-2.83191	-6.52628	1.59331
H	-2.18549	-5.20368	2.62295
H	2.76439	-7.08845	0.97075
H	2.42281	-5.96797	2.33498
H	7.04270	-3.30918	1.21827
H	5.89062	-2.89877	2.53367
H	6.90769	2.38029	2.09803
H	5.53879	1.72356	3.06183
H	2.17239	4.95716	3.22755
H	2.76774	6.39722	2.33008
H	-2.79395	6.82436	1.82340
H	-2.43899	5.49999	2.98560
H	-5.41703	2.30067	-2.91992
H	-6.76388	2.98400	-1.94526
C	-0.00924	2.46932	-6.58303
N	0.41275	2.13687	-5.21863
C	1.72689	1.86315	-4.96139
C	2.17920	1.67982	-3.69109
C	1.28887	1.78590	-2.59159
N	1.81410	1.64387	-1.34665
N	0.88065	1.63613	-0.41320
C	1.29859	1.51198	0.82363
H	2.36407	1.42752	1.06371
C	0.33159	1.45797	1.86681
C	0.72527	1.42120	3.22447
C	-0.21393	1.42300	4.21558
N	-1.54251	1.41363	3.93345
C	-2.53454	1.51522	5.01266
C	-1.95830	1.39673	2.63810
C	-1.06215	1.42222	1.61030

C	-0.07749	2.03976	-2.89657	H	2.64062	-1.73978	1.41925
C	-0.47195	2.22300	-4.18625	C	1.07431	-1.95821	2.91634
H	0.53264	1.84640	-7.29869	C	1.96523	-1.92729	4.01745
H	0.19338	3.52424	-6.79816	C	1.49158	-2.06395	5.28932
H	-1.07997	2.28366	-6.68759	N	0.15916	-2.21614	5.53862
H	2.38047	1.80748	-5.82550	C	-0.30050	-2.48922	6.90506
H	3.22552	1.45789	-3.51496	C	-0.72661	-2.23059	4.50033
H	1.77583	1.44095	3.49155	C	-0.30445	-2.09552	3.21349
H	0.05146	1.44432	5.26730	C	-1.09385	-1.42760	-1.39049
H	-3.47211	1.06697	4.67982	C	-1.92500	-1.29232	-2.46450
H	-2.70966	2.56853	5.25109	H	-1.95904	-0.64198	-5.66502
H	-2.16159	0.98917	5.89476	H	-3.33486	-0.86696	-4.54676
H	-3.03210	1.35882	2.49663	H	-2.52878	-2.28847	-5.24880
H	-1.41669	1.40571	0.58658	H	0.20873	-1.40821	-4.99073
H	-0.81190	2.13028	-2.10766	H	1.83368	-1.60119	-3.12353
H	-1.49709	2.47046	-4.44054	H	3.03170	-1.81293	3.85446
C	-2.37662	-1.27165	-4.87441	H	2.14236	-2.06756	6.15728
N	-1.44781	-1.29828	-3.73726	H	-0.21200	-3.55784	7.12923
C	-0.10874	-1.40710	-3.95333	H	0.30293	-1.92146	7.61694
C	0.77266	-1.51947	-2.92126	H	-1.34543	-2.18927	7.00432
C	0.30827	-1.57704	-1.58526	H	-1.77220	-2.37684	4.74902
N	1.24284	-1.73366	-0.60927	H	-1.03792	-2.12896	2.41882
N	0.71012	-1.84012	0.58969	H	-1.50447	-1.41235	-0.38854
C	1.56481	-1.84896	1.58994	H	-2.99871	-1.16762	-2.37497

mB_2R$_a$$^+$ $\subset$CB8: local minimum on the potential energy surface at the r2scan-3c level of theory.

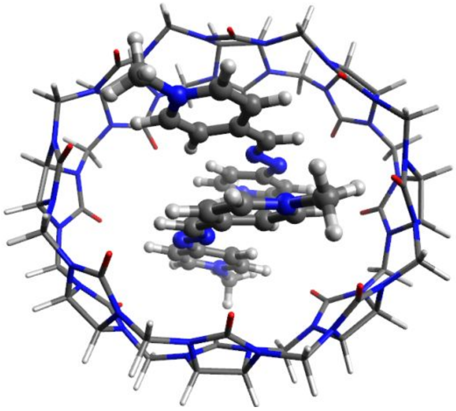

208

C	6.42750	2.44133	-0.03201
H	7.27774	3.12397	0.13292
N	5.56468	2.39559	1.14041
C	5.71058	1.19611	1.83557
O	5.27158	0.95010	2.93955
N	6.49413	0.36171	1.05791
C	6.83274	-0.98313	1.47248
N	5.99311	-2.02353	0.92409
C	5.17680	-2.79749	1.73908
O	4.96658	-2.62028	2.91958
N	4.69511	-3.84401	0.96478
C	4.03579	-4.97553	1.57692
N	2.68585	-5.20480	1.11627
C	1.57882	-4.98779	1.91992
O	1.58220	-4.52852	3.04294
N	0.46257	-5.43554	1.21243
C	-0.77510	-5.69563	1.91257
N	-1.94494	-5.13028	1.26847
C	-2.78449	-4.27015	1.96895
O	-2.50869	-3.66866	2.98823
N	-4.00462	-4.25192	1.31530
C	-5.16560	-3.58896	1.86233
N	-5.56783	-2.39524	1.14422
C	-5.71222	-1.19476	1.83813
O	-5.27336	-0.94812	2.94199
N	-6.49464	-0.36018	1.05941
C	-6.82949	0.98630	1.47152
N	-5.98826	2.02368	0.91974
C	-5.17310	2.80046	1.73334
O	-4.96117	2.62490	2.91371
N	-4.69532	3.84783	0.95777
C	-4.03698	4.98089	1.56782
N	-2.68731	5.21062	1.10692
C	-1.58019	4.99368	1.91048
O	-1.58326	4.53442	3.03339
N	-0.46438	5.44244	1.20315
C	0.77341	5.70211	1.90274
N	1.94246	5.13476	1.25916
C	2.77877	4.27133	1.95939
O	2.49967	3.66897	2.97720
N	3.99972	4.25100	1.30700
C	4.05294	5.15211	0.18390

H	4.88271	5.86994	0.30517
N	4.12052	4.52902	-1.12632
C	5.36667	4.06219	-1.70346
N	5.77501	2.74297	-1.28165
C	5.50727	1.60834	-2.02621
O	4.91865	1.57644	-3.09303
N	6.06165	0.52757	-1.35947
C	6.32512	-0.71296	-2.06886
N	5.67643	-1.86962	-1.50356
C	4.68609	-2.55429	-2.18492
O	4.17635	-2.21491	-3.23672
N	4.40789	-3.70638	-1.47133
C	3.60875	-4.76825	-2.03716
N	2.37683	-5.03454	-1.31929
C	1.17640	-5.12206	-2.01913
O	0.96404	-4.72155	-3.14535
N	0.27032	-5.78932	-1.20692
C	-1.00204	-6.25952	-1.69892
N	-2.15275	-5.58350	-1.13673
C	-3.05654	-4.89941	-1.92764
O	-2.94956	-4.67510	-3.11844
N	-4.12413	-4.53076	-1.11820
C	-5.37048	-4.06551	-1.69599
N	-5.77895	-2.74532	-1.27742
C	-5.50696	-1.61220	-2.02250
O	-4.91682	-1.58321	-3.08854
N	-6.05934	-0.52911	-1.35748
C	-6.32247	0.70954	-2.07046
N	-5.67482	1.86799	-1.50777
C	-4.68394	2.55143	-2.18943
O	-4.17311	2.20975	-3.23999
N	-4.40640	3.70495	-1.47782
C	-3.60783	4.76637	-2.04571
N	-2.37633	5.03544	-1.32801
C	-1.17582	5.12446	-2.02783
O	-0.96291	4.72407	-3.15401
N	-0.27096	5.79399	-1.21619
C	1.00160	6.26447	-1.70778
N	2.15251	5.58832	-1.14588
C	3.05473	4.90208	-1.93649
O	2.94796	4.67883	-3.12755
C	2.64441	5.82567	0.19300
H	2.68047	6.90925	0.40077
C	-0.85157	6.21358	0.03235
H	-0.66659	7.28967	0.19934
C	-2.36002	5.86614	-0.13103
H	-3.00069	6.75385	-0.27551
C	-5.30196	3.89346	-0.35265
H	-5.87473	4.82889	-0.47948
C	-6.19768	2.61166	-0.38139
H	-7.26887	2.83008	-0.53615
C	-6.84953	-0.94968	-0.20329
H	-7.92489	-0.81478	-0.40634
C	-6.43073	-2.44078	-0.02828
H	-7.28240	-3.12136	0.13767
C	-4.05524	-5.15314	0.19211
H	-4.88310	-5.87316	0.31337
C	-2.64481	-5.82274	0.20163
H	-2.67794	-6.90648	0.40915
C	0.84975	-6.20816	0.04230

H	0.66357	-7.28386	0.21047	H	-1.08404	-7.33967	-1.49216
C	2.35871	-5.86254	-0.12072	H	-1.02594	-6.08820	-2.78084
H	2.99862	-6.75120	-0.26275	H	-6.17828	-4.77042	-1.44289
C	5.30248	-3.89325	-0.34544	H	-5.23062	-4.04229	-2.78247
H	5.87431	-4.82962	-0.46967	H	-7.41224	0.88205	-2.09728
C	6.19979	-2.61265	-0.37685	H	-5.94379	0.58622	-3.09151
H	7.27048	-2.83258	-0.53289	H	-3.33201	4.46575	-3.06289
C	6.84949	0.95078	-0.20487	H	-4.21557	5.68839	-2.08758
H	7.92539	0.81794	-0.40646	H	1.08342	7.34450	-1.50051
C	5.15983	3.58923	1.85709	H	1.02574	6.09368	-2.78978
H	6.72407	-1.02439	2.56232	H	4.91115	3.28123	2.87924
H	7.88166	-1.17442	1.19663	H	5.99735	4.30756	1.87937
H	3.98680	-4.77503	2.65337	C	-0.48437	1.85948	6.34922
H	4.62888	-5.88767	1.39473	N	-0.84194	1.54492	4.96360
H	-0.92130	-6.78663	2.02019	C	-2.08770	1.07178	4.66583
H	-0.68652	-5.24148	2.90596	C	-2.48815	0.88792	3.37763
H	-4.91895	-3.28089	2.88495	C	-1.62108	1.21441	2.30487
H	-6.00369	-4.30663	1.88276	N	-2.10236	1.06708	1.04187
H	-6.71915	1.02934	2.56112	N	-1.18988	1.35454	0.12994
H	-7.87839	1.17943	1.19676	C	-1.57729	1.29022	-1.12024
H	-3.98780	4.78228	2.64461	H	-2.60541	1.02718	-1.38983
H	-4.63093	5.89214	1.38401	C	-0.62347	1.55367	-2.14541
H	0.68435	5.24932	2.89673	C	-1.00706	1.59383	-3.50592
H	0.92093	6.79306	2.00898	C	-0.08172	1.86081	-4.47462
H	6.17474	4.76768	-1.45260	N	1.22785	2.05056	-4.16839
H	5.22667	4.03650	-2.78985	C	2.18998	2.40850	-5.22195
H	7.41486	-0.88586	-2.09409	C	1.63751	1.98417	-2.87467
H	5.94742	-0.59214	-3.09058	C	0.74938	1.75163	-1.86463
H	4.21555	-5.69098	-2.07661	C	-0.32079	1.67518	2.65103
H	3.33341	-4.46954	-3.05504	C	0.02701	1.83732	3.95628

H	-0.99022	1.16722	7.02632	C	0.62385	-1.55100	-2.17328
H	-0.78139	2.88440	6.59734	C	1.01025	-1.59144	-3.53285
H	0.59620	1.76027	6.47559	C	0.08653	-1.85673	-4.50382
H	-2.73655	0.86530	5.51046	N	-1.22391	-2.04360	-4.20016
H	-3.48322	0.51015	3.17531	C	-2.18753	-2.40328	-5.25182
H	-2.04808	1.46815	-3.78328	C	-1.63628	-1.97807	-2.90697
H	-0.34287	1.94657	-5.52414	C	-0.74983	-1.74819	-1.89507
H	3.19983	2.20847	-4.86194	C	0.31696	-1.67090	2.62421
H	2.09835	3.47408	-5.45134	C	-0.02743	-1.84286	3.92918
H	1.98873	1.81161	-6.11472	H	1.00376	-1.20833	7.00282
H	2.70169	2.10939	-2.71454	H	0.78676	-2.91922	6.55307
H	1.10611	1.68433	-0.84386	H	-0.58750	-1.78891	6.45231
H	0.40102	1.92259	1.88391	H	2.74086	-0.88484	5.48331
H	0.99973	2.22065	4.24482	H	3.48096	-0.51071	3.15045
C	0.49214	-1.89019	6.31955	H	2.05188	-1.46616	-3.80825
N	0.84459	-1.55991	4.93631	H	0.34999	-1.94236	-5.55272
C	2.08921	-1.08378	4.63904	H	-1.94247	-1.86167	-6.16824
C	2.48608	-0.88965	3.35127	H	-2.14689	-3.48200	-5.42862
C	1.61742	-1.21057	2.27818	H	-3.19133	-2.13462	-4.91966
N	2.09833	-1.06108	1.01533	H	-2.70129	-2.10186	-2.75079
N	1.18679	-1.35062	0.10304	H	-1.10818	-1.68178	-0.87479
C	1.57562	-1.28652	-1.14666	H	-0.40689	-1.91147	1.85678
H	2.60365	-1.02228	-1.41496	H	-0.99941	-2.22858	4.21678

mC_2R$_a$ $^+$⊂CB8: local minimum on the potential energy surface at the r2scan-3c level of theory.

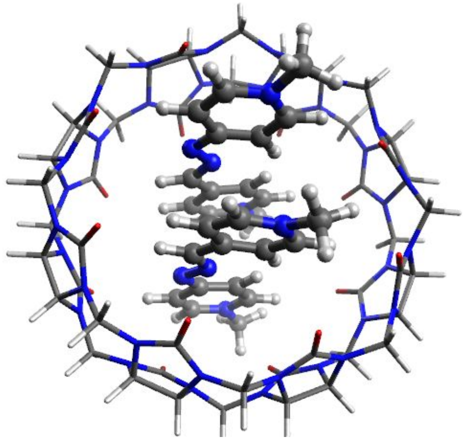

208

C	-3.24082	5.81739	0.00175
H	-4.01578	6.59100	0.13410
N	-3.23402	4.90763	1.13392
C	-2.06912	5.00533	1.88350
O	-1.84140	4.44926	2.94046
N	-1.22447	5.88364	1.22241
C	-0.00197	6.37316	1.81198
N	1.21161	5.84366	1.22016
C	2.16625	5.22367	2.01446
O	1.99890	4.81247	3.14850
N	3.34548	5.18330	1.29713
C	4.58219	4.69058	1.86931
N	5.01958	3.41116	1.36356
C	5.03788	2.27146	2.15760
O	4.57550	2.17721	3.27781
N	5.70392	1.28222	1.44896
C	6.20557	0.10133	2.13131
N	5.74681	-1.14410	1.57519
C	4.90082	-1.98925	2.27605
O	4.37257	-1.74995	3.34318
N	4.80611	-3.16906	1.55390
C	4.25295	-4.36311	2.15091
N	3.10173	-4.89176	1.45254
C	1.88664	-5.04746	2.10585
O	1.57344	-4.54548	3.16850
N	1.11357	-5.90336	1.34509
C	-0.17326	-6.38183	1.79207
N	-1.30408	-5.80867	1.09077
C	-2.31905	-5.18062	1.80775
O	-2.27000	-4.84356	2.97197
N	-3.41305	-5.07218	0.96536
C	-4.69338	-4.59513	1.43361
N	-5.10862	-3.32384	0.88088
C	-5.25198	-2.19319	1.66878
O	-4.95616	-2.08427	2.83941
N	-5.85184	-1.21237	0.87882
C	-6.45980	-0.05322	1.49288
N	-5.95099	1.21213	1.00908
C	-5.19088	2.04952	1.80422
O	-4.81019	1.82952	2.93579

N	-4.97208	3.21827	1.07966	N	-1.10794	-5.88688	-1.35582
C	-5.77516	3.27226	-0.12615	C	-1.87722	-5.02830	-2.11718
H	-6.55561	4.04806	-0.03485	O	-1.56314	-4.53352	-3.18305
N	-5.04684	3.43855	-1.36114	N	-3.08864	-4.86015	-1.46054
C	-4.60480	4.71704	-1.86585	C	-4.23497	-4.31767	-2.15558
N	-3.36241	5.20156	-1.29907	N	-4.78403	-3.12703	-1.54608
C	-2.18105	5.21737	-2.01457	C	-4.89580	-1.94764	-2.26798
O	-2.01718	4.78990	-3.14298	O	-4.36735	-1.69756	-3.33249
N	-1.22006	5.83542	-1.22592	N	-5.76014	-1.11814	-1.57075
C	-0.00306	6.35015	-1.82386	C	-6.22990	0.12542	-2.12133
N	1.21619	5.85911	-1.22864	N	-5.72822	1.30877	-1.44297
C	2.06071	4.97822	-1.88612	C	-5.07328	2.30094	-2.15740
O	1.83330	4.42024	-2.94222	O	-4.62426	2.20984	-3.28337
N	3.22458	4.88019	-1.13545	C	-6.35118	1.82569	-0.23395
C	4.44717	4.36891	-1.72672	H	-7.45004	1.80003	-0.33547
N	4.96062	3.18846	-1.07686	C	-6.31045	-1.74041	-0.39101
C	5.18965	2.02081	-1.80056	H	-7.41399	-1.73113	-0.43347
O	4.81686	1.79882	-2.93432	C	-5.70395	-3.17845	-0.42463
N	5.94976	1.18748	-1.00100	H	-6.45942	-3.96674	-0.59063
C	6.47018	-0.07419	-1.48221	C	-3.21225	-5.72835	-0.30288
N	5.86573	-1.23772	-0.87348	H	-4.00151	-6.48107	-0.47544
C	5.27044	-2.21881	-1.66571	C	-1.78494	-6.34817	-0.16839
O	4.98493	-2.11225	-2.83909	H	-1.78890	-7.45160	-0.13531
N	5.11839	-3.34766	-0.87654	C	1.78672	-6.36526	0.15555
C	4.70786	-4.61876	-1.43378	H	1.78115	-7.46841	0.11724
N	3.42623	-5.09958	-0.97294	C	3.21908	-5.75814	0.29297
C	2.32856	-5.18598	-1.81280	H	4.00186	-6.51779	0.46395
O	2.27769	-4.83150	-2.97177	C	5.71907	-3.20591	0.42760
N	1.31027	-5.81500	-1.10077	H	6.48174	-3.98925	0.58418
C	0.17479	-6.37471	-1.80520	C	6.31422	-1.76247	0.40052

H	7.41722	-1.74467	0.45481	H	5.20992	5.16732	-1.70640
C	6.33635	1.80169	0.24599	H	4.22744	4.09749	-2.76557
H	7.43431	1.77950	0.35755	H	7.56053	-0.09364	-1.31260
C	5.75649	3.24604	0.13372	H	6.26316	-0.12412	-2.55742
H	6.53491	4.02440	0.04668	H	5.47597	-5.37429	-1.19882
C	3.23262	5.79435	-0.00697	H	4.63586	-4.48929	-2.51967
H	4.01329	6.56217	-0.13987	H	0.18726	-7.47374	-1.70807
C	1.78284	6.37492	-0.00705	H	0.27963	-6.09654	-2.86011
H	1.75152	7.47773	-0.00084	H	-3.90881	-4.04791	-3.16679
C	-1.78737	6.38756	-0.00551	H	-5.02204	-5.09060	-2.21503
H	-1.74823	7.48990	-0.02634	H	-7.33315	0.13735	-2.09476
C	-4.45714	4.40001	1.72641	H	-5.88570	0.17743	-3.16047
H	-0.00060	6.07526	2.86650	H	-4.23772	4.13133	2.76605
H	0.00687	7.47380	1.73867	H	-5.21925	5.19889	1.70379
H	4.41659	4.57934	2.94688	C	-2.59074	1.02755	6.39985
H	5.37261	5.43691	1.69117	N	-2.24736	0.34703	5.14738
H	7.30909	0.11981	2.11452	C	-2.26476	-1.01969	5.08365
H	5.85194	0.15020	3.16746	C	-2.08776	-1.67989	3.90749
H	3.93153	-4.10293	3.16614	C	-1.91313	-0.95554	2.69823
H	5.03553	-5.14104	2.19830	N	-1.78515	-1.66636	1.55113
H	-0.27620	-6.10817	2.84833	N	-1.75293	-0.87976	0.48637
H	-0.19492	-7.48023	1.68948	C	-1.54471	-1.46537	-0.66468
H	-4.61592	-4.46812	2.51942	H	-1.37312	-2.54472	-0.73873
H	-5.46202	-5.35079	1.20058	C	-1.52828	-0.65634	-1.83966
H	-6.24496	-0.10249	2.56658	C	-1.33998	-1.22741	-3.11729
H	-7.55123	-0.07971	1.33103	C	-1.39526	-0.44427	-4.23547
H	-5.38939	5.46774	-1.68121	N	-1.59913	0.89601	-4.14888
H	-4.44657	4.60694	-2.94465	C	-1.78545	1.70772	-5.35856
H	-0.00494	7.45169	-1.76299	C	-1.73828	1.48598	-2.93249
H	-0.00599	6.04039	-2.87496	C	-1.70500	0.74673	-1.78753

C	-1.86672	0.46354	2.80699	H	1.50280	-2.51980	0.75893
C	-2.04512	1.07071	4.01161	C	1.55390	-0.62204	1.85271
H	-2.20282	0.45816	7.24783	C	1.40221	-1.19943	3.13248
H	-3.67800	1.12191	6.49502	C	1.42311	-0.41191	4.24869
H	-2.14537	2.02446	6.40435	N	1.55800	0.93691	4.15807
H	-2.44136	-1.53613	6.02128	C	1.71383	1.75956	5.36455
H	-2.11779	-2.76358	3.87409	C	1.65758	1.53058	2.93979
H	-1.18583	-2.29600	-3.22993	C	1.65426	0.78830	1.79639
H	-1.29469	-0.85222	-5.23535	C	1.85224	0.49872	-2.79976
H	-1.42827	2.72201	-5.16801	C	2.01352	1.10742	-4.00613
H	-2.84953	1.74619	-5.61308	H	2.21063	0.49112	-7.23882
H	-1.22261	1.26544	-6.18349	H	3.65970	1.20502	-6.48111
H	-1.88948	2.55954	-2.94731	H	2.09781	2.05703	-6.40007
H	-1.82871	1.23773	-0.82968	H	2.51708	-1.49040	-6.00344
H	-1.70938	1.07518	1.92881	H	2.22983	-2.72082	-3.85277
H	-2.05609	2.15166	4.11442	H	1.30271	-2.27438	3.24802
C	2.57567	1.07532	-6.39072	H	1.34997	-0.82203	5.25001
N	2.24925	0.38749	-5.13775	H	1.18349	1.29023	6.19600
C	2.31731	-0.97729	-5.06864	H	2.77715	1.85405	5.60751
C	2.16054	-1.63909	-3.89051	H	1.30242	2.75356	5.17625
C	1.95435	-0.91697	-2.68489	H	1.74909	2.61101	2.95202
N	1.85465	-1.62867	-1.53531	H	1.74444	1.28272	0.83652
N	1.78277	-0.84052	-0.47365	H	1.66786	1.10798	-1.92514
C	1.61072	-1.43257	0.68017	H	1.98441	2.18765	-4.11295

mD_2R$_a$ $^+$⊂CB8: local minimum on the potential energy surface at the r2scan-3c level of theory.

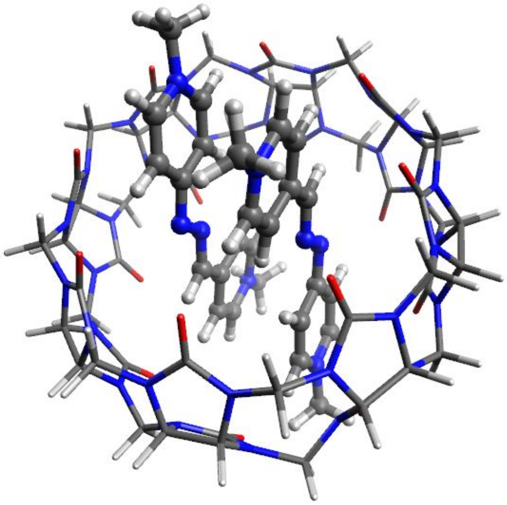

208

C	6.32833	3.00064	-0.04015
H	7.39117	3.28276	-0.12125
N	5.91590	2.25286	-1.21449
C	4.97002	2.93553	-1.96781
O	4.54762	2.60490	-3.05930
N	4.62495	4.07836	-1.26986
C	3.78591	5.10440	-1.84835
N	2.47238	5.22201	-1.25299
C	1.32086	5.05043	-2.00734
O	1.26260	4.57320	-3.12466
N	0.25861	5.54438	-1.26843
C	-1.02389	5.81227	-1.88341
N	-2.13001	5.10261	-1.28334
C	-2.96868	4.30850	-2.05495
O	-2.76004	3.93373	-3.19095
N	-4.10535	4.05446	-1.30335
C	-5.27800	3.45140	-1.89006
N	-5.65463	2.18575	-1.29298
C	-5.71995	1.03084	-2.05826
O	-5.27212	0.87943	-3.17868
N	-6.42781	0.09131	-1.32820
C	-6.68792	-1.23813	-1.82124
N	-5.88623	-2.27802	-1.19816
C	-4.98643	-3.01795	-1.95592
O	-4.57470	-2.72821	-3.06393
N	-4.67782	-4.15955	-1.24363
C	-3.80738	-5.18786	-1.77091
N	-2.49339	-5.24457	-1.16637
C	-1.34701	-5.08519	-1.94002
O	-1.30975	-4.65608	-3.07496
N	-0.27508	-5.55662	-1.19979
C	1.00847	-5.80925	-1.81792
N	2.11454	-5.09413	-1.22100
C	2.94952	-4.30051	-1.99764
O	2.74413	-3.93519	-3.13611
N	4.08745	-4.03708	-1.24600
C	5.26424	-3.45470	-1.84473
N	5.65875	-2.18925	-1.25876

C	5.75593	-1.04860	-2.04249
O	5.32394	-0.90535	-3.16892
N	6.47885	-0.11319	-1.32039
C	6.94791	-0.61267	-0.05212
H	8.04497	-0.51507	0.01626
N	6.32445	-0.04912	1.13385
C	6.76226	1.20237	1.72413
N	6.07175	2.37229	1.23459
C	5.04806	2.97645	1.94126
O	4.65331	2.65752	3.04754
N	4.58562	4.03845	1.18124
C	3.78014	5.08702	1.77753
N	2.46832	5.23303	1.19456
C	1.31383	5.05180	1.94477
O	1.26201	4.61434	3.07595
N	0.24925	5.51629	1.18656
C	-1.03871	5.77229	1.79491
N	-2.14467	5.07911	1.17218
C	-2.99622	4.27905	1.92336
O	-2.81228	3.89737	3.05985
N	-4.12425	4.03061	1.14841
C	-5.32659	3.48366	1.73584
N	-5.74731	2.22186	1.16694
C	-5.75210	1.05534	1.91086
O	-5.35127	0.92225	3.04915
N	-6.33754	0.06451	1.12754
C	-6.77147	-1.18244	1.72558
N	-6.07177	-2.35451	1.25181
C	-5.01447	-2.91293	1.94651
O	-4.59828	-2.55502	3.03151

N	-4.54399	-3.98736	1.20120
C	-3.77230	-5.03407	1.84667
N	-2.46318	-5.24551	1.28075
C	-1.30434	-5.02306	2.00349
O	-1.23547	-4.51619	3.10754
N	-0.24509	-5.50984	1.25403
C	1.04396	-5.76252	1.86217
N	2.14286	-5.06549	1.23428
C	2.99819	-4.26546	1.98106
O	2.81208	-3.87731	3.11626
N	4.12365	-4.02288	1.20524
C	5.32579	-3.46564	1.78379
N	5.74442	-2.20936	1.20130
C	5.73303	-1.03039	1.92442
O	5.31440	-0.88109	3.05558
C	6.45901	-2.09651	-0.05055
H	7.28522	-2.82746	-0.08542
C	4.12928	-4.79540	-0.01772
H	4.98955	-5.48875	-0.02489
C	2.75040	-5.52734	0.00376
H	2.84336	-6.62844	0.01663
C	-0.68306	-6.17420	0.04167
H	-0.37856	-7.23553	0.06077
C	-2.23245	-5.97468	0.05503
H	-2.79618	-6.92416	0.06190
C	-5.36304	-4.23484	0.02479
H	-5.89260	-5.19750	0.12104
C	-6.32110	-3.00113	-0.01270
H	-7.38552	-3.27468	-0.10140
C	-6.93434	0.60641	-0.07889

H	-8.03216	0.50074	-0.03917
C	-6.45698	2.09442	-0.08600
H	-7.28894	2.81839	-0.13001
C	-4.13829	4.80772	-0.06979
H	-4.99831	5.50132	-0.07003
C	-2.75865	5.53899	-0.05399
H	-2.85068	6.64015	-0.04570
C	0.68107	6.16792	-0.03110
H	0.38397	7.23159	-0.02287
C	2.22893	5.95836	-0.02997
H	2.79869	6.90446	-0.03530
C	5.35822	4.22731	-0.03319
H	5.88027	5.19867	-0.00947
C	6.73380	1.21501	-1.81829
H	3.64390	4.85163	-2.90523
H	4.30667	6.07288	-1.76413
H	-0.96021	5.49613	-2.93064
H	-1.22049	6.89839	-1.83726
H	-6.12628	4.15364	-1.81078
H	-5.05434	3.26258	-2.94618
H	-6.45469	-1.23797	-2.89221
H	-7.75466	-1.47343	-1.67488
H	-3.66777	-4.97692	-2.83718
H	-4.30600	-6.16337	-1.64677
H	0.93706	-5.48781	-2.86303
H	1.21580	-6.89395	-1.77843
H	5.03683	-3.26919	-2.90072
H	6.10421	-4.16740	-1.76619
H	7.84174	1.32853	1.54426
H	6.57251	1.14212	2.80188
H	4.32258	6.04464	1.69361
H	3.64190	4.83262	2.83457
H	-1.23626	6.85953	1.77287
H	-0.98253	5.43125	2.83473
H	-6.14707	4.21427	1.63216
H	-5.12196	3.30797	2.79817
H	-7.84844	-1.32016	1.54074
H	-6.58785	-1.10848	2.80352
H	-4.34144	-5.97869	1.80097
H	-3.63079	-4.73991	2.89293
H	0.99061	-5.42192	2.90228
H	1.24261	-6.84926	1.83969
H	6.14784	-4.19495	1.68555
H	5.12282	-3.28101	2.84477
H	6.51507	1.20270	-2.89228
H	7.79525	1.46248	-1.65818
C	2.80991	-0.18512	-5.85470
N	2.08441	-0.52507	-4.62573
C	1.08492	-1.45262	-4.63979
C	0.49020	-1.87618	-3.48982
C	0.93165	-1.38641	-2.23357
N	0.34182	-1.88555	-1.11448
N	0.88529	-1.38252	-0.01938
C	0.40131	-1.78479	1.12789
H	-0.42091	-2.50566	1.18865
C	0.99298	-1.27189	2.32074
C	0.62465	-1.76741	3.59213
C	1.26415	-1.32069	4.71413
N	2.23272	-0.37051	4.64174
C	3.00374	0.01305	5.83127

C	2.56850	0.17160	3.44125	C	-0.42226	1.81430	-0.99937
C	1.97230	-0.25333	2.29137	H	0.38837	2.54950	-1.05360
C	1.95473	-0.39855	-2.26053	C	-0.99978	1.29294	-2.19578
C	2.50003	0.00132	-3.44271	C	-0.62887	1.78812	-3.46611
H	2.15208	-0.31346	-6.71683	C	-1.25150	1.32574	-4.59171
H	3.68594	-0.83336	-5.96173	N	-2.20305	0.35921	-4.52361
H	3.13957	0.85477	-5.80323	C	-2.95985	-0.04136	-5.71671
H	0.81255	-1.84507	-5.61400	C	-2.53972	-0.18326	-3.32360
H	-0.28500	-2.63435	-3.52468	C	-1.96393	0.25925	-2.17002
H	-0.12267	-2.54934	3.68088	C	-1.98060	0.41366	2.38432
H	1.05269	-1.71558	5.70198	C	-2.52546	0.00997	3.56543
H	3.22274	1.08231	5.78976	H	-2.12912	0.24275	6.83502
H	3.94470	-0.54607	5.85347	H	-3.18986	-0.84643	5.90732
H	2.42415	-0.20546	6.72993	H	-3.65408	0.85803	6.13934
H	3.33354	0.94022	3.45670	H	-0.81658	1.82911	5.74352
H	2.24787	0.20380	1.34985	H	0.27164	2.63292	3.65069
H	2.30808	0.04341	-1.33832	H	0.10565	2.58234	-3.55643
H	3.28894	0.74381	-3.49962	H	-1.03916	1.72168	-5.57891
C	-2.81191	0.17498	5.98520	H	-3.90820	0.50458	-5.74610
N	-2.10028	0.52359	4.75096	H	-2.37644	0.18045	-6.61211
C	-1.09669	1.44785	4.76698	H	-3.16514	-1.11320	-5.67005
C	-0.50649	1.87807	3.61774	H	-3.28586	-0.97017	-3.34625
C	-0.95264	1.39584	2.35975	H	-2.23853	-0.19925	-1.22887
N	-0.36393	1.90108	1.24323	H	-2.34252	-0.01826	1.46074
N	-0.90226	1.39927	0.14518	H	-3.32239	-0.72440	3.61579

R_aH^{2+}·HQ⊂CB8: local minimum on the potential energy surface at the r2scan-3c level of theory.

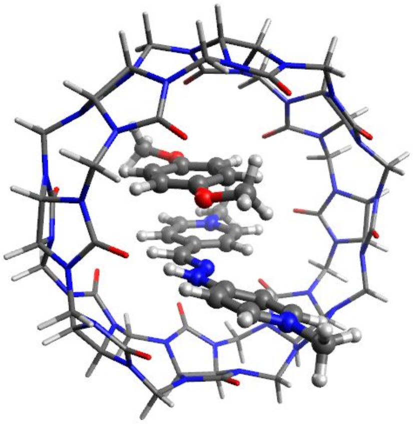

197

C	6.24311	1.15148	-0.53073
H	7.11941	1.74610	-0.84209
N	5.90404	1.42330	0.84086
C	5.79296	0.27591	1.61115
O	5.56772	0.22258	2.79872
N	6.02419	-0.81127	0.76300
C	6.36650	-2.09905	1.32958
N	5.53576	-3.19737	0.88584
C	4.46214	-3.65706	1.62900
O	4.09007	-3.23873	2.70250
N	3.90555	-4.73130	0.92648
C	3.07094	-5.67862	1.63148
N	1.74457	-5.82472	1.06839
C	0.61046	-5.52638	1.80241
O	0.56889	-4.92260	2.85877
N	-0.47297	-6.06902	1.13252
C	-1.79892	-6.08496	1.70040
N	-2.72162	-5.14036	1.09967
C	-3.44946	-4.27007	1.90747
O	-3.20865	-3.98831	3.06339
N	-4.51741	-3.80342	1.15548
C	-5.61038	-3.06739	1.75055
N	-5.73065	-1.69333	1.31593
C	-5.44124	-0.62949	2.14988
O	-5.02415	-0.70098	3.28807
N	-5.73682	0.54489	1.45494
C	-6.00826	1.76248	2.21252
N	-5.24111	2.91034	1.80875
C	-4.08866	3.30428	2.47249
O	-3.61485	2.75976	3.45223
N	-3.60614	4.43824	1.83796
C	-2.70124	5.34407	2.53819
N	-1.44553	5.56016	1.86793
C	-0.23603	5.26353	2.49303
O	-0.09478	4.68056	3.54577
N	0.76965	5.80442	1.70799
C	2.15509	5.77483	2.10885
N	2.99257	4.90262	1.30316
C	3.78251	3.94271	1.93300
O	3.64385	3.52500	3.06374
N	4.77979	3.57665	1.04265
C	4.68078	4.26626	-0.21532

H	5.65975	4.68497	-0.50744
N	4.10149	3.49966	-1.31262
C	4.89410	2.59715	-2.11877
N	5.15758	1.31304	-1.49190
C	4.93003	0.14130	-2.21015
O	4.18017	0.00400	-3.15582
N	5.74108	-0.84419	-1.66714
C	5.85541	-2.15573	-2.24516
N	5.12914	-3.20190	-1.53878
C	4.24193	-4.00810	-2.23161
O	3.73734	-3.76718	-3.31459
N	4.04505	-5.15715	-1.48581
C	3.15276	-6.20897	-1.91690
N	1.81563	-6.16548	-1.35601
C	0.70997	-5.79780	-2.10449
O	0.71940	-5.37765	-3.24506
N	-0.41697	-6.01777	-1.32054
C	-1.73080	-6.07396	-1.93356
N	-2.69988	-5.17673	-1.35276
C	-3.15948	-4.05103	-2.01697
O	-2.75360	-3.62810	-3.07988
N	-4.19316	-3.51434	-1.25415
C	-5.13181	-2.58574	-1.84275
N	-5.30559	-1.36355	-1.07891
C	-5.16247	-0.13307	-1.71809
O	-4.55323	0.06962	-2.75032
N	-5.85334	0.80594	-0.97863
C	-5.94783	2.19397	-1.36980
N	-5.08347	3.09304	-0.62760
C	-4.23753	3.94767	-1.34560
O	-3.86232	3.78319	-2.48600
N	-3.94662	5.02257	-0.52696
C	-3.10143	6.11593	-0.96526
N	-1.73244	6.04115	-0.52052
C	-0.68828	5.83862	-1.41564
O	-0.80110	5.56329	-2.59109
N	0.49430	6.04957	-0.72305
C	1.73879	6.23916	-1.44294
N	2.77360	5.29147	-1.11643
C	3.05156	4.19432	-1.91500
O	2.51224	3.91832	-2.96466
C	3.60010	5.35459	0.05628
H	4.01907	6.36810	0.17966
C	0.26759	6.58740	0.60523
H	0.65100	7.62099	0.66623
C	-1.28285	6.49615	0.77248
H	-1.75282	7.46400	1.02160
C	-4.51532	4.89677	0.78891
H	-5.00794	5.83505	1.09187
C	-5.49962	3.69803	0.63200
H	-6.55847	4.00281	0.59007
C	-6.44414	0.25564	0.21136
H	-7.50303	0.55237	0.29409
C	-6.24331	-1.27987	0.03436
H	-7.17536	-1.81922	-0.20464
C	-4.57181	-4.39178	-0.15940
H	-5.56711	-4.83223	-0.34364
C	-3.42436	-5.44502	-0.13760
H	-3.78645	-6.48763	-0.12666
C	-0.09773	-6.73152	-0.09337

H	-0.51122	-7.75368	-0.12207
C	1.46251	-6.69320	-0.05970
H	1.92384	-7.68368	0.09113
C	4.72555	-5.11022	-0.21406
H	5.25545	-6.05818	-0.02336
C	5.67806	-3.88562	-0.36840
H	6.72989	-4.16465	-0.54452
C	6.50782	-0.38531	-0.53879
H	7.57306	-0.64625	-0.66356
C	5.89460	2.74467	1.42866
H	6.24352	-2.01065	2.41530
H	7.41473	-2.34691	1.09443
H	2.95316	-5.31008	2.65725
H	3.56346	-6.66674	1.64804
H	-2.20728	-7.10590	1.60992
H	-1.71037	-5.81905	2.75968
H	-5.44149	-3.04912	2.83313
H	-6.55459	-3.59087	1.53114
H	-5.76203	1.55008	3.25880
H	-7.07892	2.01029	2.12987
H	-2.47437	4.89326	3.51136
H	-3.21820	6.30868	2.68697
H	2.19085	5.39992	3.13844
H	2.56019	6.80128	2.07585
H	5.86005	3.07461	-2.37113
H	4.32975	2.40756	-3.03919
H	6.92327	-2.42689	-2.29219
H	5.44191	-2.11039	-3.25868
H	3.60938	-7.17790	-1.66740
H	3.04758	-6.11985	-3.00373
H	-2.12061	-7.10392	-1.86977
H	-1.60751	-5.79286	-2.98560
H	-6.11373	-3.07972	-1.95987
H	-4.74449	-2.30861	-2.82984
H	-6.99765	2.51271	-1.26021
H	-5.65164	2.25763	-2.42325
H	-3.08977	6.09515	-2.06110
H	-3.55014	7.05777	-0.61361
H	2.12065	7.25473	-1.24861
H	1.51326	6.12717	-2.50955
H	5.84569	2.61091	2.51545
H	6.82947	3.26240	1.15881
C	-0.64981	-2.08808	6.74459
N	-0.96382	-1.63811	5.37736
C	-1.78367	-0.56884	5.19071
C	-2.14645	-0.15727	3.94134
C	-1.66704	-0.86504	2.82402
N	-2.04475	-0.48074	1.56988
N	-1.43943	-1.02764	0.50711
C	-1.76865	-0.62302	-0.67165
H	-2.53603	0.13489	-0.85917
C	-1.02859	-1.14087	-1.80520
C	-1.40824	-0.80509	-3.10912
C	-0.66515	-1.25774	-4.17290
N	0.42892	-2.02183	-3.97997
C	1.26395	-2.46583	-5.11254
C	0.81908	-2.36527	-2.72807
C	0.10954	-1.94873	-1.63599
C	-0.81202	-1.95968	3.03292
C	-0.48775	-2.32220	4.31053

H	-0.55747	-1.21983	7.39964
H	0.29651	-2.63028	6.73575
H	-1.44562	-2.74352	7.11158
H	-2.12471	-0.06039	6.08553
H	-2.78546	0.71309	3.83093
H	-2.28827	-0.19757	-3.29194
H	-0.91792	-1.02382	-5.20063
H	1.48825	-3.52656	-4.98380
H	2.19574	-1.89249	-5.11161
H	0.71998	-2.30307	-6.04317
H	1.71961	-2.96598	-2.67336
H	0.45537	-2.21119	-0.64346
H	-0.41890	-2.54154	2.21156
H	0.15157	-3.17521	4.50698
H	-2.60067	0.36978	1.47253
C	-0.77374	2.42705	-1.36967
C	-0.96062	2.51980	0.00485
C	-0.04803	1.95118	0.89433
C	1.09486	1.31482	0.39319
C	1.28516	1.22600	-0.98020
C	0.35189	1.76130	-1.87096
O	0.59651	1.56697	-3.19319
H	-1.49610	2.88841	-2.03534
H	-1.82095	3.04861	0.40741
H	1.83775	0.89238	1.06180
H	2.16490	0.73671	-1.38931
C	-0.10647	2.41308	-4.11530
H	0.35154	2.22980	-5.08870
H	0.01490	3.46586	-3.84245
H	-1.17534	2.16195	-4.15646
O	-0.37764	2.03182	2.21560
C	0.64012	1.72312	3.17537
H	0.90083	0.65522	3.14666
H	0.21333	1.97905	4.14530
H	1.53768	2.32889	3.01416

$R_a^{+\cdot}$·HQ⊂CB8: local minimum on the potential energy surface at the r2scan-3c level of theory.

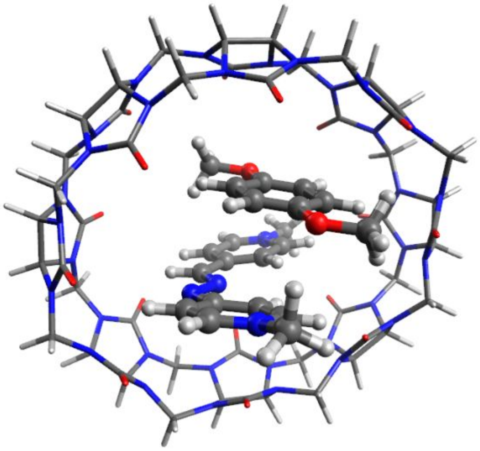

196

C	6.34378	1.35526	-0.64458
H	7.24690	1.97762	-0.77832

N	5.85404	1.47383	0.71542	N	-1.87560	5.48229	1.55005
C	5.99030	0.29591	1.43159	C	-0.74313	4.93923	2.13950
O	5.85177	0.15188	2.62759	O	-0.71786	4.24925	3.13618
N	6.36628	-0.69354	0.52821	N	0.35887	5.36879	1.39862
C	6.74172	-2.01784	0.95833	C	1.67435	5.36062	2.01249
N	5.83052	-3.07733	0.56821	N	2.68529	4.67720	1.23809
C	4.82883	-3.52758	1.41050	C	3.52052	3.74991	1.86411
O	4.63929	-3.17288	2.55471	O	3.36577	3.25997	2.95951
N	4.10453	-4.49768	0.71686	N	4.59875	3.53519	1.01287
C	3.26063	-5.40983	1.46559	C	4.55370	4.36253	-0.16099
N	1.89550	-5.47830	1.00349	H	5.51165	4.89936	-0.28901
C	0.83491	-4.96330	1.73285	N	4.17334	3.70623	-1.40228
O	0.91840	-4.32261	2.76102	C	5.12414	2.95848	-2.19441
N	-0.34076	-5.34965	1.09301	N	5.39491	1.63092	-1.69691
C	-1.58726	-5.39482	1.83524	C	5.05460	0.50290	-2.42687
N	-2.67190	-4.67561	1.20779	O	4.36770	0.45953	-3.42299
C	-3.49898	-3.86751	1.99295	N	5.69434	-0.58057	-1.82522
O	-3.26033	-3.47086	3.11273	C	5.78093	-1.85000	-2.49510
N	-4.65958	-3.64824	1.27096	N	5.12348	-2.93596	-1.78834
C	-5.76347	-2.87783	1.79462	C	4.14131	-3.67976	-2.42070
N	-5.92577	-1.57341	1.19356	O	3.53445	-3.37621	-3.43075
C	-5.88188	-0.42769	1.98231	N	3.99872	-4.86513	-1.71724
O	-5.52867	-0.36664	3.13945	C	3.01528	-5.85352	-2.08939
N	-6.38696	0.61625	1.22030	N	1.73270	-5.71982	-1.42897
C	-6.65723	1.91248	1.80142	C	0.56512	-5.55297	-2.16704
N	-5.78119	2.97888	1.37328	O	0.49620	-5.26951	-3.34655
C	-4.72295	3.42145	2.14874	N	-0.49784	-5.79766	-1.31672
O	-4.45333	3.04954	3.27240	C	-1.85178	-5.92862	-1.81074
N	-4.06307	4.41189	1.42677	N	-2.79457	-5.01591	-1.21695
C	-3.19113	5.34062	2.12241	C	-3.47097	-4.06596	-1.97410

O	-3.22175	-3.76169	-3.12131	H	-6.89483	4.02723	-0.06055
N	-4.50676	-3.57097	-1.18741	C	-6.90217	0.18018	-0.05415
C	-5.62170	-2.88614	-1.81665	H	-7.97023	0.44737	-0.14431
N	-5.91415	-1.58929	-1.26566	C	-6.64955	-1.36348	-0.04266
C	-5.66914	-0.42490	-1.97282	H	-7.58097	-1.95760	-0.04561
O	-5.13965	-0.34520	-3.06396	C	-4.68787	-4.35934	0.02191
N	-6.18004	0.62209	-1.22847	H	-5.61360	-4.95773	-0.05458
C	-6.24840	1.96307	-1.75404	C	-3.39508	-5.22690	0.07368
N	-5.40810	2.91469	-1.05792	H	-3.59781	-6.30216	0.23153
C	-4.42208	3.60590	-1.75931	C	-0.08244	-6.25654	-0.01148
O	-3.96849	3.30786	-2.84270	H	-0.52008	-7.24790	0.20263
N	-4.09453	4.72464	-1.00887	C	1.47443	-6.27923	-0.11613
C	-3.18098	5.73043	-1.48795	H	1.90372	-7.29435	-0.04524
N	-1.85926	5.68316	-0.89353	C	4.78814	-4.88303	-0.51382
C	-0.73975	5.70060	-1.72576	H	5.28895	-5.85855	-0.39378
O	-0.71862	5.44800	-2.90998	C	5.77718	-3.69795	-0.72884
N	0.33849	6.10022	-0.95118	H	6.78389	-4.01656	-1.04752
C	1.65447	6.29730	-1.51343	C	6.62164	-0.17474	-0.79264
N	2.63504	5.30818	-1.13744	H	7.65902	-0.40236	-1.09838
C	3.13419	4.37828	-2.04084	C	5.73894	2.74633	1.40119
O	2.75893	4.21480	-3.18071	H	6.76871	-2.00196	2.05408
C	3.34961	5.31167	0.11031	H	7.73938	-2.26474	0.56299
H	3.65507	6.34117	0.37238	H	3.24154	-5.04721	2.50004
C	-0.01886	6.37574	0.41535	H	3.69369	-6.42521	1.43582
H	0.36092	7.36695	0.72036	H	-1.87385	-6.45432	1.98205
C	-1.57585	6.28893	0.39818	H	-1.41308	-4.92756	2.81116
H	-2.06980	7.27374	0.47788	H	-5.57277	-2.72019	2.86280
C	-4.81047	4.79388	0.23644	H	-6.69170	-3.45971	1.66135
H	-5.25813	5.79446	0.36941	H	-6.53289	1.81169	2.88591
C	-5.86873	3.65467	0.10471	H	-7.69541	2.19931	1.56927

H	-3.06902	4.95737	3.14211
H	-3.66572	6.33713	2.15341
H	1.59039	4.83203	2.96946
H	1.98945	6.40607	2.19613
H	6.07838	3.51461	-2.25219
H	4.69549	2.85863	-3.19845
H	6.84259	-2.11403	-2.65104
H	5.28061	-1.74520	-3.46531
H	3.43519	-6.85053	-1.88065
H	2.83215	-5.74752	-3.16463
H	-2.19707	-6.96270	-1.63531
H	-1.82344	-5.72628	-2.88737
H	-6.52317	-3.51984	-1.74023
H	-5.35932	-2.74250	-2.87139
H	-7.29557	2.31297	-1.72166
H	-5.90407	1.91984	-2.79424
H	-3.04958	5.57173	-2.56475
H	-3.63165	6.72257	-1.30961
H	2.02138	7.29206	-1.21260
H	1.54579	6.25225	-2.60346
H	5.64132	2.52322	2.47005
H	6.65507	3.33767	1.22630
C	-0.28663	-0.86768	6.24010
N	-0.97781	-0.78965	4.95164
C	-1.96328	0.13242	4.76314
C	-2.52477	0.33283	3.53828
C	-2.05852	-0.39039	2.41514
N	-2.59200	-0.08060	1.19804
N	-1.93183	-0.66917	0.22207
C	-2.30175	-0.38298	-1.00387

H	-3.13886	0.29225	-1.21182
C	-1.53450	-0.91392	-2.07906
C	-1.90598	-0.68552	-3.42427
C	-1.15021	-1.19236	-4.44341
N	-0.01647	-1.89890	-4.19965
C	0.76139	-2.48552	-5.29935
C	0.38620	-2.11404	-2.91691
C	-0.34085	-1.64863	-1.86297
C	-1.04744	-1.35918	2.65051
C	-0.53921	-1.53243	3.90007
H	0.50496	-0.11146	6.28129
H	0.15733	-1.85890	6.35161
H	-1.00017	-0.70336	7.05098
H	-2.26405	0.69724	5.63888
H	-3.30657	1.07314	3.42223
H	-2.80717	-0.12856	-3.65570
H	-1.41421	-1.05742	-5.48638
H	0.49501	-3.54085	-5.41357
H	1.82497	-2.41009	-5.06494
H	0.54804	-1.94039	-6.22039
H	1.32034	-2.65385	-2.80917
H	0.02303	-1.79705	-0.85340
H	-0.66078	-1.96049	1.83904
H	0.23669	-2.25970	4.10933
C	0.72059	1.64078	0.90266
C	0.94033	1.30496	2.23131
C	1.86591	0.32163	2.57287
C	2.57661	-0.33390	1.56513
C	2.36506	0.01284	0.23243
C	1.44121	0.99993	-0.11064

O	1.31042	1.26999	-1.44597	H	0.58627	3.22631	-1.32614
H	-0.02323	2.39443	0.67237	H	-0.66101	1.96360	-1.56611
H	0.39135	1.81634	3.01620	O	1.99256	0.05260	3.91055
H	3.30039	-1.10412	1.80762	C	2.96171	-0.92983	4.28076
H	2.91943	-0.48428	-0.56052	H	2.70559	-1.91962	3.87797
C	0.36446	2.26947	-1.81836	H	2.94952	-0.96312	5.37303
H	0.46225	2.39869	-2.89633	H	3.96373	-0.65365	3.93539

MV^{2+}⊂CB7: local minimum on the potential energy surface at the r2scan-3c level of theory.

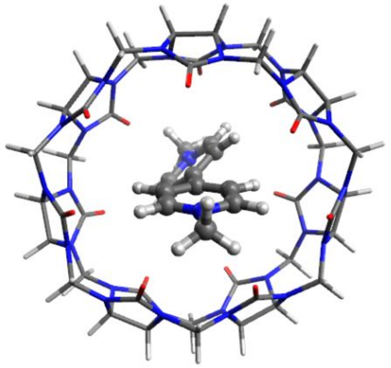

154

C	-1.05376	0.63042	5.24083
N	-0.79094	0.68031	3.78754
C	0.42023	1.08161	3.35413
C	0.72351	1.04654	2.01006
C	-1.73556	0.24248	2.92943
C	-1.47789	0.19569	1.57835
C	-0.22806	0.59379	1.09374
C	0.06371	0.55169	-0.35118
C	0.77904	1.58013	-0.97016
C	0.98231	1.55252	-2.33273
N	0.50161	0.52834	-3.06900
C	0.69337	0.54353	-4.53561
C	-0.14684	-0.50801	-2.49614
C	-0.38047	-0.51426	-1.13917
H	-0.42825	1.37114	5.73933
H	-0.80596	-0.37435	5.59484
H	-2.10731	0.84835	5.42033
H	1.13393	1.41980	4.09762
H	1.71522	1.35416	1.69225
H	-2.68768	-0.07324	3.34118
H	-2.27281	-0.13016	0.91650
H	1.15248	2.43071	-0.40933
H	1.50808	2.34530	-2.85669
H	1.64811	0.06668	-4.77635
H	0.68557	1.58120	-4.87033
H	-0.13360	0.00270	-4.99668
H	-0.48552	-1.31276	-3.13982
H	-0.89755	-1.36557	-0.70779
C	3.14289	-4.12564	0.47870
N	1.70901	-4.20757	0.69027
C	1.13307	-4.69544	1.93226
C	-0.35021	-4.97818	1.54292
N	-0.34097	-4.89365	0.10657
C	0.86994	-4.43823	-0.40036
O	1.15024	-4.29526	-1.56807
C	-1.42146	-5.31168	-0.75703
N	-2.53047	-4.38443	-0.87287
C	-3.58505	-4.22981	0.09445
C	-4.61494	-3.32597	-0.64393
N	-3.81278	-2.75363	-1.72401

C	-2.62069	-3.45867	-1.89718	C	5.25596	0.25464	0.25324
O	-1.82085	-3.29839	-2.79693	N	4.53486	-0.81606	0.90015
C	-4.41895	-2.11553	-2.88607	C	4.88158	-1.29451	2.22847
N	-4.60469	-0.69210	-2.76684	C	4.08669	-2.62943	2.32338
C	-5.64780	-0.07219	-1.98446	N	3.75533	-2.90904	0.95281
C	-5.42316	1.45112	-2.23821	C	3.92059	-1.80368	0.13211
N	-4.12804	1.48352	-2.90483	O	3.61743	-1.71986	-1.03816
C	-3.68353	0.21768	-3.25016	N	2.96478	-2.26435	3.18824
O	-2.68032	-0.04176	-3.89173	C	2.11192	-3.26310	3.80922
C	-3.64430	2.64986	-3.61119	N	1.01535	-3.71899	2.98969
N	-2.96068	3.60392	-2.76611	C	-0.26487	-3.21839	3.11453
C	-3.67453	4.60621	-1.97871	O	-0.62613	-2.34402	3.88357
C	-2.52103	5.54036	-1.51200	N	-1.07270	-3.91250	2.22496
N	-1.44895	5.20656	-2.41624	C	-2.50836	-3.94681	2.39402
C	-1.66886	4.00753	-3.06914	N	-3.24416	-3.42085	1.26149
O	-0.88386	3.42761	-3.79840	C	-4.15409	-2.38828	1.45176
C	-0.19996	5.92180	-2.50992	O	-4.18439	-1.62641	2.39936
N	0.75061	5.63577	-1.45180	N	-5.03127	-2.40335	0.38238
C	0.73297	6.31755	-0.16807	C	-6.00171	-1.34725	0.19113
C	2.15139	6.02203	0.41715	N	-5.50833	-0.19417	-0.54168
N	2.82762	5.36087	-0.67543	C	-5.51331	1.05785	0.08529
C	2.00318	5.12084	-1.76117	O	-5.58357	1.25922	1.27533
O	2.31830	4.58256	-2.80315	N	-5.45509	2.01610	-0.91440
C	4.20430	4.92171	-0.64442	C	-5.49629	3.43229	-0.61699
N	4.46751	3.74108	0.15621	N	-4.23785	4.13529	-0.74424
C	4.78724	3.73032	1.56002	C	-3.41817	4.39222	0.34549
C	5.29441	2.27676	1.80277	O	-3.62232	4.06628	1.49251
N	4.79145	1.57680	0.62524	N	-2.32549	5.13155	-0.12437
C	4.39422	2.46386	-0.37319	C	-1.58756	5.94961	0.82371
O	4.06091	2.17202	-1.50341	N	-0.15813	5.74776	0.81337

C	0.50435	5.05936	1.81425
O	0.00105	4.47079	2.74924
N	1.87042	5.18651	1.56952
C	2.82866	4.98079	2.63670
N	3.64411	3.80072	2.46133
C	3.64666	2.78085	3.39519
O	2.90087	2.67534	4.35080
N	4.67102	1.91334	3.05405
C	4.94457	0.71285	3.81852
N	4.35117	-0.51048	3.31727
C	3.20138	-1.06422	3.85435
O	2.53025	-0.60178	4.75654
H	3.30641	-4.17839	-0.60379
H	3.64146	-4.97641	0.96953
H	1.67243	-5.58754	2.29082
H	-0.71309	-5.96433	1.87670
H	-1.81771	-6.27274	-0.39924
H	-0.99625	-5.43613	-1.75925
H	-3.99120	-5.20844	0.39598
H	-5.47838	-3.87782	-1.04712
H	-3.75008	-2.28754	-3.73675
H	-5.39689	-2.58063	-3.08589
H	-6.63772	-0.42937	-2.31201
H	-6.19548	1.90870	-2.87816
H	-4.49385	3.14577	-4.11254
H	-2.92612	2.30740	-4.36476
H	-4.43603	5.11509	-2.59186
H	-2.76607	6.61284	-1.57917
H	0.27547	5.63412	-3.45407
H	-0.42172	7.00013	-2.52106
H	0.52855	7.39224	-0.30355
H	2.69941	6.92868	0.72060
H	4.48028	4.69232	-1.67962
H	4.83667	5.73625	-0.26470
H	5.52996	4.50765	1.79959
H	6.39080	2.19598	1.87426
H	6.33243	0.16851	0.48556
H	5.10474	0.14654	-0.82726
H	5.97154	-1.42100	2.33449
H	4.66335	-3.45801	2.76526
H	2.71634	-4.13929	4.09476
H	1.68652	-2.80571	4.70924
H	-2.82631	-4.98657	2.58539
H	-2.75697	-3.32444	3.26107
H	-6.29757	-0.99454	1.18561
H	-6.87401	-1.77347	-0.32664
H	-5.81749	3.52535	0.42671
H	-6.23094	3.91541	-1.27753
H	-1.95027	5.68472	1.82371
H	-1.79008	7.01612	0.62819
H	3.47242	5.87270	2.71323
H	2.27577	4.84665	3.57386
H	6.03336	0.57523	3.87338
H	4.54279	0.87702	4.82436

MV^{2+}⊂CB8: local minimum on the potential energy surface at the r2scan-3c level of theory.

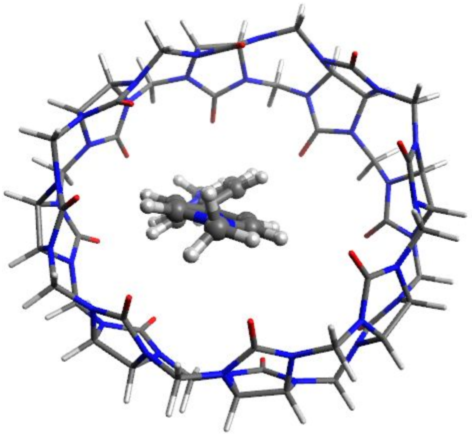

172

C	1.47948	-6.49988	-0.57074
H	1.41199	-7.55437	-0.88728
N	0.76100	-5.64902	-1.51257
C	1.60882	-4.78774	-2.18605
O	1.29768	-4.00453	-3.06933
N	2.88707	-4.99099	-1.70212
C	4.04177	-4.37571	-2.29594
N	4.57810	-3.24381	-1.55647
C	4.81442	-2.04975	-2.22041
O	4.30571	-1.69302	-3.26971
N	5.75296	-1.34356	-1.49390
C	6.35042	-0.12177	-1.97306
N	5.86256	1.09516	-1.36020
C	5.15037	2.04775	-2.06044
O	4.71948	1.94042	-3.19381
N	5.03420	3.17049	-1.24275
C	4.80061	4.47692	-1.83250
N	3.60487	5.14761	-1.37498
C	2.41766	5.06116	-2.07423
O	2.25704	4.51977	-3.15107
N	1.44011	5.72450	-1.33713
C	0.26137	6.23428	-2.02890
N	-0.99527	5.77906	-1.49775
C	-1.68095	4.69933	-2.03900
O	-1.32975	4.03936	-2.99362
N	-2.85306	4.54115	-1.30158
C	-4.01614	3.94014	-1.93298
N	-4.61931	2.87217	-1.16510
C	-4.99796	1.69664	-1.83241
O	-4.56182	1.30513	-2.88945
N	-5.98582	1.09457	-1.06721
C	-6.61686	-0.15001	-1.44919
N	-6.04019	-1.33551	-0.84579
C	-5.64414	-2.39427	-1.67334
O	-5.47061	-2.34140	-2.86714
N	-5.52948	-3.51751	-0.86639
C	-5.16622	-4.81220	-1.40281
N	-3.83885	-5.27716	-1.07017
C	-2.75168	-5.10070	-1.91553
O	-2.75556	-4.54693	-2.99139

N	-1.65695	-5.71496	-1.30102
C	-2.07247	-6.51287	-0.14914
H	-2.10795	-7.58633	-0.39937
N	-1.33035	-6.29280	1.06149
C	-0.08170	-6.93557	1.40358
N	1.10263	-6.35287	0.80963
C	2.06691	-5.71223	1.57543
O	2.03785	-5.52948	2.76885
N	3.10181	-5.34843	0.70674
C	4.40030	-4.99878	1.24694
N	4.90210	-3.70574	0.83475
C	4.75100	-2.56661	1.61197
O	4.23010	-2.49768	2.70015
N	5.35115	-1.50941	0.91190
C	5.77822	-0.34141	1.65172
N	5.40908	0.91963	1.03241
C	4.72501	1.88135	1.77187
O	4.03284	1.69032	2.74825
N	5.00633	3.11637	1.20127
C	4.49650	4.34683	1.74169
N	3.32654	4.88042	1.05089
C	2.17333	5.14474	1.78401
O	1.87529	4.64892	2.85354
N	1.43906	6.08675	1.09066
C	0.13764	6.51690	1.54717
N	-0.98576	5.81434	0.94868
C	-1.91340	5.17441	1.77085
O	-1.71657	4.79210	2.90944
N	-3.09692	5.07939	1.06739
C	-4.33661	4.64199	1.67276
N	-4.83690	3.36907	1.21698
C	-4.79064	2.22754	1.99417
O	-4.23121	2.11818	3.07142
N	-5.51449	1.24200	1.33847
C	-6.05239	0.12653	2.10242
N	-5.73728	-1.17394	1.57762
C	-4.70035	-1.94102	2.07303
O	-3.91559	-1.59982	2.94397
N	-4.73541	-3.16526	1.42850
C	-4.15561	-4.34012	2.05621
N	-3.13652	-4.98671	1.26371
C	-1.92275	-5.33002	1.86707
O	-1.48645	-4.89029	2.90682
C	-3.46216	-5.89911	0.16921
H	-4.21486	-6.64244	0.48073
C	-5.86553	-3.26203	0.50736
H	-6.59489	-4.00100	0.87890
C	-6.42143	-1.80471	0.47527
H	-7.51385	-1.74768	0.61617
C	-6.21528	1.78113	0.17033
H	-7.29420	1.87128	0.37806
C	-5.50385	3.14717	-0.04197
H	-6.19661	3.97504	-0.26887
C	-2.98911	5.57317	-0.28314
H	-3.82663	6.25001	-0.51982
C	-1.59932	6.27601	-0.29358
H	-1.66047	7.37641	-0.30776
C	2.02142	6.41120	-0.18642
H	2.03072	7.50101	-0.34758
C	3.44575	5.78318	-0.09139

H	4.24273	6.52422	0.08045	H	6.87562	-0.36329	1.78695
C	5.78501	3.01642	-0.00207	H	5.29050	-0.38607	2.63247
H	6.63694	3.71577	0.02089	H	5.29953	5.10089	1.72302
C	6.20996	1.51658	-0.02361	H	4.19734	4.15061	2.77755
H	7.28515	1.36132	0.16540	H	0.04082	7.59575	1.34869
C	6.08263	-1.99400	-0.24926	H	0.09867	6.33726	2.62721
H	7.17187	-1.97641	-0.07651	H	-5.10521	5.40659	1.48141
C	5.50488	-3.42615	-0.43565	H	-4.15946	4.56169	2.75055
H	6.26633	-4.18259	-0.68727	H	-5.62876	0.19436	3.11027
C	2.93422	-5.93324	-0.60909	H	-7.14925	0.22057	2.15868
H	3.70083	-6.70361	-0.79924	H	-4.96878	-5.05457	2.28027
C	-0.50349	-6.05282	-2.10088	H	-3.68172	-4.02400	2.99274
H	3.75315	-4.00562	-3.28569	H	-0.61515	-5.53176	-3.05887
H	4.82794	-5.14136	-2.40724	H	-0.47213	-7.14322	-2.27864
H	6.14123	-0.04842	-3.04561	C	-1.55879	1.72387	4.36607
H	7.43790	-0.18286	-1.81313	N	-1.02246	1.45870	3.01041
H	5.66338	5.12882	-1.62550	C	0.02779	2.19137	2.58214
H	4.69699	4.33674	-2.91416	C	0.57579	1.96557	1.33887
H	0.32428	5.88805	-3.06666	C	-1.57050	0.50076	2.23405
H	0.27414	7.33547	-2.00414	C	-1.05094	0.24039	0.98063
H	-3.69110	3.50869	-2.88672	C	0.04412	0.97199	0.50984
H	-4.76527	4.73115	-2.12554	C	0.63612	0.70894	-0.81918
H	-6.49987	-0.26051	-2.53375	C	1.06051	1.76745	-1.62960
H	-7.68785	-0.07985	-1.19804	C	1.60268	1.51085	-2.86939
H	-5.22146	-4.72222	-2.49380	N	1.73071	0.24129	-3.31087
H	-5.89126	-5.56257	-1.05461	C	2.29850	0.02247	-4.66153
H	-0.15152	-7.99432	1.11174	C	1.34268	-0.80579	-2.55345
H	0.04645	-6.85903	2.48951	C	0.79009	-0.59330	-1.30543
H	5.14392	-5.76012	0.96061	H	-1.89795	2.76143	4.39887
H	4.29345	-4.97841	2.33790	H	-0.75976	1.55223	5.09181

H	-2.39837	1.05651	4.55012
H	0.41604	2.95176	3.25156
H	1.44793	2.54304	1.05131
H	-2.41909	-0.04679	2.64009
H	-1.52292	-0.52750	0.37652
H	0.93287	2.80267	-1.33200
H	1.93990	2.30668	-3.52529
H	3.29399	0.47051	-4.68708
H	1.63933	0.49703	-5.39317
H	2.37199	-1.04753	-4.84453
H	1.48397	-1.80361	-2.96353
H	0.50209	-1.45877	-0.71779
